# Rituximab-induced long-term remission in childhood-onset, uncomplicated, frequently relapsing or steroid-dependent nephrotic syndrome: a randomized, placebo-controlled trial and a follow-up study

**DOI:** 10.1038/s41598-025-19214-0

**Published:** 2025-10-10

**Authors:** Kazumoto Iijima, Mayumi Sako, Tomoko Horinouchi, Tomoyuki Sakai, Tomohiko Yamamura, Riku Hamada, Yasufumi Ohtsuka, Seiji Tanaka, Koichi Kamei, Ryojiro Tanaka, Yuji Kano, Takuo Kubota, Yuko Shima, Tamaki Morohashi, Aya Inaba, Shuichiro Fujinaga, Masafumi Oka, Hiroshi Kaito, Akihide Konishi, China Nagano, Koichi Nakanishi, Kenji Ishikura, Shuichi Ito, Hidefumi Nakamura, Gian Marco Ghiggeri, Rintaro Mori, Takashi Omori, Kandai Nozu, Japanese Study Group of Kidney Disease in Children JSKDC10 Study, Kazumoto Iijima, Mayumi Sako, Tomoko Horinouchi, Tomoyuki Sakai, Tomohiko Yamamura, Riku Hamada, Yasufumi Ohtsuka, Seiji Tanaka, Koichi Kamei, Ryojiro Tanaka, Yuji Kano, Takuo Kubota, Yuko Shima, Tamaki Morohashi, Aya Inaba, Shuichiro Fujinaga, Masafumi Oka, Hiroshi Kaito, China Nagano, Koichi Nakanishi, Kenji Ishikura, Shuichi Ito, Hidefumi Nakamura, Kandai Nozu, Nana Sakakibara, Atsushi Kondo, Hideaki Kitakado, Chika Ueda, Toshihiro Sawai, Kazuna Yamamoto, Satoko Ichioka, Toshiki Masuda, Hiroshi Hataya, Ryoko Harada, Chikako Terano, Naoaki Mikami, Tomohiro Inoguchi, Kouki Tomari, Keiji Akamine, Shoichiro Shirane, Taishi Nada, Toru Kanamori, Chikako Kamae, Yosuke Inaguma, Yuhi Takagi, Toru Uchimura, Kazumoto Iijima, Kandai Nozu, Mayumi Sako, Motoshi Hattori, Nao Tsuchida, Mari Oba

**Affiliations:** 1 Hyogo Prefectural Kobe Children’s Hospital, Minatojimaminami-machi 1-6-7, Chuo-ku, Kobe, Hyogo 650-0047 Japan; 2 Department of Pediatrics, Kobe University Graduate School of Medicine, Kobe, Hyogo Japan; 3 Department of Advanced Pediatric Medicine, Kobe University Graduate School of Medicine, Kobe, Hyogo Japan; 4 Department of Clinical Research Promotion, Clinical Research Center, National Center for Child Health and Development, Tokyo, Japan; 5 Department of Pediatrics, Shiga University of Medical Science, Otsu, Shiga Japan; 6 Department of Nephrology and Rheumatology, Tokyo Metropolitan Children’s Medical Center, Fuchu, Tokyo, Japan; 7 Department of Pediatrics, Faculty of Medicine, Saga University, Saga City, Saga Japan; 8 Department of Pediatrics and Child Health, Kurume University School of Medicine, Kurume, Fukuoka Japan; 9 Division of Nephrology and Rheumatology, National Center for Child Health and Development, Tokyo, Japan; 10 Department of Nephrology, Hyogo Prefectural Kobe Children’s Hospital, Kobe, Hyogo Japan; 11 Department of Pediatrics, Dokkyo Medical University School of Medicine, Mibu, Tochigi, Japan; 12 Department of Pediatrics, Graduate School of Medicine, The University of Osaka, Suita, Osaka Japan; 13 Department of Pediatrics, Wakayama Medical University, Wakayama City, Wakayama Japan; 14 Department of Pediatrics, Nihon University School of Medicine, Tokyo, Japan; 15 Department of Pediatrics, Graduate School of Medicine, Yokohama City University, Yokohama, Kanagawa Japan; 16 Division of Nephrology, Saitama Children’s Medical Center, Saitama City, Saitama Japan; 17 Clinical and Translational Research Center, Kobe University Hospital, Kobe, Hyogo Japan; 18 Department of Child Health and Welfare (Pediatrics), Graduate School of Medicine, University of the Ryukyus, Nishihara, Okinawa Japan; 19 Department of Pediatrics, Kitasato University School of Medicine, Sagamihara, Kanagawa Japan; 20 Clinical Research Center, National Center for Child Health and Development, Tokyo, Japan; 21 Division of Nephrology, Dialysis, Transplantation, IRCCS Istituto Giannina Gaslini, Genoa, Italy; 22 Graduate School of Medicine, Kyoto University, Kyoto, Japan; 23 Department of Clinical Biostatistics, Graduate School of Medicine, Kyoto University, Kyoto, Japan; 24 Department of Pediatric Nephrology, Tokyo Women’s Medical University, School of Medicine, Tokyo, Japan; 25 Clinical Research Center, National Hospital Organization Headquarters, Tokyo, Japan; 26 Department of Clinical Data Science, Clinical Research & Education Promotion Division, National Center of Neurology and Psychiatry, Kodaira, Japan

**Keywords:** Rituximab, Uncomplicated frequently relapsing or steroid-dependent nephrotic syndrome, Children, Long-term remission, Diseases, Nephrology

## Abstract

Rituximab maintains remission of complicated frequently relapsing or steroid-dependent nephrotic syndrome (FRNS/SDNS) by depleting peripheral B cells, but most patients eventually experience relapses after B cell recovery. We performed a multicenter, double-blind, randomized, placebo-controlled trial to assess rituximab’s efficacy and safety for childhood-onset uncomplicated FRNS/SDNS (without prior treatment with glucocorticoid-sparing immunosuppressive agents) with a follow-up study to assess rituximab’s long-term effect after B cell recovery. Patients were randomly assigned to receive either rituximab (375 mg/m^2^, maximum 500 mg, once weekly for 2 weeks) or placebo. The primary endpoint was the relapse-free period. Of 43 randomized patients, 40 received the intervention (18 rituximab, 22 placebo). The relapse-free period during the 1-year trial was significantly longer in the rituximab vs. placebo groups (median: 285 vs. 81 days; *p* < 0.001). Infusion reactions were more frequent in the rituximab group (*p* < 0.001), with no difference in adverse events incidence between the groups. Interestingly, the follow-up study demonstrated markedly higher 3-year cumulative relapse-free survival probability without further treatments in the rituximab vs. placebo groups (38% vs. 9%). A mini-systematic review with meta-analyses supported the findings. Rituximab is effective and well-tolerated, potentially leading to long-term remission with substantially high rates after B cell recovery for childhood-onset uncomplicated FRNS/SDNS.

*Trial registration* JSKDC10, Clinical Trials Registry ID: jRCT1091220380; JSKDC10 follow-up study, Clinical Trials Registry ID: jRCT1050230024

**Supplementary Information:**

The online version contains supplementary material available at 10.1038/s41598-025-19214-0.

## Introduction

Idiopathic nephrotic syndrome is the most prevalent chronic glomerular disease affecting children. 80–90% of affected children achieve complete remission with steroid therapy, termed steroid-sensitive nephrotic syndrome^1^. However, 50–60% later develop frequently relapsing or steroid-dependent nephrotic syndrome (FRNS/SDNS)^1^. Long-term (6–12 months) alternate-day steroids or glucocorticoid-sparing immunosuppressive agents, such as alkylating agents, levamisole, calcineurin inhibitors, and mycophenolate mofetil, are recommended for children with FRNS/SDNS by recent guidelines^2,3^. However, long-term alternate-day steroid therapy needs periodic reassessment for tapering and discontinuing, along with long-term diligent monitoring for steroid toxicity^4^. In addition, more than half of the patients with this therapy experience treatment failure such as frequent relapses or severe steroid toxicity within 6 months^5^, and the remaining patients are highly likely to have relapses during the tapering and discontinuation process of steroids. Levamisole cannot be used in some countries, including Japan^1^. While calcineurin inhibitors and mycophenolate mofetil effectively prevent relapses during use, relapses are common upon discontinuation, leading to FRNS/SDNS recurrence^6,7^. Alkylating agents are inexpensive and produce long-term remissions in approximately one-third of patients, but are associated with fertility concerns^8^.

At least 20% of children with FRNS/SDNS experience frequent relapses and/or steroid dependence during or after treatment with steroid-sparing agents^10^, and about 30% with steroid-resistant nephrotic syndrome develop FRNS/SDNS post-remission with immunosuppressants^9^. We defined these conditions as complicated FRNS/SDNS (Table 1)^1,10^.

**Table 1:** Definitions.

Nephrotic syndrome	Severe proteinuria (≥ 40 mg/h/m^2^ in nocturnal urine) or morning urine protein/creatinine ratio ≥ 2.0 g/gCr and hypoalbuminemia (serum albumin ≤ 2.5 g/dL)
Remission	Negative morning urine protein dipstick for 3 consecutive days or morning urine protein/creatinine ratio < 0.2 g/gCr for 3 consecutive days
Date of confirmation of remission	Date when remission is confirmed at the study site
Steroid sensitivity	Remission within 4 weeks of starting daily prednisolone treatment at a dose of 60 mg/m^2^/day
Relapse	Any of the following conditions requiring prednisolone treatment:
	[1] Morning urine protein dipstick ≥ 3 + (or ≥ 300 mg/dL in quantitative urine protein test) for 3 consecutive days
	[2] Urine protein dipstick ≥ 2 + (or ≥ 100 mg/dL in quantitative urine protein test) and serum albumin ≤ 3.0 g/dL
Date of relapse	The first date of morning urine protein dipstick ≥ 3 + (or ≥ 300 mg/dL in quantitative urine protein test) for 3 consecutive days or date of urine protein dipstick ≥ 2 + (or ≥ 100 mg/dL in quantitative urine protein test) and serum albumin ≤ 3.0 g/dL (or date of diagnosis of relapse for the last 3 relapses before enrollment)
Frequent relapse	At least 2 relapses within 6 months of the first remission or at least 4 relapses within any 12-month period
Date of frequent relapse	Date of relapse meeting the definition of frequent relapse (date of the second relapse for at least 2 relapses within 6 months of the first remission or date of the fourth relapse for at least 4 recurrences within any 12 months)
Steroid dependence	Two consecutive relapses within 2 weeks after dose reduction or discontinuation of prednisolone
Date of steroid dependence	Date of the second relapse meeting the definition of steroid dependence
Steroid resistance	Failure to achieve remission despite at least 4 weeks of daily prednisolone treatment at a dose of 60 mg/m^2^/day
Date of steroid resistance	Date when failure to achieve remission despite 4 weeks of daily prednisolone treatment at a dose of 60 mg/m^2^/day is confirmed at the study site
Complicated frequently relapsing/steroid-dependent nephrotic syndrome	Patients who meet any of the following [1] to [4]:
	[1] The disease was diagnosed as frequently relapsing or steroid-dependent and is then again diagnosed as frequently relapsing or steroid-dependent after immunosuppressive therapy (e.g., cyclosporine, cyclophosphamide, mizoribine*)
	[2] The disease was diagnosed as frequently relapsing or steroid-dependent and is then again diagnosed as frequently relapsing or steroid-dependent during immunosuppressive therapy (e.g., cyclosporine, cyclophosphamide, mizoribine*)
	[3] The disease was diagnosed as steroid-resistant and is then diagnosed as frequently relapsing or steroid-dependent after immunosuppressive therapy (e.g., cyclosporine alone or in combination with methylprednisolone)
	[4] The disease was diagnosed as steroid-resistant and is then diagnosed as frequently relapsing or steroid-dependent during immunosuppressive therapy (e.g., cyclosporine alone or in combination with methylprednisolone)
	* Only when mizoribine is used in combination with other immunosuppressants
Uncomplicated frequently relapsing/steroid-dependent nephrotic syndrome	Frequently relapsing or steroid-dependent nephrotic syndrome that has not been treated with steroid-sparing immunosuppressive agents

Rituximab is a chimeric monoclonal antibody directed against cluster of differentiation 20, which induces peripheral B-cell depletion. Our previous multicenter, double-blind, placebo-controlled trial (RCRNS-01) revealed the efficacy and safety of rituximab up to at least 1 year for childhood-onset complicated FRNS/SDNS^10^. The approval of rituximab in Japan in 2014 and subsequent use in various countries reflected its established status as the standard treatment for complicated FRNS/SDNS^2,3,8,11^.

We defined FRNS/SDNS that has not been treated with glucocorticoid-sparing immunosuppressive agents as uncomplicated FRNS/SDNS to clearly distinguish it from complicated FRNS/SDNS (Table 1). Recently, rituximab has been used in European centers and elsewhere for children with uncomplicated SDNS^12^. Few trials have evaluated rituximab’s efficacy and safety for uncomplicated FRNS/SDNS in children^13,14^. Additionally, no randomized placebo-controlled trial has confirmed rituximab’s efficacy and safety for children with uncomplicated FRNS/SDNS. The primary objective of this study was to evaluate rituximab’s efficacy and safety over one year through a multicenter, double-blind, randomized, placebo-controlled trial (JSKDC10).

Rituximab’s potential to induce long-term remission in childhood-onset uncomplicated FRNS/SDNS remains unclear. In RCRNS-01 and its pharmacokinetic study (RCRNS-02), the majority of patients with complicated FRNS/SDNS eventually experienced relapses after peripheral B cell recovery, returning to a state of FRNS/SDNS, and only 3/51 (6%) patients maintained long-term remission^15^. However, in our recent JSKDC07 trial for complicated FRNS/SDNS, the duration from disease onset was shorter (6.1 vs. 8.0 years) vs. the RCRNS trials, disease activity was lower (no glucocorticoid-sparing immunosuppressive treatments at enrollment: 26% vs. 8%, respectively), and approximately 20% maintained long-term remission with rituximab monotherapy^6^. This suggests that rituximab might induce higher long-term remission rates in children with uncomplicated FRNS/SDNS, who likely have a shorter duration from onset and lower disease activity compared with those with complicated FRNS/SDNS. The secondary objective was to explore rituximab’s potential to induce long-term remission in childhood-onset uncomplicated FRNS/SDNS through a follow-up study and a mini-systematic review with meta-analyses.

## Methods

### Study design

**Fig. 1:**
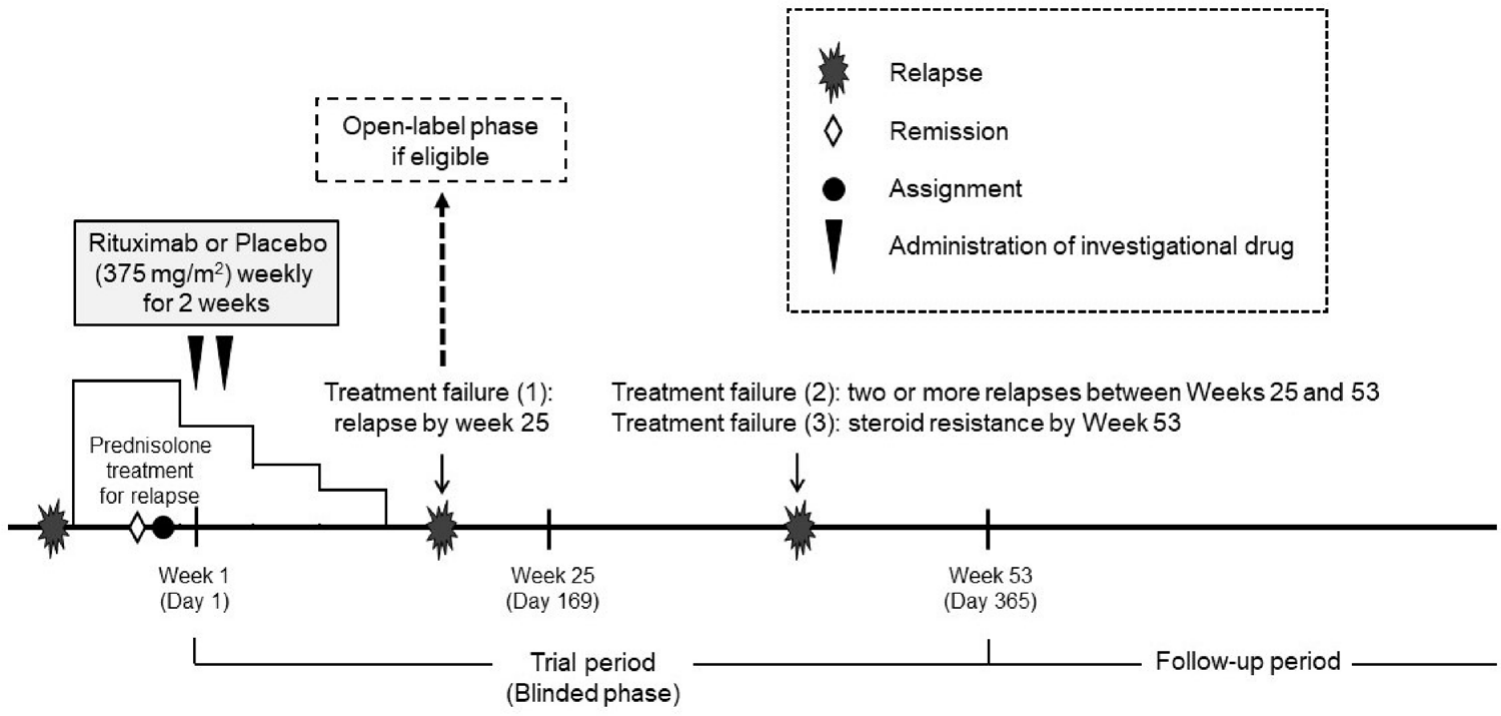
Study design. Candidates with a relapse of nephrotic syndrome were screened for eligibility to this study, and simultaneously they were treated with the standard prednisolone regimen for relapse. When the patients showed complete remission, the eligible patients were randomized into rituximab group or placebo group. When patients experienced treatment failure, their allocation code was urgently disclosed. If a patient with Treatment failure (1) was treated with the placebo, he or she could then choose to begin the standard immunosuppressive treatments deemed the best choice by the investigators or enter the open-label phase (rituximab treatment). Patients with Treatment failure (1) in the rituximab group and those with Treatment failure (2) or (3) in both groups were treated with the standard immunosuppressive treatments deemed the best choice by the investigators.

In the clinical practice recommendations by the International Pediatric Nephrology Association, published in 2022, long-term alternate-day steroid therapy is one recommended option for FRNS/SDNS^2^. However, as previously mentioned, long-term careful monitoring of steroid toxicity is necessary, and most patients experience relapses during the therapy and the tapering and discontinuation process^4,5^. Therefore, in Japan, many patient families and pediatric nephrologists do not prefer long-term alternate-day steroid therapy. In fact, the clinical practice guideline by the Japanese Society for Pediatric Nephrology does not specifically recommend long-term alternate-day steroid therapy for FRNS/SDNS^16^. A different guideline published in 2021 stated that for children with FRNS/SDNS without steroid toxicity, the same steroid regimen may be employed in subsequent relapses^3^. Indeed, in Japan, it is not uncommon in clinical practice to avoid the long-term alternate-day steroid therapy in the early phase of FRNS/SDNS with minimal or mild steroid toxicity and to try the standard steroid treatment for relapse once again in the modest hope of achieving long-term remission after the treatment, before switching to glucocorticoid-sparing immunosuppressive therapy, which may have various side effects. Consequently, participants in this trial were set to receive the standard steroid treatment for relapse at enrollment and during the trial, without the long-term alternate-day steroid therapy, but with a rescue program for patients in the control group with early relapse (treatment failure (1), see “Intervention” in detail) to receive rituximab (open-label phase) or conventional glucocorticoid-sparing immunosuppressive therapy (Fig. 1).

In our previous trial (RCRNS-01), infusion reactions were diagnosed in 79% of patients in the rituximab group and 54% in the placebo group, resulting in no statistically significant difference in the incidence between the groups^10^. However, approval of rituximab for complicated FRNS/SDNS in Japan in 2014 and its widespread use among pediatric nephrologists might have improved the diagnostic accuracy of infusion reactions. Therefore, to accurately assess the incidence of infusion reactions, we designed the present trial as a multicenter, double-blind, randomized, placebo-controlled, parallel-group trial (JSKDC10, Clinical Trials Registry ID: jRCT1091220380; Date of Registration: 6 September 2018) (Fig. 1).

The design of this trial was reviewed and approved by the Pharmaceuticals and Medical Devices Agency on 12 January, 2018. This study was performed in accordance with the Declaration of Helsinki. All experimental protocols were approved by the institutional review boards of Dokkyo Medical University Hospital, Nihon University Itabashi Hospital, National Center for Child Health and Development, Tokyo Metropolitan Children’s Medical Center, Yokohama City University Medical Center, Shiga University of Medical Science Hospital, The University of Osaka Hospital, Kobe University Hospital, Hyogo Prefectural Kobe Children’s Hospital, Wakayama Medical University Hospital, Kurume University Hospital, Saga University Hospital and Saitama Children’s Medical Center.

### Participants

Written informed consent was obtained from the legal guardians/parents of all patients (and assent of patients aged ≥ 7 years). The definition of FRNS, SDNS and associated parameters for this study are detailed in Table 1. The major inclusion criteria and exclusion criteria were as follows (JSKDC10 Protocol ver. 3.4 (Supplementary Material)):

Inclusion criteria:

1. Idiopathic nephrotic syndrome diagnosed in accordance with the criteria of the International Study of Kidney Disease in Children;
2. Idiopathic nephrotic syndrome onset before 18 years of age;
3. Diagnosis of FRNS or SDNS, with confirmation of relapse dates before group assignment;
4. No prior treatment with glucocorticoid-sparing immunosuppressive agents except glucocorticoids.

Exclusion criteria:

1. Diagnosis of steroid-resistant nephrotic syndrome;
2. Diagnosis of nephritic or secondary nephrotic syndrome;
3. History of severe infections
4. Previous receipt of any type of monoclonal antibody.

### Intervention

Zenyaku Kogyo Co., Ltd. provided clinical trial-grade rituximab and placebo manufactured by Genentech. Candidates with a relapse of nephrotic syndrome were screened for eligibility to this study, and simultaneously they were treated with the standard prednisolone regimen for relapse. When the patients showed complete remission, the eligible patients were randomized into rituximab group or placebo group. Patients received the first dose of their allocated drug within 2 weeks post-randomisation. Patients receiving rituximab received an intravenous dose of 375 mg/m^2^ (maximum 500 mg) weekly for 2 weeks. Patients receiving placebo received a matching placebo dose (Fig. 1).

The rationale for the dosage and administration of rituximab, the pre-treatment to prevent infusion reactions, the criteria for discontinuing the study drug, and prednisolone regimens for relapses at enrolment and during the trial are described in the full protocol (JSKDC10 Protocol V3.4 with a Comparative List of Changes in “Supplementary Material”).

Patients were monitored for 1 year unless they withdrew from the trial. Patients in the blinded phase were considered to have experienced treatment failure with relapse by Day 169 (Week 25) (treatment failure (1)); two or more relapses between Weeks 25 and 53 (treatment failure (2)); or steroid resistance by Week 53 (treatment failure (3)). Urgent disclosure of allocation codes occurred with treatment failure. Placebo group patients with treatment failure (1) could opt for standard glucocorticoid-sparing immunosuppressive treatment, as best determined by the investigators, or enter the open-label phase (rituximab treatment). Rituximab group patients with treatment failure (1) and those with treatment failure (2) or (3) in both groups received standard glucocorticoid-sparing immunosuppressive treatment, as best determined by the investigators (Fig. 1).

Investigators at each institution were required to monitor patients who remained in remission throughout the 1-year observation period of either phase, without treatment for relapse prevention as much as possible, until relapse occurred after the trial period.

### Outcomes

The primary endpoint was the relapse-free period (time to relapse), defined as the time from assignment to the first relapse after starting the intervention during the blinded phase. Secondary endpoints comprised the time to treatment failure, daily steroid dose, and the period of peripheral B-cell depletion during the blinded phase. Other endpoints included the relapse-free period during the open-label phase. Adverse events were recorded and assessed using the Common Terminology Criteria for Adverse Events during both the blinded and open-label phases.

### Sample size

In a non-Japanese randomized controlled trial involving pediatric patients with uncomplicated SDNS, rituximab plus concurrent steroids showed a 1-year relapse-free rate of 0.66, compared with 0 in the control (steroid) group^13^. Hazard were 0.42 and 4.60, respectively, under an exponential distribution assumption. For a desired hazard ratio (HR) of 0.09 with 5% significance and 80% power, 13 patients per group were needed. Adjusting for varying relapse-free rates of 0.50–0.40 in the rituximab group, required sample sizes were 15–17 per group. In our previous rituximab trial (RCRNS-01) for complicated FRNS/SDNS, which showed a 1-year relapse-free rate of 0.44 (rituximab) vs. 0.04 (placebo) with an HR of 0.26, 24 patients per group were required^10^. Because some patients may become complicated, we considered it appropriate to refer to the results of the RCRNS-01 trial to determine the sample size. Consequently, the target sample size was 20 patients per group, considering these uncertainties.

### Randomization and blinding

After remission of the relapse at enrollment, patients were randomly allocated to either rituximab or placebo in an approximate 1:1 ratio, using the permutation block method stratified with FRNS or SDNS.

All patients, their guardians, treating investigators, outcome-assessing physicians, and investigators including the biostatistician were blinded to the group assignments. Investigators and patients (or guardians) were unaware of peripheral blood B-cell counts, monitored centrally to ensure blinding. Additionally, all investigators remained unaware of assignments until all data were finalized after study completion. However, investigators could request disclosure of a patient’s allocation code in urgent cases, such as (1) serious adverse event risking death, (2) essential treatment needed owing to a serious adverse event, or (3) treatment failure.

### Statistical analyses

To evaluate efficacy under the blinded trial, the full analysis set (FAS, all enrolled patients who received at least one dose of the investigational drug and had necessary data) was used based on intention to treat. The primary endpoint, time to relapse, was summarized by median times for each group using the Kaplan–Meier method. The primary analysis of this endpoint was the stratified log-rank test to compare endpoint distributions, with a significance level of 0.05. Cox proportional hazards models including stratification factors were used to estimate HRs and 95% confidence intervals (CIs) for the endpoint. Secondary endpoints for time to event were analyzed similarly. Daily steroid doses during the blinded phase were compared using analysis of covariance, including the stratification factor.

For the unblinded trial, the unblinded analysis set (patients entering the open-label phase who received at least one dose of rituximab) was used.

The safety analysis set comprised patients who received at least one dose of the study drug during the blinded phase.

### Follow-up study

To assess the long-term prognosis of patients enrolled in the JSKDC10 trial (blinded and open-label phases), we retrospectively examined relapses, serum immunoglobulin G (IgG) concentrations, and other parameters until 31 December, 2022. Questionnaires were sent to investigators at each institution (Clinical Trials Registry ID: jRCT1050230024). The primary outcome was the relapse-free period by 31 December, 2022 (JSKDC10 follow-up study protocol in “Supplementary Material”).

Long-term remission was defined as sustained absence of relapse without immunosuppressive treatment for > 3 years post-rituximab, based on data indicating that children with idiopathic nephrotic syndrome who remain relapse-free for 18–24 months after initial remission have minimal risk of future relapse^17^, and data in our previous trials of rituximab for childhood-onset complicated FRNS/SDNS, showing that patients who maintained remission for > 2–3 years following rituximab treatment demonstrated a low risk of subsequent relapse^6,15^.

### Mini-systematic review

After completing our study, we performed a mini-systematic review of randomized controlled trials on childhood steroid-sensitive nephrotic syndrome, comparing rituximab with placebo or steroid monotherapy to assess long-term relapse-free survival. We searched the Cochrane Central Register of Controlled Trials (CENTRAL), MEDLINE, and EMBASE (all as of 21 September 2023), as well as the reference lists of review articles, relevant studies, and guidelines. We also contacted relevant individuals for unpublished data. Outcome measures were the numbers of children with and without relapse at 12, 24, and ≥ 36 months (See Supplementary Mini-Systematic Review in “Supplementary Material”).

## Results

### Patients

Fifty-four patients from 13 pediatric nephrology centers in Japan underwent eligibility assessment, with 43 enrolled and randomly allocated to receive either rituximab or placebo between 27 November, 2018 and 3 December, 2020. The observation period for the final enrolled patient concluded on 28 March, 2022.

**Fig. 2:**
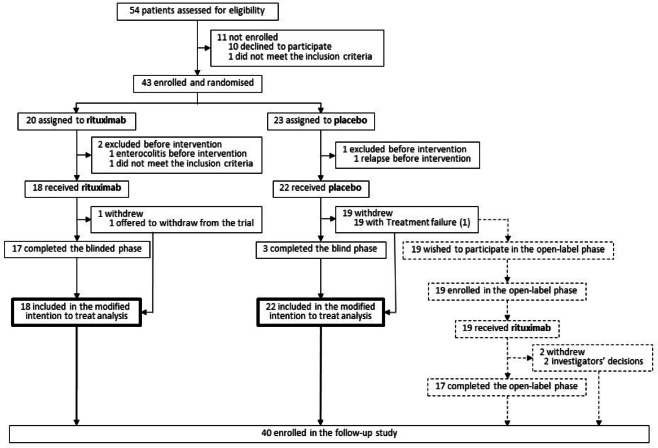
Flow diagram. The solid lines indicate the blinded phase, and the dashed lines indicate the open-label phase.

Of the 43 randomized patients, three eliminated from the FAS because they did not receive the investigational drug at all, and 40 received the assigned intervention and were included in the FAS. At the time of enrolment, all 40 patients were receiving the standard steroid treatment for relapse. Among them, 17 patients did not exhibit any steroid-related adverse events. The remaining 23 patients showed steroid-related adverse events, such as hypertension; however, these events were mild, controllable with medications, and transient. Eighteen patients received rituximab; 22 received placebo. No patient withdrew before the initial relapse. One patient from the rituximab group withdrew from the trial after treatment failure (1) on the guardian’s request. Nineteen patients from the placebo group experienced treatment failure (1), leading to their withdrawal from the blinded phase and subsequent participation in the open-label phase (Fig. 2).

Patients in the rituximab group had a higher body weight vs. those in the placebo group; baseline characteristics between the groups were otherwise similar. Approximately 50% and 75% of the patients in both groups were enrolled within 6 and 12 months from disease onset, respectively (Table 2).

**Table 2:** Baseline characteristics of children with uncomplicated frequently relapsing or steroid-dependent nephrotic syndrome.

	Rituximab (N = 18)	Placebo (N = 22)
Age at enrollment (years)	7 (4)	5 (4)
Age at disease onset (years)	5 (3)	4 (3)
Disease duration (months)	14 (29)	12 (12)
6 months or earlier	9 (50%)	11 (50%)
From 6 to 12 months	5 (28%)	5 (23%)
12 months or more	4 (22%)	6 (27%)
Male/Female	17/1	18/4
Race		
Asian	17 (94%)	22 (100%)
American Indian or Alaskan Native/Asian	1 (6%)	0 (0)
Height (cm)	117.6 (22.0)	107.6 (22.0)
Weight (kg)	26.6 (11.9)*	19.9 (11.1)
Systolic blood pressure (mmHg)	109 (11)	105 (12)
Diastolic blood pressure (mmHg)	67 (9)	62 (11)
Serum creatinine (mg/dl)	0.35 (0.10)	0.33 (0.14)
Estimated glomerular filtration rate (ml/min/1.73 m^2^)	123 (24)	117 (15)
Serum total protein (g/dl)	6.1 (0.31)	6.2 (0.3)
Serum albumin (g/dl)	3.7 (0.3)	3.9 (0.3)
Urinary protein to creatinine ratio (mg/mg)	0.10 (0.07)	0.08 (0.04)
Peripheral B cell count (/μl)	692 (505)	604 (424)
Type of nephrotic syndrome		
Frequently-relapsing nephrotic syndrome	11 (61%)	16 (73%)
Two or more relapses within 6 months after first remission	8 (44%)	10 (46%)
Four or more relapses within any 12-month period	3 (17%)	6 (27%)
Steroid-dependent nephrotic syndrome	7 (39%)	6 (27%)

Data are presented as mean (standard deviation) or n (%).

*Body weight in the rituximab group was significantly higher than that in the placebo group.

### Efficacy analyses

#### Primary analysis

**Fig. 3:**
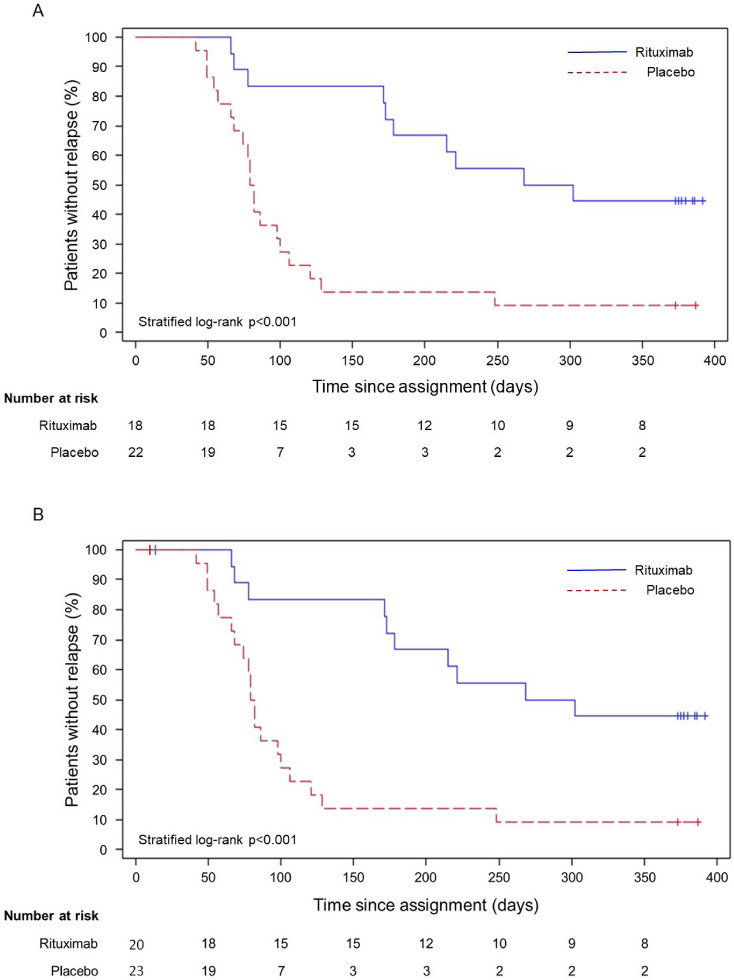
Kaplan–Meier curves for the primary outcome. (**A**) The relapse-free period during the trial was markedly longer in the rituximab group compared with that in the placebo group (median: 285 vs. 81 days, hazard ratio (95% confidence interval): 0.27 (0.12–0.59); *p* < 0.001). (**B**) Post-hoc analysis including three patients excluded from FAS. The data of three patients excluded from FAS were censored at the time an event occurred that warranted their exclusion from the FAS. The Kaplan–Meier curves of the primary analysis, which included the three cases excluded from the FAS, matched those of the original analysis exactly, except for the vertical bars indicating censoring at day 9 (placebo, 1 patient) and day 13 (rituximab, 2 patients), respectively and the number at risk at time 0.

At the end of the 1-year observation, 10 patients in the rituximab group and 20 in the placebo group had experienced relapse. The relapse-free period was markedly longer in the rituximab group (median (95%CI): 285 days (173–not reached)) vs. the placebo group (81 days (66–100)); HR: 0.27, 95%CI: 0.12–0.59; stratified log-rank test, *p* < 0.001 (Fig. 3A, Table 3a)). Note that the results of a post-hoc analysis including three patients excluded from the FAS matched those of the original analysis when these data were treated as censored at the time an event occurred that warranted their exclusion (Fig. 3B, Table 3b).

**Table 3:** a: Primary analysis: Time to relapse during the blinded phase. b: Post-hoc analysis including three patients excluded from FAS.

	No. of patients	No. of patients with relapse	No. of patients censored	Median (95%CI) (days)	Hazard ratio (95%CI)	Stratified Log-Rank test
a						
Rituximab	18	10	8	285 (173–not reached)	0.27 (0.12–0.59)	*P* < 0.001
Placebo	22	20	2	81 (66–100)		
b						
Rituximab	20	10	10	285 (173–not reached)	0.27 (0.12–0.59)	*P* < 0.001
Placebo	23	20	3	81 (66–100)		

95% CI = 95% confidence interval.

#### Secondary endpoint analyses

Analyses of secondary endpoints revealed treatment failure in 8 and 19 patients: rituximab vs. placebo groups, respectively. The time to treatment failure was longer in the rituximab vs. placebo groups (median (95%CI): not reached (175-not reached) vs. 84 days (70–102), respectively; HR: 0.24, 95%CI: 0.10–0.57; Supplementary Fig. S1, Supplementary Table S1).

The daily steroid dose post-randomisation was lower in the rituximab vs. placebo groups (mean (standard deviation): 0.36 (0.22) mg/kg/day vs. 0.89 (0.37) mg/kg/day, respectively; *p* < 0.001).

Peripheral blood B-cell depletion (< 5/µl) was observed in 15/18 patients in the rituximab group, all of whom recovered to ≥ 5/µl by Day 179. The median peripheral B-cell depletion period was 113 days (95%CI: 55–120). By Day 365, peripheral blood B cell counts had returned to levels comparable to those before rituximab administration (Day 1 vs. Day 365, mean (standard deviation): 692 (505)/µl vs. 615 (560)/µl) (Supplementary Table S2).

#### Other analyses

The HRs for relapse (rituximab vs. placebo) in the per-protocol set analysis (sensitivity analysis) and in the FRNS and SDNS subgroups were similar to those for the primary analysis (Supplementary Tables S3–S5). A post-hoc analysis revealed that the body weight discrepancy between the groups did not influence the primary outcome (weight-adjusted hazard ratio, 0.29 [95% CI: 0.12 to 0.68], Supplementary Table S6; unadjusted hazard ratio, 0.27 [95% CI: 0.12 to 0.59], Table 3a).

### Safety profile

Throughout the blinded phase, the incidence of adverse events was not statistically or clinically different between the rituximab vs. placebo groups (100% (95% CI 82–100) vs. 86% (95% CI 66–97), respectively; *p* = 0.24; Supplementary Table S7). Most adverse events were grade ≤ 3 and primarily grade 1 or 2 (Supplementary Table S8). Notably, no serious adverse events occurred in the rituximab group; one patient in the placebo group experienced a serious adverse event and recovered.

The incidence of infusion reactions was markedly higher in the rituximab vs. placebo groups (61% (95% CI 36–83) vs. 9% (95% CI 1–29), respectively; *p* < 0.001; Supplementary Table S7). However, all infusion reactions were classified as grade 1 or 2 (Supplementary Table S9) and resolved within 24 h.

Although more patients in the rituximab group had infections requiring treatment vs. the placebo group, this difference was not statistically significant (Supplementary Table S10). However, in the rituximab group, the rate of infections requiring treatment was higher during the B-cell depletion period compared with outside this period (2.1 vs. 1.2/person-years, respectively; *p* = 0.003; Supplementary Table S11).

No patients experienced decreased serum IgG concentrations during the blinded phase.

### Open-label phase

At the end of the 1-year observation, 12/19 patients had relapsed. The median relapse-free period was 249 days (95% CI 62–not reached). There were 167 adverse events among the 19 patients (100%, 95% CI 82%–100%). Infusion reactions were observed in 14 patients (74%, 95% CI 49%–91%). Four of 19 patients (21%, 95% CI: 6–46%) experienced the serious adverse event of hypoalbuminemia; all were classified as grade 3, and all patients recovered.

One patient developed a mild (grade 1) decrease in serum IgG concentrations, which resolved within 2 months.

### Follow-up study

**Fig. 4:**
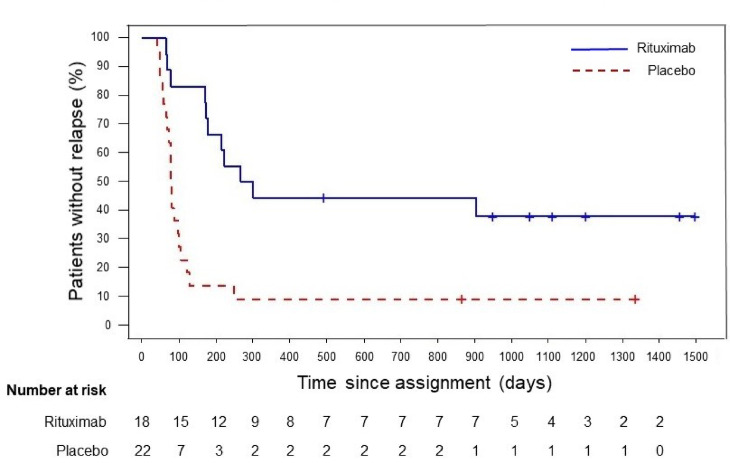
Kaplan–Meier curves during the blinded phase and follow-up period. The relapse-free period including the follow-up period was still longer in the rituximab group than in the placebo group (HR (95% CI): 0.28 (0.13–0.61)). The cumulative 2- and 3-year relapse-free survival probability in the rituximab group was 44% (95% CI 22–65%) and 38% (95% CI 17–60%), respectively, whereas in the placebo group, both probabilities were 9% (95% CI 2–25%).

In the follow-up study, we retrospectively obtained information from all participants who were in remission at the end of the trial to investigate long-term outcomes. The median observation period from randomization was 1044 days (758–1495 days). Patients who remained in remission during the trial were treatment-free until relapse. The cumulative 2- and 3-year relapse-free survival probabilities in the rituximab group were 44% (95% CI: 22–65%) and 38% (95% CI: 17–60%), respectively; in the placebo group, both probabilities were 9% (95% CI: 2–25%) (Fig. 4). Although the data quality of the trial and the follow-up study is not equivalent, the follow-up study showed a longer relapse-free period in the rituximab vs. placebo groups (HR (95%CI): 0.28 (0.13–0.61)). For the 19 patients treated with rituximab in the open-label phase, the 2- and 3-year probabilities were both 31% (95% CI 11–53%). In 37 rituximab-treated patients (18 in the blinded phase and 19 in the open-label phase), the 2- and 3-year probabilities were 38% (95% CI 22–53%) and 33% (95% CI 18–50%), respectively (Supplementary Fig. S2A and S2B).

During the follow-up, one patient experienced a mild, sustained decrease in serum IgG concentration without symptoms. Another developed Kikuchi–Fujimoto disease (subacute necrotizing lymphadenopathy), recovered with temporary steroid treatment, and had no recurrence of the disease or nephrotic syndrome relapse (data were censored when the steroid was administered).

### Mini-systematic review

**Fig. 5:**
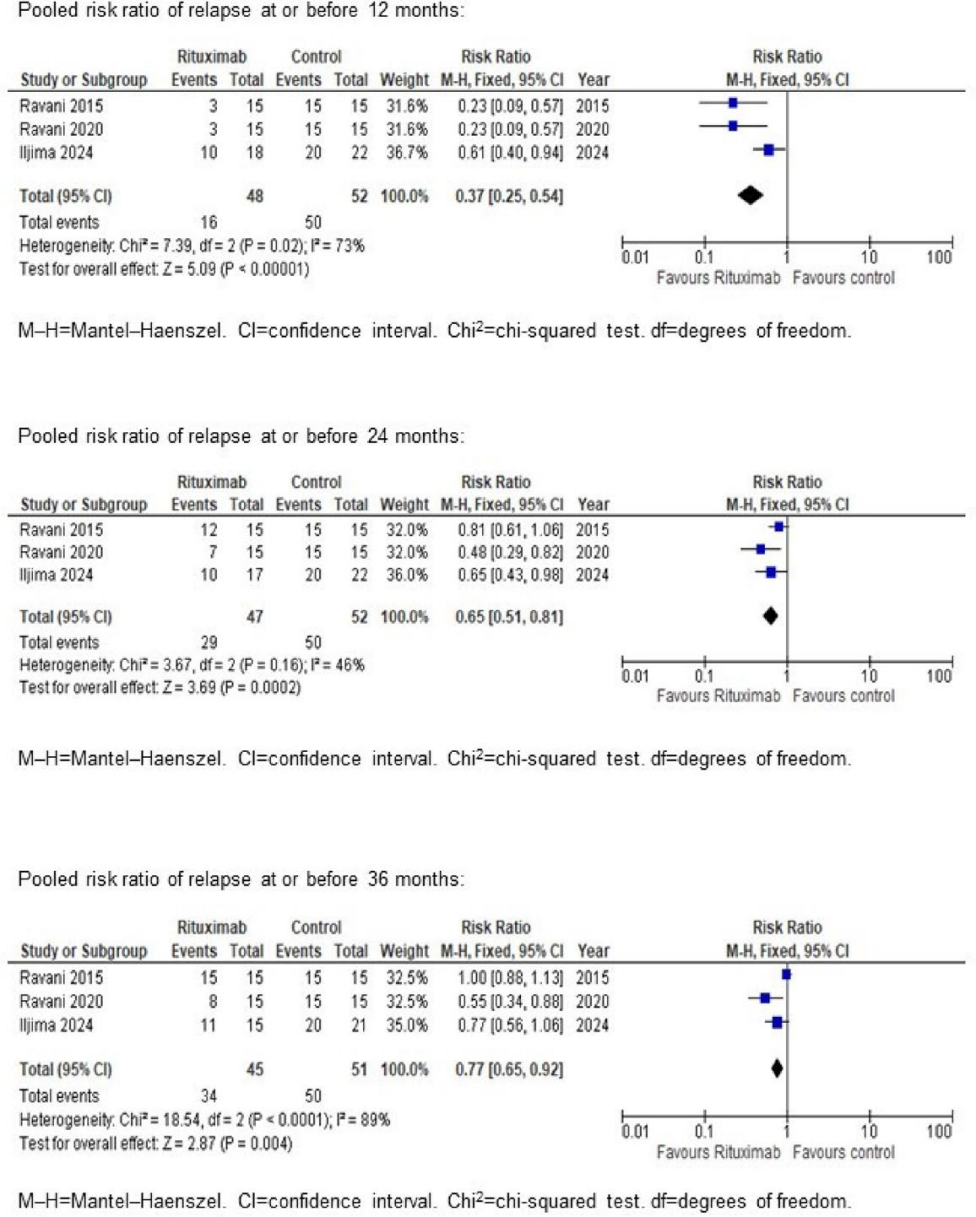
Meta-analyses of the three trials to evaluate the outcomes at 12, 24, and 36 months after the intervention. All of the pooled risk ratios were obtained from meta analyses yielding the fixed-effect models since the analyses include only three trials from two research teams (details of the explanation have been included in the supplementary material (Supplementary mini-systematic review). Meta-analyses of the three trials showed marked beneficial effects of rituximab in children with uncomplicated FRNS/SDNS during up to 36 months of follow-up.

We identified 1202 references. After applying pre-set criteria, two studies remained, both by the same author team, targeting similar populations (Supplementary Mini-Systematic Review, Supplementary Fig. S3, Supplementary Table S12). The studies were of fair quality, with insufficient data to assess rituximab’s long-term effects. Collaborating with the authors, we obtained long-term outcomes from the two trials and performed meta-analyses of all three studies, including our study for 12, 24, and 36 months. Meta-analyses using the fixed effect model showed marked benefits of rituximab in children with uncomplicated FRNS/SDNS for up to 36 months (Fig. 5) with a substantial level of statistical heterogeneity (I^2^-statistics ranges between 46–89%). Results using the random effect model were consistent with those of the fixed effect model (Supplementary Fig. S4).

## Discussion

This is the first double-blind, randomized, placebo-controlled trial to confirm the efficacy and safety of rituximab for childhood-onset uncomplicated FRNS/SDNS. Our findings showed that rituximab effectively prolonged the relapse-free period during the 1-year trial period among patients with childhood-onset uncomplicated FRNS/SDNS. Furthermore, our follow-up study indicated that rituximab might lead to long-term remission after B-cell recovery in these patients at a notably higher rate than in our previous trials for childhood-onset complicated FRNS/SDNS (6% in RCRNS-01&02^15^ and approximately 20% in JSKDC07^6^).

The placebo-controlled design demonstrated that infusion reactions were significantly more prevalent in the rituximab vs. placebo groups. All reactions were mild and transient, and other adverse events were generally mild during the trial. During follow-up, two patients experienced previously reported adverse events associated with rituximab^18–20^, both of which were well-tolerated. These findings affirm rituximab’s efficacy and tolerability in children with uncomplicated FRNS/SDNS, although vigilant monitoring for potential adverse events is warranted.

The long-term efficacy of rituximab for uncomplicated FRNS/SDNS is debated. Three years post-treatment, our trial showed a cumulative relapse-free survival probability of 38% for the rituximab group. Follow-up studies from two European trials on high-dose (Ravani et al. 2015)^13^ and very low-dose SDNS (Ravani et al. 2020)^21^ showed differing results. No patients in the high-dose group remained in remission after 3 years, compared with 7 of 15 patients in the low-dose group did (Fig. 5). Another follow-up study from a South Asian trial (RITURNS) found all rituximab-treated children relapsed within 27 months^22^.

Several potential explanations may account for these discrepancies. First, cohorts with lower disease activity may achieve long-term remission with rituximab. In Ravani et al.’s (2020) study, approximately half the patients achieved long-term remission for at least 3 years with a cumulative steroid dose < 0.4 mg/kg/day indicating lower disease activity. However, the cumulative steroid dose in our trial (mean: 0.89 mg/kg/day, based on the placebo group’s dosage during the trial period) was similar to studies with lower long-term remission rates (0.57 mg/kg/day in Ravani et al. (2020) and 0.65 mg/kg/day in RITURNS), suggesting disease activity alone does not explain remission rate variations. Second, the nephrotic syndrome type (SDNS or FRNS) may affect long-term prognosis; however, our study showed similar remission rates between FRNS and SDNS patients (Supplementary Fig. S5). Therefore, the disease type likely doesn’t explain the differences. Finally, variations in the duration between nephrotic syndrome onset or FRNS/SDNS diagnosis and rituximab treatment may be significant. In Ravani et al.’s (2015) study and RITURNS, the duration from nephrotic syndrome onset averaged approximately 2.5 years, compared with 14 months (1.2 years) in our study, with half of our patients enrolled within 6 months and 78% within 12 months (Table 2). In Ravani et al.’s (2015) study, patients received high-dose alternate-day steroids for at least 7 months before rituximab, and in RITURNS, 85% received high-dose alternate-day steroids for up to 6 months before the trial (Basu B, NRS Medical College and Hospital, Kolkata, personal communication). In contrast, our study’s mean interval for rituximab initiation from FRNS/SDNS diagnosis was 29 days (maximum: 39 days) in the blinded phase and 125 days (maximum: 162 days) in the open-label phase. Thus, earlier rituximab treatment in our trial may have influenced long-term remission rates. Recently, a prospective, multicenter, open-label, single-arm trial conducted in China demonstrated that early rituximab add-on therapy in children with initial episodes of steroid-sensitive nephrotic syndrome induced a higher 24-months relapse-free survival^23^. Although considering that 25%–30% of children with steroid-sensitive nephrotic syndrome achieve long-term remission solely through initial steroid therapy^17^ and that rituximab has potential severe side effects, ethical concerns exist about administering rituximab to this group^24^ However, the results support the above our hypothesis.

This study has several limitations. The main limitation is that the current trial did not compare rituximab with other glucocorticoid-sparing immunosuppressive drugs. The sample size for this study was determined statistically for the primary analysis of the primary endpoint, therefore, the calculated sample size of 40 may be small for the evaluation of other endpoints, especially for subgroup analyses. Other limitations include that the follow-up study after the trial was retrospective with irregular monitoring of serum IgG concentrations. However, patients who remained in remission during the trial period were confirmed treatment-free until relapse during follow-up. Therefore, we consider the integrity of the data regarding the relapse-free period to be preserved, though the data quality of the trial and the follow-up study is not equivalent. Additionally, the majority of participants were Asian, and the follow-up period was relatively short. Nonetheless, the results, including those in the open-label phase and the meta-analyses incorporating the two European studies and our study, strongly support the short- and long-term clinical usefulness of rituximab for patients with childhood-onset uncomplicated FRNS/SDNS (Supplementary Fig. S2B and Fig. 5), though a substantial level of statistical heterogeneity, as well as clinical heterogeneity reported above, warranted careful considerations when applying the findings in clinical contexts of the management.

The relationship between recently discovered anti-nephrin antibodies^25–27^ and response to rituximab was unclear in the present study. Fornoni et al. reported that rituximab not only recognizes CD20 on B lymphocytes, but might also bind sphingomyelin phosphodiesterase acid-like 3b (SMPDL-3b) protein and regulate acid sphingomyelinase activity, resulting in the prevention of disruption of the actin cytoskeleton and apoptosis of podocytes. Then, they concluded that rituximab might prevent recurrent FSGS by modulating podocyte function in an SMPDL-3b-dependent manner^28^. However, because ofatumumab, a human anti-CD20 monoclonal antibody that does not appear to bind to SMPDL-3b, is almost as effective as rituximab in pediatric nephrotic syndrome^29^, the main mechanism of action of rituximab, at least in pediatric nephrotic syndrome, appears to be the reduction in the production of autoantibodies such as anti-nephrin antibodies and/or changes in related immune pathways, by depleting B cells. Further studies are required to investigate the relationship between autoantibodies including anti-nephrin antibodies and clinical response to rituximab.

Based on the results of the JSKDC10 trial (the present trial), the Ministry of Health, Labour and Welfare of Japan approved the use of rituximab for uncomplicated FRNS/SDNS on March 27, 2025. Of note, ethical concerns for the use of rituximab in relatively early-stage FRNS/SDNS who have not been treated with glucocorticoid-sparing immunosuppressive agents and for the study design did not arise during the regulatory review process. Furthermore, a new investigator-initiated clinical trial is currently underway to evaluate the efficacy and safety of rituximab in patients who are considered to carry a high risk of progression to FRNS/SDNS (patients experience relapse within six months after achieving initial remission). This multicenter, open-label, randomized controlled trial (JSKDC12 trial, Trial ID: jRCT2051240106), whose protocol was approved by the Japanese regulatory agency, the Pharmaceuticals and Medical Devices Agency, is being conducted to address this clinical question. In parallel with JSKDC12, additional prospective studies are planned to investigate the relationship between changes in anti-nephrin antibody titers and relapse, as well as long-term prognosis, quality of life, and cost-effectiveness. As described above, efforts are underway in Japan to develop the use of rituximab for earlier stages of childhood nephrotic syndrome.

In conclusion, rituximab is effective and well-tolerated, potentially leading to long-term remission with substantially high rates after B cell recovery for childhood-onset uncomplicated FRNS/SDNS. Although further investigation is required, rituximab may be a first-line treatment for children with uncomplicated FRNS/SDNS, particularly those in the early disease stage or with mild-to-moderate disease activity.

## Supplementary Information

Below is the link to the electronic supplementary material.

Supplementary Material 1

## Data Availability

The study protocols are provided in the Supplementary Material. Anonymized participant data will be made available when the review of study results by the Pharmaceutical and Medical Devices Agency (PMDA), the governmental agency in Japan in charge of reviewing drugs and medical devices, overseeing post-market safety, and providing relief for adverse health effects, is complete, upon requests directed to the corresponding author, Kazumoto Iijima, MD, PhD. Proposals will be reviewed and approved by the sponsor, investigators, and collaborators based on scientific merit. After approval of a proposal, data can be shared through a secure online platform after signing a data access agreement.
