# Supplementary material for: Rituximab-induced long-term remission in childhood-onset, uncomplicated, frequently relapsing or steroid-dependent nephrotic syndrome: a randomized, placebo-controlled trial and a follow-up study
Source: Sci Rep. 2025 Oct 10;15:34306. doi: 10.1038/s41598-025-19214-0 (PMC12514064; doi:10.1038/s41598-025-19214-0)
Supplement: Supplementary file 1 — Supplementary Material 1 [file 41598_2025_19214_MOESM1_ESM.pdf]

**Supplementary Material**

**Supplement to: Iijima K, et al. Rituximab-induced long-term remission in childhood-onset, uncomplicated, frequently relapsing or steroid-dependent nephrotic syndrome: a randomized, placebo-controlled trial and a follow-up study.**

**Table of Contents**

Supplementary Mini-Systematic Review…………………………………………………………...... 3
Supplementary Tables……………………………………………………………………………….... 7
Supplementary Figures………………………………………………………………………………… 22
JSKDC10 Protocol v3.4 with a Comparative List of Changes……..……………………………… 28
JSKDC10 Follow-up Study Protocol……..……………………………..................................... 183

**Supplementary Mini-Systematic Review: Rituximab versus placebo or control regarding long-term outcomes in children with relapsing steroid-sensitive nephrotic syndrome without prior treatment with steroid-sparing agents**

## BACKGROUND

Clinically, one of the most critical research questions in the management of children with steroid-sensitive nephrotic syndrome (SSNS), especially for frequently relapsing or steroid-dependent nephrotic syndrome (FRNS/SDNS), is to seek the best strategy to maintain steroid-free and relapse-free survival for as long as possible. Rituximab appears to be a major non-corticosteroid immunosuppressive medication for SSNS in children and is considered a standard treatment for complicated FRNS/SDNS. However, few trials have assessed the efficacy and safety of rituximab for childhood-onset FRNS/SDNS without prior treatment of steroid-sparing agents (uncomplicated FRNS/SDNS).

## OBJECTIVES

To evaluate the long-term outcome (relapse-free survival) of rituximab in a relapsing course of SSNS without prior treatment with steroid-sparing agents in children upon the completion of our current trial.

## METHODS

*Criteria for considering studies for this review:*

Types of studies: We included randomized controlled trials (RCTs) or quasi-RCTs (RCTs in which allocation to treatment was obtained by alternation, use of alternate medical records, date of birth, or other predictable methods) that evaluated SSNS in children and compared rituximab with placebo, or control.

Participants:

Inclusion criteria: Children aged from 3 months to 18 years with relapsing SSNS (i.e., the child became oedema-free, and their urine protein was 1+ on dipstick, or < 4 mg/m$^2$/h or urine protein/creatinine ratio < 0.02 g/mmol) for 3 consecutive days while receiving corticosteroid therapy). Relapse of NS was defined as the recurrence of proteinuria measured semi-quantitatively on urinalysis or quantitatively using albumin or protein/creatinine ratios or timed urine specimens. A kidney biopsy diagnosis of minimal change disease was not required for study inclusion.

Exclusion criteria: We excluded children with steroid-resistant NS, congenital NS, or other renal or systemic forms of NS defined on kidney biopsy, clinical features, or serology (e.g., post-infectious glomerulonephritis, Henoch–Schönlein nephritis, systemic lupus erythematosus).

Interventions:

Rituximab with placebo, or control

Outcome measures:

Numbers of children with and without relapse at 12, 24, and 36 months, or longer.

*Search methods to identify studies:*

Electronic searches:

We searched the following online sources:

1. Monthly searches of the Cochrane Central Register of Controlled Trials (CENTRAL)
2. Weekly searches of MEDLINE
3. Searching of the current year of EMBASE

Searching other resources:

1. Reference lists of review articles, relevant studies, and clinical practice guidelines
2. Contacting relevant individuals/organizations to seek information about unpublished or incomplete studies

*Data collection and analysis:*

The review was performed by three authors (RM, as a methods expert, and KI and TH, as content experts). The search methods described were used to obtain titles and abstracts of studies that could be relevant to the review. Studies reported in non-English-language journals were planned to be translated before assessment; however, all identified articles were published in English. Any further information required from the original author(s) was included in the review. If necessary, disagreements were resolved by consultation.

Data extraction and risk of bias assessment was performed independently using standard data extraction forms by the same authors, who screened the studies for eligibility. Disagreements were resolved by consultation among the authors.

Regarding the quality of the studies, the following items were independently assessed by two authors using the risk of bias assessment tool (Higgins 2011).

• Was there adequate sequence generation (selection bias)?
• Was allocation adequately concealed (selection bias)?
• Was knowledge of the allocated interventions adequately prevented during the study?
* Participants and personnel (performance bias)
* Outcome assessors (detection bias)
• Were incomplete outcome data adequately addressed (attrition bias)?
• Were reports of the study free of the suggestion of selective outcome reporting (reporting bias)?
• Was the study apparently free of other problems that could put it at a risk of bias?

*Measures of treatment effect:*

For dichotomous outcomes (relapse or no relapse), results were expressed as the risk ratio with 95% confidence intervals. $I^2$ statistics were used to measure the level of heterogeneity; however, the judgement on the use of either random or fixed models for the meta-analyses were based on consideration of both clinical and statistical heterogeneity. We decided to employ the fixed-effect model as 1) the analyses only include three trials from two research teams (too few trials)[1] and 2) the selection of the random or fixed effect model is considered as not established yet.[2]

**RESULTS**

A total of 1202 references were identified from the search; 4 studies reported in 12 references were retrieved. After reviewing the 12 references, we further retrieved 8 references from the reference list of the identified references. Out of the 20 references (8 studies), we included two studies from the same author team, which were published as two papers, as shown in the PRISMA Chart (Supplementary Fig. S3) (Ravani et al., 2015; Ravani et al., 2020). The list of excluded trials is presented as Supplementary Table S12.

The quality of the identified studies was fair. Both trials were designed to evaluate non-inferiority of the intervention, and data presented in the published papers were considered insufficient to assess the long-term effects of rituximab.

In collaboration with the author team of the above two trials, we obtained data on the long-term outcomes of the participants in the trials. A series of meta-analyses of the three studies (the two previous studies and our present study), were performed using the fixed effect model to evaluate the outcomes 12, 24, and 36 months after the intervention, as shown in Figure 5 (main text). Meta-analyses of the three trials from the two different author teams showed markedly beneficial effects of rituximab in children with uncomplicated FRNS/SDNS during up to 36 months of follow-up. The results using the random effect model were consistent with those of the fixed effect model as shown in Supplementary Fig. S4.

**Implications of all of the available evidence**

Our present randomized trial confirmed that rituximab is effective and well-tolerated in childhood-onset uncomplicated FRNS/SDNS, potentially leading to relatively high long-term remission rates.

**REFERENCES**

1 Dettori JR, Norvell DC, Chapman JR. Fixed-Effect vs Random-Effects Models for Meta-Analysis: 3 Points to Consider. Global Spine J. 2022 Sep;12(7):1624-1626. doi: 10.1177/21925682221110527.

2 Charles Poole, Sander Greenland, Random-Effects Meta-Analyses Are Not Always Conservative, American Journal of Epidemiology, Volume 150, Issue 5, 1 September 1999, Pages 469–475.

## Supplementary Tables

***Supplementary Table S1*: Secondary analysis: Time to treatment failure during the blinded phase**

| | No. of patients | No. of patients with treatment failure | No. of patients censored | Median (95%CI) (days) | Hazard ratio (95%CI) |
|---|---|---|---|---|---|
| Rituximab | 18 | 8 | 10 | not reached (175 - not reached) | 0.24 (0.10 - 0.57) |
| Placebo | 22 | 19 | 3 | 84 (70 - 102) | |

95% CI=95% confidence interval.

***Supplementary Table S2*: Time course of peripheral blood B-cell counts (/µl)**

| | | Rituximab | Placebo | Wilcoxon rank sum test |
|---|---|---|---|---|
| Day 1 | Number of patients | 18 | 22 | p =0.45 |
| | Mean | 692 | 604 | |
| | Standard deviation | 505 | 424 | |
| | Minimum | 244 | 59 | |
| | Median | 504 | 462 | |
| | Maximum | 2226 | 1331 | |
| Day 29 | Number of patients | 18 | 22 | p <0.001 |
| | Mean | 4 | 357 | |
| | Standard deviation | 2 | 260 | |
| | Minimum | 1 | 115 | |
| | Median | 3 | 281 | |
| | Maximum | 9 | 1149 | |
| Day 85 | Number of patients | 17 | 8 | p <0.001 |
| | Mean | 8 | 445 | |
| | Standard deviation | 12 | 228 | |
| | Minimum | 1 | 122 | |
| | Median | 4 | 398 | |
| | Maximum | 46 | 857 | |
| Day 141 | Number of patients | 17 | 3 | p =0.015 |
| | Mean | 160 | 615 | |
| | Standard deviation | 175 | 154 | |
| | Minimum | 1 | 491 | |
| | Median | 107 | 567 | |
| | Maximum | 609 | 788 | |
| Day 197 | Number of patients | 17 | 3 | p =0.07 |
| | Mean | 256 | 577 | |
| | Standard deviation | 196 | 394 | |
| | Minimum | 44 | 278 | |
| | Median | 211 | 430 | |
| | Maximum | 747 | 1023 | |
| Day 253 | Number of patients | 17 | 3 | p =0.06 |
| | Mean | 392 | 691 | |
| | Standard deviation | 286 | 246 | |
| | Minimum | 73 | 468 | |

| | | | | |
|---|---|---|---|---|
| | Median | 355 | 651 | |
| | Maximum | 1154 | 954 | |
| Day 309 | Number of patients | 17 | 3 | p =0.34 |
| | Mean | 422 | 555 | |
| | Standard deviation | 284 | 250 | |
| | Minimum | 49 | 298 | |
| | Median | 382 | 570 | |
| | Maximum | 1136 | 798 | |
| Day 365 | Number of patients | 17 | 3 | p =0.83 |
| | Mean | 615 | 570 | |
| | Standard deviation | 560 | 322 | |
| | Minimum | 89 | 232 | |
| | Median | 432 | 605 | |
| | Maximum | 2359 | 872 | |

***Supplementary Table S3*: Per-protocol analysis: time to relapse during the blinded phase**

| | **No. of patients** | **No. of patients with relapse** | **No. of patients censored** | **Median (95%CI) (days)** | **Hazard ratio (95%CI)** |
|---|---|---|---|---|---|
| Rituximab | 18 | 10 | 8 | 285 (173 - not reached) | 0.29 (0.13 - 0.65) |
| Placebo | 19 | 17 | 2 | 82 (57 - 100) | |

95% CI=95% confidence interval.

***Supplementary Table S4*: Frequent relapse subgroup: time to relapse during the blinded phase**

| | **No. of patients** | **No. of patients with relapse** | **No. of patients censored** | **Median (95%CI) (days)** | **Hazard ratio (95%CI)** |
|---|---|---|---|---|---|
| Rituximab | 11 | 7 | 4 | 268 (78 - not reached) | 0.27 (0.10 - 0.70) |
| Placebo | 16 | 15 | 1 | 81 (66 - 106) | |

95% CI=95% confidence interval.

***Supplementary Table S5*: Steroid-dependence subgroup: time to relapse during the blinded phase**

| | **No. of patients** | **No. of patients with relapse** | **No. of patients censored** | **Median (95%CI) (days)** | **Hazard ratio (95%CI)** |
|---|---|---|---|---|---|
| Rituximab | 7 | 3 | 4 | not reached (66 - not reached) | 0.25 (0.06 - 1.12) |
| Placebo | 6 | 5 | 1 | 80 (42 - not reached) | |

95% CI=95% confidence interval.

***Supplementary Table S6*: Post-hoc analysis: time to relapse during the blinded phase with nephrotic syndrome type (frequently relapsing/steroid-dependent) and weight at enrollment as covariates**

| | No. of patients | No. of patients with relapse | No. of patients censored | Median (95%CI) (days) | Hazard ratio (95% CI) |
|---|---|---|---|---|---|
| Rituximab | 18 | 10 | 8 | not reached (173 - not reached) | 0.29 (0.12- 0.68) |
| Placebo | 22 | 20 | 2 | 81 (66-100) | |

95% CI=95% confidence interval.

*Supplementary Table S7*: **Numbers and incidences of adverse events during the blinded phase**

| | Rituximab (N=18) | Placebo (N=22) | Fisher's exact test |
|---|---|---|---|
| **No. of patients with adverse events** | 18 | 19 | |
| **No. of adverse events** | 131 | 79 | |
| **Incidence of patients with adverse events (95%CI)(%)** | 100 (82-100) | 87 (66-97) | P=0.24 |
| **No. of patients with infusion reactions** | 11 | 2 | |
| **No. of infusion reactions** | 19 | 2 | |
| **Incidence of patients with infusion reactions (95%CI)(%)** | 61 (36-83) | 9 (1-29) | P<0.001 |

95%CI=95% confidence interval.

## *Supplementary Table S8*: Numbers and incidences of adverse events by severity during the blinded phase

| Group | Rituximab | | | | | | Placebo | | | | | |
|---|---|---|---|---|---|---|---|---|---|---|---|---|
| Number of patients | 18 | | | | | | 22 | | | | | |
| Grade (NCI-CTCAE) | Grade 1 | Grade 2 | Grade 3 | Grade 4 | Grade 5 | Total | Grade 1 | Grade 2 | Grade 3 | Grade 4 | Grade 5 | Total |
| **Number of patients with at least one event** | 2 ( 11) | 14 ( 78) | 2 ( 11.1) | 0 ( 0) | 0 ( 0) | 18 (100) | 2 ( 9) | 15 ( 68) | 2 ( 9) | 0 ( 0) | 0 ( 0) | 19 ( 86) |
| **Infections and infestations** | 1 ( 6) | 10 ( 56) | 0 ( 0) | 0 ( 0) | 0 ( 0) | 11 ( 61) | 1 ( 5) | 8 ( 36) | 0 ( 0) | 0 ( 0) | 0 ( 0) | 9 ( 41) |
| Blister infected | 0 ( 0) | 1 ( 6) | 0 ( 0) | 0 ( 0) | 0 ( 0) | 1 ( 6) | 0 ( 0) | 0 ( 0) | 0 ( 0) | 0 ( 0) | 0 ( 0) | 0 ( 0) |
| Bronchitis | 0 ( 0) | 2 ( 11) | 0 ( 0) | 0 ( 0) | 0 ( 0) | 2 ( 11) | 0 ( 0) | 0 ( 0) | 0 ( 0) | 0 ( 0) | 0 ( 0) | 0 ( 0) |
| Conjunctivitis | 1 ( 6) | 0 ( 0) | 0 ( 0) | 0 ( 0) | 0 ( 0) | 1 ( 6) | 0 ( 0) | 0 ( 0) | 0 ( 0) | 0 ( 0) | 0 ( 0) | 0 ( 0) |
| Gastroenteritis | 0 ( 0) | 0 ( 0) | 0 ( 0) | 0 ( 0) | 0 ( 0) | 0 ( 0) | 0 ( 0) | 1 ( 5) | 0 ( 0) | 0 ( 0) | 0 ( 0) | 1 ( 5) |
| Gastroenteritis viral | 0 ( 0) | 1 ( 6) | 0 ( 0) | 0 ( 0) | 0 ( 0) | 1 ( 6) | 0 ( 0) | 0 ( 0) | 0 ( 0) | 0 ( 0) | 0 ( 0) | 0 ( 0) |
| Gingivitis | 1 ( 6) | 0 ( 0) | 0 ( 0) | 0 ( 0) | 0 ( 0) | 1 ( 6) | 0 ( 0) | 0 ( 0) | 0 ( 0) | 0 ( 0) | 0 ( 0) | 0 ( 0) |
| Hordeolum | 0 ( 0) | 2 ( 11) | 0 ( 0) | 0 ( 0) | 0 ( 0) | 2 ( 11) | 0 ( 0) | 1 ( 5) | 0 ( 0) | 0 ( 0) | 0 ( 0) | 1 ( 5) |
| Impetigo | 0 ( 0) | 1 ( 6) | 0 ( 0) | 0 ( 0) | 0 ( 0) | 1 ( 6) | 0 ( 0) | 0 ( 0) | 0 ( 0) | 0 ( 0) | 0 ( 0) | 0 ( 0) |
| Influenza | 0 ( 0) | 1 ( 6) | 0 ( 0) | 0 ( 0) | 0 ( 0) | 1 ( 6) | 0 ( 0) | 0 ( 0) | 0 ( 0) | 0 ( 0) | 0 ( 0) | 0 ( 0) |
| Molluscum contagiosum | 1 ( 6) | 2 ( 11) | 0 ( 0) | 0 ( 0) | 0 ( 0) | 3 ( 17) | 0 ( 0) | 0 ( 0) | 0 ( 0) | 0 ( 0) | 0 ( 0) | 1 ( 5) |
| Nasopharyngitis | 0 ( 0) | 4 ( 22) | 0 ( 0) | 0 ( 0) | 0 ( 0) | 4 ( 22) | 0 ( 0) | 4 ( 18) | 0 ( 0) | 0 ( 0) | 0 ( 0) | 4 ( 18) |
| Otitis media acute | 0 ( 0) | 0 ( 0) | 0 ( 0) | 0 ( 0) | 0 ( 0) | 0 ( 0) | 0 ( 0) | 1 ( 5) | 0 ( 0) | 0 ( 0) | 0 ( 0) | 1 ( 5) |
| Paronychia | 0 ( 0) | 1 ( 6) | 0 ( 0) | 0 ( 0) | 0 ( 0) | 1 ( 6) | 0 ( 0) | 0 ( 0) | 0 ( 0) | 0 ( 0) | 0 ( 0) | 0 ( 0) |
| Parotitis | 0 ( 0) | 1 ( 6) | 0 ( 0) | 0 ( 0) | 0 ( 0) | 1 ( 6) | 0 ( 0) | 0 ( 0) | 0 ( 0) | 0 ( 0) | 0 ( 0) | 0 ( 0) |
| Periodontitis | 0 ( 0) | 0 ( 0) | 0 ( 0) | 0 ( 0) | 0 ( 0) | 0 ( 0) | 0 ( 0) | 1 ( 5) | 0 ( 0) | 0 ( 0) | 0 ( 0) | 1 ( 5) |
| Pharyngitis | 0 ( 0) | 1 ( 6) | 0 ( 0) | 0 ( 0) | 0 ( 0) | 1 ( 6) | 0 ( 0) | 1 ( 5) | 0 ( 0) | 0 ( 0) | 0 ( 0) | 1 ( 5) |
| Rhinitis | 0 ( 0) | 0 ( 0) | 0 ( 0) | 0 ( 0) | 0 ( 0) | 0 ( 0) | 0 ( 0) | 1 ( 5) | 0 ( 0) | 0 ( 0) | 0 ( 0) | 1 ( 5) |
| Upper respiratory tract infection | 0 ( 0) | 3 ( 17) | 0 ( 0) | 0 ( 0) | 0 ( 0) | 3 ( 17) | 2 ( 9) | 0 ( 0) | 0 ( 0) | 0 ( 0) | 0 ( 0) | 2 ( 9) |
| Viral rash | 1 ( 6) | 0 ( 0) | 0 ( 0) | 0 ( 0) | 0 ( 0) | 1 ( 6) | 0 ( 0) | 0 ( 0) | 0 ( 0) | 0 ( 0) | 0 ( 0) | 0 ( 0) |
| Tinea infection | 0 ( 0) | 1 ( 6) | 0 ( 0) | 0 ( 0) | 0 ( 0) | 1 ( 6) | 0 ( 0) | 0 ( 0) | 0 ( 0) | 0 ( 0) | 0 ( 0) | 0 ( 0) |
| Conjunctivitis bacterial | 0 ( 0) | 1 ( 6) | 0 ( 0) | 0 ( 0) | 0 ( 0) | 1 ( 6) | 0 ( 0) | 0 ( 0) | 0 ( 0) | 0 ( 0) | 0 ( 0) | 0 ( 0) |
| **Immune system disorders** | 0 ( 0) | 1 ( 6) | 0 ( 0) | 0 ( 0) | 0 ( 0) | 1 ( 6) | 0 ( 0) | 1 ( 5) | 0 ( 0) | 0 ( 0) | 0 ( 0) | 1 ( 5) |
| Food allergy | 0 ( 0) | 0 ( 0) | 0 ( 0) | 0 ( 0) | 0 ( 0) | 0 ( 0) | 0 ( 0) | 1 ( 5) | 0 ( 0) | 0 ( 0) | 0 ( 0) | 1 ( 5) |
| Allergy to arthropod sting | 0 ( 0) | 1 ( 6) | 0 ( 0) | 0 ( 0) | 0 ( 0) | 1 ( 6) | 0 ( 0) | 0 ( 0) | 0 ( 0) | 0 ( 0) | 0 ( 0) | 0 ( 0) |
| **Endocrine disorders** | 1 ( 6) | 0 ( 0) | 0 ( 0) | 0 ( 0) | 0 ( 0) | 1 ( 6) | 0 ( 0) | 0 ( 0) | 0 ( 0) | 0 ( 0) | 0 ( 0) | 0 ( 0) |
| Cushingoid | 1 ( 6) | 0 ( 0) | 0 ( 0) | 0 ( 0) | 0 ( 0) | 1 ( 6) | 0 ( 0) | 0 ( 0) | 0 ( 0) | 0 ( 0) | 0 ( 0) | 0 ( 0) |
| **Metabolism and nutrition disorders** | 1 ( 6) | 0 ( 0) | 0 ( 0) | 0 ( 0) | 0 ( 0) | 1 ( 6) | 1 ( 5) | 0 ( 0) | 1 ( 5) | 0 ( 0) | 0 ( 0) | 2 ( 9) |
| Electrolyte imbalance | 0 ( 0) | 0 ( 0) | 0 ( 0) | 0 ( 0) | 0 ( 0) | 0 ( 0) | 1 ( 5) | 0 ( 0) | 0 ( 0) | 0 ( 0) | 0 ( 0) | 1 ( 5) |
| Hypoalbminemia | 0 ( 0) | 0 ( 0) | 0 ( 0) | 0 ( 0) | 0 ( 0) | 0 ( 0) | 0 ( 0) | 0 ( 0) | 1 ( 5) | 0 ( 0) | 0 ( 0) | 1 ( 5) |
| Central obesity | 1 ( 6) | 0 ( 0) | 0 ( 0) | 0 ( 0) | 0 ( 0) | 1 ( 6) | 0 ( 0) | 0 ( 0) | 0 ( 0) | 0 ( 0) | 0 ( 0) | 0 ( 0) |
| **Nervous system disorders** | 0 ( 0) | 1 ( 6) | 0 ( 0) | 0 ( 0) | 0 ( 0) | 1 ( 6) | 1 ( 5) | 0 ( 0) | 0 ( 0) | 0 ( 0) | 0 ( 0) | 1 ( 5) |
| Headache | 0 ( 0) | 1 ( 6) | 0 ( 0) | 0 ( 0) | 0 ( 0) | 1 ( 6) | 1 ( 5) | 0 ( 0) | 0 ( 0) | 0 ( 0) | 0 ( 0) | 1 ( 5) |
| **Eye disorders** | 0 ( 0) | 2 ( 11) | 0 ( 0) | 0 ( 0) | 0 ( 0) | 2 ( 11) | 0 ( 0) | 0 ( 0) | 0 ( 0) | 0 ( 0) | 0 ( 0) | 0 ( 0) |
| Blepharitis | 0 ( 0) | 1 ( 6) | 0 ( 0) | 0 ( 0) | 0 ( 0) | 1 ( 6) | 0 ( 0) | 0 ( 0) | 0 ( 0) | 0 ( 0) | 0 ( 0) | 0 ( 0) |
| Conjunctivitis allergic | 0 ( 0) | 1 ( 6) | 0 ( 0) | 0 ( 0) | 0 ( 0) | 1 ( 6) | 0 ( 0) | 0 ( 0) | 0 ( 0) | 0 ( 0) | 0 ( 0) | 0 ( 0) |
| **Ear and labyrinth disorders** | 1 ( 6) | 0 ( 0) | 0 ( 0) | 0 ( 0) | 0 ( 0) | 1 ( 6) | 0 ( 0) | 0 ( 0) | 0 ( 0) | 0 ( 0) | 0 ( 0) | 0 ( 0) |
| Ear pain | 1 ( 6) | 0 ( 0) | 0 ( 0) | 0 ( 0) | 0 ( 0) | 1 ( 6) | 0 ( 0) | 0 ( 0) | 0 ( 0) | 0 ( 0) | 0 ( 0) | 0 ( 0) |

| Group | Rituximab | | | | | | Placebo | | | | | |
|---|---|---|---|---|---|---|---|---|---|---|---|---|
| Number of patients | 18 | | | | | | 22 | | | | | |
| Grade (NCI-CTCAE) | Grade 1 | Grade 2 | Grade 3 | Grade 4 | Grade 5 | Total | Grade 1 | Grade 2 | Grade 3 | Grade 4 | Grade 5 | Total |
| **Cardiac disorders** | 1 ( 6) | 0 ( 0) | 0 ( 0) | 0 ( 0) | 0 ( 0) | 1 ( 6) | 0 ( 0) | 0 ( 0) | 0 ( 0) | 0 ( 0) | 0 ( 0) | 0 ( 0) |
| Sinus tachycardia | 1 ( 6) | 0 ( 0) | 0 ( 0) | 0 ( 0) | 0 ( 0) | 1 ( 6) | 0 ( 0) | 0 ( 0) | 0 ( 0) | 0 ( 0) | 0 ( 0) | 0 ( 0) |
| **Vascular disorders** | 0 ( 0) | 0 ( 0) | 0 ( 0) | 0 ( 0) | 0 ( 0) | 0 ( 0) | 0 ( 0) | 1 ( 5) | 2 ( 9) | 0 ( 0) | 0 ( 0) | 3 ( 14) |
| Hypertension | 0 ( 0) | 0 ( 0) | 0 ( 0) | 0 ( 0) | 0 ( 0) | 0 ( 0) | 0 ( 0) | 1 ( 5) | 2 ( 9) | 0 ( 0) | 0 ( 0) | 3 ( 14) |
| **Respiratory, thoracic and mediastinal disorders** | 4 ( 22) | 10 ( 56) | 0 ( 0) | 0 ( 0) | 0 ( 0) | 14 ( 78) | 1 ( 5) | 7 ( 32) | 0 ( 0) | 0 ( 0) | 0 ( 0) | 8 ( 36) |
| Athma | 0 ( 0) | 1 ( 6) | 0 ( 0) | 0 ( 0) | 0 ( 0) | 1 ( 6) | 0 ( 0) | 1 ( 5) | 0 ( 0) | 0 ( 0) | 0 ( 0) | 1 ( 5) |
| Cough | 3 ( 17) | 0 ( 0) | 0 ( 0) | 0 ( 0) | 0 ( 0) | 3 ( 17) | 0 ( 0) | 0 ( 0) | 0 ( 0) | 0 ( 0) | 0 ( 0) | 0 ( 0) |
| Dyspnoea | 1 ( 6) | 1 ( 6) | 0 ( 0) | 0 ( 0) | 0 ( 0) | 2 ( 11) | 0 ( 0) | 0 ( 0) | 0 ( 0) | 0 ( 0) | 0 ( 0) | 0 ( 0) |
| Hypoxia | 0 ( 0) | 1 ( 6) | 0 ( 0) | 0 ( 0) | 0 ( 0) | 1 ( 6) | 0 ( 0) | 0 ( 0) | 0 ( 0) | 0 ( 0) | 0 ( 0) | 0 ( 0) |
| Rhinitis allergic | 0 ( 0) | 1 ( 6) | 0 ( 0) | 0 ( 0) | 0 ( 0) | 1 ( 6) | 0 ( 0) | 2 ( 9) | 0 ( 0) | 0 ( 0) | 0 ( 0) | 2 ( 9) |
| Rhinorrhoea | 0 ( 0) | 2 ( 11) | 0 ( 0) | 0 ( 0) | 0 ( 0) | 2 ( 11) | 0 ( 0) | 1 ( 5) | 0 ( 0) | 0 ( 0) | 0 ( 0) | 2 ( 9) |
| Wheezing | 0 ( 0) | 3 ( 17) | 0 ( 0) | 0 ( 0) | 0 ( 0) | 3 ( 17) | 0 ( 0) | 0 ( 0) | 0 ( 0) | 0 ( 0) | 0 ( 0) | 0 ( 0) |
| Upper respiratory tract inflammation | 1 ( 6) | 8 ( 44) | 0 ( 0) | 0 ( 0) | 0 ( 0) | 9 ( 50) | 0 ( 0) | 3 ( 14) | 0 ( 0) | 0 ( 0) | 0 ( 0) | 3 ( 14) |
| Oropharyngeal discomfort | 3 ( 17) | 0 ( 0) | 0 ( 0) | 0 ( 0) | 0 ( 0) | 3 ( 17) | 0 ( 0) | 0 ( 0) | 0 ( 0) | 0 ( 0) | 0 ( 0) | 0 ( 0) |
| Oropharyngeal pain | 0 ( 0) | 0 ( 0) | 0 ( 0) | 0 ( 0) | 0 ( 0) | 0 ( 0) | 0 ( 0) | 1 ( 5) | 0 ( 0) | 0 ( 0) | 0 ( 0) | 1 ( 5) |
| Pharyngeal paraesthesia | 1 ( 6) | 0 ( 0) | 0 ( 0) | 0 ( 0) | 0 ( 0) | 1 ( 6) | 0 ( 0) | 0 ( 0) | 0 ( 0) | 0 ( 0) | 0 ( 0) | 0 ( 0) |
| **Gastrointestinal disorders** | 4 ( 22) | 3 ( 17) | 0 ( 0) | 0 ( 0) | 0 ( 0) | 7 ( 39) | 1 ( 5) | 2 ( 9) | 0 ( 0) | 0 ( 0) | 0 ( 0) | 3 ( 14) |
| Abdominal pain | 1 ( 6) | 0 ( 0) | 0 ( 0) | 0 ( 0) | 0 ( 0) | 1 ( 6) | 0 ( 0) | 0 ( 0) | 0 ( 0) | 0 ( 0) | 0 ( 0) | 0 ( 0) |
| Diarrhoea | 1 ( 6) | 2 ( 11) | 0 ( 0) | 0 ( 0) | 0 ( 0) | 3 ( 17) | 0 ( 0) | 0 ( 0) | 0 ( 0) | 0 ( 0) | 0 ( 0) | 0 ( 0) |
| Dry mouth | 1 ( 6) | 0 ( 0) | 0 ( 0) | 0 ( 0) | 0 ( 0) | 1 ( 6) | 0 ( 0) | 0 ( 0) | 0 ( 0) | 0 ( 0) | 0 ( 0) | 0 ( 0) |
| Stomatitis | 1 ( 6) | 1 ( 6) | 0 ( 0) | 0 ( 0) | 0 ( 0) | 2 ( 11) | 0 ( 0) | 1 ( 5) | 0 ( 0) | 0 ( 0) | 0 ( 0) | 1 ( 5) |
| Toothache | 0 ( 0) | 1 ( 6) | 0 ( 0) | 0 ( 0) | 0 ( 0) | 1 ( 6) | 0 ( 0) | 0 ( 0) | 0 ( 0) | 0 ( 0) | 0 ( 0) | 0 ( 0) |
| Vomiting | 1 ( 6) | 0 ( 0) | 0 ( 0) | 0 ( 0) | 0 ( 0) | 1 ( 6) | 1 ( 5) | 1 ( 5) | 0 ( 0) | 0 ( 0) | 0 ( 0) | 2 ( 9) |
| **Skin and subcutaneous tissue disorders** | 3 ( 17) | 9 ( 50) | 0 ( 0) | 0 ( 0) | 0 ( 0) | 12 ( 67) | 1 ( 5) | 5 ( 23) | 0 ( 0) | 0 ( 0) | 0 ( 0) | 6 ( 27) |
| Dermatitis | 0 ( 0) | 2 ( 11) | 0 ( 0) | 0 ( 0) | 0 ( 0) | 2 ( 11) | 0 ( 0) | 0 ( 0) | 0 ( 0) | 0 ( 0) | 0 ( 0) | 0 ( 0) |
| Dermatitis acneiform | 1 ( 6) | 0 ( 0) | 0 ( 0) | 0 ( 0) | 0 ( 0) | 1 ( 6) | 0 ( 0) | 0 ( 0) | 0 ( 0) | 0 ( 0) | 0 ( 0) | 0 ( 0) |
| Dermatitis atopic | 0 ( 0) | 1 ( 6) | 0 ( 0) | 0 ( 0) | 0 ( 0) | 1 ( 6) | 0 ( 0) | 0 ( 0) | 0 ( 0) | 0 ( 0) | 0 ( 0) | 0 ( 0) |
| Dry skin | 2 ( 11) | 0 ( 0) | 0 ( 0) | 0 ( 0) | 0 ( 0) | 2 ( 11) | 0 ( 0) | 0 ( 0) | 0 ( 0) | 0 ( 0) | 0 ( 0) | 0 ( 0) |
| Eczema | 0 ( 0) | 2 ( 11) | 0 ( 0) | 0 ( 0) | 0 ( 0) | 2 ( 11) | 0 ( 0) | 0 ( 0) | 0 ( 0) | 0 ( 0) | 0 ( 0) | 0 ( 0) |
| Eczema asteatotic | 0 ( 0) | 2 ( 11) | 0 ( 0) | 0 ( 0) | 0 ( 0) | 2 ( 11) | 0 ( 0) | 1 ( 5) | 0 ( 0) | 0 ( 0) | 0 ( 0) | 1 ( 5) |
| Miliaria | 0 ( 0) | 3 ( 17) | 0 ( 0) | 0 ( 0) | 0 ( 0) | 3 ( 17) | 0 ( 0) | 1 ( 5) | 0 ( 0) | 0 ( 0) | 0 ( 0) | 1 ( 5) |
| Perioral dermatitis | 0 ( 0) | 0 ( 0) | 0 ( 0) | 0 ( 0) | 0 ( 0) | 0 ( 0) | 0 ( 0) | 1 ( 5) | 0 ( 0) | 0 ( 0) | 0 ( 0) | 1 ( 5) |
| Rash | 0 ( 0) | 1 ( 6) | 0 ( 0) | 0 ( 0) | 0 ( 0) | 1 ( 6) | 0 ( 0) | 0 ( 0) | 0 ( 0) | 0 ( 0) | 0 ( 0) | 0 ( 0) |
| Rash maculo-papular | 0 ( 0) | 0 ( 0) | 0 ( 0) | 0 ( 0) | 0 ( 0) | 0 ( 0) | 1 ( 5) | 0 ( 0) | 0 ( 0) | 0 ( 0) | 0 ( 0) | 1 ( 5) |
| Urticaria | 2 ( 11) | 0 ( 0) | 0 ( 0) | 0 ( 0) | 0 ( 0) | 2 ( 11) | 0 ( 0) | 2 ( 9) | 0 ( 0) | 0 ( 0) | 0 ( 0) | 2 ( 9) |
| Onychomadesis | 1 ( 6) | 0 ( 0) | 0 ( 0) | 0 ( 0) | 0 ( 0) | 1 ( 6) | 0 ( 0) | 0 ( 0) | 0 ( 0) | 0 ( 0) | 0 ( 0) | 0 ( 0) |
| Urticaria chronic | 0 ( 0) | 1 ( 6) | 0 ( 0) | 0 ( 0) | 0 ( 0) | 1 ( 6) | 0 ( 0) | 0 ( 0) | 0 ( 0) | 0 ( 0) | 0 ( 0) | 0 ( 0) |
| Leukoplakia | 1 ( 6) | 0 ( 0) | 0 ( 0) | 0 ( 0) | 0 ( 0) | 1 ( 6) | 0 ( 0) | 0 ( 0) | 0 ( 0) | 0 ( 0) | 0 ( 0) | 0 ( 0) |
| **Musculoskeletal and connective tissue disorders** | 0 ( 0) | 0 ( 0) | 0 ( 0) | 0 ( 0) | 0 ( 0) | 0 ( 0) | 1 ( 5) | 0 ( 0) | 0 ( 0) | 0 ( 0) | 0 ( 0) | 1 ( 5) |
| Arthralgia | 0 ( 0) | 0 ( 0) | 0 ( 0) | 0 ( 0) | 0 ( 0) | 0 ( 0) | 1 ( 5) | 0 ( 0) | 0 ( 0) | 0 ( 0) | 0 ( 0) | 1 ( 5) |
| **Renal and urinary disorders** | 0 ( 0) | 0 ( 0) | 0 ( 0) | 0 ( 0) | 0 ( 0) | 0 ( 0) | 1 ( 5) | 0 ( 0) | 0 ( 0) | 0 ( 0) | 0 ( 0) | 1 ( 5) |
| Acute kidney injury | 0 ( 0) | 0 ( 0) | 0 ( 0) | 0 ( 0) | 0 ( 0) | 0 ( 0) | 1 ( 5) | 0 ( 0) | 0 ( 0) | 0 ( 0) | 0 ( 0) | 1 ( 5) |

| Group | Rituximab | | | | | | Placebo | | | | | |
|---|---|---|---|---|---|---|---|---|---|---|---|---|
| Number of patients | 18 | | | | | | 22 | | | | | |
| Grade (NCI-CTCAE) | Grade 1 | Grade 2 | Grade 3 | Grade 4 | Grade 5 | Total | Grade 1 | Grade 2 | Grade 3 | Grade 4 | Grade 5 | Total |
| **General disorders and administration site conditions** | 2 ( 11) | 0 ( 0) | 0 ( 0) | 0 ( 0) | 0 ( 0) | 2 ( 11) | 1 ( 5) | 2 ( 9) | 0 ( 0) | 0 ( 0) | 0 ( 0) | 3 ( 14) |
| Fever | 2 ( 11) | 0 ( 0) | 0 ( 0) | 0 ( 0) | 0 ( 0) | 2 ( 11) | 1 ( 5) | 2 ( 9) | 0 ( 0) | 0 ( 0) | 0 ( 0) | 3 ( 14) |
| **Investigations** | 2 ( 11) | 0 ( 0) | 2 ( 11) | 0 ( 0) | 0 ( 0) | 4 ( 22) | 5 ( 23) | 1 ( 5) | 0 ( 0) | 0 ( 0) | 0 ( 0) | 6 ( 27) |
| Alanine aminotransferase increased | 0 ( 0) | 0 ( 0) | 0 ( 0) | 0 ( 0) | 0 ( 0) | 0 ( 0) | 3 ( 14) | 0 ( 0) | 0 ( 0) | 0 ( 0) | 0 ( 0) | 3 ( 14) |
| Blood bilirubin increased | 1 ( 6) | 0 ( 0) | 0 ( 0) | 0 ( 0) | 0 ( 0) | 1 ( 6) | 0 ( 0) | 0 ( 0) | 0 ( 0) | 0 ( 0) | 0 ( 0) | 0 ( 0) |
| Blood creatinine increased | 0 ( 0) | 0 ( 0) | 0 ( 0) | 0 ( 0) | 0 ( 0) | 0 ( 0) | 1 ( 5) | 0 ( 0) | 0 ( 0) | 0 ( 0) | 0 ( 0) | 1 ( 5) |
| Blood pressure increased | 0 ( 0) | 0 ( 0) | 0 ( 0) | 0 ( 0) | 0 ( 0) | 0 ( 0) | 1 ( 5) | 0 ( 0) | 0 ( 0) | 0 ( 0) | 0 ( 0) | 1 ( 5) |
| Eosinophil count increased | 1 ( 6) | 0 ( 0) | 0 ( 0) | 0 ( 0) | 0 ( 0) | 1 ( 6) | 1 ( 5) | 0 ( 0) | 0 ( 0) | 0 ( 0) | 0 ( 0) | 1 ( 5) |
| gamma-glutamyltransferase increased | 0 ( 0) | 0 ( 0) | 0 ( 0) | 0 ( 0) | 0 ( 0) | 0 ( 0) | 1 ( 5) | 0 ( 0) | 0 ( 0) | 0 ( 0) | 0 ( 0) | 1 ( 5) |
| Intraocular pressure increased | 0 ( 0) | 0 ( 0) | 0 ( 0) | 0 ( 0) | 0 ( 0) | 0 ( 0) | 0 ( 0) | 1 ( 5) | 0 ( 0) | 0 ( 0) | 0 ( 0) | 1 ( 5) |
| Lymphocyte count  decreased | 0 ( 0) | 0 ( 0) | 2 ( 11) | 0 ( 0) | 0 ( 0) | 2 ( 11) | 0 ( 0) | 0 ( 0) | 0 ( 0) | 0 ( 0) | 0 ( 0) | 0 ( 0) |
| Neutrophil count decreased | 0 ( 0) | 0 ( 0) | 1 ( 6) | 0 ( 0) | 0 ( 0) | 1 ( 6) | 0 ( 0) | 0 ( 0) | 0 ( 0) | 0 ( 0) | 0 ( 0) | 0 ( 0) |
| Neutrophil count increased | 1 ( 6) | 0 ( 0) | 0 ( 0) | 0 ( 0) | 0 ( 0) | 1 ( 6) | 1 ( 5) | 0 ( 0) | 0 ( 0) | 0 ( 0) | 0 ( 0) | 1 ( 5) |
| White blood cell count increased | 1 ( 6) | 0 ( 0) | 0 ( 0) | 0 ( 0) | 0 ( 0) | 1 ( 6) | 1 ( 5) | 0 ( 0) | 0 ( 0) | 0 ( 0) | 0 ( 0) | 1 ( 5) |
| Blood phosphorus decreased | 0 ( 0) | 0 ( 0) | 0 ( 0) | 0 ( 0) | 0 ( 0) | 0 ( 0) | 1 ( 5) | 0 ( 0) | 0 ( 0) | 0 ( 0) | 0 ( 0) | 1 ( 5) |
| **Injury, poisoning and procedural complications** | 1 ( 6) | 7 ( 39) | 0 ( 0) | 0 ( 0) | 0 ( 0) | 8 ( 44) | 0 ( 0) | 4 ( 18) | 0 ( 0) | 0 ( 0) | 0 ( 0) | 4 ( 18) |
| Arthropod sting | 2 ( 11) | 2 ( 11) | 0 ( 0) | 0 ( 0) | 0 ( 0) | 4 ( 22) | 0 ( 0) | 2 ( 9) | 0 ( 0) | 0 ( 0) | 0 ( 0) | 2 ( 9) |
| Fall | 0 ( 0) | 0 ( 0) | 0 ( 0) | 0 ( 0) | 0 ( 0) | 0 ( 0) | 1 ( 5) | 0 ( 0) | 0 ( 0) | 0 ( 0) | 0 ( 0) | 1 ( 5) |
| Foot fracture | 0 ( 0) | 1 ( 6) | 0 ( 0) | 0 ( 0) | 0 ( 0) | 1 ( 6) | 0 ( 0) | 0 ( 0) | 0 ( 0) | 0 ( 0) | 0 ( 0) | 0 ( 0) |
| Ligament sprain | 0 ( 0) | 1 ( 6) | 0 ( 0) | 0 ( 0) | 0 ( 0) | 1 ( 6) | 0 ( 0) | 0 ( 0) | 0 ( 0) | 0 ( 0) | 0 ( 0) | 0 ( 0) |
| Ulna fracture | 0 ( 0) | 0 ( 0) | 0 ( 0) | 0 ( 0) | 0 ( 0) | 0 ( 0) | 0 ( 0) | 1 ( 5) | 0 ( 0) | 0 ( 0) | 0 ( 0) | 1 ( 5) |
| Contusion | 0 ( 0) | 2 ( 11) | 0 ( 0) | 0 ( 0) | 0 ( 0) | 2 ( 11) | 0 ( 0) | 0 ( 0) | 0 ( 0) | 0 ( 0) | 0 ( 0) | 0 ( 0) |
| Wound | 0 ( 0) | 1 ( 6) | 0 ( 0) | 0 ( 0) | 0 ( 0) | 1 ( 6) | 0 ( 0) | 1 ( 5) | 0 ( 0) | 0 ( 0) | 0 ( 0) | 1 ( 5) |
| Ear injury | 0 ( 0) | 0 ( 0) | 0 ( 0) | 0 ( 0) | 0 ( 0) | 0 ( 0) | 0 ( 0) | 1 ( 5) | 0 ( 0) | 0 ( 0) | 0 ( 0) | 1 ( 5) |
| Skin laceration | 0 ( 0) | 0 ( 0) | 0 ( 0) | 0 ( 0) | 0 ( 0) | 0 ( 0) | 0 ( 0) | 1 ( 5) | 0 ( 0) | 0 ( 0) | 0 ( 0) | 1 ( 5) |
| Skin abrasion | 0 ( 0) | 2 ( 11) | 0 ( 0) | 0 ( 0) | 0 ( 0) | 2 ( 11) | 0 ( 0) | 0 ( 0) | 0 ( 0) | 0 ( 0) | 0 ( 0) | 0 ( 0) |

Numbers in parentheses indicate %.

NCI-CTCAE=National Cancer Institute-Common Terminology Criteria for Adverse Events.

### *Supplementary Table S9*: Numbers and incidences of infusion reactions by severity during the blinded phase

| Group | Rituximab | | | | | | Placebo | | | | | |
|---|---|---|---|---|---|---|---|---|---|---|---|---|
| No. of patients | 18 | | | | | | 22 | | | | | |
| Grade (NCI-CTCAE) | Grade1 | Grade2 | Grade3 | Grade4 | Grade5 | 合計 | Grade1 | Grade2 | Grade3 | Grade4 | Grade5 | 合計 |
| **Number of patients with at least one event** | 6 ( 33) | 5 ( 28) | 0 ( 0) | 0 ( 0) | 0 ( 0) | 11 ( 61) | 2 ( 9.1) | 0 ( 0) | 0 ( 0) | 0 ( 0) | 0 ( 0) | 2 ( 9) |
| **Cardiac disorders** | 1 ( 6) | 0 ( 0) | 0 ( 0) | 0 ( 0) | 0 ( 0) | 1 ( 6) | 0 ( 0) | 0 ( 0) | 0 ( 0) | 0 ( 0) | 0 ( 0) | 0 ( 0) |
| Sinus tachycardia | 1 ( 6) | 0 ( 0) | 0 ( 0) | 0 ( 0) | 0 ( 0) | 1 ( 6) | 0 ( 0) | 0 ( 0) | 0 ( 0) | 0 ( 0) | 0 ( 0) | 0 ( 0) |
| **Respiratory, thoracic and mediastinal disorders** | 6 ( 33) | 5 ( 28) | 0 ( 0) | 0 ( 0) | 0 ( 0) | 11 ( 61) | 0 ( 0) | 0 ( 0) | 0 ( 0) | 0 ( 0) | 0 ( 0) | 0 ( 0) |
| Cough | 3 ( 17) | 0 ( 0) | 0 ( 0) | 0 ( 0) | 0 ( 0) | 3 ( 17) | 0 ( 0) | 0 ( 0) | 0 ( 0) | 0 ( 0) | 0 ( 0) | 0 ( 0) |
| Dyspnoea | 1 ( 6) | 1 ( 6) | 0 ( 0) | 0 ( 0) | 0 ( 0) | 2 ( 11) | 0 ( 0) | 0 ( 0) | 0 ( 0) | 0 ( 0) | 0 ( 0) | 0 ( 0) |
| Hypoxia | 0 ( 0) | 1 ( 6) | 0 ( 0) | 0 ( 0) | 0 ( 0) | 1 ( 6) | 0 ( 0) | 0 ( 0) | 0 ( 0) | 0 ( 0) | 0 ( 0) | 0 ( 0) |
| Wheezing | 0 ( 0) | 3 ( 17) | 0 ( 0) | 0 ( 0) | 0 ( 0) | 3 ( 17) | 0 ( 0) | 0 ( 0) | 0 ( 0) | 0 ( 0) | 0 ( 0) | 0 ( 0) |
| Oropharyngeal discomfort | 3 ( 17) | 0 ( 0) | 0 ( 0) | 0 ( 0) | 0 ( 0) | 3 ( 17) | 0 ( 0) | 0 ( 0) | 0 ( 0) | 0 ( 0) | 0 ( 0) | 0 ( 0) |
| Pharyngeal paraesthesia | 1 ( 6) | 0 ( 0) | 0 ( 0) | 0 ( 0) | 0 ( 0) | 1 ( 6) | 0 ( 0) | 0 ( 0) | 0 ( 0) | 0 ( 0) | 0 ( 0) | 0 ( 0) |
| **Gastrointestinal disorders** | 1 ( 6) | 0 ( 0) | 0 ( 0) | 0 ( 0) | 0 ( 0) | 1 ( 6) | 0 ( 0) | 0 ( 0) | 0 ( 0) | 0 ( 0) | 0 ( 0) | 0 ( 0) |
| Dry mouth | 1 ( 6) | 0 ( 0) | 0 ( 0) | 0 ( 0) | 0 ( 0) | 1 ( 6) | 0 ( 0) | 0 ( 0) | 0 ( 0) | 0 ( 0) | 0 ( 0) | 0 ( 0) |
| **Skin and subcutaneous tissue disorders** | 2 ( 11) | 0 ( 0) | 0 ( 0) | 0 ( 0) | 0 ( 0) | 2 ( 11) | 0 ( 0) | 0 ( 0) | 0 ( 0) | 0 ( 0) | 0 ( 0) | 0 ( 0) |
| Rash | 1 ( 6) | 0 ( 0) | 0 ( 0) | 0 ( 0) | 0 ( 0) | 1 ( 6) | 0 ( 0) | 0 ( 0) | 0 ( 0) | 0 ( 0) | 0 ( 0) | 0 ( 0) |
| Urticaria | 1 ( 6) | 0 ( 0) | 0 ( 0) | 0 ( 0) | 0 ( 0) | 1 ( 6) | 0 ( 0) | 0 ( 0) | 0 ( 0) | 0 ( 0) | 0 ( 0) | 0 ( 0) |
| **General disorders and administration site conditions** | 1 ( 6) | 0 ( 0) | 0 ( 0) | 0 ( 0) | 0 ( 0) | 1 ( 6) | 1 ( 5) | 0 ( 0) | 0 ( 0) | 0 ( 0) | 0 ( 0) | 1 ( 5) |
| Fever | 1 ( 6) | 0 ( 0) | 0 ( 0) | 0 ( 0) | 0 ( 0) | 1 ( 6) | 1 ( 5) | 0 ( 0) | 0 ( 0) | 0 ( 0) | 0 ( 0) | 1 ( 5) |
| **Investigations** | 0 ( 0) | 0 ( 0) | 0 ( 0) | 0 ( 0) | 0 ( 0) | 0 ( 0) | 1 ( 5) | 0 ( 0) | 0 ( 0) | 0 ( 0) | 0 ( 0) | 1 ( 5) |
| Blood pressure increased | 0 ( 0) | 0 ( 0) | 0 ( 0) | 0 ( 0) | 0 ( 0) | 0 ( 0) | 1 ( 5) | 0 ( 0) | 0 ( 0) | 0 ( 0) | 0 ( 0) | 1 ( 5) |

Numbers in parentheses indicate %.
NCI-CTCAE=National Cancer Institute-Common Terminology Criteria for Adverse Events.

***Supplementary Table S10*: Numbers and incidences of patients with infections during the blinded phase**

| | No. of patients | No. of patients with infections that required treatment | No. of infections that required treatment | Incidence of patients with infections that required treatment (95%CI) (%) | Fisher's exact test |
|---|---|---|---|---|---|
| Rituximab | 18 | 10 | 25 | 56 (31 - 79) | p=0.34 |
| Placebo | 22 | 8 | 11 | 36 (17 - 59) | |

95% CI=95% confidence interval.

***Supplementary Table S11*: B-cell depletion and the rates of infection in the rituximab group**

| Peripheral blood B-cell counts | Rate of infections (times/person-years) | Regression coefficient | | Hazard ratio | Chi-square value | P value |
|---|---|---|---|---|---|---|
| | | Estimate | Standard error | | | |
| Depleted | 2.1 (9/4.3) | 3.3 | 1.1 | 26.2 | 9.0 | 0.003 |
| Not depleted or recovered | 1.2 (16/13.2) | | | | | |

*Supplementary Table S12*: **List of 6 excluded studies**

| Excluded trials | Reasons for exclusion |
| --- | --- |
| QX Su, XJ Qi, YN Shen, ZY Dou, ZH Rong, X Zhao, B Yu, YX Wang, XL Wang. The Efficacy and Safety of Rituximab in the Treatment of Steroid-dependent or Frequently Relapsing Nephrotic Syndrome in Children. HK J Paediatr (New Series) 2022;27:241-248. | The study population was different. |
| Solomon N, Lalayiannis AD. Rituximab is more effective than tacrolimus in steroid-dependent nephrotic syndrome. Arch Dis Child Educ Pract Ed. 2019;104(5):279-280. doi: 10.1136/archdischild-2018-316537 | The control group was given Tacrolimus. |
| Ahn, Yo Han MDa; Kim, Seong Heon MDb; Han, Kyoung Hee MDc; Choi, Hyun Jin MD, PhDd; Cho, Heeyeon MD, PhDe; Lee, Jung Won MD, PhDf; Shin, Jae Il MD, PhDg; Cho, Min Hyun MDh; Lee, Joo Hoon MD, PhDi; Park, Young Seo MD, PhDi; Ha, Il-Soo MD, PhDd; Cheong, Hae Il MD, PhDd; Kim, Su Young MD, PhDb; Lee, Seung Joo MD, PhDf; Kang, Hee Gyung MD, PhDd,∗. Efficacy and safety of rituximab in childhood-onset, difficult-to-treat nephrotic syndrome: A multicenter open-label trial in Korea. Medicine 2018;97(46):e13157. doi: 10.1097/MD.0000000000013157 | Different study population. |
| Basu, B., Preussler, S., Sander, A. et al. Randomized clinical trial to compare efficacy and safety of repeated courses of rituximab to single-course rituximab followed by maintenance mycophenolate-mofetil in children with steroid dependent nephrotic syndrome. BMC Nephrol 2020; 21(1):520. doi: 10.1186/s12882-020-02153-5 | The control group was given Mycophenolate Mofetil |
| Basu B, Sander A, Roy B, et al. Efficacy of Rituximab vs Tacrolimus in Pediatric Corticosteroid-Dependent Nephrotic Syndrome: A Randomized Clinical Trial. JAMA Pediatr. 2018;172(8):757–764. doi:10.1001/jamapediatrics.2018.1323 | The control group was given Tacrolimus. |
| Ravani P, Lugani F, Drovandi S, Caridi G, Angeletti A, Ghiggeri GM. Rituximab vs Low-Dose Mycophenolate Mofetil in Recurrence of Steroid-Dependent Nephrotic Syndrome in Children and Young Adults: A Randomized Clinical Trial. JAMA Pediatr. 2021;175(6):631–632. doi:10.1001/jamapediatrics.2020.6150 | The control group was given Mycophenolate Mofetil |

**Supplementary Figures**

***Supplementary Figure S1*: Kaplan–Meier curves for the secondary outcome.**

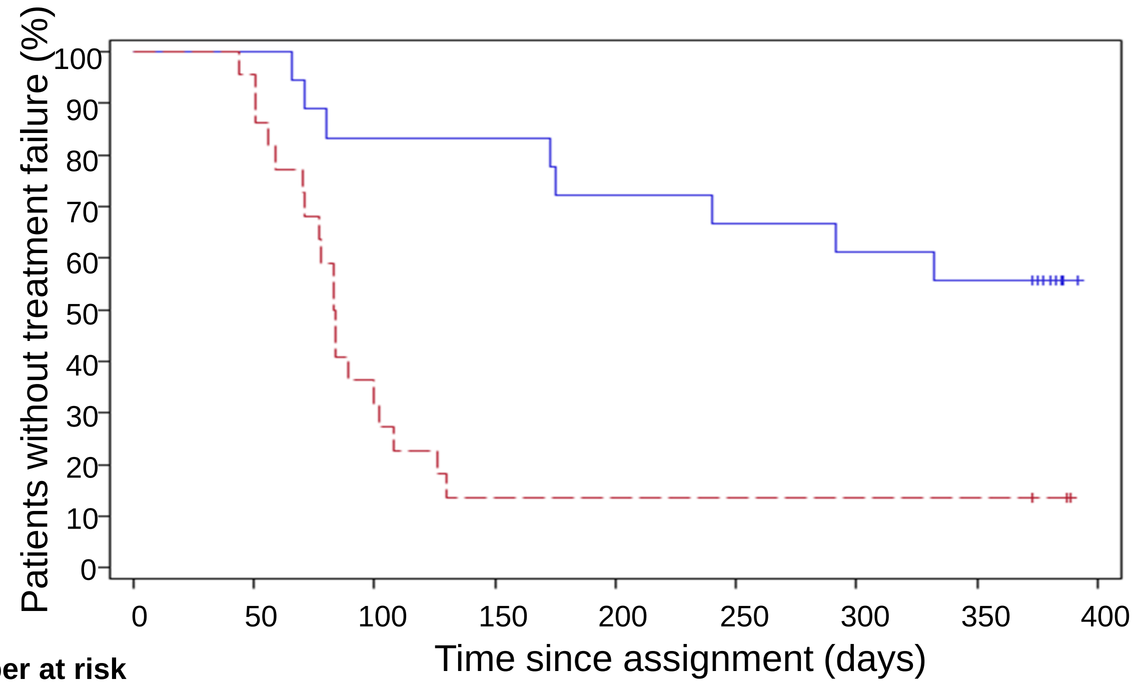


**Number at risk**

| | | | | | | | | |
|---|---|---|---|---|---|---|---|---|
| Rituximab | 18 | 18 | 15 | 15 | 13 | 12 | 11 | 10 |
| Placebo | 22 | 21 | 8 | 3 | 3 | 3 | 3 | 3 |

The time to treatment failure was longer in the rituximab vs. placebo groups (median: not reached vs. 84 days, respectively; HR: 0.24, 95% CI: 0.10–0.57).

***Supplementary Figure S2*: Kaplan–Meier curves for the relapse-free period, including the follow-up period, in patients enrolled in the open-label phase and in all patients treated with rituximab.**

A

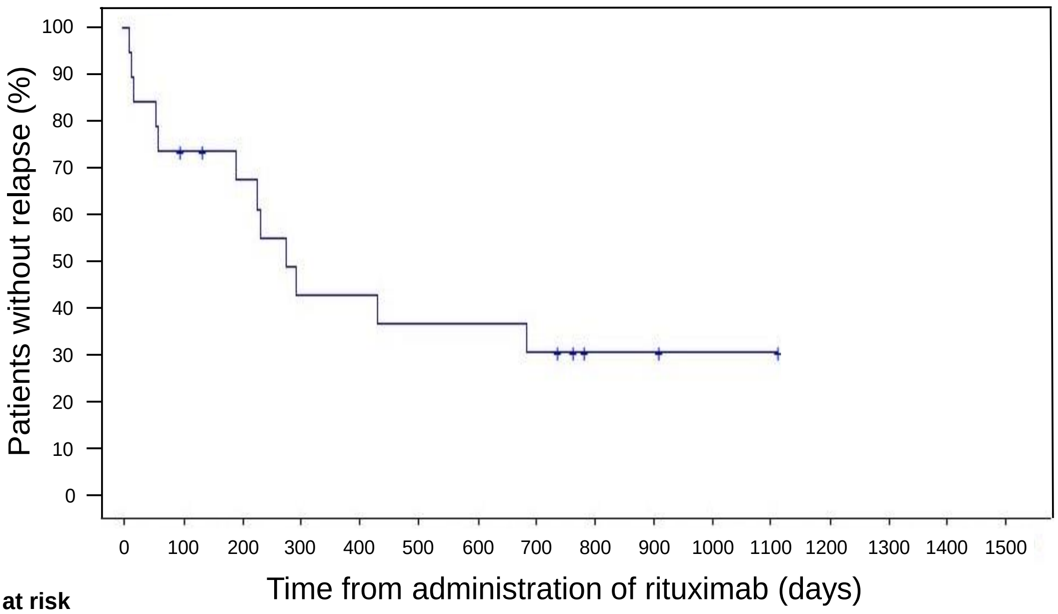


**Number at risk**

19 13 11 7 7 6 6 5 2 2 1 1

B

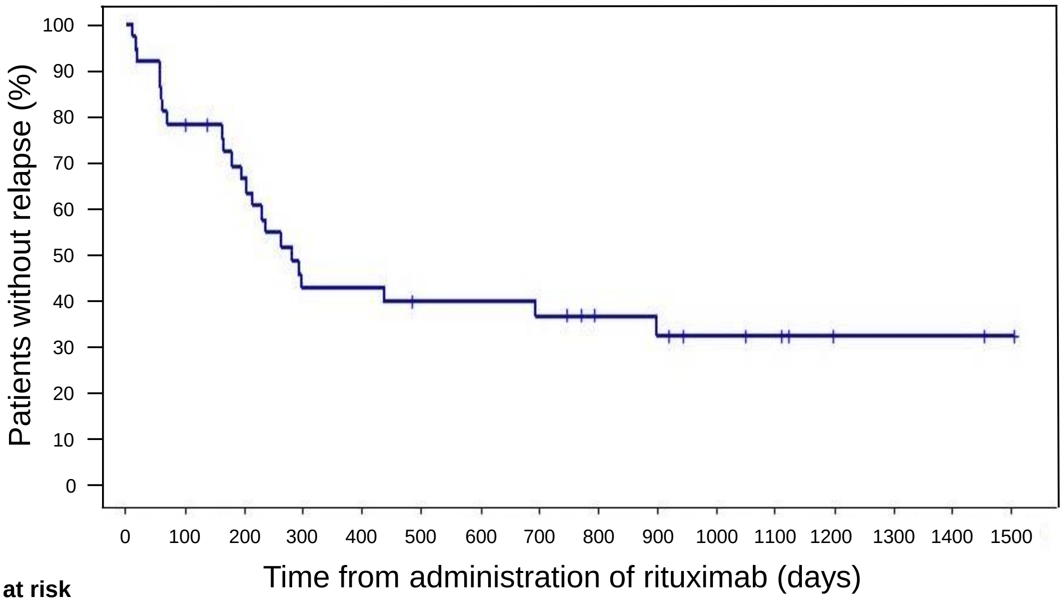


**Number at risk**

37 28 23 15 15 13 13 12 9 8 6 5 2 2 2

(A) The cumulative 2- and 3-year relapse-free survival probability in 19 patients treated with rituximab in the open-label phase were both 31% (95% CI: 11%–53%). (B) In all rituximab-treated patients (18 in the blinded phase plus 19 in the open-label phase), the 2- and 3-year probability was 38% (95% CI: 22%–53%) and 33% (95% CI: 18%–50%), respectively.

*Supplementary Figure S3*: **PRISMA Chart of Mini-Systematic Review**

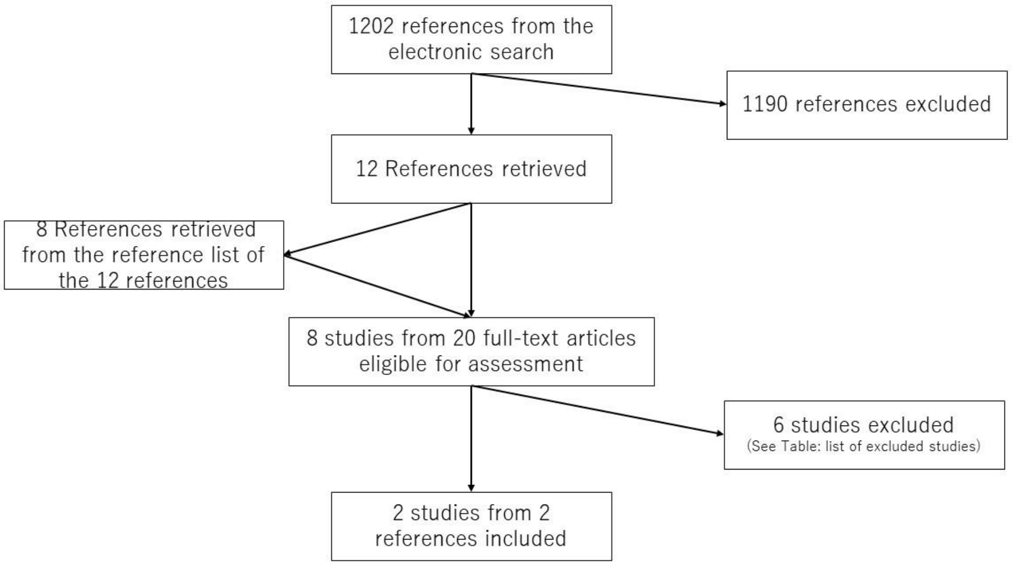

***Supplementary Figure S4*: Meta-analyses of the three trials to evaluate the outcomes at 12, 24, and 36 months after the intervention (Random effect model)**

One year follow-up

| | Rituximab | | Control | | | Risk Ratio | |
|---|---|---|---|---|---|---|---|
| **Study or Subgroup** | **Events** | **Total** | **Events** | **Total** | **Weight** | **M-H, Random, 95% CI** | **Year** |
| Ravani 2015 | 3 | 15 | 15 | 15 | 29.4% | 0.23 [0.09, 0.57] | 2015 |
| Ravani 2020 | 3 | 15 | 15 | 15 | 29.4% | 0.23 [0.09, 0.57] | 2020 |
| Iijima 2024 | 10 | 18 | 20 | 22 | 41.2% | 0.61 [0.40, 0.94] | 2024 |
| **Total (95% CI)** | | **48** | | **52** | **100.0%** | **0.34 [0.15, 0.78]** | |
| Total events | 16 | | 50 | | | | |

Heterogeneity: Tau² = 0.39; Chi² = 7.39, df = 2 (P = 0.02); I² = 73%
Test for overall effect: Z = 2.53 (P = 0.01)

Risk Ratio
M-H, Random, 95% CI

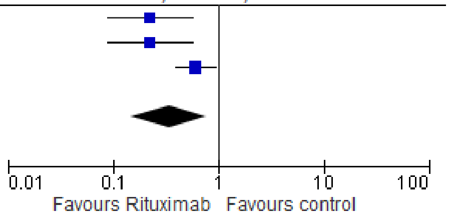


Two year follow-up

| | Rituximab | | Control | | | Risk Ratio | |
|---|---|---|---|---|---|---|---|
| **Study or Subgroup** | **Events** | **Total** | **Events** | **Total** | **Weight** | **M-H, Random, 95% CI** | **Year** |
| Ravani 2015 | 12 | 15 | 15 | 15 | 46.0% | 0.81 [0.61, 1.06] | 2015 |
| Ravani 2020 | 7 | 15 | 15 | 15 | 23.1% | 0.48 [0.29, 0.82] | 2020 |
| Iijima 2024 | 10 | 17 | 20 | 22 | 30.9% | 0.65 [0.43, 0.98] | 2024 |
| **Total (95% CI)** | | **47** | | **52** | **100.0%** | **0.67 [0.49, 0.91]** | |
| Total events | 29 | | 50 | | | | |

Heterogeneity: Tau² = 0.03; Chi² = 3.67, df = 2 (P = 0.16); I² = 46%
Test for overall effect: Z = 2.55 (P = 0.01)

Risk Ratio
M-H, Random, 95% CI

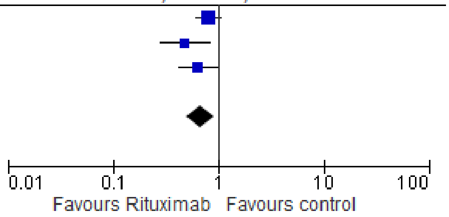


Three year follow-up

| | Rituximab | | Control | | | Risk Ratio | |
|---|---|---|---|---|---|---|---|
| **Study or Subgroup** | **Events** | **Total** | **Events** | **Total** | **Weight** | **M-H, Random, 95% CI** | **Year** |
| Ravani 2015 | 15 | 15 | 15 | 15 | 37.6% | 1.00 [0.88, 1.13] | 2015 |
| Ravani 2020 | 8 | 15 | 15 | 15 | 29.0% | 0.55 [0.34, 0.88] | 2020 |
| Iijima 2024 | 11 | 15 | 20 | 21 | 33.4% | 0.77 [0.56, 1.06] | 2024 |
| **Total (95% CI)** | | **45** | | **51** | **100.0%** | **0.77 [0.46, 1.28]** | |
| Total events | 34 | | 50 | | | | |

Heterogeneity: Tau² = 0.18; Chi² = 18.54, df = 2 (P < 0.0001); I² = 89%
Test for overall effect: Z = 1.01 (P = 0.31)

Risk Ratio
M-H, Random, 95% CI

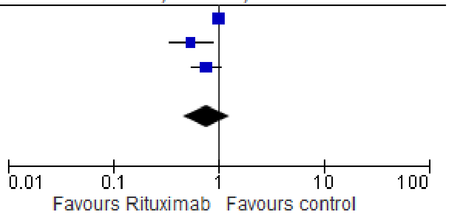

***Supplementary Figure S5*: Kaplan–Meier curves for the relapse-free period, including the follow-up period, in all patients treated with rituximab by the type of nephrotic syndrome**

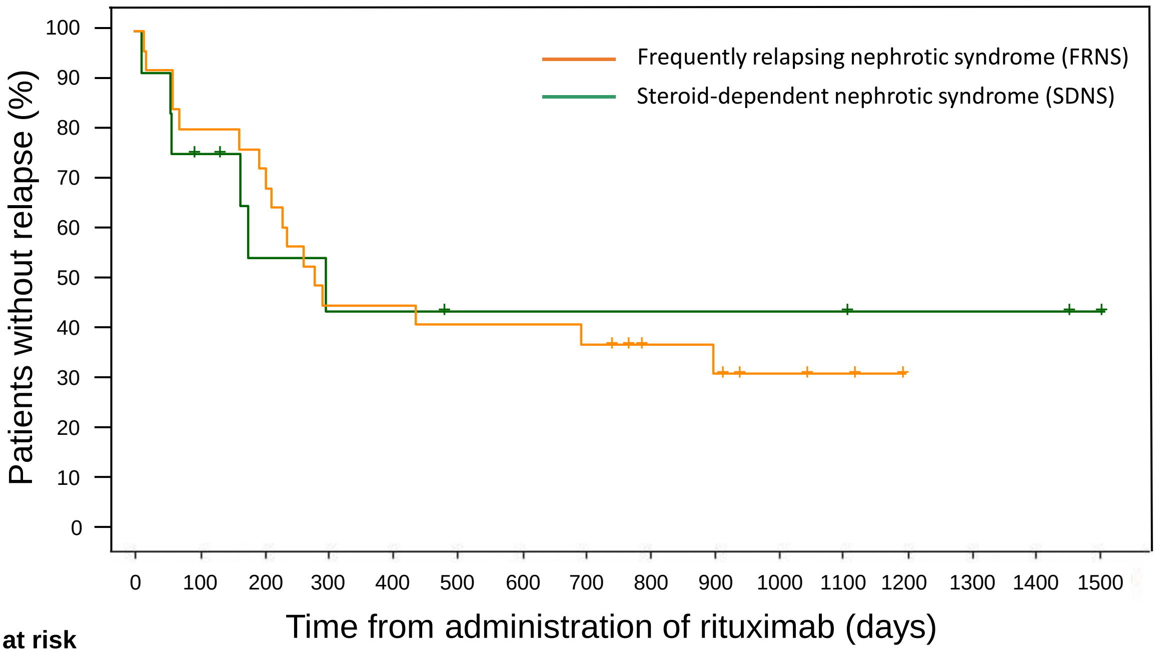


**Number at risk**

| | | | | | | | | | | | | | | | |
|---|---|---|---|---|---|---|---|---|---|---|---|---|---|---|---|
| FRNS | 25 | 20 | 18 | 11 | 11 | 10 | 10 | 9 | 6 | 5 | 3 | 2 | 0 | 0 | 0 |
| SDNS | 12 | 8 | 5 | 4 | 4 | 3 | 3 | 3 | 3 | 3 | 3 | 3 | 2 | 2 | 2 |

In 37 patients who received rituximab during the blinded and open-label phases, the cumulative relapse-free survival probabilities of FRNS vs. SDNS were 36% (95% CI: 18%–54%) vs. 43% (95% CI: 14%–70%) at 2 years after rituximab treatment and 30% (95% CI: 13%–49%) vs. 43% (95% CI: 14%–70%) at 3 years, indicating similar rates in both groups.

# A multicenter, double-blind, placebo-controlled, randomized, parallel-group trial of IDEC-C2B8 in childhood-onset uncomplicated frequently relapsing or steroid-dependent nephrotic syndrome

## Protocol

Protocol number: JSKDC10

Version number: 3.4

Date of creation: 14 January 2022

**Clinical Trial Steering Committee**

Kazumoto Iijima, Department of Pediatrics, Kobe University Hospital

Kandai Nozu, Department of Pediatrics, Kobe University Hospital

Mayumi Sako, Center for Clinical Research, National Center for Child Health and Development

Management of confidential information

The study-specific protocol, information sheet/informed consent form, case report forms, and other documents (hereinafter referred to as study-related information) are confidential and may only be provided to persons directly involved in the study (heads of the study site, the clinical trial coordinating center, the clinical trial steering committee, investigators, clinical trial collaborators, the investigational drug administrator, institutional review boards, and the independent data and safety monitoring committee). Study-related information may not be disclosed to a third party or used for purposes other than the study unless written consent has been obtained from the chairperson of the clinical trial steering committee, except when the details of the study are explained to a subject.

Table of Contents


1 Overview of the study ... 5
1.1 Diagram of the study ... 5
1.2 Objectives of the study ... 8
1.3 Study population ... 9
1.4 Target sample size and planned study period ... 9
1.5 Study schedule ... 10
1.6 Abbreviations ... 12
1.7 Abbreviations (test parameters) ... 13
1.8 Definitions ... 14
2 Introduction ... 15
2.1 Background information ... 15
2.2 Drug information ... 18
3 Objectives ... 28
3.1 Efficacy endpoints ... 28
3.2 Other endpoints ... 29
3.3 Safety endpoints ... 29
4 Study design ... 29
4.1 Type of study ... 29
4.2 Diagram of the study ... 29
4.3 Target sample size ... 31
4.4 Planned study period ... 31
4.5 Discussion of study design ... 31
5 Subject inclusion and exclusion criteria ... 34
5.1 Inclusion criteria ... 34
5.2 Exclusion criteria ... 35
6 Investigational product ... 37
6.1 Overview of the test drug (IDEC-C2B8, active) ... 37
6.2 Overview of the control (IDEC-C2B8 placebo) ... 37
6.3 Packaging and labeling of the investigational product ... 37
6.4 Handling of the investigational product ... 38
7 Investigational plan ... 39
7.1 Definition of study period ... 39
7.2 Screening phase (from informed consent to enrollment) ... 40
7.3 Observation phase ... 43
8 Blinding and key opening ... 46
8.1 Blinding ... 46
8.2 Key opening for the entire study ... 47

8.3 Emergency allocation code opening ... 47
9 Treatment plan ... 48
9.1 Treatment with prednisolone for the last relapse before enrollment ... 48
9.2 Study drug administration (blinded period) ... 50
9.3 Change of dose or date of study drug administration (blinded period) ... 54
9.4 Treatment with prednisolone for relapse during the blinded observation period ... 54
9.5 Early key opening after determination of treatment failure (1) ... 55
9.6 Confirmation of treatment criteria for IDEC-C2B8 (open-label period) treatment ... 55
9.7 IDEC-C2B8 (open-label period) treatment ... 58
9.8 Change of dose or date of IDEC-C2B8 administration (open-label period) ... 59
9.9 Relapse during the open-label observation period ... 59
9.10 Discontinuation criteria for study or IDEC-C2B8 treatment ... 60
9.11 Withdrawal from the study ... 60
10 Concomitant medications and therapies/post-study treatment ... 61
10.1 Concomitant medications and therapies ... 61
10.2 Post-study treatment ... 62
11 Observations, tests, and investigations ... 62
11.1 Schedule of observations, tests, and investigations ... 62
11.2 Observations, tests, and investigations at screening ... 65
11.3 Investigations during the study treatment period of the blinded observation period ... 66
11.4 Investigations during the blinded observation period from the end of the study treatment period to the end of the blinded observation period ... 67
11.5 Investigations at the time of recurrence during the blinded observation period ... 68
11.6 Investigations at the time of confirmation of treatment criteria for IDEC-C2B8 (open-label period) treatment ... 69
11.7 Investigations during the IDEC-C2B8 treatment period of the open-label observation period ... 70
11.8 Investigations during the open-label observation period from the end of the IDEC-C2B8 treatment period to the end of the observation phase ... 71
11.9 Investigations at the time of recurrence during the open-label observation period ... 72
11.10 Investigations at the time of discontinuation of study or IDEC-C2B8 treatment ... 72
11.11 Investigations at the time of withdrawal from the study ... 73
11.12 Special notes for laboratory tests, blood drug concentration test, and HACA test ... 74
12 Data collection ... 75
13 Efficacy evaluation ... 75
13.1 Primary endpoint ... 75
13.2 Secondary endpoints ... 76
13.3 Other endpoints ... 77

14 Safety evaluation ..... 77
14.1 Adverse events ..... 77
14.2 Adverse event assessment and assessment criteria ..... 77
14.3 Follow-up of adverse events ..... 81
14.4 Expedited reporting of adverse events ..... 81
14.5 Major expected adverse reactions to the investigational product ..... 83
15 Statistical analysis ..... 87
15.1 Analysis population ..... 87
15.2 Target sample size and its rationale ..... 87
15.3 Analysis methods ..... 88
16 Direct access to source documents, etc. ..... 92
16.1 Acceptance of direct access ..... 92
16.2 Definition of source document ..... 92
17 Quality control and quality assurance ..... 92
17.1 Data quality control ..... 92
17.2 Audit ..... 93
17.3 Provision of new information ..... 93
18 Ethics ..... 93
18.1 Ethical conduct of the study ..... 93
18.2 Explanation and informed consent ..... 93
18.3 Institutional review board (IRB) ..... 94
18.4 Subject confidentiality ..... 94
19 Data handling and record retention ..... 95
19.1 Completion and reporting of the CRF ..... 95
19.2 Protocol deviation ..... 95
19.3 Data management ..... 95
19.4 Record retention ..... 95
20 Funding sources and conflicts of interest ..... 96
20.1 Funding sources ..... 96
20.2 Conflict of interest ..... 97
21 Payment and insurance ..... 97
21.1 Payment ..... 97
21.2 Compensation for injury ..... 97
22 Publication agreement ..... 97
23 Protocol amendment ..... 97
24 Change to the protocol or discontinuation or suspension of the study ..... 98
25 Study organization ..... 99
26 References ..... 100

27 Appendices........................................................................................................................ 102
Appendix 1. Standard height/weight table in 2000 ........................................................................ 103
Appendix 2. Height-specific prednisolone dose table...................................................................... 107
Appendix 3. Height-specific study drug dose table ........................................................................ 108
Appendix 4. Pediatric sex- and age-specific blood pressure norms table[22] .................................. 109
Appendix 5. Estimated glomerular filtration rate[23-25]........................................................................110
Appendix 6. Pediatric age-specific liver escape enzyme (GOT) norms table[26]...............................111
Appendix 7. Pediatric age-specific liver escape enzyme (GPT) norms table[26] ...............................112

[Revision history]

| | |
|---|---|
| Version 1.0 | 29 June 2018 |
| Version 1.1 | 25 July 2018 |
| Version 1.2 | 27 May 2019 |
| Version 2.0 | 2 December 2019 |
| Version 2.1 | 11 May 2020 |
| Version 3.0 | 8 September 2020 |
| Version 3.1 | 1 April 2021 |
| Version 3.2 | 2 August 2021 |
| Version 3.3 | 5 November 2021 |
| Version 3.4 | 14 January 2022 |

# 1 Overview of the study

## 1.1 Diagram of the study

This is a multicenter, double-blind, placebo-controlled, randomized, parallel-group study. However, a subject who is found to have been assigned to the placebo group after early key opening may receive IDEC-C2B8 in the open-label period if he/she wishes.

The study period for each subject is from the day of informed consent to the last day of the blinded observation period. For subjects who enter the open-label period and receive IDEC-C2B8 after early key opening, the study period will end on the last day of the open-label observation period.

The day of first study drug administration in the blinded period is designated as Day 1 of the blinded period, and the day of first IDEC-C2B8 administration in the open-label period is designated as Day 1 of the open-label period.

Table 1-1 Diagram of the study (without early key opening)

| | Screening phase | | | | | Observation phase | | | |
|---|---|---|---|---|---|---|---|---|---|
| | | | | | | Day 1 of the blinded period | Day 8 of the blinded period | •••••• | Day 365 of the blinded period |
| | | Screening Confirmation of eligibility | | | | | | | |
| Relapse | Informed consent | ←→ 35 days | | Enrollment/assignment | ←→ 14 days | First study drug administration | Second study drug administration | | End of the study |
| | | Remission | ←→ 7 days | | | | | | |

Table 1-2 Diagram of the study (with early key opening followed by standard treatment)

| | Screening phase | | | | | Observation phase | | | | | | | |
|---|---|---|---|---|---|---|---|---|---|---|---|---|---|
| | | | | | | Day 1 of the blinded period | Blinded period Day 8 | ••• | | | | ••• | Day 365 of the blinded period |
| | | Screening Confirmation of eligibility | | | | | | | | | | | |
| Relapse | Informed consent | ←→ 35 days | | Enrollment/assignment | ←→ 14 days | First study drug administration | Second study drug administration | | treatment failure (1) (2) (3) | Early key opening | Standard treatment | | End of the study |
| | | Remission | ←→ 7 days | | | | | | | | | | |

Table 1-3 Diagram of the study (with early key opening followed by switching to IDEC-C2B8 treatment [open-label period])

| | Screening phase | | | | |
|---|---|---|---|---|---|
| | | | | | |
| Relapse | Informed consent | Screening Confirmation of eligibility<br>←→<br>35 days | | Enrollment/assignment | ←→<br>14 days |
| | | Remission | ←→<br>7 days | | |

→ Followed by the table below

Continued from table above →

| Observation phase | | | | | | | | | | | | | |
|---|---|---|---|---|---|---|---|---|---|---|---|---|---|
| Blinded period | | | | Switching period | | | | | | Open-label period | | | |
| Day 1 | Day 8 | ••• | Up to Day 168 | | ••• | | | | | Day 1 | Day 8 | ••• | Day 365 |
| First study drug administration | Second study drug administration | | treatment failure (1) | Early key opening | | Remission | ←→<br>7 days | Confirmation of IDEC-C2B8 treatment criteria | ←→<br>14 days | First IDEC-C2B8 administration | Second IDEC-C2B8 administration | | End of the study |

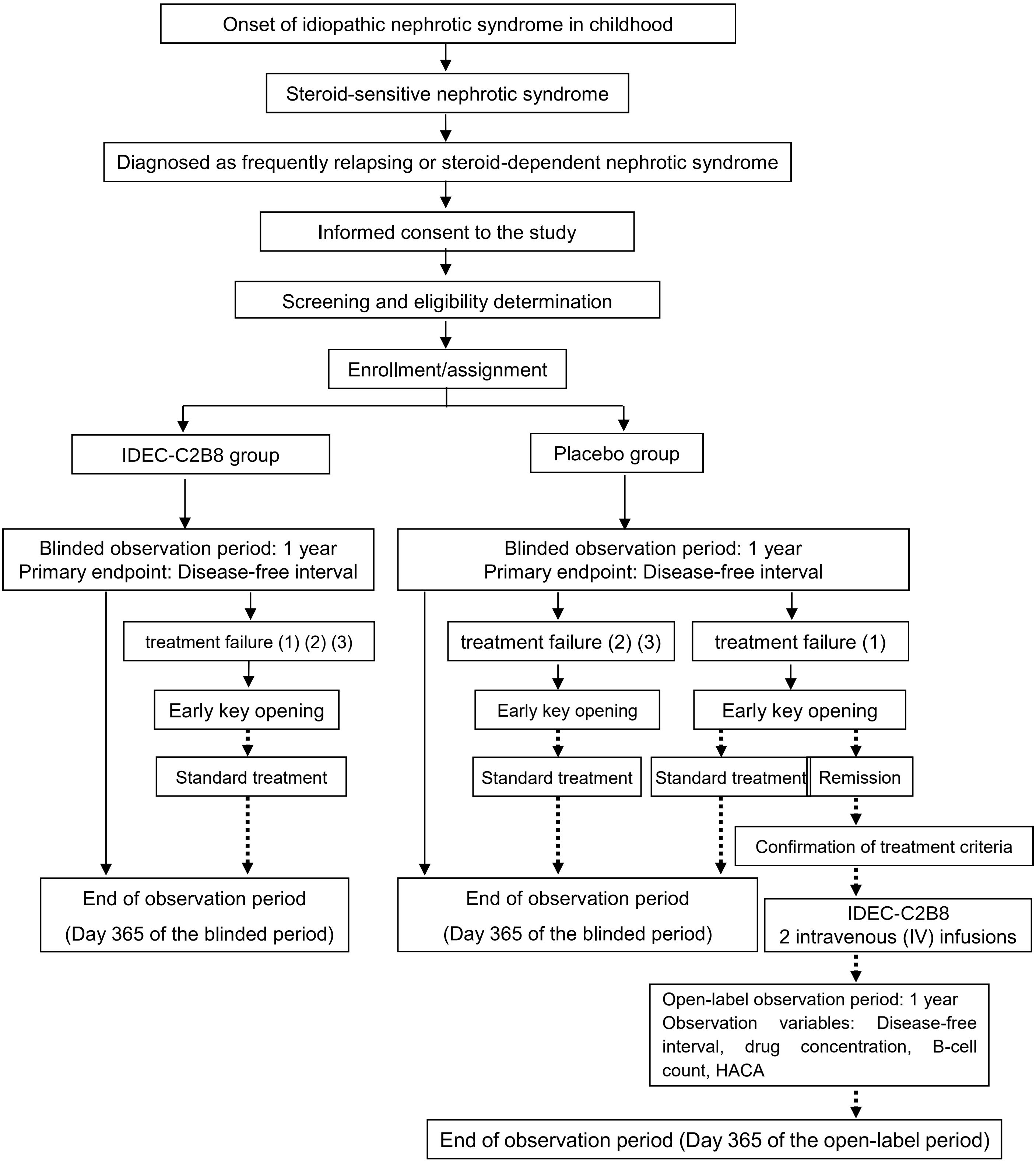


* IDEC-C2B8 treatment in the open-label period will be provided only to patients with treatment failure (1) (7.3.2).

Figure 1-1 Flowchart of the study

## 1.2 Objectives of the study

In patients with childhood-onset uncomplicated frequently relapsing or steroid-dependent nephrotic syndrome, the efficacy of the study drug administered at a dose of 375 mg/$m^2$ (up to 500 mg) once a week for two weeks will be verified, and the safety will be evaluated.

During the open-label period, the relapse-free period in the open-label observation period will be investigated. In addition, blood drug concentration and HACA will be measured to investigate the time course of blood drug concentration, HACA production status, and the relationship between the time course of peripheral B-cell count and relapse or adverse events after IDEC-C2B8 administration to patients with childhood-onset nephrotic syndrome at a dose of 375 mg/$m^2$ (up to 500 mg) twice.

### 1.2.1 Efficacy endpoints

(1) Primary endpoint

1) Relapse-free period during the blinded observation period (Days 1 to 365 of the blinded period)

It is defined as the time from the day of enrollment/assignment to the day of the first relapse after the start of study treatment during the blinded observation period (Days 1 to 365 of the blinded period). For relapse after the day of enrollment/assignment followed by confirmation of steroid sensitivity, the relapse-free period will start on the day of re-confirmation of eligibility.

(2) Secondary endpoints

1) Time to treatment failure

It is defined as the time from the day of enrollment/assignment to the day of determination of treatment failure during the blinded observation period (Days 1 to 365 of the blinded period). For relapse after the day of enrollment/assignment followed by confirmation of steroid sensitivity, the relpase-free period will start on the day of re-confirmation of eligibility.

2) Total steroid dose

It is defined as the total dose of steroid administered during the blinded observation period (Days 1 to 365 of the blinded period) after the day of confirmation of the last relapse before study treatment.

3) Change in peripheral B-cell count during the observation period (Days 1 to 365 of the blinded period and Days 1 to 365 of the open-label period)

i) Peripheral B-cell count (cells/μL)

ii) Time to peripheral B-cell count normalization

It is defined as the time from the day of confirmation of peripheral B-cell depletion during the observation period (Days 1 to 365 of the blinded period and Days 1 to 365 of the open-label period) to confirmation of peripheral B-cell count normalization (normal level ≥ 5 cells/μL).

(3) Other endpoints

1) Relapse-free period during the open-label period

It is defined as the time from the day of confirmation of IDEC-C2B8 treatment criteria to the day of

the first relapse after the start of IDEC-C2B8 treatment.

2) Blood IDEC-C2B8 concentration

3) HACA production status

#### 1.2.2 Safety endpoints

Occurrence of adverse events (AEs) and adverse drug reactions (ADRs)

### 1.3 Study population

Patients with childhood-onset idiopathic nephrotic syndrome diagnosed as frequently relapsing or steroid-dependent who have not started immunosuppressive therapy

#### 1.3.1 Inclusion and exclusion criteria

See 5, "Subject inclusion and exclusion criteria."

### 1.4 Target sample size and planned study period

Target sample size: 40 subjects (20 subjects per group)

Planned enrollment period: From November 2018 to March 2021 (29 months)

Planned study period: From November 2018 to September 2022 (47 months)

Blinded period From November 2018 to March 2022 (41 months)

## 1.5 Study schedule

See 11.1, "Schedule of Observations, tests, and Investigations."

Table 1-4 Study schedule in the blinded period (* also applicable to early key opening followed by standard treatment)

| | Screening phase*1 | Blinded observation period (study treatment period) | | Blinded observation period (after study treatment) | | | | | | | | | | | | | | Withdrawal from the study |
|---|---|---|---|---|---|---|---|---|---|---|---|---|---|---|---|---|---|---|
| Day | | 1 | 8 | 29 | 57 | 85 | 113 | 141 | 169 | 197 | 225 | 253 | 281 | 309 | 337 | 365 | Relapse | |
| Week | | 1 | 2 | 5 | 9 | 13 | 17 | 21 | 25 | 29 | 33 | 37 | 41 | 45 | 49 | 53 | | |
| Month (4 weeks/month) | | | | 1 | 2 | 3 | 4 | 5 | 6 | 7 | 8 | 9 | 10 | 11 | 12 | | | |
| Informed consent | ○ | | | | | | | | | | | | | | | | | |
| Subject demographics | ○ | | | | | | | | | | | | | | | | | |
| Study drug administration | | ○ | ○ | | | | | | | | | | | | | | | |
| Medical examination | ○ | ○ | ○ | ○ | ○ | ○ | ○ | ○ | ○ | ○ | ○ | ○ | ○ | ○ | ○ | ○ | ○ | ○ |
| Concomitant medication survey | ○ | ○ | ○ | ○ | ○ | ○ | ○ | ○ | ○ | ○ | ○ | ○ | ○ | ○ | ○ | ○ | ○ | ○ |
| Patient diary | ○ | ○ | ○ | ○ | ○ | ○ | ○ | ○ | ○ | ○ | ○ | ○ | ○ | ○ | ○ | ○ | ○ | |
| Height/weight | ○ | ○ | ○ | ○ | ○ | ○ | ○ | ○ | ○ | ○ | ○ | ○ | ○ | ○ | ○ | ○ | ○ | ○ |
| Blood pressure, pulse, body temperature | ○ | ○ | ○ | ○ | ○ | ○ | ○ | ○ | ○ | ○ | ○ | ○ | ○ | ○ | ○ | ○ | ○ | ○ |
| Pregnancy test | ○*2 | | | | | | | | | | | | | | | | | |
| Virology | ○ | | | | | | | | | | | | | | | | | |
| Electrocardiography | ○ | | | | | | | | | | | | | | | ○ | | ○ |
| Chest X-ray | ○ | | | | | | | | | | | | | | | ○ | | ○ |
| Recurrence assessment | | ○ | ○ | ○ | ○ | ○ | ○ | ○ | ○ | ○ | ○ | ○ | ○ | ○ | ○ | ○ | ○ | ○ |
| Adverse event assessment | | ○ | ○ | ○ | ○ | ○ | ○ | ○ | ○ | ○ | ○ | ○ | ○ | ○ | ○ | ○ | ○ | ○ |
| Hematology | ○*3 | ○ | ○ | ○ | ○ | ○ | ○ | ○ | ○ | ○ | | ○ | | ○ | | ○ | ○ | ○ |
| Serum chemistry | ○*3 | ○ | ○ | ○ | ○ | ○ | ○ | ○ | ○ | ○ | | ○ | | ○ | | ○ | ○ | ○ |
| Immunoglobulins | | ○ | | ○ | | ○ | | | ○ | | | ○ | | | | ○ | | ○ |
| eGFR | ○ | | | | | | | | | | | | | | | ○ | | ○ |
| Urinalysis | ○ | ○ | ○ | ○ | ○ | ○ | ○ | ○ | ○ | ○ | ○ | ○ | ○ | ○ | ○ | ○ | ○ | ○ |
| Peripheral B-cell count*4*5 | ○ | ● | | ● | | ● | | ● | | ● | | ● | | ● | | ● | ● | ● |

○ Measured/performed locally ● Measured externally (central measurement)

*1 Performed after informed consent and within 35 days before enrollment, except for hematology and serum chemistry
*2 Confirmed by serum or urine HCG test in postmenarchal females only
*3 Performed after informed consent and within 14 days before enrollment. Any test data collected after the last recurrence and within 14 days before enrollment may be used even if collected before informed consent, requiring no re-examination.
*4 During the screening phase, the CD20-positive or CD19-positive cell count will be measured locally. During the blinded observation period, the CD19-positive cell count will be measured centrally.
*5 It is not necessary to measure peripheral B cells after treatment failure in subjects in the placebo group who are determined as treatment failure (1) and switched to standard treatment or subjects in the placebo group who are determined as treatment failure (2) or (3).

Table 1-5 Study schedule in the open-label period (IDEC-C2B8 treatment)

| | Switching period | Open-label observation period (IDEC-C2B8 treatment period) | | Open-label observation period (after the end of IDEC-C2B8 treatment) | | | | | | | | | | | | | | Withdrawal from the study |
|---|---|---|---|---|---|---|---|---|---|---|---|---|---|---|---|---|---|---|
| Day | | 1 | 8 | 29 | 57 | 85 | 113 | 141 | 169 | 197 | 225 | 253 | 281 | 309 | 337 | 365 | Relapse | |
| Week | | 1 | 2 | 5 | 9 | 13 | 17 | 21 | 25 | 29 | 33 | 37 | 41 | 45 | 49 | 53 | | |
| Month (4 weeks/month) | | | | 1 | 2 | 3 | 4 | 5 | 6 | 7 | 8 | 9 | 10 | 11 | 12 | | | |
| Confirmation of treatment | ○*1 | | | | | | | | | | | | | | | | | |
| IDEC-C2B8 administratio | | ○*2 | ○ | | | | | | | | | | | | | | | |
| Medical examination | ○ | ○ | ○ | ○ | ○ | ○ | ○ | ○ | ○ | ○ | ○ | ○ | ○ | ○ | ○ | ○ | ○ | ○ |
| Concomitant medication survey | ○ | ○ | ○ | ○ | ○ | ○ | ○ | ○ | ○ | ○ | ○ | ○ | ○ | ○ | ○ | ○ | ○ | ○ |
| Patient diary | ○ | ○ | ○ | ○ | ○ | ○ | ○ | ○ | ○ | ○ | ○ | ○ | ○ | ○ | ○ | ○ | ○ | |
| Height/weight | ○ | ○ | ○ | ○ | ○ | ○ | ○ | ○ | ○ | ○ | ○ | ○ | ○ | ○ | ○ | ○ | ○ | ○ |
| Blood pressure, pulse, body temperature | ○ | ○ | ○ | ○ | ○ | ○ | ○ | ○ | ○ | ○ | ○ | ○ | ○ | ○ | ○ | ○ | ○ | ○ |
| Pregnancy test | ○*3 | | | | | | | | | | | | | | | | | |
| Virology | ○*4 | | | | | | | | | | | | | | | | | |
| Electrocardiography | ○ | | | | | | | | | | | | | | | ○ | | ○ |
| Chest X-ray | ○ | | | | | | | | | | | | | | | ○ | | ○ |
| Recurrence assessment | | ○ | ○ | ○ | ○ | ○ | ○ | ○ | ○ | ○ | ○ | ○ | ○ | ○ | ○ | ○ | ○ | ○ |
| Adverse event assessment | ○ | ○ | ○ | ○ | ○ | ○ | ○ | ○ | ○ | ○ | ○ | ○ | ○ | ○ | ○ | ○ | ○ | ○ |
| Hematology | ○*5 | ○ | ○ | ○ | ○ | ○ | ○ | ○ | ○ | ○ | | ○ | | ○ | | ○ | ○ | ○ |
| Serum chemistry | ○*5 | ○ | ○ | ○ | ○ | ○ | ○ | ○ | ○ | ○ | | ○ | | ○ | | ○ | ○ | ○ |
| Immunoglobulins | | ○ | | ○ | | ○ | | | ○ | | | ○ | | | | ○ | | ○ |
| eGFR | ○ | | | | | | | | | | | | | | | ○ | | ○ |
| Urinalysis | ○ | ○ | ○ | ○ | ○ | ○ | ○ | ○ | ○ | ○ | ○ | ○ | ○ | ○ | ○ | ○ | ○ | ○ |
| Peripheral B-cell count*6 | ○*7 | ● | | ● | | ● | | ● | | ● | | ● | | ● | | ● | ● | ● |
| HACA | | ●*8 | | | | ● | | | ● | | | ● | | | | ● | ● | ● |
| Blood drug concentration | | ●*9 | ●*9 | ● | | ● | ● | | ● | | | ● | | | | ● | | ● |

○ Measured/performed locally ● Measured externally (central measurement)

*1 Within 7 days from the day of confirmation of remission
*2 Within 14 days from the day of confirmation of IDEC-C2B8 treatment criteria
*3 Confirmed by serum or urine HCG test in postmenarchal females only
*4 No re-examination will be required if performed within 85 days before confirmation of IDEC-C2B8 treatment criteria.
*5 Performed within 14 days before confirmation of IDEC-C2B8 treatment criteria
*6 During the switching period, the CD20-positive or CD19-positive cell count will be measured locally. During the open-label observation period, the CD19-positive cell count will be measured centrally.
*7 No re-measurement will be required if measured within 35 days before confirmation of IDEC-C2B8 treatment criteria.
*8 Measured before IDEC-C2B8 administration
*9 Measured immediately before IDEC-C2B8 administration and 30 minutes after the end of IDEC-C2B8 administration

## 1.6 Abbreviations

| Abbreviation | |
|---|---|
| ANCA | Antineutrophil Cytoplasmic Antibody |
| CRF | Case Report Form |
| CTCAE | Common Terminology Criteria for Adverse Events |
| EDC | Electronic Data Capture |
| FAS | Full Analysis Set |
| GCP | Good Clinical Practice |
| HACA | Human Anti- Chimeric Antibody |
| INN | International Non-proprietary Names |
| ISKDC | International Study of Kidney Disease in Children |
| JAN | Japanese Accepted Names |
| JCOG | Japan Clinical Oncology Group |
| MedDRA | Medical Dictionary for Regulatory Activities |
| PPS | Per Protocol Set |
| PML | Progressive Multifocal Leukoencephalopathy |
| SAS | Safety Analysis Set |
| TPN | Total Parenteral Nutrition |
| UAS | Unblined Analysis Set |
| ULN | Upper Limit of Normal, |

## 1.7 Abbreviations (test parameters)

| Abbreviation | |
|---|---|
| ALT(GPT) | L-alanine aminotransferase<br>Glutamic Pyruvic Transaminase |
| Al-P | Alkaline Phosphatase |
| AST(GOT) | Aspartate aminotransferase<br>Glutamic Oxaloacetic Transaminase |
| AUC | Area Under the Curve |
| BUN | Blood Urea Nitrogen |
| Ca | Calcium |
| Cl | Chlorine |
| Cmax | Maximum Drug Concentration |
| CRP | C-Reactive Protein |
| eGFR | Estimate Glomerular Filtration Rate |
| FDP | Fibrinogen/Fibrin Degradation Products |
| Hb | Hemoglobin |
| HBc antibody | Hepatitis B core Antibody |
| HBs antigen | Hepatitis B surface Antigen |
| HBs antibody | Hepatitis B surface Antibody |
| HBV | Hepatitis B virus |
| HCG | Human Corionic Gonadotropin |
| HCV | Hepatitis C virus |
| HIV | Human Immunodeficiency Virus |
| Ht | Hematocrit |
| IgA | Immunoglobulin A |
| IgG | Immunoglobulin G |
| IgM | Immunoglobulin M |
| K | Kalium |
| LDH | Lactate Dehydrogenase |
| Na | Natrium |
| P | Phosphorus |
| Plt | Platelet |
| RBC | Red Blood Cell |
| $T_{1/2}$ | T-half |
| WBC | White Blood Cell |

## 1.8 Definitions

In this study, terms are defined as follows according to the definitions in the Clinical Practice Guideline for Pediatric Idiopathic Nephrotic Syndrome 2013 (Japanese Society for Pediatric Nephrology; hereinafter referred to as the Clinical Practice Guideline):

| | |
|---|---|
| Nephrotic syndrome | Severe proteinuria (≥ 40 mg/h/m$^2$ in nocturnal urine) or morning urine protein/creatinine ratio ≥ 2.0 g/gCr and hypoalbuminemia (serum albumin ≤ 2.5 g/dL) |
| Remission | Negative morning urine protein dipstick for 3 consecutive days or morning urine protein/creatinine ratio < 0.2 g/gCr for 3 consecutive days |
| Date of confirmation of remission | Date when remission is confirmed at the study site |
| Steroid sensitivity | Remission within 4 weeks of starting daily prednisolone treatment at a dose of 60 mg/m$^2$/day |
| Relapse | Any of the following conditions requiring prednisolone treatment:<br>[1] Morning urine protein dipstick ≥ 3+ (or ≥ 300 mg/dL in quantitative urine protein test) for 3 consecutive days<br>[2] Urine protein dipstick ≥ 2+ (or ≥ 100 mg/dL in quantitative urine protein test) and serum albumin ≤ 3.0 g/dL |
| Date of relapse | The first date of morning urine protein dipstick ≥ 3+ (or ≥ 300 mg/dL in quantitative urine protein test) for 3 consecutive days or date of urine protein dipstick ≥ 2+ (or ≥ 100 mg/dL in quantitative urine protein test) and serum albumin ≤ 3.0 g/dL (or date of diagnosis of relapse for the last 3 relapses before enrollment) |
| Frequent relapse | At least 2 relapses within 6 months of the first remission or at least 4 relapses within any 12-month period |
| Date of frequent relapse | Date of relapse meeting the definition of frequent relapse (date of the second relapse for at least 2 relapses within 6 months of the first remission or date of the fourth relapse for at least 4 relapses within any 12 months) |
| Steroid dependence | Two consecutive relapses within 2 weeks after dose reduction or discontinuation of prednisolone |
| Date of steroid dependence | Date of the second relapse meeting the definition of steroid dependence |
| Steroid resistance | Failure to achieve remission despite at least 4 weeks of daily prednisolone treatment at a dose of 60 mg/m$^2$/day |
| Date of steroid resistance | Date when failure to achieve remission despite 4 weeks of daily prednisolone treatment at a dose of 60 mg/m$^2$/day is confirmed at the study site |
| Complicated frequently relapsing/steroid-dependent nephrotic syndrome | Patients who meet any of the following [1] to [4]:<br>[1] The disease was diagnosed as frequently relapsing or steroid-dependent and is then again diagnosed as frequently relapsing or steroid-dependent after immunosuppressive therapy (e.g., cyclosporine, cyclophosphamide, mizoribine*).<br>[2] The disease was diagnosed as frequently relapsing or steroid-dependent and is then again diagnosed as frequently relapsing or steroid-dependent during immunosuppressive therapy (e.g., cyclosporine, cyclophosphamide, mizoribine*).<br>[3] The disease was diagnosed as steroid-resistant and is then diagnosed as frequently relapsing or steroid-dependent after immunosuppressive therapy (e.g., cyclosporine alone or in combination with methylprednisolone).<br>[4] The disease was diagnosed as steroid-resistant and is then diagnosed as frequently relapsing or steroid-dependent during immunosuppressive therapy (e.g., cyclosporine alone or in combination with methylprednisolone).<br>* Only when mizoribine is used in combination with other immunosuppressants |

# 2 Introduction

## 2.1 Background information

### 2.1.1 Childhood-onset nephrotic syndrome

Nephrotic syndrome refers to glomerular basement membrane damage in the kidney that results in protein leakage from blood into urine, leading to severe proteinuria, hypoproteinemia, and generalized edema. The etiology has not been sufficiently clarified, although it is considered that cytokines produced by T cells may act on the slit membrane to increase protein permeability.

Childhood-onset nephrotic syndrome commonly occurs in infants aged 2 to 6 years and often manifests as eyelid and lower leg edema. Idiopathic nephrotic syndrome of unknown etiology accounts for approximately 90% of cases.

### 2.1.2 Treatment of childhood-onset idiopathic nephrotic syndrome

The first-line treatment at the onset of childhood-onset idiopathic nephrotic syndrome is oral corticosteroids (hereinafter referred to as steroids). Approximately 80% to 90% of patients achieve remission with steroids (steroid-sensitive nephrotic syndrome). However, the disease recurs in 80% of these patients, and in half of them (approximately 30% to 40% of patients with childhood-onset idiopathic nephrotic syndrome), it progresses to frequently relapsing nephrotic syndrome, which repeatedly recurs over a relatively short period of time, or steroid-dependent nephrotic syndrome, which recurs with dose reduction or discontinuation of steroids.[1,2] In frequently relapsing or steroid-dependent nephrotic syndrome, long-term continuous use of steroids causes adverse reactions such as failure to thrive, obesity, diabetes mellitus, cataract, glaucoma, hypertension, osteoporosis, and necrosis of the femoral head, and it is therefore recommended to use immunosuppressants for the purpose of dose reduction or withdrawal from steroids. In Japan, immunosuppressants recommended in the Clinical Practice Guideline are cyclosporine (blood trough concentrations of 80 to 100 ng/mL for 6 months, followed by 60 to 80 ng/mL), cyclophosphamide (at doses of 2 to 2.5 mg/kg/day [up to 100 mg] for 8 to 12 weeks), and mizoribine (at high doses of 7 to 10 mg/kg/day).[3]

While cyclosporine can be used to reduce or discontinue steroids in most patients, it has been reported that the disease recurs after discontinuation of cyclosporine, repeatedly recurs due to decreased sensitivity to cyclosporine during therapy, or does not respond to cyclosporine when resumed after discontinuation due to good response in many of them. Among adverse reactions to cyclosporine, chronic nephrotoxicity is the most serious problem, and the incidence of chronic nephrotoxicity increases when it is administered at moderate doses (trough concentration of approximately 100 ng/mL) for 2 years or more,[4] requiring caution when it is used for a long period of time or re-administered. Since chronic nephrotoxicity of cyclosporine cannot be adequately diagnosed only by urine or blood test, renal biopsy at intervals of 2 to 3 years is recommended to assess nephrotoxicity,[3] posing a great burden on patients.

Cyclophosphamide has been demonstrated in multiple randomized controlled studies to be useful in the treatment of frequently relapsing nephrotic syndrome, but has been reported in a Japanese randomized controlled study to be of limited efficacy for steroid-dependent nephrotic syndrome.[3] Significant adverse reactions include gonadal disorders, particularly azoospermia in boys, to which adolescent and post-adolescent patients are more

vulnerable.[5)] Since the incidence of gonadal disorders such as azoospermia increases at a cumulative dose of > 300 mg/kg,[3)] it is difficult to administer it for a long period of time or re-administer it.

Mizoribine has been shown to be highly safe, but has not been demonstrated to be very effective in preventing recurrence,[6)] and high-dose mizoribine requires large-scale prospective controlled studies to evaluate the efficacy and safety.

Complicated nephrotic syndrome is defined as a disease that remains frequently relapsing or steroid-dependent without withdrawal from steroids due to failure of standard immunosuppressive therapy for maintaining remission. Only rituximab (4 doses) is currently approved as a standard treatment for complicated frequently relapsing or steroid-dependent nephrotic syndrome.

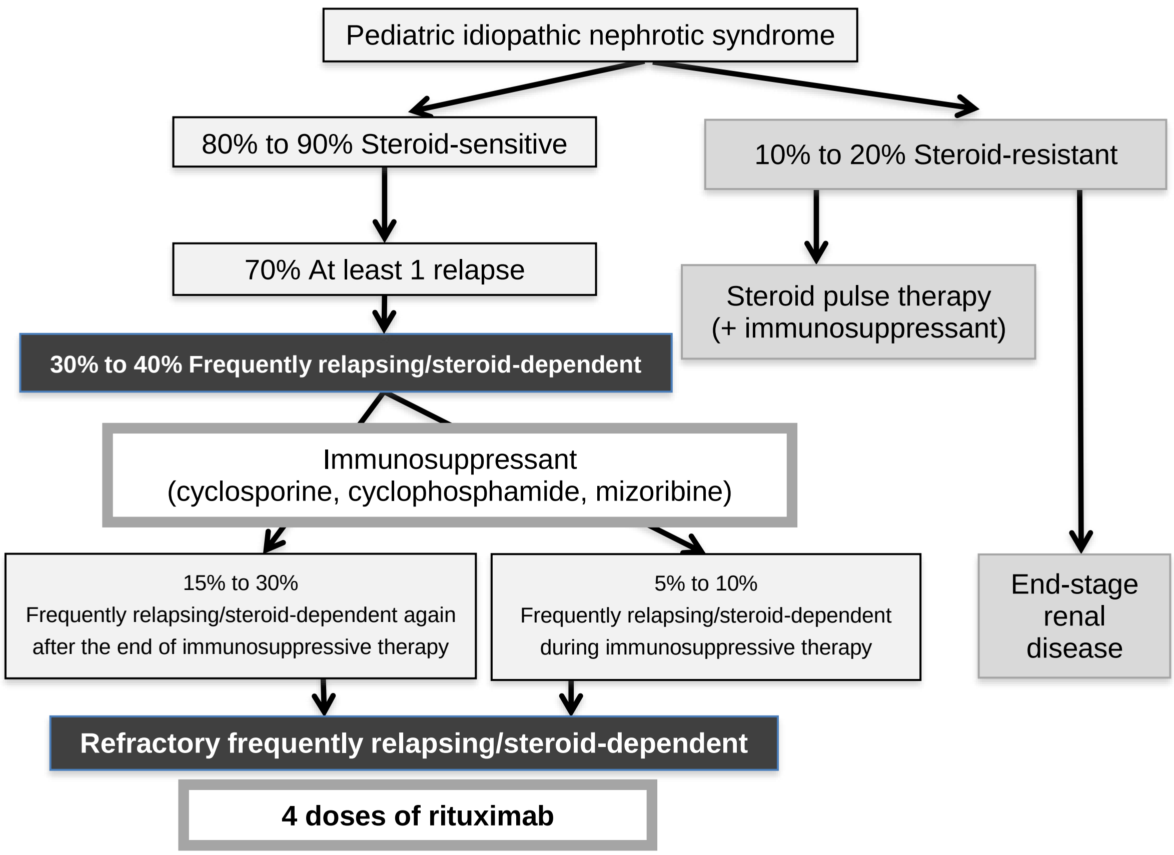


Figure 2-1 Course and treatment of nephrotic syndrome

### 2.1.3 Development of IDEC-C2B8 in childhood-onset nephrotic syndrome

The test drug IDEC-C2B8 (rituximab [genetic recombination]) is a monoclonal antibody against CD20, a differentiation antigen expressed on the B cell surface, and a chimeric anti-CD20 monoclonal antibody consisting of the constant region of a human immunoglobulin (IgG1κ) and the variable region of a mouse anti-CD20 antibody.

IDEC-C2B8, which specifically damages CD20-positive B cells and is therefore considered to be effective for various diseases caused by B cell abnormalities,[7, 8)] has been approved in Japan for the treatment of CD20-positive B-cell non-Hodgkin's lymphoma, CD20-positive B-cell lymphoproliferative disorder in

immunocompromised patients, and ANCA associated vasculitis, and has been approved in more than 100 countries worldwide, including Japan, the US, and Europe.

A multicenter, double-blind, placebo-controlled, randomized study of IDEC-C2B8 in childhood-onset complicated frequently relapsing or steroid-dependent nephrotic syndrome (RCRNS-01)[9)] conducted by Iijima et al. demonstrated the superiority of IDEC-C2B8 over placebo in terms of relapse-free period, the primary endpoint. Based on these results, IDEC-C2B8 was approved for the treatment of compicated nephrotic syndrome (frequently relapsing or steroid-dependent) in Japan in 2014.

The development of IDEC-C2B8 in compicated nephrotic syndrome (frequently relapsing or steroid-dependent) was initiated because of concerns for short stature and osteoporosis associated with long-term continuous steroid therapy, needs to treat patients whose disease does not respond to immunosuppressants or becomes frequently relapsing or steroid-dependent again after discontinuation of immunosuppressants, and concerns for patients who experience adverse reactions to immunosuppressants in childhood-onset nephrotic syndrome, etc.. Given case reports in non-Japanese academic journals and international congresses that IDEC-C2B8 treatment of childhood-onset complicated frequently relapsing or steroid-dependent nephrotic syndrome prevented relapse of nephrotic syndrome and allowed dose reduction or withdrawal from steroids and immunosuppressants,[10-15)] the development was supported by expectations for this new effective and safe drug for childhood-onset complicated frequently relapsing or steroid-dependent nephrotic syndrome.

In recent years, study results suggesting the efficacy of IDEC-C2B8 for uncomplicated frequently relapsing or steroid-dependent disease have been reported. Among patients with steroid-dependent nephrotic syndrome, the relapse occured within 6 months in 2 of 15 patients who received IDEC-C2B8 at a single dose of 375 mg/m$^2$ versus 14 of 15 patients under standard steroid treatment only.[16)] During long-term follow-up of subjects in a clinical trial in complicated frequently relapsing or steroid-dependent nephrotic syndrome conducted by Iijima et al. over approximately 5 years (median 59 months), 5 of 13 subjects receiving no immunosuppressant concomitantly with the first dose of IDEC-C2B8 required no additional dose of IDEC-C2B8 or resumption of immunosuppressant, and 3 experienced no relapse. On the other hand, all of 46 subjects receiving an immunosuppressant concomitantly with the first dose of IDEC-C2B8 experienced relapse and required an additional dose of immunosuppressant and/or IDEC-C2B8.[17)]

Immunosuppressants such as cyclosporine, cyclophosphamide, and mizoribine are recommended for the treatment of frequently relapsing or steroid-dependent nephrotic syndrome.[3,18)] However, these drugs cause serious adverse reactions and need to be administered orally every day or every other day on a long-term basis, impairing medication adherence. On the other hand, IDEC-C2B8 is considered to be relatively well tolerated, with no deaths or life-threatening serious adverse events reported as far as previous safety-related findings in complicated frequently relapsing or steroid-dependent nephrotic syndrome (results of RCRNS-01) are concerned. In addition, since it requires only several IV infusions and therefore has no problem with medication adherence, it may be clinically useful in pediatric patients diagnosed with uncomplicated frequently relapsing or steroid-dependent nephrotic syndrome.

Based on the belief that these facts show that IDEC-C2B8 may serve as a standard treatment for uncomplicated frequently relapsing or steroid-dependent nephrotic syndrome, the present development plan was drafted.

While it has been reported that patients with the first episode of pediatric idiopathic nephrotic syndrome have a significantly higher risk of developing frequently relapsing or steroid-dependent nephrotic syndrome in the future if (1) the time to remission is ≥ 9 days with standard steroid treatment and (2) the time from remission to the first relapse is < 6 months,[19)] the risk is unpredictable immediately after the onset. In addition, it has not been reported in the world that IDEC-C2B8 was administered from early on to treat the first episode, and there is a consensus among pediatric nephrologists around the world that IDEC-C2B8 should not be administered as initial therapy for the first episode of pediatric idiopathic nephrotic syndrome.

## 2.2 Drug information

### 2.2.1 Test drug IDEC-C2B8

As for the mechanism of action of IDEC-C2B8, it has been demonstrated in *in vitro* studies that it binds to the CD20 antigen on the B-cell surface to specifically damage B cells by complement-dependent cytotoxicity and antibody-dependent cell-mediated cytotoxicity and thereby eliminate B cells from the body.

The dosage and administration of IDEC-C2B8 (rituximab [genetic recombination]) for the indications already approved in Japan are presented below.

(1) CD20-positive B-cell non-Hodgkin's lymphoma

The usual adult dosage is 375 mg/m$^2$ of rituximab (genetic recombination) administered by IV infusion every week. Up to 8 doses may be administered. When co-administered with any other anti-neoplastic agent, rituximab (genetic recombination) should be administered once per cycle according to the dosing interval of the concomitant anti-neoplastic agent.

For maintenance therapy, the usual adult dosage is 375 mg/m$^2$ of rituximab (genetic recombination) administered by IV infusion. The dosing interval is approximately 8 weeks and up to 12 doses may be administered.

(2) CD20-positive chronic lymphocytic leukemia

When co-administered with any other anti-neoplastic agent, the usual adult dosage is 375 mg/m$^2$ of rituximab (genetic recombination) administered by IV infusion as the initial dose and then 500 mg/m$^2$ for the second and subsequent doses administered once per cycle according to the dosing interval of the concomitant anti-neoplastic agent. Up to 6 doses may be administered.

(3) CD20-positive B-cell lymphoproliferative disorder in immunocompromised patients

The usual dosage is 375 mg/m$^2$ of rituximab (genetic recombination) administered by IV infusion every week. Up to 8 doses may be administered.

(4) Granulomatosis with polyangiitis, microscopic polyangiitis, chronic idiopathic thrombocytopenic purpura, acquired thrombotic thrombocytopenic purpura, and systemic scleroderma

The usual adult dosage is 375 mg/m$^2$ of rituximab (genetic recombination) administered by IV infusion once a week for 4 weeks.

(5) Complicated nephrotic syndrome (frequently relapsing or steroid-dependent)

The usual dosage is 375 mg/m$^2$ of rituximab (genetic recombination) administered by IV infusion once

a week for 4 weeks. Up to 500 mg may be administered per dose.

(6) Refractory pemphigus vulgaris and pemphigus foliaceus

The usual adult dosage is 1,000 mg/body of rituximab (genetic recombination) administered by IV infusion once a week for 2 weeks.

(7) Suppression of antibody-related rejection in ABO-incompatible kidney/liver transplantation

The usual dosage is 375 mg/m$^2$ of rituximab (genetic recombination) administered by IV infusion. The dose may be reduced according to the patient's condition.

(8) Premedication for indium ($^{111}$In) ibritumomab tiuxetan (genetic recombination) injection and yttrium ($^{90}$Y) ibritumomab tiuxetan (genetic recombination) injection

The usual adult dosage is 250 mg/m$^2$ of rituximab (genetic recombination) administered by IV infusion once.

### 2.2.2 Indication to be acquired based on the results of this study

The indication to be acquired based on the results of this study is "childhood-onset nephrotic syndrome (frequently relapsing or steroid-dependent)."

### 2.2.3 Nonclinical studies

A summary of results of nonclinical studies conducted with IDEC-C2B8 to date is provided in Table 2-1.

Table 2-1 Results of nonclinical studies of IDEC-C2B8

| Type of study | Species/strain | Mode of administration | Duration of treatment | Dose (mg/kg[A]) | GLP compliance |
|---|---|---|---|---|---|
| Single dose toxicity study | Cynomolgus monkey | Intravenous | Single dose | 10, 30, 100 | Compliant |
| Repeat dose toxicity study | Cynomolgus monkey | Intravenous | 1 month (4 weekly doses) | 0, 0.25, 2.1, <u>16.8</u> | Compliant |
| Repeat dose toxicity study | Cynomolgus monkey | Intravenous | 2 months (4 or 8 weekly doses) | 0, <u>20</u> | Compliant |
| Genotoxicity study | Not conducted | | | | |
| Carcinogenicity study | Not conducted | | | | |
| Reproductive and developmental toxicity study | See 2.2.4, "Maternal and fetal effects." | | | | |
| Local tolerance test[B] (vascular irritation) | Cynomolgus monkey | Intravenous | 2 months (8 weekly doses) | 0, 20 | Compliant |
| Pyrogen test | Japanese white rabbit | Intravenous | Single dose | 0, 30, 50, 100 | Compliant |
| Cross-reactivity test with normal human tissues | Normal human tissues | *In vitro* | - | 10 μg/mL | Compliant |

A The underlined dose in the repeated dose toxicity study is the no observed adverse effect level (NOAEL), unless otherwise stated.
B Based on the histopathology of the injection site in the 2-month repeated dose toxicity study

### 2.2.4 Maternal and fetal effects

Since IDEC-C2B8 is a monoclonal antibody and is essentially homogeneous to immunoglobulins, placental

transfer to the fetus may occur in a mother treated with it.

The fetal effects of IDEC-C2B8 administered during organogenesis were investigated in cynomolgus monkeys. After intravenous administration to cynomolgus monkeys during gestation, parturition, or lactation, a decrease in B cells was observed in dams and fetuses, but IDEC-C2B8 was well tolerated at doses up to 100 mg/kg and did not appear to affect the incidence of prenatal death or stillbirth. After intravenous administration to pregnant cynomolgus monkeys during organogenesis (Days 20 to 50 of gestation), a decrease in lymphocytes was observed, but IDEC-C2B8 was well tolerated at doses up to 100 mg/kg with no significant difference in the abortion rate or fetal mortality compared with the control group and appeared to be devoid of maternal toxicity, fetal toxicity, or teratogenicity.

In B cell knockout mice, which inherently lack B cells, hypoplasia of lymphoid tissues such as lymph nodes and Peyer's patch results in reduced resistance to exogenous antigens such as viruses, indicating that B cells are essential for the formation of lymphoid tissues in fetuses.

Based on the above, when IDEC-C2B8 is administered to a pregnant mother, it may be transferred through the placenta to the fetus and damage B cells of the fetus, and lack of B cells during fetal organogenesis may result in hypoplasia of lymphoid tissues.

It has been reported that a patient with B-cell lymphoma who received IDEC-C2B8 in combination with an anticancer agent at Week 21 of gestation safely delivered a girl by cesarean at Week 35 of gestation.

### 2.2.5 Japanese clinical trials in patients with complicated nephrotic syndrome

An overview of Japanese clinical trials in childhood-onset complicated nephrotic syndrome is provided below.

Table 2-2 Overview of a multicenter, double-blind, placebo-controlled, randomized study of IDEC-C2B8 in childhood-onset complicated nephrotic syndrome (RCRNS-01)

| | |
|---|---|
| Title of the study: A multicenter, double-blind, placebo-controlled, randomized study of IDEC-C2B8 in childhood-onset complicated nephrotic syndrome | |
| Names of investigators: Kunihiko Aya, Kenji Ishikura, Shuichi Ito, Yoshiyuki Ohtomo, Hiroshi Kaito, Koichi Kamei, Shouri Takahashi,<br>Ryojiro Tanaka, Koichi Nakanishi, Kandai Nozu, and Kenichiro Miura (a total of 11 investigators) | |
| Study sites: A total of 9 sites, including Wakayama Medical University Hospital, National Center for Child Health and Development, Okayama University Hospital, Kobe University Hospital, and the University of Tokyo Hospital | |
| Publication (citation):<br>Iijima K, Sako M, Nozu K, Mori R, Tuchida N, Kamei K, Miura K, Aya k, Nakanishi K, Ohtomo Y, Takahashi S, Tanaka R, Kato H, Nakamura H, Ishikura K, Ito S, Ohashi Y. Rituximab for childhood-onset, complicated, frequently relapsing nephrotic syndrome or steroid-dependent nephrotic syndrome: a multicentre, double-blind, randomised, placebo-controlled trial: Lancet 2014; 384: [9)] | |
| Duration of the study (years): (4 years) | Phase of development: |

| Date of first subject's informed consent 06 November 2008<br>Date of completion of last subject's observation 10 November 2011 | Phase 3 |
|---|---|
| Objectives: To verify the efficacy and safety of IDEC-C2B8 in patients with childhood-onset complicated nephrotic syndrome by comparing IDEC-C2B8 administered at a dose of 375 mg/m$^2$ (up to 500 mg) 4 times with placebo | |
| Study method: A multicenter, double-blind, placebo-controlled, randomized study | |
| Number of subjects (planned and analyzed):<br>Planned: A total of 60 subjects (30 subjects in the IDEC-C2B8 group and 30 subjects in the placebo group)<br>Analyzed: A total of 52 subjects (27 subjects in the IDEC-C2B8 group and 25 subjects in the placebo group)<br>Of randomized subjects, a total of 48 (24 subjects per group) were included in the full analysis set (FAS), a total of 46 (23 subjects per group) were included in the per protocol set (PPS), and a total of 48 (24 subjects per group) were included in the safety analysis set. Since enrollment only in the IDEC-C2B8 group was continued after the completion of randomization, the total number of subjects increased to 59. The efficacy and safety were also investigated separately in the population including 7 subjects who were included in the study after randomization to receive IDEC-C2B8. | |
| Diagnosis and key inclusion criteria: Patients who met all of the following criteria 1 to 8 were included in the study:<br>1. Patients with idiopathic nephrotic syndrome.<br>2. The age at the onset (first onset) of idiopathic nephrotic syndrome is ≥ 1 year and <18 years, and the age at enrollment is ≥ 2 years.<br>3. Any of the following conditions (1) to (3) are met:<br>(1) The disease was diagnosed as frequently relapsing or steroid-dependent and is then again diagnosed as frequently relapsing or steroid-dependent after immunosuppressive therapy (e.g., cyclosporine, cyclophosphamide, mizoribine).<br>(2) The disease was diagnosed as frequently relapsing or steroid-dependent and is then again diagnosed as frequently relapsing or steroid-dependent during immunosuppressive therapy (e.g., cyclosporine, cyclophosphamide, mizoribine).<br>(3) The disease was diagnosed as steroid-resistant after the onset of idiopathic nephrotic syndrome and is then diagnosed as frequently relapsing or steroid-dependent during or after immunosuppressant therapy (cyclosporine alone or in combination with methylprednisolone).<br>4. The dates of the last 3 relapses of nephrotic syndrome before enrollment can be confirmed.<br>5. Steroid sensitivity is evident in the treatment of the last relapse before enrollment.<br>6. At least 5 CD20-positive cells are found per μL of peripheral blood.<br>7. Patients can be hospitalized for 2 days and 1 night from the day of administration to the next day for all scheduled days of study drug administration.<br>8. After full explanation of the conduct of the study, informed consent has been obtained from the patient (aged 20 years or older) or parents or legal guardian using an informed | |

| |
|---|
| consent form approved by the institutional review board (IRB). |
| Dose, mode of administration, and batch number of study drug:<br>Dose and mode of administration: Under hospitalization, study drug (IDEC-C2B8 or placebo) was administered by IV infusion at a dose of 375 mg/$m^2$ (maximum 500 mg) once a week for 4 weeks.<br>Batch number: Two lots (NS-01-1 or NS-01-2) were used.<br>Test drug (IDEC-C2B8): Each vial contains 500 mg of IDEC-C2B8 in 50 mL.<br>Control (placebo): Each vial does not contain 500 mg of IDEC-C2B8 in 50 mL. |
| Treatment duration: Since the study drug was administered once a week for 4 weeks, the duration of study treatment was 22 days. The duration of observation was 1 year from the day of first study drug administration. Assuming that the number of subjects meeting the inclusion criteria will be 20 to 30 per year, the planned duration of enrollment was 3 years and the duration of the study was 4 years. |
| Criteria for evaluation: Endpoints<br>Efficacy:<br>1. Primary endpoint: Relapse-free period<br>2. Secondary endpoints: Time to treatment failure, time to frequent relapse, time to steroid dependence, time to steroid resistance, relapse rate, steroid dose, change in total steroid dose before and after enrollment/assignment (over 365 days), etc.<br>3. Other endpoints: Blood IDEC-C2B8 concentration, time course of peripheral B-cell count, duration of peripheral B-cell depletion, and proportion of subjects with human anti-chimeric antibody (HACA) production<br>Safety: Subjective or objective symptoms, physical examination, laboratory tests, infusion reaction, and infection requiring treatment |
| Main statistical methods<br>Assuming that the proportion of subjects in remission over 6 months will be 40% in the IDEC-C2B8 group (p1) and 10% in the placebo group (p0), 30 subjects per group were required to provide 90% power to detect a significant difference at a two-sided level of 5% (one-sided level of 2.5%).<br>Analysis of efficacy: For the primary endpoint, cumulative relapse-free curve was calculated for the relapse-free interval by the Kaplan-Meier method and compared by the log-rank test in the FAS. A Cox proportional hazards model was used to estimate the hazard ratio and its 95% confidence interval (CI). A one-sided significance level of 2.5% was used. For the secondary endpoints, survival analysis was performed in the same manner as for the primary endpoint. A two-sided significance level of 5% was used. The person-years method was used for the relapse rate. Summary statistics were calculated for the steroid dose at each time point. The Wilcoxon rank sum test was performed to compare the difference in total steroid dose over 365 days before and after enrollment.<br>Other endpoints: Summary statistics were calculated for the peripheral B-cell count after study drug administration. For the B-cell depletion duration, cumulative depletion curve was calculated by the Kaplan-Meier method. The proportion of subjects with HACA production was calculated by the Kaplan-Meier |

method.
Analysis of safety: The number and incidence of AEs were calculated for each group by severity. The severity (grade) was the worst grade observed during the study. Fisher's exact test was used for between-group comparison as needed. For laboratory test values, summary statistics were calculated for each group at each time point. The incidence of abnormal laboratory test values was tabulated for each group at each time point.

Summary-Conclusions

Results of interim analysis: An interim efficacy analysis was performed in a total of 42 subjects on 11 May 2010. The median relapse-free period in the FAS, which was the primary endpoint, was 234 days (95% CI, 170 to 358 days) in the IDEC-C2B8 group and 100 days (95% CI, 76 to 156 days) in the placebo group, demonstrating the superiority of IDEC-C2B8 (log-rank test, p=0.00015). Since there was no problem with the safety results, it was decided to complete the randomization and continue the study. The efficacy and safety results were consistent with those from the final analysis.

Efficacy results: There were no marked between-group differences in patient demographics at enrollment. The median relapse-free period in the FAS was 267.0 days (95% CI, 223.0 to 374.0 days) in the IDEC-C2B8 group and 101.0 days (95% CI, 70.0 to 155.0 days) in the placebo group, showing that the relapse-free period was significantly longer in the IDEC-C2B8 group than in the placebo group (hazard ratio, 0.267; 95% CI, 0.135 to 0.528; log-rank test, p<0.001). As for the secondary endpoints, the superiority of IDEC-C2B8 was demonstrated in terms of time to treatment failure and time to steroid dependence. The relapse rate (episodes/person-year) was significantly reduced from 4.171 in the placebo group to 1.542 in the IDEC-C2B8 group. The results of steroid dose showed that IDEC-C2B8 may reduce the steroid dose. After IDEC-C2B8 administration, the B-cell count continued to decrease for approximately 3 months, but began to increase approximately 5 months later. The median B-cell depletion duration was 148.0 days (95% CI, 131.0 to 170.0 days). The proportion of subjects with HACA production was 4.2% and 14.3% on Days 169 and 365, respectively.

Safety results: Twenty of 24 subjects in the IDEC-C2B8 group and 23 of 24 subjects in the placebo group received 4 doses as specified. A total of 357 AEs occurred in 24 subjects in the IDEC-C2B8 group and 251 AEs occurred in 23 subjects in the placebo group. ADRs occurred similarly in the two groups. Major AEs in the IDEC-C2B8 group included upper respiratory tract infection in 16 subjects (66.7%), C-reactive protein increased in 11 subjects (45.8%), and lymphocyte count decreased in 9 subjects (37.5%). AEs in the placebo group included upper respiratory tract infection in 14 subjects (58.3%), lymphocyte count decreased in 10 subjects (41.7%), and alanine aminotransferase increased in 8 subjects (33.3%). Grade 4 AEs occurred only in the IDEC-C2B8 group and were neutrophil count decreased in 2 subjects (8.3%) and hyperuricaemia in 1 subject (4.2%). Grade 3 AEs included hypoproteinaemia in 5 subjects (20.8%), lymphocyte count decreased in 3 subjects (12.5%), and neutrophil count decreased in 2 subjects (8.3%) in the IDEC-C2B8 group and hypoproteinaemia in 5 subjects (20.8%), lymphocyte count decreased in 4 subjects (16.7%), and alanine aminotransferase increased in 2 subjects (8.3%) in the placebo group.

No deaths occurred. Sixteen serious AEs (SAEs) occurred in 10 subjects (41.7%) in the IDEC-C2B8 group

| |
|---|
| and 7 SAEs occurred in 6 subjects (25.0%) in the placebo group; however, no significant between-group difference was observed, and almost all events resolved. Forty-one events of infusion reaction, an AE characteristic to IDEC-C2B8, occurred in 19 subjects (79.2%) in the IDEC-C2B8 group and 26 events occurred in 13 subjects (54.2%) in the placebo group, but none were ≥ Grade 3 in severity.<br>The incidence of infection requiring treatment (episodes/person-year) was not significantly different between the IDEC-C2B8 and placebo groups (4.550 and 3.447, respectively), but was significantly lower in subjects with recovery from peripheral B-cell depletion or no depletion (3.242) than in those with depletion (8.440) (hazard ratio, 0.412; 95% CI, 0.278 to 0.609; p<0.001).<br>Conclusions: IDEC-C2B8 administered at a dose of 375 mg/m$^2$ (up to 500 mg) 4 times is effective in prolonging the relapse-free period and reducing the relapse rate in childhood-onset complicated frequently relapsing or steroid-dependent nephrotic syndrome. B cells are temporarily eliminated after IDEC-C2B8 administration, but can recover several months later. HACA production and AEs are within the expected range, and IDEC-C2B8 is well tolerated. After IDEC-C2B8 administration, however, preventive measures against infection are especially necessary during B-cell depletion. |
| Remarks:<br>Japan Medical Association Center for Clinical Trials ID: JMA-IIA00021<br>Conflict of interest: This study was funded by the Japan Medical Association Center for Clinical Trials under a "Clinical Trial Promotion Project." All decisions regarding the planning, conduct, and publication of the study were made by the investigator. The pharmaceutical company manufacturing and marketing the investigational product was involved only in providing the investigational product and safety information and measuring blood drug concentrations and blood HACA concentrations.<br>This study was conducted in accordance with current GCP, including retention of essential documents. |

Table 2-3 Overview of a pharmacokinetic study of IDEC-C2B8 in childhood-onset refractory nephrotic syndrome (RCRNS-02)

| | |
|---|---|
| Title of the study: A pharmacokinetic study of IDEC-C2B8 in childhood-onset complicated nephrotic syndrome | |
| Names of investigators: Kunihiko Aya, Kenji Ishikura, Shuichi Ito, Yoshiyuki Ohtomo, Hiroshi Kaito, Koichi Kamei, Shouri Takahashi,<br>Ryojiro Tanaka, Koichi Nakanishi, Kandai Nozu, and Kenichiro Miura (a total of 11 investigators) | |
| Study sites: A total of 9 sites, including Wakayama Medical University Hospital, National Center for Child Health and Development, Okayama University Hospital, Kobe University Hospital, and the University of Tokyo Hospital | |
| Publication (citation): Publication is in preparation at the time of completing a clinical study report. | |
| Duration of the study (years): (4 years)<br>Date of first subject's informed consent 11 November 2008<br>Date of completion of last subject's observation 26 December 2011 | Phase of development:<br>Phase 3 |

| |
|---|
| Objectives: To characterize the pharmacokinetic profile and to confirm the efficacy and safety in patients with childhood-onset complicated nephrotic syndrome administered IDEC-C2B8 at a dose of 375 mg/m$^2$ (up to 500 mg) once a week for 4 weeks. |
| Study method: A multicenter, open-label, single-arm study with central enrollment<br>The pharmacokinetic study was conducted in participants in a "multicenter, double-blind, placebo-controlled, randomized study of IDEC-C2B8 in childhood-onset complicated nephrotic syndrome (hereinafter referred to as Study RCRNS-01)" who received placebo with treatment failure or subjects who received IDEC-C2B8 by 31 December 2007. |
| Number of subjects (planned and analyzed):<br>Planned: 20 subjects<br>Analyzed: 20 subjects from Study RCRNS-01 and 3 subjects who received IDEC-C2B8 by 31 December 2007, totaling 23 subjects |
| Diagnosis and key inclusion criteria: Patients who met all of the following criteria 1 to 7 were included in the study:<br>1. Patients with idiopathic nephrotic syndrome.<br>2. The age at the onset (first onset) of idiopathic nephrotic syndrome is ≥ 1 year and <18 years, and the age at enrollment is ≥ 2 years.<br>3. Any of the following conditions (1) to (3) are met:<br>(1) Participants in Study RCRNS-01 who received placebo and experienced relapse by Week 13 (Day 85) of study treatment<br>(2) Participants in Study RCRNS-01 who received placebo and were diagnosed with frequently relapsing or steroid-dependent nephrotic syndrome between the day after Week 13 (Day 86) and Week 53 (Day 365)<br>(3) Patients with childhood-onset complicated nephrotic syndrome who received IDEC-C2B8 by 31 December 2007<br>4. Steroid sensitivity is evident in the treatment of the last relapse before enrollment.<br>5. At least 5 CD20-positive cells are found per μL of peripheral blood.<br>6. Patients can be hospitalized for 2 days and 1 night from the day of administration to the next day for all scheduled days of study drug administration.<br>7. After full explanation of the conduct of the study, informed consent has been obtained from the subject (aged 20 years or older) or custodial parent or legal guardian (hereinafter referred to as legal representative) using an informed consent form approved by the IRB. |
| Dose, mode of administration, and batch number of study drug:<br>● Dose and mode of administration: Under hospitalization, IDEC-C2B8 was administered by IV infusion at a dose of 375 mg/m$^2$ (maximum 500 mg) once a week for 4 weeks.<br>● Batch number: Two lots (NS-02-1 or NS-02-2) were used.<br>● IDEC-C2B8: Each vial contains 500 mg of IDEC-C2B8 in 50 mL. |

| |
|---|
| Treatment duration: Since the study drug was administered once a week for 4 weeks, the duration of study treatment was 22 days. The duration of observation was 1 year from the day of first study drug administration. The planned duration of enrollment was 3 years and the duration of the study was 4 years. The duration of observation for the 21st and subsequent subjects was 9 months. |
| Criteria for evaluation: Endpoints<br>Efficacy:<br>1. Primary endpoint: Pharmacokinetics of IDEC-C2B8 [1] area under the blood concentration-time curve (AUC), [2] maximum drug concentration (Cmax), [3] half-time ($T_{1/2}$), [4] clearance, [5] mean residence time (MRT), and [6] volume of distribution at steady state (Vds)<br>2. Efficacy: Relapse-free period, relapse rate, proportion of subjects with relapse, proportion of subjects with frequent relapse, proportion of subjects with steroid dependence, proportion of subjects with steroid resistance, change in total steroid dose, etc.<br>3. Other endpoints: Peripheral B-cell count, duration of peripheral B-cell depletion, and proportion of subjects with human anti-chimeric antibody (HACA) production<br>4. Safety: Subjective or objective symptoms, physical examination, laboratory tests, infusion reaction, infection requiring treatment, etc. |
| Main statistical methods<br>1. Primary endpoint: Blood IDEC-C2B8 concentrations in each subject were used to calculate the AUC and Cmax by a non-model analysis method and the other parameters by a compartment model analysis method.<br>2. Efficacy endpoints: Cumulative relapse curve was calculated for the relapse-free interval by the Kaplan-Meier method. The relapse rate was calculated by the person-years method (episodes/person-year). For the change in total steroid dose, the Wilcoxon rank sum test was performed to compare the difference in total steroid dose before and after enrollment/assignment (over 365 days).<br>3. Other endpoints: For the peripheral B-cell count, summary statistics were calculated at each time point. For the B-cell depletion duration, cumulative depletion curve was calculated by the Kaplan-Meier method. The proportion of subjects with HACA production was calculated by the Kaplan-Meier method.<br>4. Safety endpoints: The number and incidence of AEs were calculated for each group by severity. The severity (grade) was the worst grade observed during the study. Fisher's exact test was used for between-group comparison as needed. For laboratory test values, summary statistics were calculated for each group at each time point. The incidence of abnormal laboratory test values was tabulated for each group at each time point. |
| Summary-Conclusions<br>1. Pharmacokinetic results: All of 23 enrolled subjects received the study drug, and 22 of them completed 4 doses as specified. The blood IDEC-C2B8 concentration reached a maximum immediately after each study drug administration in 23 subjects analyzed. The concentration decreased after study drug |

administration, but remained constant during study drug administration. The mean AUC, Cmax, half-life K10_HL, clearance, MRT, and Vds (± standard deviation) in 22 subjects who completed 4 doses were 366000 (±110000) μg•h/mL, 421000 (±84700) ng/mL, 234 (±86.7) h, 0.00750 (±0.00236) L/h, 337 (±125) h, and 2.42 (±0.877) L, respectively. The relationships with age, height, and dose (375 mg/m$^2$ and 500 mg/body groups) were investigated. As for the dose, the trough blood drug concentration increased sequentially from the first dose to the fourth dose in both the 375 mg/m$^2$ and 500 mg/body groups, and there was no difference in B-cell depletion status, indicating that the dose was sufficient as the antibody against the antigen. In addition, parameter values calculated on a per-BSA (and per-dose) basis were also investigated. The clearance (per BSA) was similar in the 375 mg/m$^2$ and 500 mg/body groups and did not differ by body size (height) or age.

2. Efficacy results: In the full analysis set (23 subjects), the median relapse-free period was 287.0 days (95% CI, 211.0 to 344.0 days) and the relapse rate was 1.346 episodes/per person-year, similar to those observed in Study RCRNS-01. The proportion of subjects with relapse was 65.2% (95% CI, 42.7% to 83.6%), the proportion of subjects with frequent relapse was 4.3% (95% CI, 0.1% to 22.0%), the proportion of subjects with steroid dependence was 13.0% (95% CI, 2.8% to 33.6%), and the disease did not become steroid-resistant in any subject. As in Study RCRNS-01, it was shown that the steroid dose may be reduced after study drug administration. After study drug administration, the B-cell count decreased rapidly and recovered approximately 5 months later. The median B-cell depletion duration was 166.0 days (95% CI, 124.0 to 184.0 days). The proportion of subjects with HACA production was 4.3%, 9.4%, and 16.9% on Days 169, 253, and 365, respectively.
3. Safety Results: Both AEs and ADRs occurred in all subjects (23 subjects) in the safety analysis set, with a total of 280 AEs and 258 ADRs, all of which were known events. No deaths occurred. SAEs occurred in 4 subjects (7 events), but all these events resolved or were resolving. Infusion reaction occurred in 12 subjects (25 events), but all events were mild in severity with Grade 1. The incidence of infection requiring treatment was significantly lower in subjects with recovery from peripheral B-cell depletion or no depletion (2.357 episodes/person-year) than in those with depletion (6.008 episodes/person-year) (hazard ratio, 0.446; 95% CI, 0.261 to 0.764; p=0.003).

Conclusions: After IDEC-C2B8 administration at a dose of 375 mg/m$^2$ once a week for 4 weeks in childhood-onset complicated nephrotic syndrome, time course of blood IDEC-C2B8 concentration, B-cell depletion, HACA production, and safety were all within the expected range, and the efficacy such as relapse-free period and relapse rate was similar to that in Study RCRNS-01. Based on the above, it was demonstrated that IDEC-C2B8 is effective and well tolerated in the treatment of childhood-onset complicated frequently relapsing or steroid-dependent nephrotic syndrome. However, preventive measures against infection are especially necessary during B-cell depletion.

Remarks:

Japan Medical Association Center for Clinical Trials ID: JMA-IIA00021

Conflict of interest: This study was funded by the Japan Medical Association Center for Clinical Trials under a "Clinical Trial Promotion Project." All decisions regarding the planning, conduct, and publication of the

study were made by the investigator. The pharmaceutical company manufacturing and marketing the investigational product was involved only in providing the investigational product and safety information and measuring blood drug concentrations and blood HACA concentrations.
This study was conducted in accordance with current GCP, including retention of essential documents.

# 3 Objectives

In patients with childhood-onset non-complicated frequently relapsing or steroid-dependent nephrotic syndrome, the efficacy of the study drug administered at a dose of 375 mg/m$^2$ (up to 500 mg) once a week for 2 weeks will be verified, and the safety will be evaluated.
During the open-label period, the relapse-free period in the open-label observation period will be investigated. In addition, blood drug concentration and HACA will be measured to investigate the time course of blood drug concentration, HACA production status, and the relationship between the time course of peripheral B-cell count and relapse or adverse events after IDEC-C2B8 administration to patients with childhood-onset nephrotic syndrome at a dose of 375 mg/m$^2$ (up to 500 mg) twice.

## 3.1 Efficacy endpoints

### 3.1.1 Primary endpoint

(1) Relapse-free period during the blinded observation period (Days 1 to 365 of the blinded period)
It is defined as the time from the day of enrollment/assignment to the day of the first relapse after the start of study treatment during the blinded observation period (Days 1 to 365 of the blinded period). For relapse after the day of enrollment/assignment followed by confirmation of steroid sensitivity, the relapse-free period will start on the day of re-confirmation of eligibility.

### 3.1.2 Secondary endpoints

(1) Time to treatment failure
It is defined as the time from the day of enrollment/assignment to the day of determination of treatment failure during the blinded observation period (Days 1 to 365 of the blinded period). For relapse after the day of enrollment/assignment followed by confirmation of steroid sensitivity, the time to treatment failure will start on the day of re-confirmation of eligibility.

(2) Total steroid dose
It is defined as the total dose of steroid administered during the blinded observation period (Days 1 to 365 of the blinded period) after the day of confirmation of the last relapse before study treatment.

(3) Change in peripheral B-cell count during the observation phase (Days 1 to 365 of the blinded period and Days 1 to 365 of the open-label period)
  1) Peripheral B-cell count (cells/μL)
  2) Time to peripheral B-cell count recovery
  It is defined as the time from the day of confirmation of peripheral B-cell depletion during the observation

phase (Days 1 to 365 of the blinded period and Days 1 to 365 of the open-label period) to confirmation of peripheral B-cell count recovery (≥ 5 cells/μL).

### 3.2 Other endpoints

(1) Relapse-free period during the open-label period

It is defined as the time from the day of confirmation of IDEC-C2B8 treatment criteria to the day of the first relapse after the start of IDEC-C2B8 treatment.

(2) Blood IDEC-C2B8 concentration

(3) HACA production status

### 3.3 Safety endpoints

Occurrence of adverse events (AEs) and adverse drug reactions (ADRs)

## 4 Study design

### 4.1 Type of study

This is a multicenter, double-blind, placebo-controlled, randomized, parallel-group study. However, a subject who is found to have been assigned to the placebo group after early key opening may receive IDEC-C2B8 in the open-label period if he/she wishes.

### 4.2 Diagram of the study

The study period for each subject is from the day of informed consent to the last day of the blinded observation period. For subjects who enter the open-label period and receive IDEC-C2B8 after early key opening, the study period will end on the last day of the open-label observation period.

The day of first study drug administration in the blinded period is designated as Day 1 of the blinded period, and the day of first IDEC-C2B8 administration in the open-label period is designated as Day 1 of the open-label period.

Table 4-1 Diagram of the study (without early key opening)

<table>
<tr><td></td><td colspan="3">Screening phase</td><td colspan="2"></td><td colspan="4">Observation phase</td></tr>
<tr><td></td><td colspan="3"></td><td colspan="2"></td><td>Day 1 of the blinded period</td><td>Day 8 of the blinded period</td><td>••••••</td><td>Day 365 of the blinded period</td></tr>
<tr><td></td><td rowspan="3">Informed consent</td><td colspan="2">Screening<br>Confirmation of eligibility</td><td rowspan="3">Enrollment/assignment</td><td></td><td rowspan="3">First study drug administration</td><td rowspan="3">Second study drug administration</td><td rowspan="3"></td><td rowspan="3">End of the study</td></tr>
<tr><td rowspan="2">Relapse</td><td colspan="2">←→<br>35 days</td><td>←→<br>14 days</td></tr>
<tr><td>Remission</td><td>←→<br>7 days</td><td></td></tr>
</table>

Table 4-2 Diagram of the study (with early key opening followed by standard treatment)

<table>
<tr><td></td><td colspan="3">Screening phase</td><td colspan="2"></td><td colspan="9">Observation phase</td></tr>
<tr><td></td><td colspan="3"></td><td colspan="2"></td><td>Day 1 of the blinded period</td><td>Blinded period Day 8</td><td>•••</td><td></td><td></td><td></td><td>•••</td><td>Day 365 of the blinded period</td></tr>
<tr><td></td><td rowspan="3">Informed consent</td><td colspan="2">Screening<br>Confirmation of eligibility</td><td rowspan="3">Enrollment/assignment</td><td></td><td rowspan="3">First study drug administration</td><td rowspan="3">Second study drug administration</td><td rowspan="3"></td><td></td><td rowspan="3">Early key opening</td><td rowspan="3">Standard treatment</td><td rowspan="3"></td><td rowspan="3">End of the study</td></tr>
<tr><td rowspan="2">Relapse</td><td colspan="2">←→<br>35 days</td><td>←→<br>14 days</td><td rowspan="2">treatment failure<br>(1) (2) (3)</td></tr>
<tr><td>Remission</td><td>←→<br>7 days</td><td></td></tr>
</table>

Table 4-3 Diagram of the study (with early key opening followed by switching to IDEC-C2B8 treatment [open-label period])

<table>
<tr><td></td><td colspan="3">Screening phase</td><td colspan="2"></td></tr>
<tr><td></td><td colspan="3"></td><td colspan="2"></td></tr>
<tr><td></td><td rowspan="3">Informed consent</td><td colspan="2">Screening<br>Confirmation of eligibility</td><td rowspan="3">Enrollment/assignment</td><td></td></tr>
<tr><td rowspan="2">Relapse</td><td colspan="2">← 35 days →</td><td>← 14 days →</td></tr>
<tr><td>Remission</td><td>← 7 days →</td><td></td></tr>
</table>

→ Followed by the table below

Continued from table above →

| Observation phase | | | | | | | | | | | | | |
|---|---|---|---|---|---|---|---|---|---|---|---|---|---|
| Blinded period | | | | Switching period | | | | | | Open-label period | | | |
| Day 1 | Day 8 | ••• | Up to Day 168 | | ••• | | | | | Day 1 | Day 8 | ••• | Day 365 |
| First study drug administration | Second study drug administration | | | Early key opening | | | | Confirmation of IDEC-C2B8 treatment criteria | | First IDEC-C2B8 administration | Second IDEC-C2B8 administration | | End of the study |
| | | | treatment failure (1) | | | Remission | ←→ 7 days | | ←→ 14 days | | | | |

## 4.3 Target sample size

Target sample size: 40 subjects (20 subjects per group)

## 4.4 Planned study period

Planned enrollment period: From November 2018 to March 2021 (29 months)

Planned study period: From November 2018 to September 2022 (47 months)

Blinded period From November 2018 to March 2022 (41 months)

## 4.5 Discussion of study design

This is a clinical trial in patients with childhood-onset uncomplicated frequently relapsing or steroid-dependent nephrotic syndrome consisting of a confirmatory part to evaluate the efficacy and safety of IDEC-C2B8 during the blinded period and an exploratory part to investigate blood IDEC-C2B8 concentrations, change in peripheral B cells, and HACA production during the open-label period.

In Japan, IDEC-C2B8 is indicated in adults with CD20-positive B-cell non-Hodgkin's lymphoma at a dose of 375 mg/m$^2$ once a week for up to 8 weeks and approved for the treatment of Wegener's granulomatosis, microscopic polyangiitis, and chronic idiopathic thrombocytopenic purpura at a dose of 375 mg/m$^2$ once a week for 4 weeks and childhood-onset complicated frequently relapsing or steroid-dependent nephrotic syndrome at a dose of 375 mg/m$^2$ (up to 500 mg) once a week for 4 weeks. Since the planned dose of 375 mg/m$^2$ (up to 500 mg) once a week for 2 weeks in this study is within the range of the approved dosage and administration, safety information has been obtained from clinical experience. As for the efficacy, on the other hand, no clinical studies have been conducted in Japan, although there are Japanese and non-Japanese reports and data suggesting the efficacy at a dose of 375 mg/m$^2$ (up to 500 mg) once a week for 2 weeks in uncoplicated frequently relapsing or steroid-dependent nephrotic syndrome. Therefore, this multicenter, double-blind, placebo-controlled, randomized, parallel-group study is planned.

In a previous clinical trial in complicated frequently relapsing or steroid-dependent nephrotic syndrome, which was conducted in patients with highly active disease that could not be maintained in remission with existing

therapies, there was concern about early relapse. Therefore, the criteria for treatment failure were established so that when a treatment failure was determined, the double blind could be urgently unblinded and the treatment could be selected as deemed best by the inbestigators.

The present study will be conducted in patients with a diagnosis of frequently relapsing or steroid-dependent disease that is not complicated but requires immunosuppressive therapy and may therefore include patients with highly active disease that will become complicated. Since this is an placebo-controlled study and there is concern about early relapse even with IDEC-C2B8 treatment, it is considered ethically necessary to establish the treatment failure criteria (7.3.2) as in the clinical trial (RCRNS-01) for patients with complicated frequently relapsing or steroid-dependent nephrotic syndrome, and to provide patients with the presumably best treatment option later.

On the other hand, while the peripheral B-cell count will be measured in this study to investigate the relationship between the time course of peripheral B-cell count after 2 doses of IDEC-C2B8 and relapse, more accurate results will be obtained by collecting data from as many subjects as possible because of expected large individual differences. Therefore, an open-label period in which subjects with treatment failure (1) in the placebo group who wish to receive IDEC-C2B8 will receive 2 doses of IDEC-C2B8 in the same manner as in the blinded period is included so that the peripheral B-cell count can be measured in these subjects.

In addition, blood drug concentration and HACA will be measured during the open-label period to investigate the time course of blood drug concentration, HACA production, and the relationship between the time course of peripheral B-cell count and relapse or AEs after 2 doses of IDEC-C2B8 to patients with childhood-onset uncomplicated frequently relapsing or steroid-dependent nephrotic syndrome. It is desirable to collect data on blood drug concentration and HACA production after 2 doses of IDEC-C2B8 from as many subjects as possible. However, since half of the subjects will receive placebo during the blinded period and no blood drug concentration data can be obtained from them, blood drug concentration and HACA will be measured only in subjects treated with IDEC-C2B8 during the open-label period, taking into account the burden of blood sampling on placebo-treated subjects.

In summary, the purpose of administering IDEC-C2B8 to subjects with treatment failure (1) in the placebo group in this study is to collect data on the time course of peripheral B-cell count and relapse after 2 doses of IDEC-C2B8 from more subjects.

### 4.5.1 Rationale for the study population

To evaluate the efficacy of IDEC-C2B8 in patients with childhood-onset uncomplicated frequently relapsing or steroid-dependent nephrotic syndrome in this study, the study population is patients with frequently relapsing or steroid-dependent disease who have not started standard immunosuppressive therapy.

Steroid-dependent disease is generally considered to be more active frequently relapsing disease, and approximately 60% of cases of pediatric frequently relapsing disease in Japan are steroid-dependent.[21)] Frequently relapsing nephrotic syndrome and steroid-dependent nephrotic syndrome have often been handled as the same patient population in previous Japanese clinical studies in pediatric nephrotic syndrome. Based on the above, uncomplicated frequently relapsing nephrotic syndrome and steroid-dependent nephrotic syndrome are

considered to be the same patient population.

### 4.5.2 Rationale for the primary endpoint

Childhood-onset idiopathic nephrotic syndrome that has become frequently relapsing or steroid-dependent requires long-term continuous steroid use because of frequent relapse. The goal of treatment for steroid withdrawal or reduction is to maintain remission for a longer period of time. Accordingly, the relapse-free period (duration of remission) is considered to be the most important efficacy endpoint.

### 4.5.3 Rationale for the screening and observation phases

To assess eligibility, it is necessary to determine the presence or absence of HIV, HCV, HBV, or other viral infections, the presence or absence of CD20-positive cells in peripheral blood, and steroid sensitivity. Since it takes approximately 7 days to screen for viral infections and up to 28 days from the start day of treatment of relapse to determine steroid sensitivity, the screening phase is 35 days from the day of informed consent.

In recent years, multiple reports have suggested the efficacy of IDEC-C2B8 for uncomplicated frequently relapsing or steroid-dependent disease. In patients with uncomplicated steroid-dependent nephrotic syndrome overseas, 2 of 15 subjects in the rituximab group experienced recurrence within 6 months after single administration of rituximab at a dose of 375 mg/m$^2$, compared with 14 of 15 subjects in the steroid monotherapy group.[16)] In the present study as well, subjects not treated with IDEC-C2B8 (placebo group) are likely to experience relapse within 6 months. In addition, since the International Study of Kidney Disease in Children (ISKDC) defines frequent relapse as at least 4 relapses within any 12 months, the observation phase for assessment of relapse-free status is 1 year from the start day of study treatment.

### 4.5.4 Rationale for the dosage and administration of IDEC-C2B8

While the approved dosage and administration of IDEC-C2B8 for complicated frequently relapsing or steroid-dependent nephrotic syndrome is 375 mg/m$^2$ (up to 500 mg) once a week for 4 weeks, it has been reported that fewer doses of IDEC-C2B8 is effective for childhood-onset uncomplicated frequently relapsing or steroid-dependent nephrotic syndrome. According to a non-Japanese retrospective analysis of rituximab administered to patients with steroid-dependent nephrotic syndrome at a dose of 375 mg/m$^2$ once a week for 1 to 4 weeks (1 to 4 infusions), the time to the first relapse was shorter with 1 to 2 infusions (10.3 ±3.5 months, n=16) than with 3 to 4 infusions (23.3 ±18.7 months, n=11) ($p<0.05$), but the proportion of patients in long-term remission did not differ significantly between 1 to 2 infusions (8/21 patients) and 3 to 4 infusions (4/10 patients) (p=n.s.).[20)]

Also, a non-Japanese open-label study was conducted in patients with uncomplicated steroid-dependent nephrotic syndrome by administering rituximab at a dose of 375 mg/m$^2$ once a week for 2 weeks, with additional third and fourth infusions if B cells were not depleted based on the peripheral B-cell count measured before each dose. In this study, B cells were adequately suppressed after the second dose in all patients, requiring no further doses (Basu B et al., 2017.5, poster presentation at the workshop of the International Pediatric Nephrology Association and personal communication at that time).

In 12 patients with complicated steroid-dependent nephrotic syndrome who received rituximab at a single dose

of 375 mg/m$^2$ in a Japanese study, the peripheral B-cell count decreased rapidly from 238.7±314.7/mm$^3$ at baseline to 28.3±29.2/mm$^3$ 24 hours post-dose, began to recover 4 months post-dose, and almost returned to the baseline level 6 months post-dose. Of the 12 patients, 9 relapsed within 1 year (median, 129 days post-dose), with most relapses observed after recovery of B-cell count.[15)]

Data on B-cell depletion after 2 doses are also available from a Japanese study in patients with disease other than nephrosis. In a clinical trial of IDEC-C2B8 administered twice before ABO-incompatible kidney transplantation, the peripheral B-cell count was depleted 1 week after the first dose of IDEC-C2B8, remained depleted until at least Week 26, and tended to recover slightly at Weeks 38 to 50 (data submitted at the time of approval).

In addition, given that a reduced number of doses of IDEC-C2B8 may reduce the treatment burden and safety risk, we set the administration of IDEC-C2B8 at a dose of 375 mg/m$^2$ (up to 500 mg) once a week for 2 weeks in childhood-onset uncomplicated frequently relapsing or steroid-dependent nephrotic syndrome in the present study.

# 5 Subject inclusion and exclusion criteria

## 5.1 Inclusion criteria

Patients who meet all of the following criteria will be included in the study:

(1) Patients with childhood-onset idiopathic nephrotic syndrome (The diagnostic criteria for idiopathic nephrotic syndrome at initial diagnosis must be based on the criteria of the International Study of Kidney Disease in Children [ISKDC].)

(2) Patients with the onset (first onset) of idiopathic nephrotic syndrome at an age of <18 years

(3) Patients whose last relapse before enrollment has been diagnosed as frequently relapsing (at least 2 relapses within 6 months of the first remission or at least 4 relapses within any 12 months) or steroid-dependent (2 consecutive relapses within 2 weeks after dose reduction or discontinuation of prednisolone) and for whom the dates of relapses based on which the diagnosis was made can be confirmed

(4) Patients who have not been treated with immunosuppressants (e.g., cyclosporine, cyclophosphamide, mizoribine), excluding topical drugs used to treat other diseases, since the initial onset of nephrotic syndrome

(5) Patients with steroid sensitivity (remission within 4 weeks of starting daily prednisolone treatment at a dose of 60 mg/m$^2$/day) in the treatment of the last relapse before enrollment

(6) Patients with ≥ 5 CD20-positive cells* per µL of peripheral blood

* CD19-positive cells may be used at a study site where CD20-positive cells cannot be measured.

(7) Patients who can be hospitalized for 2 days and 1 night from the day of administration to the next day for the scheduled day of first study drug administration and can visit the study site throughout the observation phase

(8) Patients who (aged 20 years or older) or whose (aged less than 20 years) legal representative (patient's legal guardian) have provided written informed consent to participate in the study after full explanation of the

conduct of the study (except for patients aged 16 years or older and less than 20 years, who will also be required to provide written informed consent)

[Rationale for the inclusion criteria]

(1) and (2) To include only patients with childhood-onset idiopathic nephrotic syndrome in the study

(3) To include only patients with frequently relapsing or steroid-dependent nephrotic syndrome in the study according to the study objectives

(4) To evaluate the efficacy

(5) Because steroid sensitivity is the most important prognostic factor

(6) Because IDEC-C2B8 is a monoclonal antibody specific for CD20 antigen

(7) To administer the study drug safely and to promptly detect and manage AEs immediately after administration

(8) Based on Article 50 of the GCP Ministerial Ordinance

## 5.2 Exclusion criteria

Patients who meet any of the following criteria will not be included in the study:

(1) Patients who have been diagnosed with steroid resistance

(2) Patients with a previous diagnosis of nephritic-nephrotic syndrome such as IgA nephropathy or suspected secondary nephrotic syndrome before enrollment

(3) Patients with any of the following infections (1) to (6):

1) Patients with existing or previous (within 6 months before enrollment) serious infection requiring inpatient treatment (e.g., pneumonia, pyelonephritis)

2) Patients with existing or previous (within 6 months before enrollment) opportunistic infection (e.g., cytomegalovirus infection, systemic fungal infection, Pneumocystis infection, non-tuberculous mycobacterial infection)

3) Patients with active tuberculosis

4) Patients with previous or suspected tuberculosis infection

5) Patients with active hepatitis B or C, or hepatitis B virus carriers

6) Patients with known HIV infection

(4) Patients with existing or previous angina pectoris, cardiac failure, myocardial infarction, or severe arrhythmia (Grade 4 findings in the Common Terminology Criteria for Adverse Events v4.0 Japanese JCOG version [CTCAE v4.0-JCOG] [corresponding to the CTCAE v4.03/MedDRA v12.0 described as MedDRA/Jv20.1 in Japanese, 12 September 2017])

(5) Patients who have received live vaccine within 4 weeks before enrollment

(6) Patients with uncontrolled hypertension* despite treatment with antihypertensives at enrollment * ≥ 99th percentile in the pediatric sex- and age-specific blood pressure norms table[22] (Appendix 4)

(7) Patients with reduced renal function (estimated glomerular filtration rate** < 60 mL/min/1.73 $m^2$) at enrollment ** See the estimated glomerular filtration rate[23)-25] (Appendix 5).

(8) Patients with existing or previous autoimmune disease (e.g., Hashimoto's thyroiditis [chronic hyroiditis], Crohn's disease, ulcerative colitis, rheumatoid arthritis, idiopathic thrombocytopenic purpura, systemic lupus erythematosus, autoimmune hemolytic anemia, sclerosis) or vascular purpura

(9) Patients with existing or previous malignancy (including "suspected" malignancy with no definitive diagnosis)

(10) Patients who have undergone organ transplantation (excluding corneal or hair transplantation)

(11) Patients with a history of drug allergy to methylprednisolone, acetaminophen, or d-chlorpheniramine maleate

(12) Patients who have at least one of the following laboratory parameters at enrollment in the measurement after informed consent and within 14 days before enrollment for 1) to 5) and after informed consent and within 35 days before enrollment for 6) to 7):

1) White blood cells < 3,000/μL
2) Neutrophils < 1,500/μL
3) Platelets < 50,000/μL
4) AST (GOT) ≥ 2.5 times the upper limit of normal in the pediatric age-specific liver escape enzyme (GOT) norms table[26) ](Appendix 6)
5) ALT (GPT) ≥ 2.5 times the upper limit of normal in the pediatric age-specific liver escape enzyme (GPT) norms table[26)] (Appendix 7)
6) Positive for HBs antigen, HBs antibody, HBc antibody, or HCV antibody,
excluding patients positive only for HBs antibody who have been vaccinated against HB and are negative for HBV-DNA (less than detection sensitivity) at enrollment
7) Positive for HIV antibody

(13) Patients who have used any monoclonal antibody (mouse, rat, chimeric, or human), including rituximab

(14) Patients who have received another investigational product within 6 months before enrollment or who are planning to participate in another clinical trial during participation in the study

(15) Patients of childbearing potential who do not agree to use contraception during the observation phase (confirmation by serum or urine HCG at screening is mandatory)

(16) Females who are pregnant, who may be pregnant, or who are breastfeeding

(17) Patients who, in the opinion of the investigators, are ineligible for participation in the study

[Rationale for the exclusion criteria]

(1) Because this is not a target disease

(2) To exclude secondary nephrotic syndrome

(3) to (12) Because it is not desirable to include these patients in the study for safety reasons

(13) and (14) Because the safety or efficacy evaluation in the study may be affected

(15) and (16) Since IDEC-C2B8 is homogeneous to immunoglobulins, which are known to cross the placenta and enter the fetus, there is concern that IDEC-C2B8 administered to a mother may affect the fetus.

(17) To exclude patients who are not eligible for participation in the study

# 6 Investigational product

The investigational product in the blinded period consists of the test drug and the control, which are indistinguishable from each other in appearance.

The investigational product (IDEC-C2B8) in the open-label period following early key opening is the same as the test drug in the blinded period, except for packaging and labeling.

Zenyaku Kogyo Company, Limited will request Genentech (California, US) to manufacture the investigational product and will provide it to the study sites.

## 6.1 Overview of the test drug (IDEC-C2B8, active)

(1) Development code and name

Development code: IDEC-C2B8

Non-proprietary name:

[JAN] (Japanese name) Rituximab (genetic recombination)

(English) Rituximab (genetic recombination)

[INN] Rituximab

(2) Nature: A chimeric anti-CD20 monoclonal antibody consisting of the constant region of a human immunoglobulin (IgG1κ) and the variable region of a mouse anti-CD20 antibody (molecular weight, 144,510 Da)

(3) Special note: The test drug IDEC-C2B8 has been approved for the treatment of B-cell non-Hodgkin's lymphoma and complicated nephrotic syndrome (frequently relapsing or steroid-dependent) and is already on the market. The commercial product is designated as a biological product.

(4) Dosage form: Injection (containing 500 mg of rituximab (genetic recombination) in 50 mL per vial)

(5) Excipients: Polysorbate 80, sodium chloride, sodium citrate hydrate, anhydrous citric acid, and pH adjuster

(6) Description: A colorless to light yellow clear or slightly white turbid liquid with a pH value of 6.5±0.3 and an osmotic pressure ratio of approximately 1 relative to physiological saline

(7) Storage conditions: Store in a cool place (2 to 8°C), avoiding freezing

## 6.2 Overview of the control (IDEC-C2B8 placebo)

(1) Dosage form: Injection (placebo vial that does not contain rituximab [genetic recombination] in 50 mL per vial and is indistinguishable from the test drug in appearance)

(2) Excipients: Polysorbate 80, sodium chloride, sodium citrate hydrate, anhydrous citric acid, and pH adjuster

(3) Description: A colorless to light yellow clear or slightly white turbid liquid

(4) Storage conditions: Store in a cool place (2 to 8°C), avoiding freezing

## 6.3 Packaging and labeling of the investigational product

(1) Packaging: One dose (1 vial) for a single subject will be individually boxed and 2 doses (2 individual boxes) will be packed in a large box.

(2) Labeling: Individual boxes and vials will be labeled with the following information: for clinical trial use,

drug number, content of study drug, storage method, expiration date, name, affiliation/job title, and address of the coordinating investigator (the chairperson of the clinical trial steering committee in this study), serial number, etc. A sample labeling is shown in the figure below.

1) Blinded period

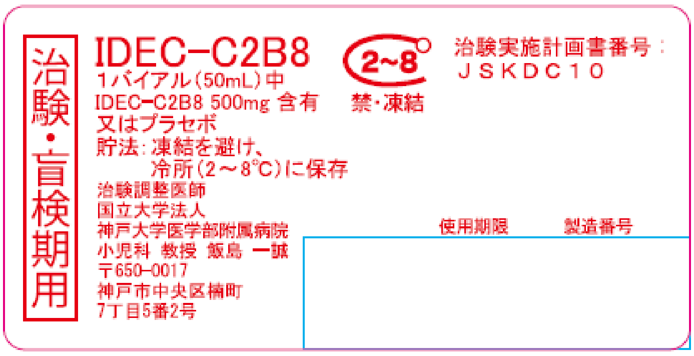


2) Open-label period

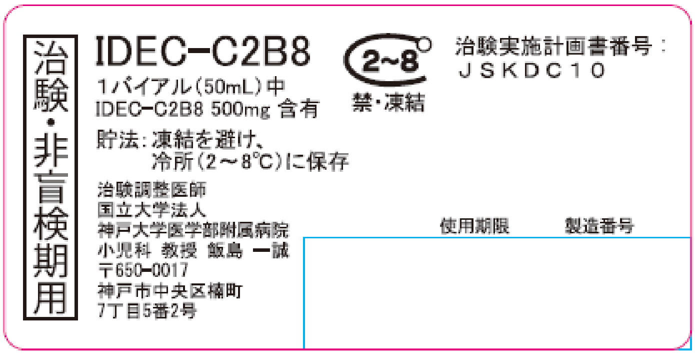


## 6.4 Handling of the investigational product

The investigational product will be delivered, stored, managed, and collected according to the "Procedures for Provision/Receipt, Management, Handling, and Return of Investigational Products."

# 7 Investigational plan

## 7.1 Definition of study period

The study period for each subject is from the day of informed consent to the last day of the blinded observation period. For subjects who enter the open-label period and receive IDEC-C2B8, the study period will end on the last day of the open-label observation period.

(1) Screening phase

Informed consent will be obtained after the last relapse and the patient will be screened. Subjects will be enrolled within 35 days after informed consent and within 7 days from the day of confirmation of remission.

(2) Blinded observation period

The blinded observation period is from the day of first study drug administration (Day 1 of the blinded period) to Week 53 (Day 365 of the blinded period).

The study treatment period is as follows and 8 days long if treatment is completed:

Start day of treatment: Day of first study drug administration (within 14 days of enrollment)

End day of treatment: Day 8 of the blinded period if study treatment is completed as specified or day of last study drug administration if study treatment is discontinued

* For subjects who receive standard treatment after early key opening, the observation phase will end on Day 365 of the blinded period.

* For subjects who enter the open-label period after early key opening to receive IDEC-C2B8, the blinded observation period will end on the day before early key opening.

(3) Switching period

The "switching period" is defined as the period from the day of early key opening to the day before first IDEC-C2B8 administration in the open-label period.

The treatment criteria in the open-label period will be confirmed within 7 days from the day of confirmation of remission after relapse.

(4) Open-label observation period

The open-label observation period is from the day of first IDEC-C2B8 administration (Day 1 of the open-label period) to Week 53 (Day 365 of the open-label period).

The IDEC-C2B8 treatment period is as follows and 8-day long if treatment is completed:

Start day of treatment: Day of first IDEC-C2B8 administration (within 14 days from the day of confirmation of treatment criteria in the open-label period)

End day of treatment: Day 8 of the open-label period if IDEC-C2B8 treatment is completed as specified or day of last IDEC-C2B8 administration if IDEC-C2B8 treatment is discontinued

## 7.2 Screening phase (from informed consent to enrollment)

If nephrotic syndrome recurs in a candidate subject of the study, the investigator will explain the details of the study and obtain written consent to participate in the study. After informed consent is obtained, the patient will be screened to confirm subject eligibility. After the eligibility is confirmed, the patient will be immediately enrolled.

### 7.2.1 Explanation and informed consent

During the informed consent process, the investigator will hand over the information sheet for the study approved by the IRB and fully explain the contents to obtain voluntary written informed consent to participate in the study (18.2). Prior to informed consent, each candidate subject will be given sufficient time and opportunity to discuss and ask questions about the contents of the information sheet and other study-related issues, and these questions will be fully answered.

(1) If a patient is 20 years old or older, an explanation will be given directly to the patient to obtain written informed consent. If a patient is less than 20 years old, an explanation will be given to his/her legal representative (custodial parent or legal guardian) to obtain written informed consent.

(2) The information sheet (for adults) and informed consent form will be prepared so that individuals aged approximately 16 years can understand, and two assent forms will be prepared so that individuals aged approximately 7 to 12 years and those aged approximately 12 to 15 years can understand, respectively.

(3) For patients aged 20 years or older and legal representatives, the information sheet (for adults) will be used for explanation. For patients aged less than 20 years, one of three information sheets (one for adults and two for children) will be selected and used for explanation according to age and comprehension.

(4) Patients aged 16 years or older and less than 20 years will also be required to provide written informed consent.

(5) Patients aged 7 years or older and less than 16 years will be required to fully understand an assent form and then sign and date an acknowledgment form in order to obtain assent. Assent may be obtained orally from patients aged 7 years or older and less than 12 years, and this oral assent will be noted on the informed consent form.

(6) The informed consent form will be sealed with the name or signed and dated by the investigator who gives the explanation, the study coordinator (if a supplementary explanation is given), and the patient who provides consent (aged 20 years or older) or the legal representative. In addition, the relationship between the subject and his/her legal representative will be recorded.

(7) The investigator will give a copy of the informed consent form to the patient (aged 20 years or older) or legal representative, and the investigator will retain the original.

(8) If informed consent is obtained, the date of informed consent will be recorded in the medical chart, etc. If a subject (aged 20 years or older) or legal representative makes a request for withdrawal from the study during the screening phase or study period, the time and reason for withdrawal will be recorded in the medical chart, etc.

### 7.2.2 Screening and confirmation of eligibility

(1) After obtaining informed consent, the investigator will screen the patient to confirm eligibility. Any hematology, serum chemistry, or virology data after the last relapse (hematology or serum chemistry within 14 days before enrollment and virology within 35 days before enrollment) may be used for screening even if the data are collected before informed consent, and it is not necessary to repeat the test. If laboratory data before informed consent are used, written consent must be obtained from the subject or his/her legal representative after explanation with the information sheet describing the use of the data.
(2) To determine steroid sensitivity, the investigator will ask the subject and legal representative to start completing the patient diary from the day after the day of informed consent.
(3) The patient screened and confirmed to be eligible will be enrolled within 7 days from the day of confirmation of remission.

### 7.2.3 Notes for completion and operation of patient diary

The patient diary will be completed by the subject or guardian (legal representative) from the day after the day of informed consent to the end of the observation phase. Any entry made by a minor subject should be checked by his/her guardian (legal representative) whenever possible. The investigator will explain the following to the subject or guardian (legal representative):

(1) Write in block print with a black or blue ballpoint pen or ink.
(2) Make any correction with strikethrough or the like so that the entry before correction can be read.
(3) If the morning urine protein test is not performed, do not leave the "date of test" field blank but draw a diagonal line to distinguish it from a missing entry. If there is a reason, describe the details in the memo field.
(4) Record the morning urine protein result in the patient diary every day throughout the study period and bring it on the day of medical examination.
(5) The investigator or study coordinator will fill in the necessary information (date of visit and expected date of next visit) in the physician's entry field of the patient diary.

At the medical examination, the investigator or study coordinator will ask the subject and guardian (legal representative) about the morning urine test status between visits and check the patient diary.

### 7.2.4 Determination of steroid sensitivity

Steroid sensitivity is defined as remission within 4 weeks of starting treatment (negative morning urine protein dipstick for 3 consecutive days).
The investigator will determine steroid sensitivity within 4 weeks of starting prednisolone treatment for the last relapse. The qualitative morning urine protein test result between visits will be checked in the patient diary at the medical examination, and urinalysis (11.2) will be performed at the study site to confirm that the subject is

negative for morning urine protein for 3 consecutive days. At the medical examination, the subject and guardian (legal representative) will be asked about the morning urine protein test status and the patient diary will be checked. The following are also considered negative for morning urine protein:

(1) ± in qualitative morning urine protein test at the study site
(2) < 30 mg/dL in quantitative morning urine protein test at the study site
(3) Morning urine protein/creatinine ratio < 0.2 at the study site if ± is observed in qualitative morning urine protein test performed by the subject

### 7.2.5 Enrollment

Subjects will be enrolled within 7 days from the day of confirmation of remission after relapse.
After obtaining informed consent from a patient, the investigator will follow the following procedures until the start of study treatment:

(1) After obtaining informed consent from a patient, the investigator will screen him/her to determine eligibility.
(2) The investigator will access the electronic data capture (EDC) using a pre-issued user ID and password to enter the information required for subject enrollment.
(3) If it is determined via the EDC that the patient is eligible, the investigator will confirm the enrollment number and drug number described in the "Enrollment Confirmation Form" and retain the "Enrollment Confirmation Form." The enrollment number and drug number allocated to the subject will also be communicated to the investigational product provider.
(4) The investigator will fill out the "Drug Supply Request Form" and send it to the investigational product provider by e-mail.

    <Investigational product provider>
    GCP Office, Prescription Products Development Department, Zenyaku Kogyo Company, Limited
    5-6-15 Otsuka, Bunkyo-ku, Tokyo 112-8650, Japan
    Phone: 03-3946-1113
    E-mail address: GCPjimukyoku@mail.zenyaku.co.jp
    Business hours: 9:00 to 17:00 on weekdays

(5) The investigational product provider will confirm the consistency between the "Enrollment Confirmation Form" and the "Drug Supply Request Form" and deliver the investigational product to the study site.

### 7.2.6 Assignment

Subjects will be randomly assigned to active treatment or placebo in an approximately 1:1 ratio in order of enrollment by the stratified permutation block method with "frequent relapse/steroid dependence" as the randomization factor. The randomization factor is "steroid dependence" for subjects who meet both "frequent relapse" and "steroid dependence."
The algorithm for assignment will be determined by the statistical analysis manager.

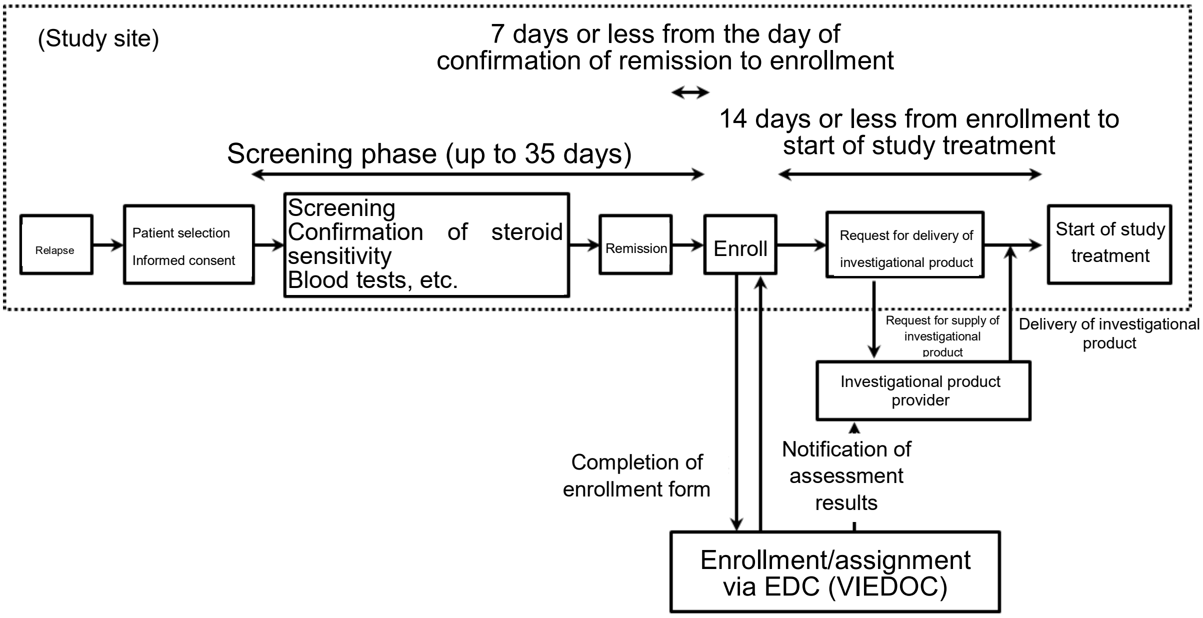


Figure 7-1 Flow to study treatment

### 7.2.7 Exclusion after enrollment and re-enrollment

A subject who fails to receive the first dose of study drug within 14 days from the day of enrollment/assignment at the discretion of the investigator is not qualified to participate in the study and will therefore be excluded from the study after enrollment.

In the event of relapse after the day of enrollment/assignment, the first dose of study drug may be administered beyond 14 days from the day of enrollment/assignment if the disease is steroid-sensitive (remission within 4 weeks of starting daily prednisolone treatment at a dose of 60 mg/m$^2$/day). In such a case, the eligibility must be reconfirmed.

For re-enrollment, the following procedures will be followed:

(1) After obtaining informed consent, the investigator will screen the patient to confirm eligibility (7.2.2).

 1) Any hematology or serum chemistry data after the last relapse before re-enrollment (within 14 days before re-enrollment) may be used for screening, and it is not necessary to repeat the test.

 2) If virology data within 85 days before re-enrollment are available, it is not necessary to repeat the test.

(2) The patient screened and confirmed to be eligible will be enrolled promptly (7.2.5).

## 7.3 Observation phase

The observation phase for each subject is from the day of first study drug administration (Day 1 of the blinded period) to Week 53 (Day 365 of the blinded period). For subjects who enter the open-label period and receive IDEC-C2B8, the observation phase will end on the last day of the open-label observation period (Day 365 of the open-label period).

The investigator will administer the first dose of study drug within 14 days from the day of enrollment/assignment and perform observations, tests, and investigations according to the study schedule,

whether study treatment is completed or discontinued (11.1).

### 7.3.1 Relapse of nephrotic syndrome

In this study, relapse and date of relapse are defined as described below.

The investigator will assess relapse at the medical examination based on the results of quantitative morning urine protein test recorded in the patient diary and the results of quantitative morning urine protein test performed at the study site (≥ 100 mg/dL in quantitative morning urine protein test at the study site corresponds to ≥ 2+ in morning urine protein dipstick). Relapse that meets the definition of relapse in the study will be recorded in the medical record and the recurrence status will be entered into the EDC. In addition, the date of relapse will be confirmed in the patient diary and recorded in the medical record.

| | |
|---|---|
| Relapse | Any of the following conditions requiring prednisolone treatment:<br>[1] Morning urine protein dipstick ≥ 3+ (or ≥ 300 mg/dL in quantitative urine protein test) for 3 consecutive days<br>[2] Urine protein dipstick ≥ 2+ (or ≥ 100 mg/dL in quantitative urine protein test) and serum albumin ≤ 3.0 g/dL |
| Date of relapse | The first date of morning urine protein dipstick ≥ 3+ (or ≥ 300 mg/dL in quantitative urine protein test) for 3 consecutive days or date of urine protein dipstick ≥ 2+ (or ≥ 100 mg/dL in quantitative urine protein test) and serum albumin ≤ 3.0 g/dL |
| Frequent relapse | At least 2 relapses within 6 months of the first remission or at least 4 relapses within any 12-month period |
| Date of frequent relapse | Date of relapse meeting the definition of frequent relapse (date of the second relapse for at least 2 relapses within 6 months of the first remission or date of the fourth relapse for at least 4 relapses within any 12 months) |
| Steroid dependence | Two consecutive relapses within 2 weeks after dose reduction or discontinuation of prednisolone |
| Date of steroid dependence | Date of the second relapse meeting the definition of steroid dependence |
| Steroid resistance | Failure to achieve remission despite at least 4 weeks of daily prednisolone treatment at a dose of 60 $mg/m^2$/day |
| Date of steroid resistance | Date when failure to achieve remission despite 4 weeks of daily prednisolone treatment at a dose of 60 $mg/m^2$/day is confirmed at the study site |

### 7.3.2 Definition of treatment failure

In this study, treatment failure is defined as one of the following:

Treatment failure (1): Relapse between the day of first study drug administration (Day 1 of the blinded period) and the day before Week 25 (Day 168 of the blinded period)

Treatment failure (2): Two relapses between Week 25 (Day 169 of the blinded period) and Week 53 (Day 365 of the blinded period)

Treatment failure (3): Steroid resistance diagnosed during the blinded observation period (Days 1 to 365 of the blinded period)

Treatment failure described above will be determined during the blinded observation period. Any event meeting the above definition after early key opening will not be determined as treatment failure.

### 7.3.3 Handling of treatment failure

When determined as treatment failure, the subject will be assessed for the efficacy and safety. Then, the emergency code will be open according to the procedures for code storage and opening, and the following rules will be followed (8.3.2):

(1) A subject with treatment failure (1) who has been assigned to the placebo group will be switched to one of the following after the physician decides whether or not to continue his/her participation in the study and asks him/her about his/her desire for the next treatment:

1) Standard treatment

Treatment considered best by the investigator (standard treatment), such as the initiation of immunosuppressants, in accordance with the Clinical Practice Guideline will be provided. Observations, investigations, and tests will be continued until the end of the blinded observation period (Week 53, Day 365 of the blinded period) according to the study schedule in Table 11-1. It is unnecessary to measure peripheral B cells.

2) Open-label period (IDEC-C2B8 treatment)

Prednisolone will be administered for relapse (9.4) to confirm remission. The treatment criteria for IDEC-C2B8 (open-label period) treatment will be confirmed within 7 days from the day of confirmation of remission after relapse to start treatment in the open-label period (IDEC-C2B8). If virology is performed within 85 days before confirmation of the IDEC-C2B8 treatment criteria or the peripheral B-cell count is measured within 35 days before the confirmation of the IDEC-C2B8 treatment criteria, it is not necessary to repeat it. With the day of first IDEC-C2B8 administration as Day 1 of the open-label period, observations, investigations, and tests will be continued until Week 53 (Day 365 of the open-label period) according to the study schedule in Table 11-2, and peripheral B cells, HACA, and blood drug concentration will also be measured.

(2) A subject with treatment failure (1) who has been assigned to the IDEC-C2B8 treatment group will receive treatment considered best by the investigator (standard treatment), such as the initiation of immunosuppressants, in accordance with the Clinical Practice Guideline. Observations, investigations, and tests will be continued until the end of the blinded observation period (Week 53, Day 365 of the blinded period) according to the study schedule in Table 11-1.

(3) A subject with treatment failure (2) or (3) who has been assigned to the placebo group will receive treatment considered best by the investigator (standard treatment), such as the initiation of immunosuppressants, in accordance with the Clinical Practice Guideline. Observations, investigations, and tests will be continued until the end of the blinded observation period (Week 53, Day 365 of the blinded period) according to the study schedule in Table 11-1. Thereafter, it is unnecessary to measure peripheral B cells.

(4) A subject with treatment failure (2) or (3) who has been assigned to the IDEC-C2B8 treatment group will receive treatment considered best by the investigator (standard treatment), such as the initiation of immunosuppressants, in accordance with the Clinical Practice Guideline. Observations, investigations, and tests will be continued until the end of the blinded observation period (Week 53, Day 365 of the blinded

period) according to the study schedule in Table 11-1.

# 8 Blinding and key opening

## 8.1 Blinding

### 8.1.1 Type and level of blinding

This is a placebo-controlled, double-blind, parallel-group trial. The EDC will be used to enroll and assign subjects. Only the drug number corresponding to the assigned treatment group will be output to the investigator. Blinding will be maintained by not accessing allocation codes corresponding to drug numbers, except when the randomization manager enters allocation codes into the assignment program.

### 8.1.2 Procedures to ensure blinding during the blinded period

(1) Confirmation of indistinguishability of investigational product supplies

Prior to blinding of study drug, the randomization manager will confirm the indistinguishability of the test drug and the control using the formulations for randomization.

(2) Maintenance of blinding

To maintain blinding, the laboratory test facility will seal peripheral B-cell count results in the blinded period until key opening at the end of the entire study and will not inform the study site or the investigator of the results.

The investigator will not measure the peripheral B-cell count at the study site during the subject's blinded observation period, and if measured, the subject will be handled as non-evaluable. If any of the conditions for emergency allocation code opening (8.3.1) is met, the peripheral B-cell count may be measured at the study site only after opening of the emergency allocation code.

If measured at the study site for the purpose of diagnosis or discussion on treatment after the end of the subject's blinded observation period, the investigator will not inform anyone other than the subject and legal representative of the results.

(3) Creation and storage of allocation codes

The randomization manager will create "allocation codes" based on the assignment table creation specification and seal and store them until all data and assessments are finalized after the end of the entire study.

(4) Creation and storage of emergency allocation codes

The randomization manager will create "emergency allocation codes" so that an investigational product can be identified in the event of treatment failure or medical emergency. "Emergency allocation codes" will be sealed and stored by the randomization manager.

(5) Storage of emergency allocation codes

After confirming the assigned drug group, the investigator will seal and store the emergency allocation code according to the "Procedures for Emergency Allocation Code Opening" (will not describe allocation results in the medical record).

## 8.2 Key opening for the entire study

To maintain blinding, "allocation codes" and "emergency allocation codes" will be open after all data and assessments are locked after the end of the entire study.

## 8.3 Emergency allocation code opening

### 8.3.1 Conditions for emergency allocation code opening

If any of the following is met, the investigator may request opening of the subject's emergency allocation code:

(1) An SAE that results in death or is life-threatening occurs.

(2) Any other SAE that appears to make this information essential for discussion of treatment occurs.

(3) The subject is determined as treatment failure (7.3.2).

If it is deemed necessary to report an AE to Japanese or foreign regulatory authorities, the investigational product provider may request opening of the subject's emergency allocation code.

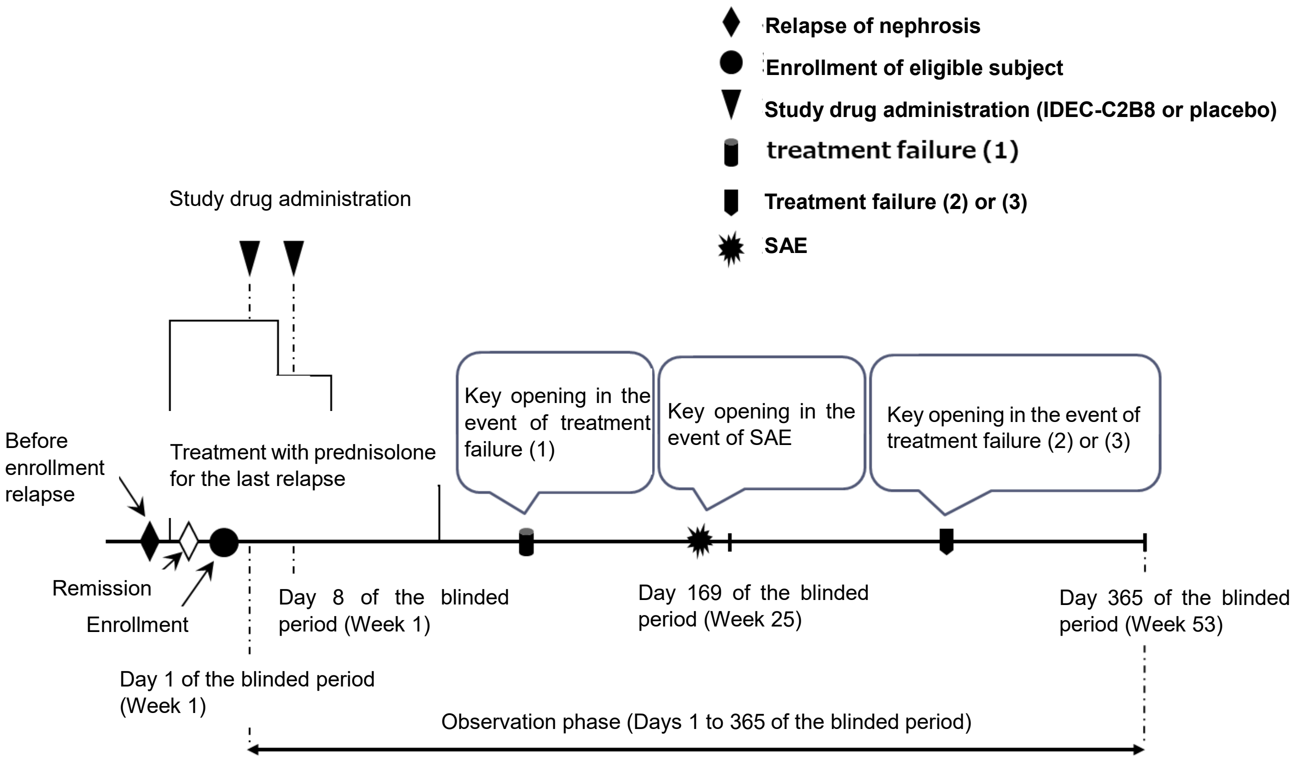


Figure 8-1 Conditions for emergency allocation code opening

### 8.3.2 Procedure for emergency allocation code opening

(1) If any of the conditions for emergency opening appears to be met, the investigator will describe the reason and drug number in the "Request for emergency allocation code opening" and send it to the study coordinating office by e-mail.

(2) The investigator will evaluate the efficacy (presence or absence of recurrence and date of relapse) and safety

(presence or absence and type of AE and causal relationship) for the subject and promptly enter the results into the EDC.

(3) Upon receipt of a request for emergency allocation code opening, the study coordinating office will cooperate with the data management manager to confirm and lock the efficacy and safety evaluation data of the subject (including the electronic signature in the EDC) and will promptly notify the study coordinating committee.

(4) If emergency allocation code opening is deemed appropriate after discussion with the efficacy and safety evaluation committee as needed, the study coordinating committee will request the randomization manager to break the emergency allocation code for the subject.

(5) The randomization manager will mail an unopen emergency allocation code to the investigator at the study site.

(6) In the event of opening, the randomization manager will record the circumstances leading to opening and the address to which the emergency allocation code is sent.

(7) The investigator will open the emergency allocation code. The investigator is recommended to provide appropriate medical care to the subject after opening the emergency allocation code to confirm the assigned drug group.

(8) The investigator will record the circumstances leading to emergency allocation code opening and the extent of notification of code opening results in the medical record (code opening results will not be recorded in the medical record).

(9) The investigator will seal and store the emergency allocation code according to the "Procedures for Emergency Allocation Code Opening."

# 9 Treatment plan

Study treatment in this study is defined as treatment with prednisolone for the last relapse, study drug administration (including the open-label period), and treatment with prednisolone for relapse during the study period (from the day of informed consent to the end of the blinded/open-label observation period).

Subjects will not receive new treatment until relapse during the study period. Rituximab will not be administered on an unscheduled basis using leftover study drug or commercially available drug (10.1.2).

## 9.1 Treatment with prednisolone for the last relapse before enrollment

Treatment with prednisolone for the last relapse before enrollment will be completed by tapering the dose from Regimen (1) to Regimen (4) as described below according to the relapse treatment method of the ISKDC. The duration of treatment with prednisolone before enrollment is included in the duration of Regimen (1).

Since the study population is subjects with frequently relapsing/steroid-dependent nephrotic syndrome, which is presumed to be highly active, the duration of treatment with Regimen (1) is as follows:

1) "4 weeks" if the patient is receiving prednisolone at the time of the last relapse before enrollment
2) "4 weeks" is recommended if the patient is not receiving prednisolone at the time of the last relapse

before enrollment, but "until negative urine protein dipstick for 3 days" is acceptable.

The dose of prednisolone will be calculated based on the body surface area* (in increments of 5 mg, that is, the ones place is 0 for ≥ 0 and < 2.5, 5 for ≥ 2.5 and < 7.5, and 0 for ≥ 7.5 with 1 being added to the tens place).

* The body surface area will be calculated from height and height-based standard weight (Appendix 1) using the Du Bois formula.

Body surface area (BSA) ($m^2$) = weight (kg)$^{0.425}$ × height (cm)$^{0.725}$ × 0.007184 (Du Bois)

The maximum dose in Regimen (1) is 60 mg/day.

The dose of prednisolone in Regimen (2) and subsequent regimens will be determined based on the height at enrollment according to the height-specific prednisolone dose table (Appendix 2).

In any of the following, the dose of prednisolone will be reduced as appropriate if deemed necessary by a treating physician:

1) History of adverse reaction to prednisolone
2) Adverse reaction to prednisolone during treatment

<When the maximum dose is 60 mg/day for 60 mg/$m^2$/day>

(1) 60 mg/$m^2$/day (up to 60 mg/day) in 3 divided doses every day (or in 2 divided doses if deemed necessary by the investigator)
(2) 60 mg/$m^2$ (up to 60 mg/day) once every other morning for 14 days
(3) 30 mg/$m^2$ (up to 30 mg) once every other morning for 14 days
(4) 15 mg/$m^2$ (up to 15 mg) once every other morning for 14 days and discontinued

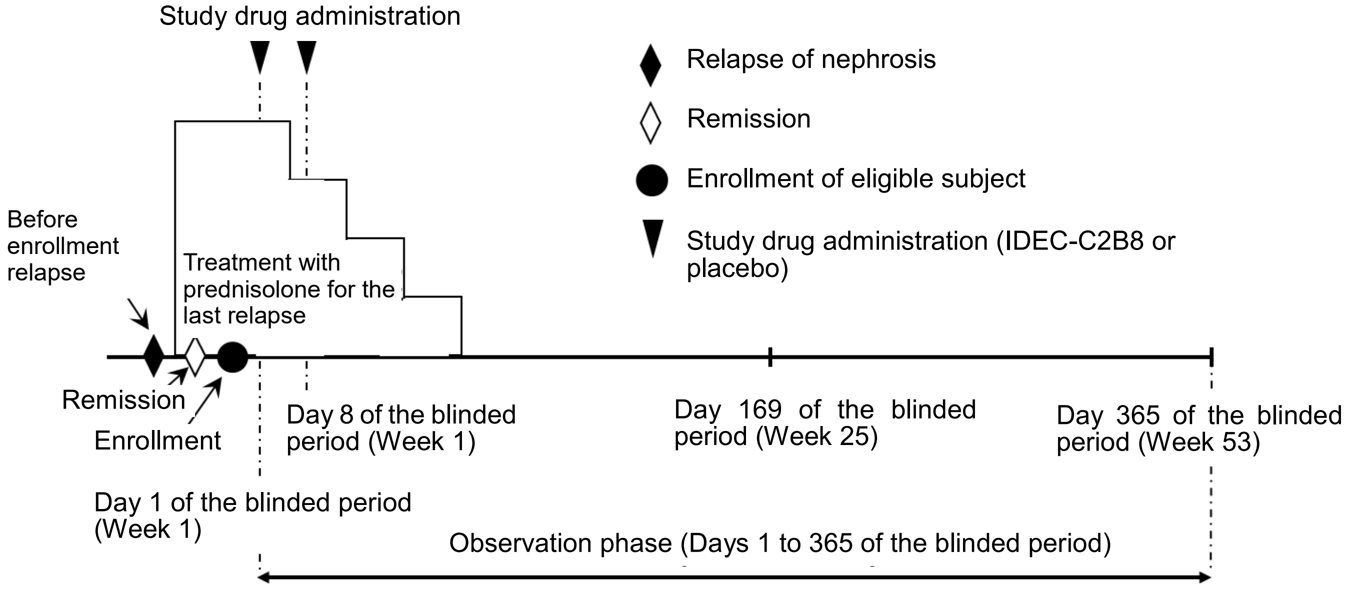


Figure 9-1 Treatment plan (blinded period)

## 9.2 Study drug administration (blinded period)

The investigator will administer the first dose of study drug (for the blinded period) within 14 days of enrollment (the day of first study drug administration is designated as Week 1 [Day 1 of the blinded period]).

The study drug (for the blinded period) in each group to which subjects are assigned will be administered at a dose of 375 mg/m$^2$ (up to 500 mg) once a week for 2 weeks (Days 1 and 8 of the blinded period).

### 9.2.1 Method for preparing study drug

(1) The study drug will be diluted 10-fold with physiological saline or 5% glucose for injection to a final concentration of 1 mg/mL immediately before administration, and IV infusion will be completed within 24 hours of preparation.

(2) Do not mix the diluted solution with other drugs. In addition, do not vigorously stir or foam the solution during dilution.

### 9.2.2 Method for administering study drug

The first dose of study drug will be administered on an inpatient basis (at least 2 days and 1 night including the day of administration and the next day), not on an outpatient basis. If infusion reaction does not occur or is mild (≤ Grade 1 except for pyrexia, which is ≤ Grade 2) after the first dose, the second dose may be administered on an outpatient basis. The day of study drug administration will be changed according to 9.3.2 if applicable.

(1) Dose of study drug

The dose of study drug in the blinded period will be determined based on the height at enrollment according to the height-specific dose table (Appendix 3).

The specified dose per IV infusion cannot be changed.

| |
|---|
| Dosage and administration of study drug<br>375 mg/m$^2$ (up to 500 mg) once a week for 2 weeks (Days 1 and 8 of the blinded period) |

(2) Pretreatment

Pretreatment with an oral antipyretic analgesic, an oral antihistamine, and intravenous methylprednisolone (9.2.3) will be given approximately 30 minutes before each study drug administration to prevent infusion reaction.

1) Acetaminophen will be administered orally.
2) D-chlorpheniramine maleate will be administered orally.
3) Methylprednisolone sodium succinate will be administered by IV infusion.

(3) Standard IV infusion rate of study drug (Figure 9-2 and Figure 9-3)

The new regimen is recommended in this study, but the conventional regimen may be used. In both regimens, the study drug may be administered at a slow rate at the discretion of the Investigator, but infusion should be completed within 24 hours of preparation.

1) New regimen

<First dose>

The study drug will be initially administered at an infusion rate of 50 mg/h for the first 30 minutes, which will then be increased by 50 mg/h every 30 minutes (up to 300 mg/h) while monitoring the subject's condition.

<Second dose>

If infusion reaction is mild (≤ Grade 1 except for pyrexia, which is ≤ Grade 2) after the first dose, the study drug will be initially administered at an infusion rate of 100 mg/h for the first 30 minutes, which will then be increased by 100 mg/h every 30 minutes (up to 300 mg/h) while monitoring the subject's condition.

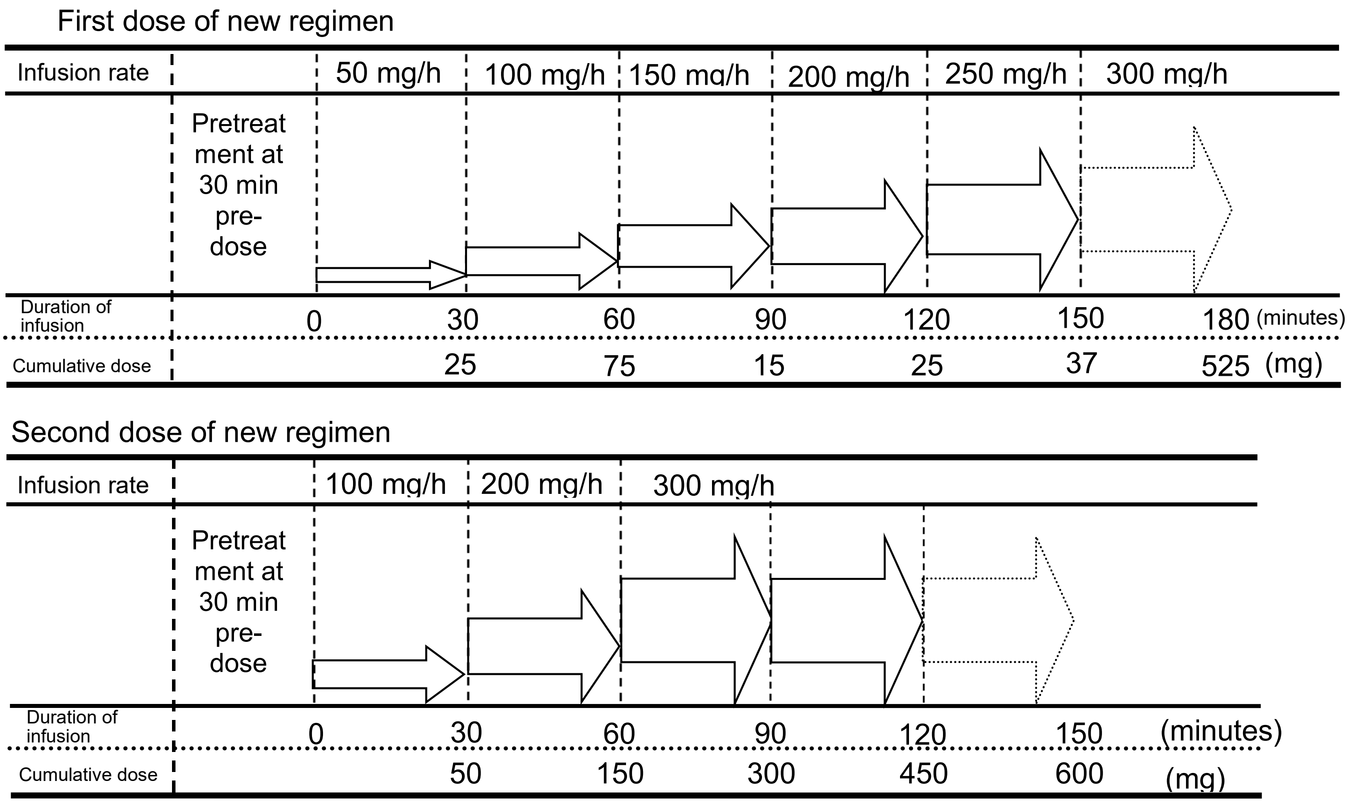


Figure 9-2 New regimen

2) Conventional regimen

<First dose>

The study drug will be initially administered at an infusion rate of 25 mg/h for the first 1 hour, which will then be increased to 100 mg/h for the next 1 hour and 200 mg/h thereafter while monitoring the subject's condition.

<Second dose*>

If infusion reaction is mild (≤ Grade 1 except for pyrexia, which is ≤ Grade 2) after the first dose, the study drug will be initially administered at an infusion rate of 100 mg/h for the first 1 hour, which will then be increased to 200 mg/h while monitoring the subject's condition.

* In studies in B-cell lymphoma, the incidence of infusion reaction following IDEC-C2B8 administration was highest after the first dose, when B cells in peripheral blood were rapidly destroyed, and then decreased to less than half after the second and subsequent doses.

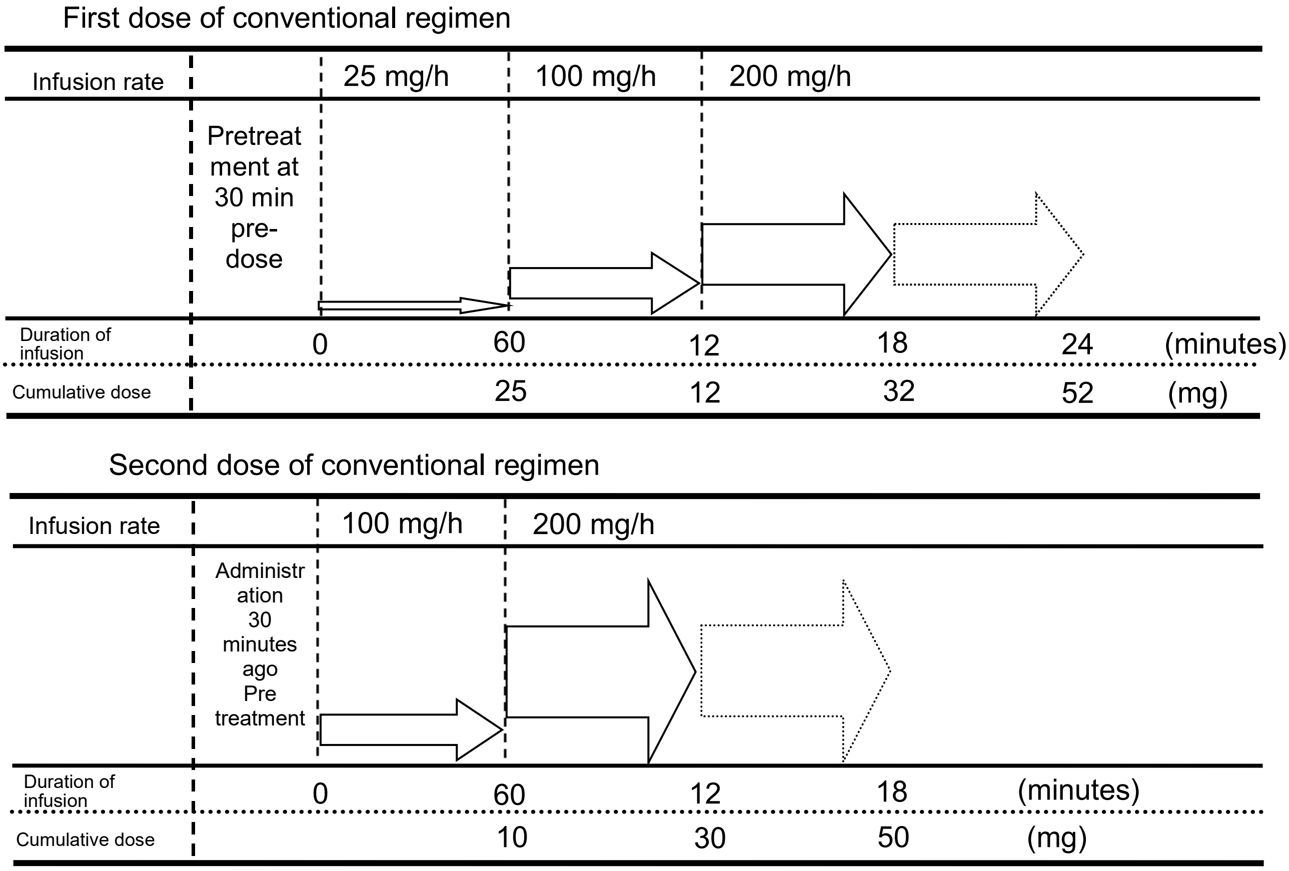


Figure 9-3 Conventional regimen

(4) In non-Japanese clinical studies of IDEC-C2B8 in autoimmune diseases, HACA tended to be produced in subjects in whom IDEC-C2B8 failed to reduce peripheral B cells sufficiently. Therefore, the second and subsequent doses should be administered while closely monitoring the subject's condition.

(5) IDEC-C2B8 administration to patients at high risk of developing infusion reaction

Since subjects with existing or previous respiratory disease are at high risk of developing dyspnea and/or bronchospasm, and subjects with existing or previous heart disease are at high risk of developing myocardial infarction and/or arrhythmia, IDEC-C2B8 should be administered while carefully and frequently monitoring the subject's condition.

In subjects expected to experience serious infusion reaction, IDEC-C2B8 may be administered at a low IV infusion rate (< 200 mg/h) without increasing the rate.

### 9.2.3 Pretreatment before study drug administration

Pretreatment with an oral antipyretic analgesic, an oral antihistamine, and intravenous methylprednisolone will be given 30 (±15) minutes before each study drug administration to prevent infusion reaction.

(1) Oral antipyretic analgesic: Acetaminophen (e.g., CALONAL®)

1) Subjects with a height-based standard weight of ≥ 50 kg or subjects aged 16 years or older will receive acetaminophen at a dose of 300 mg (tablets).
2) Subjects who do not fall under 1) or who cannot take tablets orally will receive acetaminophen at a standard dose of 10 to 15 mg/kg (0.5 mL/kg for syrup, up to 300 mg).

(2) Oral antihistamine: D-chlorpheniramine maleate (e.g., POLARAMINE®)

1) Subjects with a height-based standard weight of ≥ 50 kg or subjects aged 16 years or older will receive an oral dose of 2.0 mg (tablets).
2) Subjects who do not fall under 1) or who cannot take tablets orally will receive d-chlorpheniramine maleate in syrup or dry syrup at the following doses:

| Age | Dose | | |
|---|---|---|---|
| | D-chlorpheniramine maleate | Syrup (content of 0.04%) | Dry syrup (content of 0.2%) |
| < 3 years | 0.4mg | 1.0mL | 0.2g |
| 3 to < 5 years | 0.6mg | 1.5mL | 0.3g |
| 5 to < 8 years | 0.8mg | 2.0mL | 0.4g |
| 8 to < 12 years | 1.0mg | 2.5mL | 0.5g |
| 12 to 15 years | 1.2mg | 3.0mL | 0.6g |

(3) Intravenous corticosteroid: Methylprednisolone (e.g., Solu-Medrol®)

1) Subjects with a height-based standard weight of ≥ 50 kg or subjects aged 16 years or older will receive an IV infusion of methylprednisolone at a dose of 125 mg.
2) Subjects who do not fall under 1) will receive methylprednisolone at a dose of 1.0 to 1.5 mg/kg, the dose for pediatric bronchial asthma.

### 9.2.4 Management of infusion reaction associated with IV infusion of study drug

If infusion reaction occurs during IV infusion, the IV infusion will be slowed or interrupted according to the severity of the reaction as described below, and supportive care will be provided as needed.

(1) Grade 1

The investigator will make a clinical decision whether to continue the IV infusion at a reduced or unchanged infusion rate or interrupt the infusion temporarily. If IV infusion is temporarily interrupted, the infusion will be resumed at a rate equal to or less than half of the previous rate once the symptoms resolve.

(2) Grade 2

The investigator will make a clinical decision whether to continue the IV infusion at a reduced infusion rate or interrupt the infusion temporarily. If IV infusion is temporarily interrupted, the infusion will be

resumed at a rate equal to or less than half of the previous rate once the symptoms improve to ≤ Grade 1.

The investigator will make a clinical decision to resume the infusion after temporary interruption or increase the infusion rate after resumption of infusion or reduction in infusion rate based on Figure 9-2 and Figure 9-3.

While the time from interruption of infusion to resumption of infusion is not specified, infusion should be completed within 24 hours of preparation, and a subject who cannot complete infusion will be discontinued from further study treatment (11.10).

(3) Grade 3 or higher nonhematologic toxicity (excluding abnormal laboratory values)
The IV infusion will be discontinued and supportive care will be provided as needed. The subject will be discontinued from further study treatment.

Supportive care includes (1) non-steroidal antipyretic analgesics (e.g., pyrexia, analgesia), (2) antihistamines (allergic symptoms), (3) antibiotics/antivirals, (4) antihypertensives/vasopressors/vasodilators, (5) antiemetics, (6) stomach medicine/antidiarrheals/laxatives, (7) oxygen inhalation, and (8) other medications deemed necessary by the Investigator. If serum sickness-like symptoms are observed, symptomatic treatment with steroid will be provided.

## 9.3 Change of dose or date of study drug administration (blinded period)

### 9.3.1 Change of dose

The specified dose per IV infusion cannot be changed. If the dose specified at each time point cannot be administered due to an AE, further study treatment will be discontinued.

### 9.3.2 Change of date of second study drug administration

(1) If the study drug cannot be administered as scheduled due to holidays or subject's personal circumstances, the date of administration may be changed within 2 days (administration may be delayed by up to 7 days if the study drug cannot be administered as scheduled due to long holidays such as the year-end and New Year holidays). After the change, the next dose will be administered within 7±2 days from the previous dose.

(2) If, in the opinion of the investigator, the study drug cannot be administered as scheduled due to an AE, administration may be delayed by up to 7 days (administration must be delayed if Grade 3 or higher nonhematologic toxicity is observed before the start of administration). If the date of delayed administration falls on a holiday, 2 additional days (a total of 9 days) of delay will be allowed. However, if more than 7 days of delay is required due to an AE, further study treatment will be discontinued.

## 9.4 Treatment with prednisolone for relapse during the blinded observation period

In this study, relapse and date of relapse are defined as described below.

The investigator will assess relapse at the medical examination based on the results of urine protein test performed at the study site.

| | |
|---|---|
| Relapse | Any of the following conditions requiring prednisolone treatment:<br>[1] Morning urine protein dipstick ≥ 3+ (or ≥ 300 mg/dL in quantitative urine protein test) for 3 consecutive days<br>[2] Urine protein dipstick ≥ 2+ (or ≥ 100 mg/dL in quantitative urine protein test) and serum albumin ≤ 3.0 g/dL |
| Date of relapse | The first date of morning urine protein dipstick ≥ 3+ (or ≥ 300 mg/dL in quantitative urine protein test) for 3 consecutive days or date of urine protein dipstick ≥ 2+ (or ≥ 100 mg/dL in quantitative urine protein test) and serum albumin ≤ 3.0 g/dL |

Any relapse during the blinded observation period (7.3.1) will be treated with prednisolone according to the relapse treatment method of the ISKDC, with secondary adrenocortical insufficiency taken into account.
Treatment with prednisolone will be started within 14 days after the day of relapse. The dose of prednisolone will be determined based on the height at the diagnosis of relapse according to the height-specific dose table (Appendix 2).
In the event of an adverse reaction to prednisolone, the dose of prednisolone will be reduced as appropriate if deemed necessary by the investigator.

(1) 60 mg/m$^2$/day (up to 60 mg/day) in 3 divided doses every day (or in 2 divided doses until negative urine protein dipstick for 3 days if deemed necessary by the Investigator)
(2) 60 mg/m$^2$ (up to 60 mg/day) once every other morning for 14 days
(3) 30 mg/m$^2$ (up to 30 mg/day) once every other morning for 14 days
(4) 15 mg/m$^2$ (up to 15 mg/day) once every other morning for 14 days and discontinued

### 9.5 Early key opening after determination of treatment failure (1)

For subjects determined as treatment failure (1), the efficacy and safety up to recurrence will be assessed in parallel with treatment with prednisolone for relapse, with entry to the open-label period taken into account, followed by early key opening (8.3.2).
A subject who is found to have been assigned to the placebo group after early key opening may enter the open-label period (IDEC-C2B8 treatment) if he/she wishes to receive IDEC-C2B8.

### 9.6 Confirmation of treatment criteria for IDEC-C2B8 (open-label period) treatment

The treatment criteria for IDEC-C2B8 (open-label period) treatment will be confirmed within 7 days from the day of confirmation of remission after relapse. In the event of relapse after confirmation of treatment criteria, the first dose of IDEC-C2B8 (open-label period) may be administered beyond 14 days from the day of confirmation of treatment criteria if the disease is steroid-sensitive (remission within 4 weeks of starting daily prednisolone treatment at a dose of 60 mg/m2/day). In such a case, the treatment criteria must be reconfirmed.
(1) The investigator will perform tests necessary to confirm the IDEC-C2B8 treatment criteria. If virology is

performed within 85 days before confirmation of the IDEC-C2B8 treatment criteria or the peripheral B-cell count is measured within 35 days before the confirmation of the IDEC-C2B8 treatment criteria, it is not necessary to repeat it.

(2) The investigator will confirm that the IDEC-C2B8 treatment criteria are met.
The treatment criteria for IDEC-C2B8 (open-label period) treatment are described in 9.6.1.

(3) If the treatment criteria are met, the investigator will access the EDC to enter the information necessary for confirmation of the IDEC-C2B8 treatment criteria.

(4) If it is determined via the EDC that the IDEC-C2B8 treatment criteria are met, the investigator will check the "IDEC-C2B8 Treatment Criteria Confirmation Form" for the subject to enter the open-label period. Entry of the subject to the open-label period will be communicated to the investigational product provider.

(5) The investigator will fill out the "Drug Supply Request Form" and send it to the investigational product provider by e-mail.
<Investigational product provider> GCP Office, Prescription Products Development Department, Zenyaku Kogyo Company, Limited

5-6-15 Otsuka, Bunkyo-ku, Tokyo 112-8650, Japan
Phone: 03-3946-1113 FAX: 03-3946-1202
E-mail address: GCPjimukyoku@mail.zenyaku.co.jp
Business hours: 9:00 to 17:00 on weekdays

(6) The investigational product provider will confirm the consistency between the "IDEC-C2B8 Treatment Criteria Confirmation Form" and the "Drug Supply Request Form" and deliver IDEC-C2B8 (for the open-label period) to the study site.

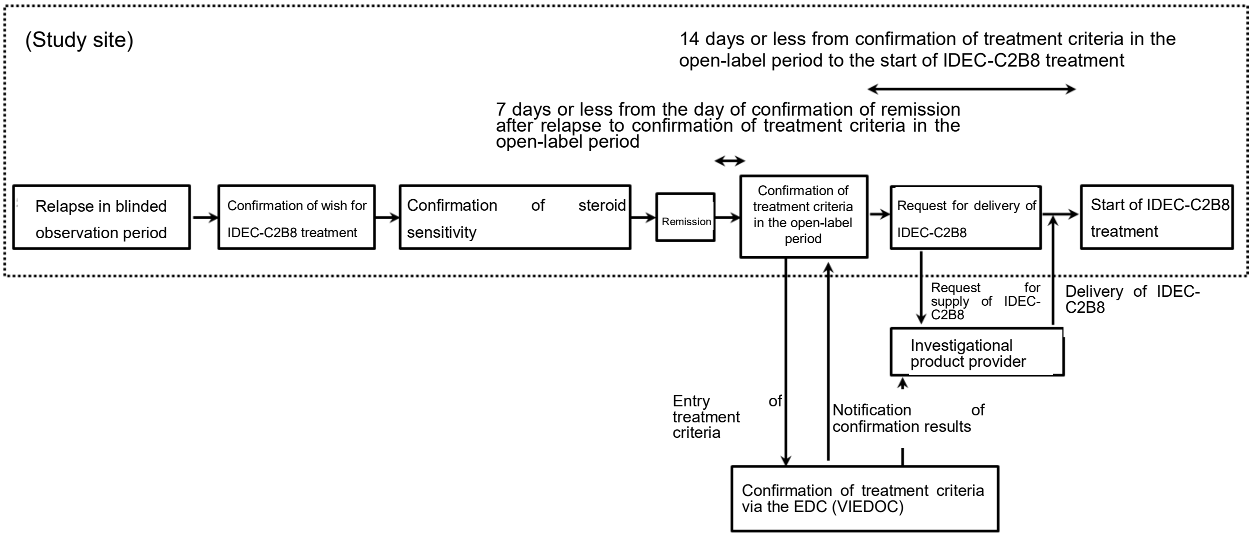


### 9.6.1 Treatment criteria for IDEC-C2B8 (open-label period) treatment

➢ Inclusion criteria

Subjects who meet all of the following criteria will be included in the open-label period:

(1) Patients who received placebo during the blinded period and experienced relapse between the day of first

study drug administration (Day 1 of the blinded period) and the day before Week 25 (Day 168 of the blinded period) (subjects with treatment failure (1) who received placebo)

(2) Patients who have not been treated with immunosuppressants (e.g., cyclosporine, cyclophosphamide, mizoribine), excluding topical drugs used to treat other diseases, since determination of treatment failure (1)

(3) Patients with steroid sensitivity (remission within 4 weeks of starting daily prednisolone treatment at a dose of 60 mg/$m^2$/day) in the treatment of recurrence corresponding to treatment failure (1)

(4) Patients with ≥ 5 CD20-positive cells* per μL of peripheral blood * CD19-positive cells may be used at a study site where CD20-positive cells cannot be measured.

(5) Patients who can be hospitalized for 2 days and 1 night from the day of administration to the next day for the scheduled day of first IDEC-C2B8 administration and can visit the study site throughout the observation phase

[Rationale for the inclusion criteria]

(1) To include only patients who can enter the open-label period

(2) To evaluate the efficacy

(3) Because steroid sensitivity is the most important prognostic factor

(4) Because IDEC-C2B8 is a monoclonal antibody specific for CD20 antigen

(5) To administer IDEC-C2B8 safely and to promptly detect and manage AEs immediately after administration

- Exclusion criteria

Patients who meet any of the following criteria will not be included in the study:

(1) Patients with any of the following infections (1) to (4):

1) Patients with serious infection requiring inpatient treatment (e.g., pneumonia, pyelonephritis)

2) Patients with opportunistic infection (e.g., cytomegalovirus infection, systemic fungal infection, Pneumocystis infection, non-tuberculous mycobacterial infection)

3) Patients with active tuberculosis

4) Patients with previous or suspected tuberculosis infection

(2) Patients with angina pectoris, cardiac failure, myocardial infarction, or severe arrhythmia (Grade 4 findings in the Common Terminology Criteria for Adverse Events v4.0 Japanese JCOG version [CTCAE v4.0-JCOG] [corresponding to the CTCAE v4.03/MedDRA v12.0 described as MedDRA/Jv20.1 in Japanese, 12 September 2017])

(3) Patients who have received live vaccine within 4 weeks before confirmation of IDEC-C2B8 treatment criteria

(4) Patients with uncontrolled hypertension* despite treatment with antihypertensives at the time of confirmation of IDEC-C2B8 treatment criteria * ≥ 99th percentile in the pediatric sex- and age-specific blood pressure norms table[22] (Appendix 4)

(5) Patients with reduced renal function (estimated glomerular filtration rate** < 60 mL/min/1.73 $m^2$) at the

time of confirmation of IDEC-C2B8 treatment criteria ** See the estimated glomerular filtration rate[23)-25)] (Appendix 5).

(6) Patients with autoimmune disease (e.g., Hashimoto's thyroiditis [chronic thyroiditis], Crohn's disease, ulcerative colitis, rheumatoid arthritis, idiopathic thrombocytopenic purpura, systemic lupus erythematosus, autoimmune hemolytic anemia, sclerosis) or vascular purpura

(7) Patients with malignancy (including "suspected" malignancy with no definitive diagnosis)

(8) Patients with a history of drug allergy to methylprednisolone, acetaminophen, or d-chlorpheniramine maleate

(9) Patients who have at least one of the following laboratory parameters at the time of confirmation of IDEC-C2B8 treatment criteria in the measurement after informed consent and within 14 days before confirmation of IDEC-C2B8 treatment criteria for 1) to 5) and after informed consent and within 85 days before confirmation of IDEC-C2B8 treatment criteria for 6) to 7):

1) White blood cells < 3,000/μL
2) Neutrophils < 1,500/μL
3) Platelets < 50,000/μL
4) AST (GOT) ≥ 2.5 times the upper limit of normal in the pediatric age-specific liver escape enzyme (GOT) norms table[26)] (Appendix 6)
5) ALT (GPT) ≥ 2.5 times the upper limit of normal in the pediatric age-specific liver escape enzyme (GPT) norms table[26)] (Appendix 7)
6) Positive for HBs antigen, HBs antibody, HBc antibody, or HCV antibody, excluding patients positive only for HBs antibody who have been vaccinated against HB and are negative for HBV-DNA (less than detection sensitivity) at enrollment
7) Positive for HIV antibody

(10) Patients who have used any monoclonal antibody (mouse, rat, chimeric, or human), including rituximab

(11) Patients who are planning to participate in another clinical trial during participation in the study

(12) Patients of childbearing potential who do not agree to use contraception during the observation phase

(13) Females who are pregnant, who may be pregnant, or who are breastfeeding

(14) Patients who, in the opinion of the Investigator, are ineligible for participation in the open-label period

[Rationale for the exclusion criteria]

(1) to (9) Because it is not desirable to include these patients in the open-label period for safety reasons

(10) and (11) Because the safety or efficacy evaluation in the open-label period may be affected

(12) and (13) Since IDEC-C2B8 is homogeneous to immunoglobulins, which are known to cross the placenta and enter the fetus, there is concern that IDEC-C2B8 administered to a mother may affect the fetus.

(14) To exclude patients who are not eligible for participation in the study

## 9.7 IDEC-C2B8 (open-label period) treatment

The investigator will administer the first dose of IDEC-C2B8 within 14 days from the day of confirmation of

treatment criteria for IDEC-C2B8 (open-label period) treatment (the day of first IDEC-C2B8 administration is designated as Week 1 [Day 1 of the open-label period]).

IDEC-C2B8 will be administered at a dose of 375 mg/m$^2$ (up to 500 mg) once a week for 2 weeks (Days 1 and 8 of the open-label period).

The dose of IDEC-C2B8 in the open-label period will be determined based on the height at enrollment according to the height-specific dose table (Appendix 3) (using the height at enrollment, not at the time of confirmation of treatment criteria in the open-label period).

The specified dose per IV infusion cannot be changed.

| |
|---|
| Dosage and administration of IDEC-C2B8<br>375 mg/m$^2$ (up to 500 mg) once a week for 2 weeks (Days 1 and 8 of the open-label period) |

In addition, observations, investigations, and tests will be continued until Week 53 (Day 365 of the open-label period) according to the study schedule in Table 11-2, and peripheral B cells, HACA, and blood drug concentrations will also be measured.

Preparation and administration of IDEC-C2B8, pretreatment before IDEC-C2B8 administration, and management of infusion reaction associated with IV infusion of IDEC-C2B8 will follow the procedures in 9.2, "Study drug administration (blinded period)."

## 9.8 Change of dose or date of IDEC-C2B8 administration (open-label period)

Any change of dose or date of IDEC-C2B8 administration (open-label period) will be made according to the procedures in 9.3, "Change of dose or date of study drug administration (blinded period)."

## 9.9 Relapse during the open-label observation period

In this study, relapse and date of relapse are defined as described below.

The investigator will assess relapse at the medical examination based on the results of urine protein test performed at the study site.

| | |
|---|---|
| Relapse | Any of the following conditions requiring prednisolone treatment:<br>[1] Morning urine protein dipstick ≥ 3+ (or ≥ 300 mg/dL in quantitative urine protein test) for 3 consecutive days<br>[2] Urine protein dipstick ≥ 2+ (or ≥ 100 mg/dL in quantitative urine protein test) and serum albumin ≤ 3.0 g/dL |
| Date of relapse | The first date of morning urine protein dipstick ≥ 3+ (or ≥ 300 mg/dL in quantitative urine protein test) for 3 consecutive days or date of urine protein dipstick ≥ 2+ (or ≥ 100 mg/dL in quantitative urine protein test) and serum albumin ≤ 3.0 g/dL |

In the event of relapse during the open-label observation period, observations, investigations, and tests will be performed according to the study schedule in Table 11-2. Relapse will be treated with treatment considered best by the investigator (standard treatment) in accordance with the Clinical Practice Guideline for Pediatric

Idiopathic Nephrotic Syndrome.

## 9.10 Discontinuation criteria for study or IDEC-C2B8 treatment

If any of the conditions listed below occurs, the investigator will discontinue study treatment for the subject and perform investigations as soon as possible as scheduled at the time of discontinuation of study or IDEC-C2B8 treatment (11.10).

After discontinuation of study or IDEC-C2B8 treatment, the subject will remain in the study unless withdrawn from the study as specified in 9.11, “Withdrawal from the study," and the investigator will continue observations, investigations, and tests according to the study schedule (11.1 or 11.2). Subjects withdrawn from study or IDEC-C2B8 treatment due to toxicity or refusal by the subject/legal representative (9.10) will be followed up from the start day of study treatment to the end of the observation phase unless lost to follow-up.

(1) Relapse during study or IDEC-C2B8 treatment
(2) Grade 3 or higher nonhematologic toxicity (excluding abnormal laboratory values) during IV infusion of study drug or IDEC-C2B8 (9.2.4)
(3) Failure to administer study drug or IDEC-C2B8 according to the specified schedule
    1) Failure to complete study drug or IDEC-C2B8 administration within 24 hours of preparation due to an AE (9.2.1)
    2) Failure to administer a specified dose due to an AE (9.3.1)
    3) More than 7 days of delay due to an AE (9.3.2 (2))
(4) Subject's (aged 20 years or older) or legal representative's request for discontinuation of study or IDEC-C2B8 treatment
(5) Found to be ineligible at enrollment (not meeting the inclusion criteria or meeting the exclusion criteria) (including being found to be ineligible at enrollment after the start of study or IDEC-C2B8 treatment)
(6) Any other condition that, in the opinion of the investigator, makes it difficult to continue study or IDEC-C2B8 treatment, for instance, due to an AE

## 9.11 Withdrawal from the study

A subject will be withdrawn from the study if he/she is not only withdrawn from study or IDEC-C2B8 treatment, but also becomes unable to comply with the study schedule (11.1), including efficacy and safety assessments, for any reason described in (1) to (4) after the start of study treatment.

(1) Subject's (aged 20 years or older) or legal representative's request for withdrawal from the study or withdrawal of consent to participate in the study
(2) Failure to visit the study site due to the subject's personal reason (e.g., being too busy, moving, changing hospital, SAE, death)
(3) Discontinuation of the study itself
(4) Any other condition that, in the opinion of the investigator, makes it difficult to remain in the study

In the event of (1) to (4), the investigator will promptly withdraw the subject from the study and report the time and reason for withdrawal. Appropriate efforts should be made to identify the reason, with full respect for the rights of the subject.
At that time, tests will be performed to ensure the safety of the subject (only peripheral B-cell count in the blinded period and peripheral B-cell count, HACA, and blood IDEC-C2B8 concentration in the open-label period). The investigation items scheduled at the time of withdrawal from the study (11.11) will be observed, investigated, or tested to the extent possible.
Any SAE at the time of withdrawal should be appropriately treated by the investigator, and the outcome of the event should be followed up by telephone or letter as long as possible until the end of the entire study (scheduled last observation date for the last enrolled subject).

# 10 Concomitant medications and therapies/post-study treatment

## 10.1 Concomitant medications and therapies

### 10.1.1 Reporting of concomitant medication and therapy

(1) From the day of informed consent to the day before first study drug administration
The name and route of administration of concomitant medication and the name and purpose of concomitant therapy will be entered into the EDC if used to treat nephrotic syndrome, concomitant disease, or comorbidity.

(2) Observation phase (from Day 1 of the blinded period to Day 365 of the blinded/open-label period)
The name, route of administration, and purpose of all concomitant medications and therapies used during the observation phase, regardless of the treatment purpose (e.g., underlying disease, AE), will be entered into the EDC. All medications used to treat concomitant disease since before the start of the study will also be entered.

### 10.1.2 Prohibited medications and therapies

Concomitant use of the following medications and therapies will be prohibited during the study period (from the day of informed consent to the end of the blinded/open-label observation period [Day 365 of the blinded/open-label period]).

(1) Commercially available rituximab
(2) Immunosuppressants or immunosuppressive alkylating agents, etc.,
    excluding standard treatment given for treatment failure (7.3.2) or after relapse during the open-label observation period (9.9)
(3) Plasmapheresis
(4) Live vaccines
    Excluding subjects whose peripheral B-cell count has recovered to the level at the time of confirmation of IDEC-C2B8 treatment criteria during the open-label observation period
(5) Other investigational products or drugs not approved in Japan

### 10.2 Post-study treatment

Post-study treatment is not specified but will be given at the discretion of the investigator. It is recommended to treat relapse, frequently relapsing, steroid-dependent, or steroid-resistant nephrotic syndrome according to the Clinical Practice Guideline if diagnosed.[3)]

## 11 Observations, tests, and investigations

### 11.1 Schedule of observations, tests, and investigations

During the study period, the investigator will perform observations, tests, and investigations according to the specified schedule. All blood samples on the day of study drug administration will be collected immediately before administration.

If study treatment is discontinued, investigations will be performed as soon as possible as scheduled at the time of discontinuation of study or IDEC-C2B8 treatment (11.10), and observations, investigations, and tests will be continued according to the study schedule in Table 11-1 or Table 11-2.

Deviation "within ±7 days" will be allowed from Week 5 (Day 29 of the blinded/open-label period) to Week 13 (Day 85 of the blinded/open-label period). Deviation "within ±14 days" will be allowed from Week 17 (Day 113 of the blinded/open-label period) to Week 53 (Day 365 of the blinded/open-label period), but the next observation, test, or investigation must be performed at least 14 days apart.

Any deviation made will be effective only on the relevant day, and subsequent observations, tests, and investigations will be performed as specified. Any change of date of study drug administration will be entered into the EDC with the reason, even if it is within an acceptable range.

Table 11-1 Study schedule in the blinded period

(* also applicable to early key opening followed by standard treatment)

| | Screening phase*1 | Blinded observation period (study treatment period) | | Blinded observation period (after study treatment) | | | | | | | | | | | | | Relapse | Withdrawal from the study |
|---|---|---|---|---|---|---|---|---|---|---|---|---|---|---|---|---|---|---|
| Day | | 1 | 8 | 29 | 57 | 85 | 113 | 141 | 169 | 197 | 225 | 253 | 281 | 309 | 337 | 365 | | |
| Week | | 1 | 2 | 5 | 9 | 13 | 17 | 21 | 25 | 29 | 33 | 37 | 41 | 45 | 49 | 53 | | |
| Month (4 weeks/month) | | | | 1 | 2 | 3 | 4 | 5 | 6 | 7 | 8 | 9 | 10 | 11 | 12 | | | |
| Informed consent | ○ | | | | | | | | | | | | | | | | | |
| Subject demographics | ○ | | | | | | | | | | | | | | | | | |
| Study drug administration | | ○ | ○ | | | | | | | | | | | | | | | |
| Medical examination | ○ | ○ | ○ | ○ | ○ | ○ | ○ | ○ | ○ | ○ | ○ | ○ | ○ | ○ | ○ | ○ | ○ | ○ |
| Concomitant medication survey | ○ | ○ | ○ | ○ | ○ | ○ | ○ | ○ | ○ | ○ | ○ | ○ | ○ | ○ | ○ | ○ | ○ | ○ |
| Patient diary | ○ | ○ | ○ | ○ | ○ | ○ | ○ | ○ | ○ | ○ | ○ | ○ | ○ | ○ | ○ | ○ | ○ | |
| Height/weight | ○ | ○ | ○ | ○ | ○ | ○ | ○ | ○ | ○ | ○ | ○ | ○ | ○ | ○ | ○ | ○ | ○ | ○ |
| Blood pressure, pulse, | ○ | ○ | ○ | ○ | ○ | ○ | ○ | ○ | ○ | ○ | ○ | ○ | ○ | ○ | ○ | ○ | ○ | ○ |
| Pregnancy test | ○*2 | | | | | | | | | | | | | | | | | |
| Virology | ○ | | | | | | | | | | | | | | | | | |
| Electrocardiography | ○ | | | | | | | | | | | | | | | ○ | | ○ |
| Chest X-ray | ○ | | | | | | | | | | | | | | | ○ | | ○ |
| Recurrence assessment | | ○ | ○ | ○ | ○ | ○ | ○ | ○ | ○ | ○ | ○ | ○ | ○ | ○ | ○ | ○ | ○ | ○ |
| Adverse event assessment | | ○ | ○ | ○ | ○ | ○ | ○ | ○ | ○ | ○ | ○ | ○ | ○ | ○ | ○ | ○ | ○ | ○ |
| Hematology | ○*3 | ○ | ○ | ○ | ○ | ○ | ○ | ○ | ○ | ○ | | ○ | | ○ | | ○ | ○ | ○ |
| Serum chemistry | ○*3 | ○ | ○ | ○ | ○ | ○ | ○ | ○ | ○ | ○ | | ○ | | ○ | | ○ | ○ | ○ |
| Immunoglobulins | | ○ | | ○ | | ○ | | | ○ | | | ○ | | | | ○ | | ○ |
| eGFR | ○ | | | | | | | | | | | | | | | ○ | | ○ |
| Urinalysis | ○ | ○ | ○ | ○ | ○ | ○ | ○ | ○ | ○ | ○ | ○ | ○ | ○ | ○ | ○ | ○ | ○ | ○ |
| Peripheral B-cell count*4*5 | ○ | ● | | ● | | ● | | ● | | ● | | ● | | ● | | ● | ● | ● |

○ Measured/performed locally ● Measured externally (central measurement)

*1 Performed after informed consent and within 35 days before enrollment, except for hematology and serum chemistry

*2 Confirmed by serum or urine HCG test in postmenarchal females only

*3 Performed after informed consent and within 14 days before enrollment. Any test data collected after the last relapse and within 14 days before enrollment may be used even if collected before informed consent, requiring no re-examination.

*4 During the screening phase, the CD20-positive or CD19-positive cell count will be measured locally. During the blinded observation period, the CD19-positive cell count will be measured centrally.

*5 It is not necessary to measure peripheral B cells after treatment failure in subjects in the placebo group who are determined as treatment failure (1) and switched to standard treatment or subjects in the placebo group who are determined as treatment failure (2) or (3).

Table 11-2 Study schedule in the open-label period (IDEC-C2B8 treatment)

| | Switching period | Open-label observation period (IDEC-C2B8 treatment period) | | Open-label observation period (after the end of IDEC-C2B8 treatment) | | | | | | | | | | | | | | Withdrawal from the study |
|---|---|---|---|---|---|---|---|---|---|---|---|---|---|---|---|---|---|---|
| Day | | 1 | 8 | 29 | 57 | 85 | 113 | 141 | 169 | 197 | 225 | 253 | 281 | 309 | 337 | 365 | Relapse | |
| Week | | 1 | 2 | 5 | 9 | 13 | 17 | 21 | 25 | 29 | 33 | 37 | 41 | 45 | 49 | 53 | | |
| Month (4 weeks/month) | | | | 1 | 2 | 3 | 4 | 5 | 6 | 7 | 8 | 9 | 10 | 11 | 12 | | | |
| Confirmation of treatment criteria | ○*1 | | | | | | | | | | | | | | | | | |
| IDEC-C2B8 administration | | ○*2 | ○ | | | | | | | | | | | | | | | |
| Medical examination | ○ | ○ | ○ | ○ | ○ | ○ | ○ | ○ | ○ | ○ | ○ | ○ | ○ | ○ | ○ | ○ | ○ | ○ |
| Concomitant medication survey | ○ | ○ | ○ | ○ | ○ | ○ | ○ | ○ | ○ | ○ | ○ | ○ | ○ | ○ | ○ | ○ | ○ | ○ |
| Patient diary | ○ | ○ | ○ | ○ | ○ | ○ | ○ | ○ | ○ | ○ | ○ | ○ | ○ | ○ | ○ | ○ | ○ | |
| Height/weight | ○ | ○ | ○ | ○ | ○ | ○ | ○ | ○ | ○ | ○ | ○ | ○ | ○ | ○ | ○ | ○ | ○ | ○ |
| Blood pressure, pulse, body temperature | ○ | ○ | ○ | ○ | ○ | ○ | ○ | ○ | ○ | ○ | ○ | ○ | ○ | ○ | ○ | ○ | ○ | ○ |
| Pregnancy test | ○*3 | | | | | | | | | | | | | | | | | |
| Virology | ○*4 | | | | | | | | | | | | | | | | | |
| Electrocardiography | ○ | | | | | | | | | | | | | | | ○ | | ○ |
| Chest X-ray | ○ | | | | | | | | | | | | | | | ○ | | ○ |
| Recurrence assessment | | ○ | ○ | ○ | ○ | ○ | ○ | ○ | ○ | ○ | ○ | ○ | ○ | ○ | ○ | ○ | ○ | ○ |
| Adverse event assessment | ○ | ○ | ○ | ○ | ○ | ○ | ○ | ○ | ○ | ○ | ○ | ○ | ○ | ○ | ○ | ○ | ○ | ○ |
| Hematology | ○*5 | ○ | ○ | ○ | ○ | ○ | ○ | ○ | ○ | ○ | | ○ | | ○ | | ○ | ○ | ○ |
| Serum chemistry | ○*5 | ○ | ○ | ○ | ○ | ○ | ○ | ○ | ○ | ○ | | ○ | | ○ | | ○ | ○ | ○ |
| Immunoglobulins | | ○ | | ○ | | ○ | | | ○ | | | ○ | | | | ○ | | ○ |
| eGFR | ○ | | | | | | | | | | | | | | | ○ | | ○ |
| Urinalysis | ○ | ○ | ○ | ○ | ○ | ○ | ○ | ○ | ○ | ○ | ○ | ○ | ○ | ○ | ○ | ○ | ○ | ○ |
| Peripheral B-cell count*6 | ○*7 | ● | | ● | | ● | | ● | | ● | | ● | | ● | | ● | ● | ● |
| HACA | | ●*8 | | | | ● | | | ● | | | ● | | | | ● | ● | ● |
| Blood drug concentration | | ●*9 | ●*9 | ● | | ● | ● | | ● | | | ● | | | | ● | | ● |

○ Measured/performed locally ● Measured externally (central measurement)

*1 Within 7 days from the day of confirmation of remission
*2 Within 14 days from the day of confirmation of IDEC-C2B8 treatment criteria
*3 Confirmed by serum or urine HCG test in postmenarchal females only
*4 No re-examination will be required if performed within 85 days before confirmation of IDEC-C2B8 treatment criteria.
*5 Performed within 14 days before confirmation of IDEC-C2B8 treatment criteria
*6 During the switching period, the CD20-positive or CD19-positive cell count will be measured locally. During the open-label observation period, the CD19-positive cell count will be measured centrally.
*7 No re-measurement will be required if measured within 35 days before confirmation of IDEC-C2B8 treatment criteria.
*8 Measured before IDEC-C2B8 administration
*9 Measured immediately before IDEC-C2B8 administration and 30 minutes after the end of IDEC-C2B8 administration

## 11.2 Observations, tests, and investigations at screening

During the screening phase, the investigator will observe, test, and investigate the following items and enter the results into the EDC:

Table 11-3 Observation, test, and investigation items at screening

| Item | Details | Where | |
|---|---|---|---|
| | | Local | External |
| Subject demographics | Sex, date of birth, and race | ○ | |
| | Height and weight | ○ | |
| Date of informed consent | | ○ | |
| Disease/treatment history of nephrotic syndrome | Date of first diagnosis of nephrotic syndrome, date of relapse after onset of nephrotic syndrome, and date of diagnosis of frequent relapse/steroid dependence | ○ | |
| Medical history and concomitant disease | | ○ | |
| Concomitant medication/therapy*1 | Name and route of administration of medication and/or name and purpose of therapy | ○ | |
| Steroid sensitivity | | ○ | |
| Vital signs | Pulse rate, body temperature, and blood pressure (systolic and diastolic) | ○ | |
| Hematology*2 | WBC, neutrophil count, Plt, RBC, Hb, Ht, and WBC differential | ○ | |
| Serum chemistry*2 | AST, ALT, CRP, serum creatinine, BUN, uric acid, total protein, serum albumin, Na, K, Ca, P, Cl, and eGFR | ○ | |
| Peripheral B-cell count | CD20-positive or CD19-positive cell count | ○ | |
| Virology | HIV antibody, HBs antigen, HBs antibody, HBc antibody, and HCV antibody (and HBV-DNA quantification only for HBs antibody-positive patients) | ○ | |
| Pregnancy test*3 | Urine or blood HCG | ○ | |
| Urinalysis*4 | Qualitative morning urine protein, qualitative morning urine occult blood, quantitative morning urine protein, and quantitative morning urine creatinine | ○ | |
| Electrocardiography | | ○ | |
| Chest X-ray | | ○ | |

*1 Any medication that has continued to be used to treat nephrotic syndrome or concomitant disease will be recorded.
*2 Performed after informed consent and within 14 days before enrollment
*3 Mandatory for postmenarchal females only
*4 Urinalysis will be performed on the day of confirmation of remission (if informed consent is obtained after confirmation of remission, data collected from the day of informed consent to the day of enrollment will be used).

## 11.3 Investigations during the study treatment period of the blinded observation period

### 11.3.1 Immediately before study drug administration

The investigator will observe and test the items listed below immediately before administration on each day of study drug administration and enter the results into the EDC. In addition, samples to be outsourced to a contract laboratory will be collected.

Table 11-4 Investigation items immediately before study drug administration

| Item | Details | Where | |
|---|---|---|---|
| | | Local | External |
| Concomitant medication/therapy | Name and route of administration of medication and/or name and purpose of therapy | ○ | |
| General symptoms | Subjective and objective findings | ○ | |
| Height and weight | | ○ | |
| Vital signs | Blood pressure (systolic and diastolic), pulse rate, and body temperature | ○ | |
| Relapse assessment | Date of relapse and steroid sensitivity or resistance | ○ | |
| Hematology | WBC, WBC differential, RBC, Hb, Ht, and Plt | ○ | |
| Serum chemistry | BUN, serum creatinine, uric acid, total protein, serum albumin, AST, ALT, Na, K, Ca, P, Cl, and CRP | ○ | |
| Immunoglobulins*1 | IgG, IgM, and IgA | ○ | |
| Peripheral B-cell count*1 | CD19-positive cells | | ○ |
| Urinalysis | Qualitative morning urine protein, qualitative morning urine occult blood, quantitative morning urine protein, and quantitative morning urine creatinine | ○ | |
| Adverse event assessment | | ○ | |

*1 Only immediately before first administration

### 11.3.2 During IV infusion of study drug

The investigator will test and observe the items listed below during IV infusion of study drug and enter the results into the EDC.

Subjects will be frequently observed for infusion reaction and any infusion reaction will be treated with appropriate measures such as reduction in IV infusion rate or supportive care (9.2.4).

(1) Dose of study drug
(2) Start time of IV infusion (time when IV infusion of study drug is actually started) and infusion rate
(3) Time when IV infusion is accelerated, slowed, or interrupted and infusion rate after change, if applicable
(4) End time of IV infusion (time when IV infusion of specified dose of study drug is completed)
(5) Presence or absence of AEs during IV infusion

AEs such as infusion reaction will be entered into the EDC with the date and time of onset, type and seriousness, relationship to study drug, and presence or absence of supportive care (type, dose, and route of administration, if applicable), and vital signs (blood pressure, pulse rate, and body temperature) will be

measured if necessary.

(6) Reason and date and time of discontinuation of study treatment, if applicable

(7) Time course if IV infusion is slowed or temporarily interrupted due to infusion reaction, etc. and then resumed

(8) Error in drug administration (overdose, medication error, or misuse/abuse) in terms of the date of the error, action taken after the error, presence or absence of ADRs due to the error, nature, time course, and outcome of the error, etc., if applicable

### 11.3.3 From the end of IV infusion of study drug to the following day

Subjects will be kept at rest for at least 30 minutes after completion of each study drug administration. Subjects will be observed for general symptoms and monitored for vital signs (blood pressure, pulse rate, and body temperature) 1 hour (±30 minutes) after the end of IV infusion. AEs such as infusion reaction will be entered into the EDC as such with the date and time of onset and details.

Special attention should be paid to the onset of infusion reaction for 24 hours after the start of study drug administration.

## 11.4 Investigations during the blinded observation period from the end of the study treatment period to the end of the blinded observation period

At each scheduled visit, the investigator will perform tests and observations according to the study schedule in Table 11-1 and enter the results into the EDC. In addition, samples to be outsourced to a contract laboratory will be collected.

Table 11-5 Investigation items during the blinded observation period

| Item | Details | Where | |
|---|---|---|---|
| | | Local | External |
| Concomitant medication/therapy | Name and route of administration of medication and/or name and purpose of therapy | ○ | |
| General symptoms | Subjective and objective findings | ○ | |
| Height and weight | | ○ | |
| Vital signs | Blood pressure (systolic and diastolic), pulse rate, and body temperature | ○ | |
| Relapse assessment | Presence or absence of relapse, date of relapse, and presence or absence of steroid sensitivity or resistance | ○ | |
| Adverse event assessment | | ○ | |
| Hematology | WBC, WBC differential, RBC, Hb, Ht, and Plt | ○ | |
| Serum chemistry | BUN, serum creatinine, uric acid, total protein, serum albumin, AST, ALT, Na, K, Ca, P, Cl, CRP, and eGFR[*1] | ○ | |
| Immunoglobulins | IgG, IgM, and IgA | ○ | |
| Peripheral B-cell count | CD19-positive cells | | ○ |
| Urinalysis | Qualitative morning urine protein, qualitative morning urine occult blood, quantitative morning urine protein, and quantitative morning urine creatinine | ○ | |
| Electrocardiography/chest X-ray[*1] | | ○ | |

*1 Performed only at the end of the blinded observation period

## 11.5 Investigations at the time of relapse during the blinded observation period

If the definition of relapse in this study is met (7.3.1), the investigator will test and observe the items listed below and enter the results into the EDC. In addition, samples to be outsourced to a contract laboratory will be collected.

Table 11-6 Investigation items at the time of recurrence during the blinded observation period

| Item | Details | Where | |
|---|---|---|---|
| | | Local | External |
| Concomitant medication/therapy | Name and route of administration of medication and/or name and purpose of therapy | ○ | |
| General symptoms | Subjective and objective findings | ○ | |
| Height and weight | | ○ | |
| Vital signs | Blood pressure (systolic and diastolic), pulse rate, and body temperature | ○ | |
| Relapse assessment | Presence or absence of relapse, date of confirmation of relapse *1, date of relapse, start date of treatment of recurrence, status of treatment of recurrence, and date of early key opening (if applicable) | ○ | |
| Hematology | WBC, WBC differential, RBC, Hb, Ht, and Plt | ○ | |
| Serum chemistry | BUN, serum creatinine, uric acid, total protein, serum albumin, AST, ALT, Na, K, Ca, P, Cl, and CRP | ○ | |
| Peripheral B-cell count | CD19-positive cells | | ○ |
| Urinalysis | Test results used to diagnose relapse (qualitative morning urine protein, quantitative morning urine protein, and quantitative morning urine creatinine) | ○ | |
| Adverse event assessment | | ○ | |

*1 This refers to the date when urinalysis is performed at the study site to confirm relapse (date of urinalysis).

## 11.6 Investigations at the time of confirmation of treatment criteria for IDEC-C2B8 (open-label period) treatment

Only for subjects who enter the open-label period, the investigator will test and observe the following items and enter the results into the EDC:

Table 11-7 Investigation items at the time of confirmation of treatment criteria for IDEC-C2B8 (open-label period) treatment

| Item | Details | Where | |
|---|---|---|---|
| | | Local | External |
| Concomitant medication/therapy | Name and route of administration of medication and/or name and purpose of therapy | ○ | |
| General symptoms | Subjective and objective findings | ○ | |
| Height | | ○ | |
| Vital signs | Blood pressure (systolic and diastolic) | ○ | |
| Steroid sensitivity | | ○ | |
| Adverse event assessment | | ○ | |
| Hematology*1 | WBC, neutrophil count, and Plt | ○ | |
| Serum chemistry*1 | AST, ALT, CRP, serum creatinine, and eGFR | ○ | |
| Virology*2 | HIV antibody, HBs antigen, HBs antibody, HBc antibody, and HCV antibody (and HBV-DNA quantification only for HBs antibody-positive patients) | ○ | |
| Pregnancy test*3 | Urine or blood HCG (mandatory for postmenarchal females only) | ○ | |
| Peripheral B-cell count*4 | CD20-positive or CD19-positive cell count | ○ | |
| Urinalysis | Qualitative morning urine protein, qualitative morning urine occult blood, quantitative morning urine protein, and quantitative morning urine creatinine | ○ | |
| Electrocardiography/chest X-ray | | ○ | |

*1 Performed within 14 days before confirmation of IDEC-C2B8 treatment criteria
*2 No re-examination will be required if performed within 85 days before confirmation of IDEC-C2B8 treatment criteria.
*3 Mandatory for postmenarchal females only
*4 No re-measurement will be required if measured within 35 days before confirmation of IDEC-C2B8 treatment criteria.

## 11.7 Investigations during the IDEC-C2B8 treatment period of the open-label observation period

### 11.7.1 Immediately before IDEC-C2B8 administration

The investigator will test and observe the items listed below immediately before administration on the day of IDEC-C2B8 administration and enter the results into the EDC. In addition, samples to be outsourced to a contract laboratory will be collected.

Table 11-8 Investigation items immediately before IDEC-C2B8 administration

| Item | Details | Where | |
|---|---|---|---|
| | | Local | External |
| Concomitant medication/therapy | Name and route of administration of medication and/or name and purpose of therapy | ○ | |
| General symptoms | Subjective and objective findings | ○ | |
| Height and weight | | ○ | |
| Vital signs | Blood pressure (systolic and diastolic), pulse rate, and body temperature | ○ | |
| Relapse assessment | Presence or absence of relapse, date of relapse, and presence or absence of steroid sensitivity or resistance | ○ | |
| Adverse event assessment | | ○ | |
| Hematology | WBC, WBC differential, RBC, Hb, Ht, and Plt | ○ | |
| Serum chemistry | BUN, serum creatinine, uric acid, total protein, serum albumin, AST, ALT, Na, K, Ca, P, Cl, and CRP | ○ | |
| Immunoglobulins*1 | IgG, IgM, and IgA | ○ | |
| Peripheral B-cell count*1 | CD19-positive cells | | ○ |
| HACA*1 | | | ○ |
| Blood drug concentration*2 | | | ○ |
| Urinalysis | Qualitative morning urine protein, qualitative morning urine occult blood, quantitative morning urine protein, and quantitative morning urine creatinine | ○ | |

*1 Only immediately before first administration
*2 Measured within 30 minutes before IDEC-C2B8 administration and within 30 minutes after the end of IDEC-C2B8 administration

### 11.7.2 During IV infusion of IDEC-C2B8

The investigator will test and observe the items listed below during IV infusion of IDEC-C2B8 and enter the results into the EDC.

Subjects will be frequently observed for infusion reaction and any infusion reaction will be treated with appropriate measures such as reduction in IV infusion rate or supportive care (9.2.4).

(1) Dose of IDEC-C2B8
(2) Start time of IV infusion (time when IV infusion of IDEC-C2B8 is actually started) and infusion rate
(3) Time when IV infusion is accelerated, slowed, or interrupted and infusion rate after change, if applicable
(4) End time of IV infusion (time when IV infusion of specified dose of IDEC-C2B8 is completed)
(5) Presence or absence of AEs during IV infusion

AEs such as infusion reaction will be entered into the EDC with the date and time of onset, type and seriousness, relationship to IDEC-C2B8, and presence or absence of supportive care (type, dose, and route of administration, if applicable), and vital signs (blood pressure, pulse rate, and body temperature) will be measured if necessary.

(6) Reason and date and time of discontinuation of IDEC-C2B8 treatment, if applicable
(7) Time course if IV infusion is slowed or temporarily interrupted due to infusion reaction, etc. and then resumed
(8) Error in drug administration (overdose, medication error, or misuse/abuse) in terms of the date of the error, action taken after the error, presence or absence of ADRs due to the error, nature, time course, and outcome of the error, etc., if applicable

### 11.7.3 From the end of IV infusion of IDEC-C2B8 to the following day

Subjects will be kept at rest for at least 30 minutes after completion of each IDEC-C2B8 administration. Subjects will be observed for general symptoms and monitored for vital signs (blood pressure, pulse rate, and body temperature) 1 hour (±30 minutes) after the end of IV infusion. AEs such as infusion reaction will be entered into the EDC as such with the date and time of onset and details.

Special attention should be paid to the onset of infusion reaction for 24 hours after the start of IDEC-C2B8 administration.

## 11.8 Investigations during the open-label observation period from the end of the IDEC-C2B8 treatment period to the end of the observation phase

At each scheduled visit, the investigator will observe the subject, perform tests and observations according to the study schedule in Table 11-2, and enter the results into the EDC. In addition, samples to be outsourced to a contract laboratory will be collected.

Table 11-9 Investigation items during the open-label observation period

| Item | Details | Where | |
|---|---|---|---|
| | | Local | External |
| Concomitant medication/therapy | Name and route of administration of medication and/or name and purpose of therapy | ○ | |
| General symptoms | Subjective and objective findings | ○ | |
| Height and weight | | ○ | |
| Vital signs | Blood pressure (systolic and diastolic), pulse rate, and body temperature | ○ | |
| Relapse assessment | Presence or absence of relapse, date of relapse, and presence or absence of steroid sensitivity or resistance | ○ | |
| Adverse event assessment | | ○ | |
| Hematology | WBC, WBC differential, RBC, Hb, Ht, and Plt | ○ | |
| Serum chemistry | BUN, serum creatinine, uric acid, total protein, serum albumin, AST, ALT, Na, K, Ca, P, Cl, CRP, and eGFR*1 | ○ | |
| Immunoglobulins | IgG, IgM, and IgA | ○ | |

| | | | |
|---|---|---|---|
| Peripheral B-cell count | CD19-positive cells | | ○ |
| HACA | | | ○ |
| Blood drug concentration | | | ○ |
| Urinalysis | Qualitative morning urine protein, qualitative morning urine occult blood, quantitative morning urine protein, and quantitative morning urine creatinine | ○ | |
| Electrocardiography/chest X-ray*1 | | ○ | |

*1 Performed only at the end of the open-label observation period

## 11.9 Investigations at the time of relapse during the open-label observation period

If the definition of recurrence in this study is met (7.3.1), the investigator will test and observe the items listed below and enter the results into the EDC. In addition, samples to be outsourced to a contract laboratory will be collected.

Table 11-10 Investigation items at the time of relapse during the open-label observation period

| Item | Details | Where | |
|---|---|---|---|
| | | Local | External |
| Concomitant medication/therapy | Name and route of administration of medication and/or name and purpose of therapy | ○ | |
| General symptoms | Subjective and objective findings | ○ | |
| Height and weight | | ○ | |
| Vital signs | Blood pressure (systolic and diastolic), pulse rate, and body temperature | ○ | |
| Relapse assessment | Presence or absence of relapse, date of confirmation of relapse *1, date of relapse, start date of treatment of relapse, and status of treatment of relapse | ○ | |
| Hematology | WBC, WBC differential, RBC, Hb, Ht, and Plt | ○ | |
| Serum chemistry | BUN, serum creatinine, uric acid, total protein, serum albumin, AST, ALT, Na, K, Ca, P, Cl, and CRP | ○ | |
| Peripheral B-cell count | CD19-positive cells | | ○ |
| HACA | | | ○ |
| Urinalysis | Test results used to diagnose relapse (qualitative morning urine protein, quantitative morning urine protein, and quantitative morning urine creatinine) | ○ | |
| Adverse event assessment | | ○ | |

*1 This refers to the date when urinalysis is performed at the study site to confirm relapse (date of urinalysis).

## 11.10 Investigations at the time of discontinuation of study or IDEC-C2B8 treatment

The Investigator will test and observe the items listed below as soon as possible at the time of discontinuation of study or IDEC-C2B8 treatment and enter the results into the EDC. In addition, samples to be outsourced to a contract laboratory will be collected.

Table 11-11 Investigation items at the time of discontinuation of study or IDEC-C2B8 treatment

| Item | Details | Where | |
|---|---|---|---|
| | | Local | External |
| Concomitant medication/therapy | Name and route of administration of medication and/or name and purpose of therapy | ○ | |
| General symptoms | Subjective and objective findings | ○ | |
| Vital signs | Blood pressure (systolic and diastolic), pulse rate, and body temperature | ○ | |
| Relapse assessment | Presence or absence of relapse and date of relapse | ○ | |
| Adverse event assessment | | ○ | |
| HACA*1 | | | ○ |
| Blood drug concentration*1,2 | | | ○ |

*1 Measured only during the open-label period
*2 Performed within 30 minutes of the decision to discontinue if treatment is discontinued during IV infusion

## 11.11 Investigations at the time of withdrawal from the study

The investigator will test and observe the items listed below as soon as possible at the time of withdrawal from the study and enter the results into the EDC. In addition, samples to be outsourced to a contract laboratory will be collected.

Table 11-12 Investigation items at the time of withdrawal from the study

| Item | Details | Where | |
|---|---|---|---|
| | | Local | External |
| Concomitant medication/therapy | Name and route of administration of medication and/or name and purpose of therapy | ○ | |
| General symptoms | Subjective and objective findings | ○ | |
| Height and weight | | ○ | |
| Vital signs | Blood pressure (systolic and diastolic), pulse rate, and body temperature | ○ | |
| Relapse assessment | Presence or absence of relapse, date of relapse, and presence or absence of steroid sensitivity or resistance | ○ | |
| Adverse event assessment | | ○ | |
| Hematology | WBC, WBC differential, RBC, Hb, Ht, and Plt | ○ | |
| Serum chemistry | BUN, serum creatinine, uric acid, total protein, serum albumin, AST, ALT, Na, K, Ca, P, Cl, CRP, and eGFR | ○ | |
| Immunoglobulins | IgG, IgM, and IgA | ○ | |
| Peripheral B-cell count | CD19-positive cells | | ○ |
| HACA*1 | | | ○ |
| Blood drug concentration*1 | | | ○ |
| Urinalysis | Qualitative morning urine protein, qualitative morning urine occult blood, quantitative morning urine protein, and quantitative morning urine creatinine | ○ | |
| Electrocardiography/chest X-ray | | ○ | |

*1 Measured only during the open-label period

If a subject is withdrawn from the study, the investigator will enter the date of withdrawal from the study (date of decision to withdraw) and the reason for withdrawal into the EDC. Appropriate efforts should be made to identify the reason, with full respect for the rights of the subject.
Tests and AE assessment will be performed at the time of withdrawal from the study during the observation phase to ensure the safety of the subject, if applicable.
Any SAE at the time of withdrawal from the study should be appropriately treated by the Investigator, and the outcome of the event should be followed up by telephone or letter as long as possible until the end of the entire study (scheduled last observation date for the last enrolled subject).

## 11.12 Special notes for laboratory tests, blood drug concentration test, and HACA test

### 11.12.1 Peripheral B cells

Measurement will be outsourced to a contract laboratory (SRL, Inc.) (using a dedicated tube of the contract laboratory).
CD20 (19)-positive cell count = white blood cell count × differential lymphocyte percentage (%) × CD20 (19)-positive cells (%)

### 11.12.2 Blood drug concentration

Measurement of blood drug concentrations will be outsourced to a non-Japanese contract laboratory (QPS, Netherlands).
Samples will be collected by SRL, Inc.

### 11.12.3 HACA

HACA measurement will be outsourced to a non-Japanese contract laboratory (QPS, Netherlands).
Samples will be collected by SRL, Inc.

The purpose of HACA measurement is not to determine whether or not to continue IDEC-C2B8 treatment, but to investigate the relationship between HACA production and safety/efficacy such as the development of allergic symptoms or B-cell elimination.

### 11.12.4 Volume of samples (volume of blood collected) required for measurement by a contract laboratory and method for storing and sending samples

The investigator will collect, store, and send each sample as described below.

Table 11-13 Volume of samples (volume of blood collected) required for measurement by a contract laboratory and method for storing and sending samples

| Item | Sample volume mL | Method for storing and sending samples | Where | |
|---|---|---|---|---|
| | | | Local | External |
| Peripheral B cells | 5 | Samples will be stored at room temperature (using a dedicated tube of the contract laboratory) and delivered to the contract laboratory on the day of collection. | | ○ |
| Blood IDEC-C2B8 drug concentration | 2 | Serum will be separated (using a dedicated tube) and stored frozen (≤ -20°C) until delivery to the contract laboratory. | | ○ |
| HACA | 2 | Serum will be separated (using a dedicated tube) and stored frozen (≤ -20°C) until delivery to the contract laboratory. | | ○ |

# 12 Data collection

The investigator will enter case report data for each subject into the EDC.

For input, the EDC will be accessed using a pre-issued user ID and password, and entries or corrections will be made while referring to the EDC operation manual and input manual.

After confirming that there are no errors in the CRF completed, the investigator will sign it electronically in the EDC.

Any inquiry from the data center regarding CRF entries will be promptly responded to by reviewing the EDC. Any paper inquiry form issued by the data center will be appropriately stored with the medical chart, etc.

# 13 Efficacy evaluation

## 13.1 Primary endpoint

(1) Relapse-free period during the blinded observation period (Days 1 to 365 of the blinded period)

The relpase-free period, which is the primary endpoint, is defined as the time from the day of enrollment/assignment to the day of the first relapse after the start of study treatment during the observation phase (from Day 1 of the blinded period to Day 365 of the blinded/open-label period). For relapse after the day of enrollment/assignment followed by confirmation of steroid sensitivity, the relapse-free period will start on the day of re-confirmation of eligibility.

A subject who completes the observation phase without relapse will be censored on the last observation day when he/she is confirmed to be free of relapse, and a subject who is lost to follow-up or dies will be censored on the last day when he/she is confirmed to be free of relapse before he/she becomes lost to follow-up. A subject who receives prohibited therapy (10.1.2) before confirmation of recurrence will be censored on the start day of the therapy.

| | |
|---|---|
| Relapse | Any of the following conditions requiring prednisolone treatment:<br>[1] Morning urine protein dipstick ≥ 3+ (or ≥ 300 mg/dL in quantitative urine protein test) for 3 consecutive days<br>[2] Urine protein dipstick ≥ 2+ (or ≥ 100 mg/dL in quantitative urine protein test) and serum albumin ≤ 3.0 g/dL |
| Date of relapse | The first date of morning urine protein dipstick ≥ 3+ (or ≥ 300 mg/dL in quantitative urine protein test) for 3 consecutive days or date of urine protein dipstick ≥ 2+ (or ≥ 100 mg/dL in quantitative urine protein test) and serum albumin ≤ 3.0 g/dL (or date of diagnosis of relapse for the last 3 relapses before enrollment) |

## 13.2 Secondary endpoints

(1) Time to treatment failure

It is defined as the time from the day of enrollment/assignment to the day of determination of treatment failure during the blinded observation period (Days 1 to 365 of the blinded period). For relapse after the day of enrollment/assignment followed by confirmation of steroid sensitivity, the relapse-free period will start on the day of re-confirmation of eligibility.

A subject who completes the blinded observation period without being determined as treatment failure will be censored on the last day when he/she is confirmed to be free of treatment failure, and a subject who is lost to follow-up or dies without being determined as treatment failure during the blinded observation period will be censored on the last day when he/she is confirmed to be free of treatment failure before he/she becomes lost to follow-up. A subject who receives prohibited therapy (10.1.2) before confirmation of recurrence will be censored on the start day of the therapy.

(2) Total steroid dose

It is defined as the total dose of steroid administered during the blinded observation period (Days 1 to 365 of the blinded period) after the day of confirmation of the last recurrence before study treatment.

(3) Change in peripheral B-cell count during the observation phase (Days 1 to 365 of the blinded period and Days 1 to 365 of the open-label period)

1) Peripheral B-cell count (cells/μL)

Peripheral B-cell count at each time point during the observation phase (Days 1 to 365 of the blinded period and Days 1 to 365 of the open-label period)

2) Time to peripheral B-cell count normalization

It is defined as the time from the day of confirmation of peripheral B-cell depletion during the observation phase (Days 1 to 365 of the blinded period and Days 1 to 365 of the open-label period) to confirmation of peripheral B-cell count normalization (normal level ≥ 5 cells/μL). A subject who is lost to follow-up or dies will be censored on the last day when peripheral B-cell depletion is confirmed before he/she becomes lost to follow-up or dies. A subject in whom peripheral B-cell depletion is not confirmed throughout the observation phase (Days 1 to 365 of the blinded period and Days 1 to 365 of the open-label period) will be considered to have normalized on Day 0.

### 13.3 Other endpoints

(1) Relapse-free period during the open-label period

It is defined as the time from the day of confirmation of IDEC-C2B8 treatment criteria to the day of the first relapse after the start of IDEC-C2B8 treatment.

(2) Blood IDEC-C2B8 concentration

Serum IDEC-C2B8 concentration measured during the observation phase (Days 1 to 365 of the open-label period)

(3) HACA production status

Presence or absence of HACA production measured during the observation phase (Days 1 to 365 of the open-label period)

## 14 Safety evaluation

### 14.1 Adverse events

An adverse event (AE) is any untoward medical event (symptom, sign, disease, or abnormal laboratory finding) that occurs in a subject after the first dose of investigational product, whether or not considered related to the investigational product. An adverse drug reaction (ADR) is an AE for which a causal relationship to the investigational product cannot be ruled out. In this study, AEs that occur during the observation phase (from Day 1 of the blinded period to Day 365 of the blinded/open-label period) will be assessed, and relapse of nephrotic syndrome will not be handled as an AE.

Any AE should be appropriately treated by the investigator immediately.

### 14.2 Adverse event assessment and assessment criteria

The investigator will assess the occurrence of AEs throughout the study period and enter the items listed below into the EDC.

AE terms and intensity (grade) will be assessed using the Common Terminology Criteria for Adverse Events v4.0 Japanese JCOG version [CTCAE v4.0-JCOG] [corresponding to the CTCAE v4.03/MedDRA v12.0 described as MedDRA/Jv20.1 in Japanese, 12 September 2017]).

Table 14-1 Report items for adverse events

| Item | Assessment |
|---|---|
| AE term | (According to the CTCAE v4.0) |
| Start and stop dates | (and time of onset and resolution if an AE occurs on the day of administration) |
| Intensity (grade)* | Grade 1, Grade 2, Grade 3, Grade 4, or Grade 5 (according to the CTCAE v4.0) |
| Seriousness | 0. Not serious, 1. Serious |
| Action taken with investigational product | 0. None, 1. Slowed, 2. Interrupted, 3. Discontinued, 4. Dose reduced, 5. Other (specify) |
| Other treatment | 0. No, 1. Yes (specify) |
| Outcome | 1. Resolved, 2. Resolving, 3. Resolved with sequelae, 4. Not resolved, 5. Died, 6. Unknown |
| Relationship to investigational product | 1. Not related, 2. Related (including unknown) |

* The intensity (grade) of AEs means the severity. Grades 1 to 5 defined by the CTCAE v4.0 are presented below.

Grade 1: Mild (intervention not indicated; asymptomatic abnormal imaging/laboratory test)

Grade 2: Moderate (minimal, local or noninvasive intervention indicated)

Grade 3: Severe (significant symptoms requiring hospitalization or invasive intervention/IVR/transfusion/therapeutic endoscopy/surgery)

Grade 4: Life-threatening or disabling/incapacitating adverse events (e.g., acute life-threatening metabolic/cardiovascular complications; intensive care or urgent intervention [e.g., emergency IVR/therapeutic endoscopy/surgery] indicated)

Grade 5: Death due to an adverse event

### 14.2.1 Intensity (grade) of major adverse events

Major AEs excerpted from the CTCAE v4.0 are presented below.

| Grade | 1<br>Mild | 2<br>Moderate | 3<br>Severe | 4<br>Life-threatening or disabling | 5<br>Death |
|---|---|---|---|---|---|
| Hypertension | Prehypertension (systolic BP 120 - 139 mmHg or diastolic BP 80 - 89 mmHg) | Stage 1 hypertension (systolic BP 140 - 159 mmHg or diastolic BP 90 - 99 mmHg); medical intervention indicated; recurrent or persistent (>=24 hrs); symptomatic increase by >20 mmHg (diastolic) or to >140/90 mmHg if previously WNL; monotherapy indicated<br>Pediatric: recurrent or persistent (>=24 hrs) BP >ULN; monotherapy indicated | Stage 2 hypertension (systolic BP >= 160 mm Hg or diastolic BP >=100 mm Hg); medical intervention indicated; more than one drug or more intensive therapy than previously used indicated<br>Pediatric: Same as adult | Life-threatening consequences (e.g., malignant hypertension, transient or permanent neurologic deficit, hypertensive crisis); urgent intervention indicated<br>Pediatric: Same as adult | Death |
| Hypotension | Asymptomatic; intervention not indicated | Non-urgent medical intervention indicated | Medical intervention or hospitalization indicated | Life-threatening consequences; urgent intervention indicated | Death |
| Fever | 38.0 - 39.0 degrees C | >39.0 - 40.0 degrees C | >40.0 degrees C for <=24 hrs | >40.0 degrees C for >24 hrs | Death |
| Chills | Mild sensation of cold; shivering; chattering of teeth | Moderate tremor of the entire body; narcotics indicated | Severe or prolonged, not responsive to narcotics | - | - |
| Pruritus | Mild or localized; topical intervention indicated | Intense or widespread; intermittent; skin changes from scratching (e.g., edema, papulation, excoriations, lichenification, oozing/crusts); oral intervention indicated; limiting instrumental ADL | Intense or widespread; constant; limiting self care ADL or sleep; oral corticosteroid or immunosuppressive therapy indicated | - | - |
| Urticaria | Urticarial lesions covering <10% BSA; topical intervention indicated | Urticarial lesions covering 10 - 30% BSA; oral intervention indicated | Urticarial lesions covering >30% BSA; IV intervention indicated | - | - |
| Adrenal insufficiency | Asymptomatic; clinical or diagnostic observations only; intervention not indicated | Moderate symptoms; medical intervention indicated | Severe symptoms; hospitalization indicated | Life-threatening consequences; urgent intervention indicated | Death |
| Nausea | Loss of appetite without alteration in eating habits | Oral intake decreased without significant weight loss, dehydration or malnutrition | Inadequate oral caloric or fluid intake; tube feeding, TPN, or hospitalization indicated | - | - |
| Dysgeusia | Altered taste but no change in diet | Altered taste with change in diet (e.g., oral supplements); noxious or unpleasant taste; loss of taste | - | - | - |
| Infection | Mild | Moderate | Severe | Life-threatening [illegible] | Death |
| Acidosis (metabolic or respiratory) | pH <normal, but >=7.3 | - | pH <7.3 | Life-threatening consequences | Death |
| SGPT | >ULN - 3.0 × ULN** | >3.0 - 5.0 x ULN** | >5.0 - 20.0 × ULN** | >20.0 × ULN** | - |
| Amylase | >ULN - 1.25 × ULN** | >1.5 - 2.0 × ULN** | >2.0 - 5.0 × ULN** | >5.0 × ULN** | - |
| SGOT | >ULN - 3.0 × ULN** | >3.0 - 5.0 x ULN** | >5.0 - 20.0 × ULN** | >20.0 × ULN** | - |
| Cataract | Asymptomatic; clinical or diagnostic observations only; intervention not indicated | Symptomatic; moderate decrease in visual acuity (20/40 or better) | Symptomatic with marked decrease in visual acuity (worse than 20/40 but better than 20/200); operative intervention indicated (e.g., cataract surgery) | Blindness (20/200 or worse) in the affected eye | - |
| Glaucoma | Elevated intraocular pressure (EIOP) with single topical agent for intervention; no visual field deficit | EIOP causing early visual field deficits; multiple topical or oral agents indicated; limiting instrumental ADL | EIOP causing marked visual field deficits (e.g., involving both superior and inferior visual fields); operative intervention indicated; limiting self care ADL | Blindness (20/200 or worse) in the affected eye | - |
| Bronchospasm | Mild symptoms; intervention not indicated | Symptomatic; medical intervention indicated; limiting instrumental ADL | Limiting self care ADL; oxygen saturation decreased | Life-threatening respiratory or hemodynamic compromise; intubation or urgent | Death |

| Grade | 1<br>Mild | 2<br>Moderate | 3<br>Severe | 4<br>Life-threatening or disabling | 5<br>Death |
|---|---|---|---|---|---|
| | | | | intervention indicated | |
| Wheezing | Detectable airway noise with minimal symptoms | Moderate symptoms; medical intervention indicated; limiting instrumental ADL | Severe respiratory symptoms; limiting self care ADL; oxygen therapy or hospitalization indicated | Life-threatening consequences; urgent intervention indicated | |
| Cough | Symptomatic; nonprescription medication indicated | Moderate symptoms; medical intervention indicated; limiting instrumental ADL | Severe symptoms; limiting self care ADL | - | - |
| Laryngeal edema | Asymptomatic; clinical or diagnostic observations only; intervention not indicated | Symptomatic; medical intervention indicated (e.g., dexamethasone, epinephrine, antihistamines) | Stridor; respiratory distress; hospitalization indicated | Life-threatening airway compromise; urgent intervention indicated (e.g., tracheotomy or intubation) | Death |

### 14.2.2 Seriousness

A serious adverse event (SAE) is an AE that

(1) results in death,
(2) is life-threatening,
(3) requires inpatient hospitalization or prolongation of existing hospitalization (excluding inpatient hospitalization or prolongation of existing hospitalization for re-examination or follow-up),
(4) results in disability/incapacity (dysfunction that interferes with activities of daily living),
(5) may result in disability/incapacity,
(6) is serious according to (1) to (5),
(7) or is a congenital anomaly/birth defect.

Other situations, i.e., significant events that do not immediately threaten life or result in death or hospitalization but may jeopardize the subject or require intervention to prevent the above-mentioned outcomes, should be assessed on medical and scientific grounds as to whether or not expedited reporting is necessary and should normally be considered serious (e.g., bronchospasm requiring intensive care, blood dyscrasias or convulsions that do not result in hospitalization, drug dependence or abuse).

### 14.2.3 Relationship to investigational product

The relationship between an AE and the study drug or IDEC-C2B8 will be determined as (1) or (2) below (replace the term "study drug" with "IDEC-C2B8" in the open-label period).

(1) At least possibly related to the study drug

A causal relationship of the event to the study drug cannot be ruled out or the event cannot be explained by any cause other than the study drug.

(2) Not related to the study drug

It is clear that the event is due to reasons other than the study drug, such as 1) to 5) below.

1) Physiological variation
2) Due to primary disease

3) Due to comorbidity (specify comorbidity)
4) Due to medication other than the study drug (specify medication)
5) Other (specify reason)

## 14.3 Follow-up of adverse events

The investigator will follow up any AE occurring during the observation phase (from Day 1 of the blinded period to Day 365 of the blinded/open-label period) until it resolves or returns to the same grade as before the start of study treatment. Follow-up may be terminated when the investigator considers further follow-up unnecessary.

## 14.4 Expedited reporting of adverse events

### 14.4.1 When a serious adverse event occurs

(1) If an AE is considered serious, the investigator will complete the "Serious Adverse Event Report" and report the event to the head of the study site as soon as possible and to the clinical trial steering committee, regardless of causality.

(2) The clinical trial steering committee will review the SAE received from the investigator and provide the investigators at the other study sites with information of this AE.

(3) The investigator at each study site will review the report received from the clinical trial steering committee, discuss with the clinical trial steering committee as needed, and report his/her opinion (including the necessity to report to the Minister of Health, Labour and Welfare) to the clinical trial steering committee.

(4) The clinical trial steering committee will report the SAE received to the investigational product provider. If reporting to the Minister of Health, Labour and Welfare is considered necessary, an initial report should be submitted to the investigational product provider promptly.

(5) If the investigator considers it necessary to report to the Minister of Health, Labour and Welfare, the clinical trial steering committee will complete Attachment Forms 7 and 8*[1] of Case Report Form for Adverse Reaction to Investigational Product/Infection (hereinafter referred to as "Forms 7 and 8") and Attachment 1 Summary Form for Reporting Clinical Trial Adverse Drug Reaction*[2] (hereinafter referred to as "Summary Form") and report the event to the Minister of Health, Labour and Welfare. The clinical trial steering committee will retain copies of "Forms 7 and 8" and "Summary Form."

*1 Specified in Reporting of Adverse Drug Reactions in Clinical Trials to the Pharmaceuticals and Medical Devices Agency (PFSB Notification No. 1215003 dated 15 December 2005 revised from PFSB Notification No. 0330001 dated 30 March 2004)

*2 Specified in Revision of "Reporting of Adverse Drug Reactions in Clinical Trials by Sponsor-investigators after the Establishment of the Pharmaceuticals and Medical Devices Agency" (PFSB/ELD Notification No. 1025005 dated 25 October 2005)

(6) The deadline for reporting to the Minister of Health, Labour and Welfare is stipulated in Article 273 of the Enforcement Regulations of the Pharmaceutical and Medical Device Act and other regulations as described below. The reporting deadline is relative to the day when the investigator becomes aware of the onset of the event.

| | |
|---|---|
| Report within 7 days | Unknown event corresponding to (1) or (2) in 14.2.2 |
| Report within 15 days | Known event corresponding to (1) or (2) and unknown event corresponding to (3), (4), (5), (6), or (7) in 14.2.2 |

An unknown event is defined as an event whose occurrence or trend of occurrence such as number, incidence, or conditions of occurrence cannot be predicted from the investigator's brochure (including ADRs already reported to each investigator and the Minister of Health, Labour and Welfare), and a known event is defined as an event that can be predicted from the investigator's brochure.

(7) If an SAE occurring at another study site is reported to the Minister of Health, Labour and Welfare, the investigator will report the contents of Forms 7 and 8 received from the clinical trial steering committee to the head of the study site as soon as possible.

(8) The investigator at the study site where the event occurs will report any additional information of the AE to the head of the study site as soon as possible and to the clinical trial steering committee. The clinical trial steering committee will communicate this additional information to the investigators at the other study sites and the investigational product provider. Additional information that, in the opinion of the investigator, should be reported as addition to or withdrawal of the previous ADR report or as a new event will be handled in accordance with (5).

### 14.4.2 When other events (pregnancy and overdose, medication error, or misuse/abuse of investigational product) are identified

The investigator will report any event as described in (1) to (4) below to the clinical trial steering committee as soon as possible. Form 1 or 2 in the "Procedures for Handling of Safety Information" will be used for reporting. The clinical trial steering committee will review the reported event as described in (1) to (4) and report it to the investigational product provider.

For the reported event as described in (1) to (4), the investigational product provider will report safety information, etc. to the non-Japanese manufacturer of IDEC-C2B8 in accordance with the agreement with the manufacturer.

(1) Pregnancy

A subject or the partner of a male subject becomes pregnant.

(2) Overdose

The investigational product is administered at a dose higher than specified in the protocol.

E.g., administration at a dose of > 375 mg/m$^2$ or 500 mg rather than at a dose of 375 mg/m$^2$ (up to 500 mg)

(3) Medication error

The investigational product is administered via a route not specified in the protocol, a different drug is dispensed, or a wrong drug is administered. This includes an attempted error in which the drug was not actually administered to the subject.

(4) Misuse/abuse

The investigational product is inappropriately used by intentionally using a regimen not specified in the protocol.

### 14.4.3 Handling of safety information provided by the investigational product provider

After reviewing the information described in (1) to (4) provided by the investigational product provider, the clinical trial steering committee will promptly provide this safety information to the investigator at the study site. The investigator will report the safety information obtained from the clinical trial steering committee to the head of the study site as soon as possible.

(1) Information of unknown serious ADRs associated with the investigational product provided by the investigational product provider
(2) Information of study reports and domestic/overseas action reports regarding the investigational product provided by the investigational product provider
(3) Information of development safety update report (annual report) for the investigational product provided by the investigational product provider
(4) Information of revision of the investigator's brochure and package insert for the investigational product provided by the investigational product provider

## 14.5 Major expected adverse reactions to the investigational product

Major expected adverse reactions to the investigational product are excerpted below from the package insert for the formulation of rituximab (genetical recombination) (Rituxan®). See the package insert for details.

(1) Clinically significant adverse reactions

1) Infusion reaction (incidence unknown)

Infusion reaction (symptoms: fever, chills, nausea, headache, pain, pruritus, rash, cough, feeling of collapse, angioedema, etc.) that often occurs during infusion of Rituxan or within 24 hours after the start of infusion has been reported in approximately 90% of treated patients, and these symptoms are usually minimal to moderate in severity and occur mainly during or after the first dose. In addition, serious adverse reactions such as anaphylaxis, pulmonary disorders, and cardiac disorders (e.g., hypotension, angioedema, hypoxemia, bronchospasm, pneumonia [including interstitial pneumonia and allergic pneumonia], obliterative bronchiolitis, pulmonary infiltration, acute respiratory distress syndrome, myocardial infarction, ventricular fibrillation, cardiogenic shock) may occur. Serious infusion reaction has also been reported in patients pretreated with antihistamines, antipyretic analgesics, and/or corticosteroids. If an abnormality occurs, administration should be immediately discontinued, appropriate measures (e.g., oxygen inhalation, administration of vasopressors, bronchodilators, corticosteroids, antipyretic analgesics, and/or antihistamines) should be taken, and the patient should be closely monitored until the symptoms resolve.

2) Tumor lysis syndrome (incidence unknown)

If an abnormality occurs, administration should be immediately discontinued, appropriate measures (e.g., administration of physiological saline, antihyperuricemics, etc., dialysis) should be taken, and the patient

should be closely monitored until the symptoms resolve.

3) Fulminant hepatitis or exacerbation of hepatitis due to reactivation of hepatitis B virus (incidence unknown)

   Hepatic failure secondary to fulminant hepatitis or exacerbation of hepatitis due to reactivation of hepatitis B virus may occur and has been reported to be fatal in some cases. If an abnormality occurs, appropriate measures such as administration of antivirals should be immediately taken.

4) Hepatic function disorder and jaundice (incidence unknown)

   Hepatic function disorder and/or jaundice with abnormal liver function tests such as increased AST (10.0%), increased ALT (10.3%), increased Al-P (4.0%), and increased total bilirubin (4.0%) may occur.

5) Mucocutaneous symptoms (incidence unknown)

   Oculomucocutaneous syndrome (Stevens-Johnson syndrome), toxic epidermal necrolysis (TEN), pemphigus-like symptoms, lichenoid dermatitis, and vesiculobullous dermatitis have been reported with fatal outcome in some cases.

6) Cytopenia

   Pancytopenia (incidence unknown), leukopenia (47.5%), neutropenia (45.8%), agranulocytosis (incidence unknown), and thrombocytopenia (14.3%) may occur, and serious cytopenia has been reported. It has been reported that neutropenia occurred more than 4 weeks after the last dose of Rituxan.

7) Infections (43.0%)

   Since serious bacterial, fungal, or viral infections (e.g., sepsis, pneumonia) may occur, patients should be closely monitored during and after treatment with Rituxan.

8) Progressive multifocal leukoencephalopathy (PML) (incidence unknown)

   Patients should be closely monitored during and after treatment with Rituxan. If symptoms such as disturbed consciousness, cognitive disorder, paralytic symptom (hemiplegia or quadriplegia), or language disorder occur, diagnostic MRI and cerebrospinal fluid examination should be performed, administration should be discontinued, and appropriate measures should be taken.

9) Interstitial pneumonia (incidence unknown)

10) Cardiac disorders (12.0%)

    Ventricular or atrial arrhythmia, angina pectoris, and myocardial infarction have been reported.

11) Renal disorders (incidence unknown)

    Renal disorders such as increased serum creatinine (1.0%) or increased BUN (2.8%) may occur, and renal disorders requiring dialysis have been reported. If decreased urine output or increased serum creatinine or BUN is observed, administration should be discontinued and appropriate measures should be taken.

12) Gastrointestinal perforation/obstruction (incidence unknown)

    If an abnormality occurs, X-ray or CT scan should be performed immediately to determine the site of bleeding and the presence or absence of findings of perforation/obstruction, and appropriate measures should be taken.

13) Decreased blood pressure (6.8%)

Blood pressure may decrease transiently.

14) Cranial nerve symptoms such as posterior reversible encephalopathy syndrome (incidence unknown)

Posterior reversible encephalopathy syndrome (symptoms: seizure, headache, psychiatric symptoms, visual disturbance, hypertension, etc.) may occur. In addition, visual and hearing impairments such as blindness and deafness, and cranial nerve disorders such as sensory disturbance and facial paralysis have been reported within 6 months after the end of treatment with Rituxan.

(2) Other adverse reactions

| | ≥ 5% | < 5% | Incidence unknown |
|---|---|---|---|
| Respiratory | Laryngopharyngitis (28.8%), rhinitis (19.3%), oropharyngeal discomfort (12.5%), cough, respiratory disorder | Wheezing, epistaxis | |
| Circulatory | Increased blood pressure (14.3%), tachycardia, flushing | Palpitations, vasodilatation, bradycardia, peripheral ischemia | |
| Gastrointestinal | Nausea/vomiting (18.0%), stomatitis, abdominal pain, anorexia, diarrhea | Dry mouth, constipation, tenesmus | |
| Hypersensitivity | Pyrexia (36.5%), chills (16.3%), pruritus (16.3%), rash (15.8%), hot flush (11.8%) | Arthralgia, urticaria, myalgia, influenza-like symptoms | Serum sickness |
| General | Pain (30.5%), malaise (18.8%), feeling of collapse (18.0%), headache (16.0%), heavy sweating, edema | Chest pain, increased weight, asthenia | |
| Neuropsychiatric | Dysesthesia | Numbness, dizziness, insomnia | |
| Blood/coagulation | Anemia (21.3%) | Eosinophilia | Increased fibrin degradation products [FDP, D-dimer], thrombocytosis |
| Kidney | Electrolyte abnormality | Increased BUN, increased creatinine | |
| Liver | Increased ALT (10.3%), increased AST (10.0%) | Increased Al-P, increased total bilirubin | |
| Other | Increased CRP (18.7%), increased LDH (11.3%) | Decreased total protein, herpes zoster, increased uric acid, conjunctivitis, asteatotic eczema, administration site reaction (e.g., pain, swelling), decreased albumin, hiccups | Muscle twitching |

Note) The incidences of ADRs were tabulated based on the results of a Japanese phase II clinical study in B-cell non-Hodgkin's lymphoma, a Japanese phase II clinical study in chronic lymphocytic leukemia, a Japanese phase III clinical study in refractory nephrotic syndrome, a Japanese phase II clinical study in systemic scleroderma, a Japanese phase II clinical study in refractory pemphigus vulgaris and pemphigus foliaceus, and open-label clinical studies in ABO-incompatible kidney transplantation.

# 15 Statistical analysis

## 15.1 Analysis population

The analysis sets are defined as described below. Details of case handling will be specified in a statistical analysis plan by the statistical analysis manager before data lock. The full analysis set will be used to analyze the efficacy endpoints in the final analysis, and analyses will also be performed on the per protocol set for reference. The safety analysis set will be used to analyze the safety endpoints.
No interim analysis will be performed in this study.

(1) Full analysis set (FAS)
A subset of subjects who received at least one dose of study drug during the blinded period and had data collected for the primary post-treatment assessment

(2) Per protocol set (PPS)
A subset of subjects in the FAS, excluding those who violated the protocol during the blinded period

(3) Safety analysis set (SAS)
A subset of subjects who received at least one dose of study drug during the blinded period

(4) Unblinded analysis set (UAS)
A subset of subjects who entered the open-label period and received at least one dose of IDEC-C2B8

## 15.2 Target sample size and its rationale

According to Kaplan-Meier plot of relapse-free period in a non-Japanese open-label randomized controlled study in which pediatric patients with uncomplicated steroid-dependent nephrotic syndrome received rituximab at a single dose of 375 mg/m$^2$ in addition to steroids,[16)] the 1-year relapse-free rate was 0.66 in the rituximab group and 0 in the steroid monotherapy group. Assuming an exponential distribution and a 1-year relapse-free rate of 0.01 in the steroid monotherapy group based on the results of the above study, the hazard ratio is 0.09, with hazard of 0.42 in the rituximab group and 4.60 in the steroid monotherapy group. The number of subjects required to verify the superiority over 12 months, the duration of participation of each subject in the study, at a two-sided significance level of 5% and a power of 80% is estimated to be 13 per group. Assuming that the 1-year relapse-free rate in the rituximab group is 0.50 and 0.40, the necessary number of subjects calculated under the same conditions is 15 and 17 per group, respectively.
According to Kaplan-Meier plot of relapse-free period in a Japanese phase III placebo-controlled randomized study in which patients with complicated nephrotic syndrome (frequently relapsing or steroid-dependent) received rituximab at a dose of 375 mg/m$^2$ every week for 4 doses,[9)] the 1-year relapse-free rate was 0.44 in the rituximab group and 0.04 in the placebo group. Assuming an exponential distribution based on the results of this study, the hazard ratio is 0.255, with hazard of 0.82 in the rituximab group and 3.21 in the placebo group. The number of subjects required to verify the superiority over 12 months, the duration of participation of each subject in the study, at a two-sided significance level of 5% and a power of 80% is estimated to be 24 per group.
The planned study population for the present study is patients with childhood-onset nephrotic syndrome diagnosed as uncomplicated frequently relapsing or steroid-dependent. Although the disease may become

complicated in some of these subjects, it may be more appropriate to refer to the results of the above non-Japanese study in pediatric patients with uncomplicated steroid-dependent nephrotic syndrome.
Based on the above, the target sample size is determined to be 20 treated subjects per group, taking into account the uncertainty.

## 15.3 Analysis methods

Details of statistical analysis methods will be specified in a statistical analysis plan.

### 15.3.1 Primary analysis and assessment criteria

The objective of the primary analysis in this study is to test whether active treatment is significantly superior to placebo in terms of relapse-free period, which is the primary endpoint. The null hypothesis in the primary analysis that "the relapse-free period is equal between the two groups" will be tested by stratified log-rank test using the randomization factor, with the FAS defined in 15.1 as the primary analysis set. Since statistical significance is not of concern when active treatment is inferior to placebo, the test will be one-sided. A one-sided significance level of 2.5% will be used. If statistically significant superiority of active treatment over placebo is shown, it will be concluded that IDEC-C2B8 (rituximab) is a useful treatment. Analysis results in the PPS will be used only for reference.
Relapse-free curve, median relapse-free period, and relapse-free rate at each time point will be estimated by the Kaplan-Meier method, and 95% confidence intervals (CIs) will be calculated using the Greenwood formula. To estimate the treatment effect, the hazard ratio of treatment effect between the groups and its 95% CI will be calculated using the Cox proportional hazards model with the randomization factor as a covariate.
For reference, subgroup analysis will be performed on the FAS, with each level of the randomization factor for analysis (frequent relapse and steroid dependence) as a subgroup. In the subgroup analysis of relapse-free period, which is the primary endpoint, relapse-free curve, median relapse-free period, and relapse-free rate at each time point will be estimated by the Kaplan-Meier method for each subgroup, and between-group comparison will be performed by the log-rank test. In addition, the hazard ratio of treatment effect between the groups and its 95% CI will be estimated for each subgroup using the Cox proportional hazards model.

### 15.3.2 Analyses of secondary endpoints (efficacy endpoints)

Analyses of secondary endpoints will not be adjusted for multiplicity. A two-sided significance level of 5% will be used for all tests.

(1) Time to treatment failure

For the time to treatment failure, an event chart will be plotted for each group to estimate non-treatment failure rate curve and non-treatment failure rate at each time point by the Kaplan-Meier method, and between-group comparison will be performed by stratified log-rank test using the randomization factor. In addition, the hazard ratio of treatment effect between the groups and its 95% CI will be estimated using the Cox proportional hazards model with the randomization factor as a covariate.

(2) Total steroid dose

For the total dose per day up to the end of the blinded period, the total dose per day from the day of confirmation of the last relapse before study treatment to the blinded observation period will be calculated and the difference in the mean will be compared between the groups by analysis of covariance with the randomization factor as a covariate. In addition, the between-group difference in the mean calculated using a model including the randomization factor and its 95% CI will be estimated.

(3) Peripheral B-cell count

For the time course of peripheral B-cell count, time course of peripheral B-cell count will be plotted for each subject, and the proportion of subjects with peripheral B-cell count normalization and its 95% CI will be estimated for each group at each time point of the blinded period. In addition, between-group comparison will be performed by Fisher's exact test at each time point of the blinded period.

(4) Time to peripheral B-cell count normalization

For the time to peripheral B-cell count normalization, cumulative incidence curve and median time to peripheral B-cell count normalization and its 95% CI will be estimated by the Kaplan-Meier method for each group in the blinded period and for IDEC-C2B8-treated subjects in the open-label period.

(5) Relationship between the presence or absence of peripheral B-cell count normalization and the first relapse

To investigate the relationship between the presence or absence of peripheral B-cell count normalization and the first relapse, a 2-by-2 contingency table of the presence or absence of peripheral B-cell count normalization versus the presence or absence of the first relapse at each time point will be constructed for Fisher's exact test, and the conditional odds ratio and its 95% CI based on the hypergeometric distribution will be estimated.

(6) Relationship between the presence or absence of peripheral B-cell count normalization and the presence or absence of AEs

To investigate the relationship between the presence or absence of peripheral B-cell count normalization and the presence or absence of AEs, a 2-by-2 contingency table of the presence or absence of peripheral B-cell count normalization versus the presence or absence of AEs at each time point will be constructed for Fisher's exact test, and the conditional odds ratio and its 95% CI based on the hypergeometric distribution will be estimated.

AEs in this analysis will be classified into two categories: all AEs and infections requiring treatment.

### 15.3.3 Sensitivity analysis

Details will be specified in a statistical analysis plan.

(1) Sensitivity analysis of the effect of using the day of first study drug administration (Day 1 of the blinded period) as the starting point

The primary endpoint (3.1.1 and 13.1) and the time to treatment failure, which is a secondary endpoint (3.1.2 and 13.2), will be analyzed with Day 1 of the blinded period as the starting point in the same manner.

(2) Sensitivity analysis of the effect of using the day of enrollment/assignment as the starting point

The primary endpoint (3.1.1 and 13.1) will be analyzed in all subjects (including those who relapse after

the day of enrollment/assignment) with the day of enrollment/assignment as the starting point.

(3) Sensitivity analysis of the effect of changing the definition of relapse

The relapse-free period, which is the primary endpoint, will be analyzed as described in 15.3.1 by handling (1), (2), and (3) listed in 10.1.2, "Prohibited medications and therapies" as events if administered.

#### 15.3.4 Analyses of other endpoints

The following will be analyzed in subjects who enter the open-label period (IDEC-C2B8 treatment):

(1) Relapse-free period during the open-label period

For the relapse-free period from the day of confirmation of IDEC-C2B8 treatment criteria, relapse-free curve and median relapse-free period will be estimated by the Kaplan-Meier method.

(2) Time course of blood IDEC-C2B8 concentration

For the time course of serum IDEC-C2B8 concentration, the mean ± standard deviation of serum IDEC-C2B8 concentration after the start of IDEC-C2B8 administration will be plotted for all subjects treated with IDEC-C2B8. In addition, the pharmacokinetic parameters Cmax (ng/mL), T1/2 (h), mean residence time (h), AUC (μg•h/mL), clearance (L/h), and volume of distribution (L) will be estimated, and values in individual subjects as well as a summary of number of subjects, mean, standard deviation, minimum, median, and maximum will be presented.

(3) Change in proportion of subjects with HACA production

For HACA production, the proportion of subjects with HACA production and its 95% CI will be estimated at each time point.

(4) Relationship between HACA production and relapse or AEs

To investigate the relationship between HACA production and relapse or AEs, a 2-by-2 contingency table of the presence or absence of HACA production versus the presence or absence of relapse at each time point will be constructed for Fisher's exact test, and the conditional odds ratio and its 95% CI based on the hypergeometric distribution will be estimated.

A similar analysis will be performed for the presence or absence of HACA production versus the presence or absence of AEs at each time point. AEs in this analysis will be classified into two categories: all AEs and infections requiring treatment.

#### 15.3.5 Analysis of safety

Occurrence of AEs and ADRs, laboratory parameters, and vital signs will be tabulated for the blinded period (including the switching period) and the open-label period. MedDRA terms will be used as AE terms.

(1) Occurrence of AEs and ADRs

The number and proportion of subjects with AE will be presented for each treatment group and tabulated by system organ class, AE (symptom and finding), and grade. For each AE observed, the worst grade during the study will be used as the severity (grade).

The number and proportion of subjects with SAE will also be tabulated and listed for each group. In addition, the number and proportion of subjects will be tabulated by AE leading to discontinuation during the study

treatment period (by individual AEs and system organ class) for each treatment group.

In addition, the proportion of subjects and incidence will be calculated for the period when blinding is maintained (until the day of early key opening or the last day of the blinded observation period). Between-group comparison will be performed by Fisher's exact test and Poisson distribution test.

ADRs will be analyzed in the same manner.

(2) Laboratory parameters and vital signs

1) Tabulation of laboratory parameter (hematology, serum chemistry, and urinalysis) and vital sign measurements

Summary statistics will be calculated for each treatment group at each time point.

2) Tabulation of abnormal laboratory values (hematology, serum chemistry, and urinalysis)

For each abnormal laboratory value, the incidence will be tabulated for each treatment group at each time point.

### 15.3.6 Analysis using a dataset including data from subjects who enter the open-label period (IDEC-C2B8 treatment)

The following will be analyzed using a dataset created by combining data from subjects who enter the open-label period and receive IDEC-C2B8 and data from subjects who receive IDEC-C2B8 during the blinded period:

(1) Relapse-free period

For the relapse-free period, event chart and Kaplan-Meier relapse-free curve will be plotted to estimate the relapse-free rate and its 95% CI at each time point. The relapse-free period in the open-label period will start on the day of confirmation of IDEC-C2B8 treatment criteria.

(2) Time to peripheral B-cell count normalization

For the time to peripheral B-cell count normalization, cumulative incidence curve will be estimated by the Kaplan-Meier method. In addition, median time to normalization and its 95% CI will be estimated.

(3) AEs and ADRs

The number and proportion of subjects with AE or ADR will be presented and tabulated by system organ class, AE (symptom and finding), and grade. For each AE or ADR observed, the worst grade during the observation phase will be used as the severity (grade).

The number and proportion of subjects with SAE will also be tabulated and listed. In addition, the number and proportion of subjects will be tabulated by AE or ADR leading to discontinuation during the study treatment period (by individual AEs and system organ class).

(4) Laboratory parameters (hematology, serum chemistry, and urinalysis) and vital signs

For laboratory parameters (hematology, serum chemistry, and urinalysis) and vital signs, summary statistics will be calculated at each time point. For each abnormal laboratory value (hematology, serum chemistry, and urinalysis), the incidence will be tabulated at each time point.

# 16 Direct access to source documents, etc.

## 16.1 Acceptance of direct access

The head of the study site and the investigator will accept inspections by monitors, auditors, the IRB, and regulatory authorities and will provide direct access to all study-related records, including source documents listed below, upon request.

The monitor will directly access study-related records, including source documents, at all study sites to confirm that the case report form (CRF) entries are accurate, complete, and consistent with the source documents. In addition, essential documents will be directly accessed at all study sites to confirm that all study-related records to be retained at the study site are accurately and completely prepared and retained.

## 16.2 Definition of source document

The following documents will serve as source documents in this study:

1) Informed consent form
2) Medical records (including examination slips)
3) Nursing record
4) Patient diary
5) Laboratory data
6) Drug accountability log, drug supply form, and drug collection form
7) List of subject identification codes
8) Other records from which the CRF was completed

The following items will be recorded in the medical record:

1) Subject demographics (concomitant disease, medical history, and disease history)
2) Presence or absence of use of other drugs
3) Presence or absence of subjective or objective symptoms
4) Presence or absence of laboratory tests and abnormal changes
5) Presence or absence, severity, and seriousness of AE, status of study or IDEC-C2B8 treatment, causal relationship to study drug or IDEC-C2B8, and comments
6) Presence or absence of discontinuation of study or IDEC-C2B8 treatment and comments
7) Assessment
8) Investigator's comments

# 17 Quality control and quality assurance

## 17.1 Data quality control

The investigator will perform monitoring and quality control according to the progress of the study to confirm that the study is being conducted in compliance with the GCP Ministerial Ordinance and the protocol and to ensure the accuracy, completeness, and reliability of data.

Before the start of the study, the investigator and the monitor will explain the protocol and the procedure for completing the CRF to the subinvestigator, etc. In addition, the investigational product manager will be informed of how to manage the investigational product. During the conduct of the study, the monitor will make periodic visits to the study site to confirm the status of compliance with the protocol, consistency between the CRF and source documents, and essential documents.

### 17.2 Audit

The auditor will perform audits as part of quality assurance activities to assess whether or not the study is being conducted in compliance with the GCP Ministerial Ordinance and the protocol, independently and separately from routine monitoring and study quality control operations.

### 17.3 Provision of new information

If important new information becomes available that may be relevant to a subject's consent (i.e., information on the occurrence of disease, disability, or death suspected to be caused by the investigational product, information on the occurrence of infection, or other information on the quality, efficacy, and safety of the investigational product), the investigator will promptly revise the information sheet/informed consent form based on this information and obtain approval from the IRB. In addition, the investigator will inform a participating subject and legal representative of the revision, confirm his/her willingness to remain in the study, and obtain consent again using the revised informed consent form and information sheet. The procedures are the same as those described in 7.2.1.

## 18 Ethics

### 18.1 Ethical conduct of the study

This study will be conducted in accordance with the principles of the Declaration of Helsinki and in compliance with Article 14, Paragraph 3 and Article 80-2 of the Pharmaceutical and Medical Device Act, the Ministerial Ordinance on Good Clinical Practice for Drugs (MHW Ordinance No. 28 dated 27 March 1997) and its amendments, operation notifications, and this protocol.

### 18.2 Explanation and informed consent

During the informed consent process, the investigator will hand over the information sheet describing the following explanatory matters and fully explain them to obtain written informed consent to participate in the study:

#### 18.2.1 Explanatory matters

(1) That a clinical trial involves research
(2) Objective of the study
(3) Study methods

(4) Planned duration of the subject's participation in the study
(5) Number of subjects planned to participate in the study
(6) Anticipated clinical benefits or risks to the subject's physical or mental health or inconvenience
(7) Availability of other treatments and anticipated significant benefits and risks associated with these treatments
(8) Compensation and treatment available to the subject in the event of a study-related injury
(9) That participation in the study is voluntary and that the subject may refuse or withdraw from the study at any time; that refusal or withdrawal will not penalize the subject or deprive the subject of any benefit that would accrue if the subject does not participate in the study
(10) That the subject will be informed in a timely manner if information becomes available that may affect the subject's willingness to remain in the study
(11) Condition or reason for terminating participation in the study
(12) That monitors, auditors, the IRB at the study site, and regulatory authorities will be given access to the source medical records; that the privacy of the subject will be protected during this process; and that the subject is authorizing access by signing or sealing the informed consent form
(13) That the privacy of the subject will be protected even if the results of the study are published
(14) Details of any expenses that the subject will incur
(15) Details of money to be paid to the subject (e.g., arrangements for calculation of payment)
(16) Name and contact information of the investigator
(17) Person(s) at the study site to contact for further information regarding the study and the subject's rights or to notify or contact in the event of a study-related injury
(18) Subject's responsibilities
(19) Matters related to the IRB, including the type (name and founder) of IRB that will review and discuss the suitability of the study and matters that will be reviewed and discussed by each IRB

## 18.3 Institutional review board (IRB)

The investigator will obtain approval from the IRB prior to conducting the study. Any amendment to the protocol must also be approved by the IRB. Any information that may affect the safety of the subject or the conduct of the study will be promptly reported to the other investigators and the head of the study site, and approval for continuing the study will be obtained from the IRB.

## 18.4 Subject confidentiality

Subject confidentiality will be protected as follows:

Consideration will be given to the confidentiality of the subject when completing and handling the CRF.

Subjects will be identified by unique subject identification codes.

The IRB, monitors, auditors, and regulatory authorities will maintain subject confidentiality during direct access to source documents.

Consideration will also be given to the confidentiality when the results of the study are published or when the

investigational product provider submits an application for approval.

# 19 Data handling and record retention

## 19.1 Completion and reporting of the CRF

After completing observations and investigations in individual subjects, the investigator will promptly enter case report data for each subject into the EDC.

For input, the EDC will be accessed using a pre-issued user ID and password, and entries or corrections will be made while referring to the EDC operation manual and input manual.

After confirming that there are no errors in the CRF completed, the investigator will sign it electronically in the EDC.

If there is any inconsistency between data in the CRF and the relevant source document, the investigator will complete the "Explanation of Inconsistency between Case Report Form and Source Document" to explain the reason and will seal this form with his/her name.

## 19.2 Protocol deviation

Any failure to follow the protocol in order to eliminate an immediate hazard to a subject or for any other medically compelling reason will be recorded by the investigator in the "Emergency Protocol Deviation Report." The investigator will immediately submit to the head of the study site a report describing the deviation and the reason for it, which will then be submitted to the IRB via the head of the study site.

Any deviation other than emergency deviation will be handled by the investigator in accordance with the regulations of the study site.

## 19.3 Data management

The person in charge of data management will collect and manage data using the validated EDC in accordance with the pre-established "Procedures for Data Management."

## 19.4 Record retention

Study-related documents or records will be retained for the period specified below. The investigational product provider will notify the study site in writing when a partial change to marketing approval of the investigational product is approved or when it is no longer necessary to retain records due to discontinuation of development.

### 19.4.1 Investigator

The investigator will retain records until 1) or 2) below, whichever occurs later. If the investigational product provider requests a longer retention period, the retention period will be determined in consultation with the investigational product provider. To retain records, a person responsible for retention will be appointed for each record. When the retention period ends, the head of the study site or the founder of the IRB will be notified.

1) Date of approval of partial change to marketing approval based on this study (or 3 years after the decision

to discontinue development if applicable)

2) 3 years after discontinuation or completion of the study

#### 19.4.2 Study site

The head of the study site will retain records until 1) or 2) below, whichever occurs later. If the investigational product provider or the investigator requests a longer retention period, the retention period will be determined in consultation with the investigational product provider and the investigator. To retain records, a person responsible for retention will be appointed for each record.

The head of the study site or the person responsible for retention will take measures to ensure that these records be not lost or destroyed during this retention period and can be provided upon request.

1) Date of approval of partial change to marketing approval based on this study (or 3 years after the decision to discontinue development if applicable)
2) 3 years after discontinuation or completion of the study

#### 19.4.3 Institutional review board (IRB)

The founder of the IRB will retain standard operating procedures, a list of members (including the qualification and affiliation of each member), a list of occupation and affiliation of each member, submitted documents, and records such as summary of minutes of meetings and letters until 1) or 2) below, whichever occurs later. If the investigator requires a longer retention period, the retention period and method will be discussed with the investigator.

1) Date of approval of partial change to marketing approval based on this study (or date of the decision to discontinue development or date of notification that a decision has been made not to attach documents relating to clinical study results, if applicable)
2) 3 years after discontinuation or completion of the study

#### 19.4.4 Investigational product provider

Since the investigational product is designated as a biological product for the approved indications, records will be retained in accordance with the legal regulations pertaining to biological products.

# 20 Funding sources and conflicts of interest

## 20.1 Funding sources

This study will be funded by Zenyaku Kogyo Company, Limited, the investigational product provider, based on the "Agreement on the Conduct of the Investigator-Initiated Clinical Trial." In addition, the investigational product (blinded period), IDEC-C2B8 (open-label period), and safety information will be provided by Zenyaku Kogyo Company, Limited, the investigational product provider.

### 20.2 Conflict of interest

Any potential conflict of interest between the clinical trial steering committee (chairperson and members), investigator and Zenyaku Kogyo Company, Limited, the investigational product provider, with respect to the conduct and publication of the study will be reviewed by the conflict-of-interest committee of the study site to which each belongs in order to maintain fairness in the interest of the study.
Zenyaku Kogyo Company, Limited will provide the investigational product and safety information of the investigational product and analyze the pharmacokinetics of the investigational product (not involved in efficacy or safety results of the study or discussion thereof), but this will not affect the study results, because the company will not be involved in data collection, management, or analysis in this study.

## 21 Payment and insurance

### 21.1 Payment

The investigational product (blinded period) and IDEC-C2B8 (open-label period) will be provided free of charge by Zenyaku Kogyo Company, Limited, the investigational product provider. Medical expenses during the study period will be covered by health insurance, except for the costs of the investigational product and external measurements (peripheral B cells, blood drug concentration, and HACA).

### 21.2 Compensation for injury

If a subject experiences an injury as a result of participation in the study, the study site will take necessary and appropriate measures such as providing a medical system for the treatment. In the study, however, neither out-of-pocket medical expenses nor medical allowances will be paid. Any injury caused by intent or gross negligence of a subject or his/her legal representative (custodial parent or legal guardian) may not be compensated for.
An investigator-initiated study insurance will be purchased to fulfill the obligation to compensate for study-related injury. The head of the study site to which the chairperson of the clinical trial steering committee belongs will be a policyholder, and the investigator and study site will be insured under the above insurance. In addition, the investigator will purchase a medical liability insurance.

## 22 Publication agreement

The results of this study will be submitted to medical journals and presented at academic conferences as the results of a joint study, regardless of the results. A paper or presentation will be co-authored by the chairperson and members of the clinical trial steering committee, investigators at study sites where a certain number of eligible subjects are enrolled, and the statistical analysis manager.
The results of individual subjects enrolled in the study must not be published prior to publication as a joint study.

## 23 Protocol amendment

Upon learning important information for the proper conduct of the study, such as the quality, efficacy, and safety

of the investigational product, the investigator and the clinical trial steering committee will discuss with other investigators and revise the protocol as necessary, and the revised protocol must be agreed on by the investigators and reviewed by the IRB of each study site.

If necessary due to the revision of the protocol, the investigator will promptly revise the informed consent form or information sheet, report the revision to the head of the study site, and submit the revised informed consent form or information sheet to the IRB for review. If the informed consent form or information sheet is revised, the investigator will inform a participating subject and legal representative of the revision, confirm his/her willingness to remain in the study, and obtain consent again using the revised informed consent form and information sheet. The procedures are the same as those described in Section 7.2.1.

# 24 Change to the protocol or discontinuation or suspension of the study

Any of the events listed below will be discussed by the clinical trial steering committee with the statistical analysis manager and the results of the discussion will be reported to the independent data and safety monitoring committee.

The independent data and safety monitoring committee will further investigate as needed and make recommendations to the clinical trial steering committee regarding the necessity of changing the protocol and whether or not to continue the study (continuation, suspension, or discontinuation).

Based on the recommendation of the independent data and safety monitoring committee, the clinical trial steering committee will discuss with the statistical analysis manager to decide whether or not to change the protocol or whether or not to continue the study (continuation, suspension, or discontinuation) and will notify the investigator and the efficacy and safety evaluation committee of its decision.

If the study is discontinued or suspended, the investigator will promptly inform the subject or his/her legal representative (custodial parent or legal guardian) and administer appropriate treatment and measures to the subject.

(1) A safety issue, such as serious ADR, occurs.
(2) The scientific rationale for the development of the investigational product is lost.
(3) The regulatory authority recommends to discontinue the study.
(4) Any other situation that requires a change to the protocol occurs.
(5) Any other situation that requires the discontinuation or suspension of part or all of the study occurs.

If any of the following events occurs, the investigator or the head of the study site will discontinue or suspend the study at the study site:

(1) Significant or persistent non-compliance by the investigator is detected.
(2) The IRB decides to discontinue or suspend the study.

# 25 Study organization

See Attachment 1, "Study organization."

## 26 References


1. Arbeitsgemeinschaft fur Padiatrische Nephrologie. Short versus standard prednisone therapy for initial treatment of idiopathic nephrotic syndrome in children. Lancet 1988; 1: 380-383.
2. Tarshish P, Tobin JN, Bernstein J, Edelman CM Jr. Prognostic significance of the early course of minimal change nephrotic syndrome: report of the International Study of Kidney Disease in Children. J Am Soc Nephrol 1997; 8:769-776.
3. 飯島一誠,本田雅敬,中西浩一他．日本小児腎臓病学会学術委員会：小児特発性ネフローゼ症候群診療ガイドライン 2013. 2013.
4. Iijima K, Hamahira K, Kobayashi A, Nakamura H, Yoshikawa N. Immunohistochemical analysis of renin activity in chronic cyclosporine nephropathy in childhood nephrotic syndrome. J Am Soc Nephrol 2000; 11: 2265-2271.
5. Latta K, von Schnakenburg C, Ehrich JH. A meta-analysis of cytotoxic treatment for frequently relapsing nephrotic syndrome in children. Pediatr Nephrol 2001; 16: 271-282.
6. Yoshioka K, Ohashi Y, Sakai T, Ito H, Yoshikawa N, Nakamura H, et al. A multicenter trial of mizoribine compared with placebo in children with frequently relapsing nephrotic syndrome. Kidney Int 2000; 58: 317-324.
7. Anolik J, Sanz I, Looney J. B cell depletion therapy in systemic lupus erythematosus. Current Rheumatology Reports 2003; 5: 350-356.
8. Looney RJ. Treating human autoimmune disease by depletion B cells. Ann Rheum Dis; 2002; 61(10): 863-866.
9. Iijima K, Sako M, Nozu K, Mori R, Tuchida N, Kamei K, Miura K, Aya k, Nakanishi K, Ohtomo Y, Takahashi S, Tanaka R, Kato H, Nakamura H, Ishikura K, Ito S, Ohashi Y. Rituximab for childhood-onset, complicated, frequently relapsing nephrotic syndrome or steroid-dependent nephrotic syndrome: a multicentre, double-blind, randomised, placebo-controlled trial: Lancet 2014; 384:
10. Benz K, Dotsch J, Rascher W, Stachel D. Change of the course of steroid-dependent nephrotic syndrome after rituximab therapy. Pediatr Nephrol 2004; 19: 794-797.
11. Gilbert RD, Hulse E, Rigden S. Rituximab therapy for steroid-dependent minimal change nephrotic syndrome. Pediatr Nephrol 2006; 21: 1698-1700.
12. Francois H, Daugas E, Bensman A, Ronco P. Unexpected efficacy of rituximab in multirelapsing minimal change nephrotic syndrome in the Adult: First case report and pathophysiological considerations. Am J Kidney Dis 2007; 9: 158-161.
13. Smith GC. Is there a role for rituximab in the treatment of idiopathic childhood nephrotic syndrome? Pediatr Nephrol 2007; 22(6): 893-898.
14. Hofstra JM, Deegens JK, Wetzels JF. Rituximab: effective treatment for severe steroid-dependent minimal change nephrotic syndrome? Nephrol Dial Transplant 2007; 22(7): 2100-2.
15. Kamei K, Ito S, Nozu K, Fujinaga S, Nakayama M, Sako M, Saito M, Yoneko M, Iijima K. Single dose of rituximab for refractory steroid-dependent nephrotic syndrome in children: Pediatr Nephro 2009; 24: 1321-28

16. Ravani P, Rossi R, Bonanni A, Quinn RR, Sica F, Bodria M, Pasini A, Montini G, Edefonti A, Belingheri M, Giovanni DD, Barbano G, Innocenti LD, Scolari F, Murer L, Reiser J, Fornoni A, Ghiggeri GM. Rituximab in Children with Steroid-Depenent Nephrotic Syndrome: A Multicenter, Open-Label, Noninferiority, Randomized Controlled Trial. J AM Soc Nephrol 2015; 26: 2259-66.
17. Kamei K, Ishikura K, Sako M, Tanaka R, Nozu K, Kaito H, Nakanishi K, Ohtomo Y, Miura K, Takahashi S, Morimoto T, Kubota W, Ito S, Nakamura H, Iijima K. Long-term outcome of childhood-onset complicated nephrotic syndrome after a multicenter, double-blind, randomized, placebo-controlled trial of rituximab: Pediatr Nephrol 2017 Jun 29. doi: 10.1007/s00467-017-3718-0. [Epub ahead of print]
18. Kidney Disease: Improving Global Outcomes (KDIGO) Glomerulonephritis Work Group. KDIGO Clinical Practice Guideline for Glomerulonephritis. Kidney Inter Suppl. 2012; 2: 139-274.
19. Nakanishi K, Iijima K, Ishikura K, Hataya H, Nakazato H, Sasaki S, Honda M, Yoshikawa N; Japanese Study Group of Renal Disease in Children. Two-year outcome of the ISKDC regimen and frequent-relapsing risk in children with idiopathic nephrotic syndrome. Clin J Am Soc Nephrol. 2013 May;8(5):756-62. doi: 10.2215/CJN.09010912.
20. Kemper MJ, Gellermann J, Habbig S, Krmar RT, Dittrich K, Jungraithmayr T, Pape L, Patzer L, Billing H, Weber L, Pohl M, Rosenthal K, Rosahl A, Mueller-Wiefel DE, Dotsch J. Long-term follow-up after rituximab for steroid-dependent idiopathic nephrotic syncrome: Nephrol Dial Transplant 2012; 27: 1910-1915.
21. Iijima K, Sako M, Oba MS, Ito S, Hataya H, Tanaka R, et al. Cyclosporine C2 Monitoring for the Treatment of Frequently Relapsing Nephrotic Syndrome in Children: A Multicenter Randomized Phase II Trial. Clin J Am Soc Nephrol. 2014; 9: 271-278.
22. 佐地勉,石川司朗,越前宏俊,岡田知雄,小川俊一,鈴木康之,他．日本循環器学会．循環器病の診断と治療に関するガイドライン（2010-2011 年度合同研究班報告）小児期心疾患における薬物療法ガイドライン．循環器病の診断と治療に関するガイドライン 2012．東京：2012.167-187.
23. Uemura O, Ishikura K, Gotoh Y, Honda M. Creatinine-based estimated glomerular filtration rate for children younger than 2 years. Clin Exp Nephrol. 2018;22: 483-484.
24. Uemura O, Nagai T, Ishikura K, Ito S, Hataya H, Gotoh Y, et al. Creatinine-based equations to estimate glomerular filtration rate in Japanese children and adolescents with chronic kidney disease. Clin Exp Nephrol. 2014;18: 626-633.
25. 日本小児腎臓病学会編．CKD 診療ガイド 2012．日腎会誌 2012; 54: 1031-1189.
26. 田中敏章編著.新しい小児の臨床検査基準値ポケットガイド第 2 版.東京:株式会社じほう;2014.

# 27 Appendices

Appendix 1. Standard height/weight table in 2000

Appendix 2. Height-specific prednisolone dose table

Appendix 3. Height-specific study drug dose table

Appendix 4. Pediatric sex- and age-specific blood pressure norms table[22)]

Appendix 5. Estimated glomerular filtration rate[23-25)]

Appendix 6. Pediatric age-specific liver escape enzyme (GOT) norms table[26)]

Appendix 7. Pediatric age-specific liver escape enzyme (GPT) norms table[26)]

Appendix 1. Standard height/weight table in 2000

<Boys>

| Chronological age (years/months) | Standard height (cm) | Standard weight (kg) |
|---|---|---|
| 0•0 | 49.0 (2.1) | 3.0 (0.4) |
| 0•1 | 53.5 (2.2) | 4.3 (0.6) |
| 0•2 | 57.9 (2.2) | 5.5 (0.7) |
| 0•3 | 61.4 (2.2) | 6.4 (0.8) |
| 0•4 | 64.2 (2.3) | 7.1 (0.9) |
| 0•5 | 66.2 (2.3) | 7.7 (0.8) |
| 0•6 | 67.8 (2.4) | 8.0 (0.9) |
| 0•7 | 69.2 (2.4) | 8.2 (0.9) |
| 0•8 | 70.5 (2.4) | 8.6 (1.0) |
| 0•9 | 71.7 (2.5) | 8.9 (1.0) |
| 0•10 | 72.8 (2.5) | 9.1 (0.9) |
| 0•11 | 73.9 (2.5) | 9.2 (0.9) |
| 1•0 | 75.0 (2.6) | 9.3 (0.9) |
| 1•1 | 76.0 (2.6) | 9.5 (0.9) |
| 1•2 | 76.9 (2.6) | 9.8 (1.0) |
| 1•3 | 77.8 (2.7) | 9.9 (1.0) |
| 1•4 | 78.7 (2.7) | 10.1 (1.0) |
| 1•5 | 79.6 (2.8) | 10.3 (1.1) |
| 1•6 | 80.5 (2.8) | 10.5 (1.2) |
| 1•7 | 81.4 (2.8) | 10.6 (1.1) |
| 1•8 | 82.3 (2.9) | 10.9 (1.1) |
| 1•9 | 83.1 (2.9) | 11.2 (1.2) |
| 1•10 | 83.9 (2.9) | 11.3 (1.2) |
| 1•11 | 84.7 (3.0) | 11.4 (1.1) |
| 2•0 | 85.4 (3.0) | 11.6 (1.2) |
| 2•1 | 86.2 (3.1) | 11.8 (1.2) |
| 2•2 | 86.9 (3.1) | 12.0 (1.2) |
| 2•3 | 87.6 (3.1) | 12.1 (1.3) |
| 2•4 | 88.3 (3.2) | 12.3 (1.3) |
| 2•5 | 88.9 (3.2) | 12.5 (1.3) |
| 2•6 | 89.6 (3.2) | 12.7 (1.3) |
| 2•7 | 90.2 (3.3) | 12.8 (1.3) |
| 2•8 | 90.8 (3.3) | 13.0 (1.4) |
| 2•9 | 91.5 (3.3) | 13.2 (1.4) |
| 2•10 | 92.1 (3.4) | 13.3 (1.4) |
| 2•11 | 92.7 (3.4) | 13.5 (1.5) |

| Chronological age (years/months) | Standard height (cm) | Standard weight (kg) |
|---|---|---|
| 3•0 | 93.3 (3.5) | 13.7 (1.5) |
| 3•1 | 94.0 (3.5) | 13.9 (1.6) |
| 3•2 | 94.6 (3.5) | 14.0 (1.6) |
| 3•3 | 95.1 (3.6) | 14.2 (1.7) |
| 3•4 | 95.7 (3.6) | 14.4 (1.7) |
| 3•5 | 96.3 (3.6) | 14.5 (1.7) |
| 3•6 | 96.9 (3.7) | 14.7 (1.8) |
| 3•7 | 97.5 (3.7) | 14.8 (1.8) |
| 3•8 | 98.0 (3.7) | 15.0 (1.8) |
| 3•9 | 98.6 (3.8) | 15.1 (1.8) |
| 3•10 | 99.1 (3.8) | 15.3 (1.9) |
| 3•11 | 99.7 (3.9) | 15.4 (1.9) |
| 4•0 | 100.2 (3.9) | 15.6 (2.0) |
| 4•1 | 100.8 (3.9) | 15.8 (2.0) |
| 4•2 | 101.3 (4.0) | 15.9 (2.1) |
| 4•3 | 101.9 (4.0) | 16.1 (2.1) |
| 4•4 | 102.4 (4.0) | 16.3 (2.1) |
| 4•5 | 103.0 (4.1) | 16.4 (2.1) |
| 4•6 | 103.5 (4.1) | 16.6 (2.1) |
| 4•7 | 104.0 (4.1) | 16.7 (2.2) |
| 4•8 | 104.6 (4.2) | 16.9 (2.2) |
| 4•9 | 105.1 (4.2) | 17.0 (2.2) |
| 4•10 | 105.6 (4.3) | 17.3 (2.3) |
| 4•11 | 106.2 (4.3) | 17.5 (2.4) |
| 5•0 | 106.7 (4.3) | 17.7 (2.5) |
| 5•1 | 107.3 (4.4) | 17.9 (2.6) |
| 5•2 | 107.8 (4.4) | 18.1 (2.8) |
| 5•3 | 108.3 (4.4) | 18.3 (2.9) |
| 5•4 | 108.9 (4.5) | 18.5 (2.9) |
| 5•5 | 109.4 (4.5) | 18.7 (2.9) |
| 5•6 | 110.0 (4.5) | 18.9 (3.0) |
| 5•7 | 110.5 (4.6) | 19.1 (3.0) |
| 5•8 | 111.1 (4.6) | 19.3 (3.0) |
| 5•9 | 111.6 (4.7) | 19.6 (3.0) |
| 5•10 | 112.2 (4.7) | 19.8 (3.1) |
| 5•11 | 112.7 (4.7) | 20.1 (3.2) |

| Chronological age (years/months) | Standard height (cm) | Standard weight (kg) |
|---|---|---|
| 6•0 | 113.3 (4.8) | 20.3 (3.3) |
| 6•1 | 113.9 (4.8) | 20.6 (3.4) |
| 6•2 | 114.5 (4.8) | 20.8 (3.5) |
| 6•3 | 115.0 (4.9) | 21.1 (3.5) |
| 6•4 | 115.6 (4.9) | 21.3 (3.6) |
| 6•5 | 116.1 (4.9) | 21.6 (3.7) |
| 6•6 | 116.7 (5.0) | 21.8 (3.8) |
| 6•7 | 117.2 (5.0) | 22.0 (3.8) |
| 6•8 | 117.7 (5.0) | 22.2 (3.9) |
| 6•9 | 118.2 (5.0) | 22.5 (3.9) |
| 6•10 | 118.6 (5.0) | 22.7 (4.0) |
| 6•11 | 119.1 (5.0) | 22.9 (4.0) |
| 7•0 | 119.6 (5.1) | 23.1 (4.1) |
| 7•1 | 120.1 (5.1) | 23.3 (4.2) |
| 7•2 | 120.6 (5.1) | 23.5 (4.2) |
| 7•3 | 121.1 (5.1) | 23.8 (4.3) |
| 7•4 | 121.5 (5.1) | 24.0 (4.3) |
| 7•5 | 122.0 (5.1) | 24.2 (4.4) |
| 7•6 | 122.5 (5.1) | 24.4 (4.4) |
| 7•7 | 123.0 (5.2) | 24.7 (4.5) |
| 7•8 | 123.4 (5.2) | 25.0 (4.6) |
| 7•9 | 123.9 (5.2) | 25.2 (4.7) |
| 7•10 | 124.4 (5.2) | 25.5 (4.8) |
| 7•11 | 124.8 (5.3) | 25.8 (4.9) |
| 8•0 | 125.3 (5.3) | 26.1 (5.0) |
| 8•1 | 125.8 (5.3) | 26.3 (5.1) |
| 8•2 | 126.2 (5.3) | 26.6 (5.2) |
| 8•3 | 126.7 (5.4) | 26.9 (5.3) |
| 8•4 | 127.2 (5.4) | 27.2 (5.4) |
| 8•5 | 127.6 (5.4) | 27.4 (5.5) |
| 8•6 | 128.1 (5.5) | 27.7 (5.6) |
| 8•7 | 128.6 (5.5) | 28.0 (5.7) |
| 8•8 | 129.0 (5.5) | 28.3 (5.8) |
| 8•9 | 129.5 (5.5) | 28.6 (5.9) |
| 8•10 | 129.9 (5.5) | 28.9 (6.0) |
| 8•11 | 130.4 (5.6) | 29.2 (6.1) |

<Continued for boys>

| Chronological age (years/months) | Standard height (cm) | Standard weight (kg) |
|---|---|---|
| 9•0 | 130.9 (5.6) | 29.5 (6.2) |
| 9•1 | 131.3 (5.6) | 29.7 (6.3) |
| 9•2 | 131.8 (5.6) | 30.0 (6.4) |
| 9•3 | 132.2 (5.7) | 30.3 (6.5) |
| 9•4 | 132.7 (5.7) | 30.6 (6.6) |
| 9•5 | 133.1 (5.7) | 30.9 (6.7) |
| 9•6 | 133.6 (5.7) | 31.2 (6.8) |
| 9•7 | 134.1 (5.8) | 31.5 (6.9) |
| 9•8 | 134.5 (5.8) | 31.9 (7.0) |
| 9•9 | 135.0 (5.8) | 32.2 (7.1) |
| 9•10 | 135.4 (5.9) | 32.5 (7.2) |
| 9•11 | 135.9 (5.9) | 32.8 (7.3) |
| 10•0 | 136.4 (5.9) | 33.2 (7.4) |
| 10•1 | 136.8 (6.0) | 33.5 (7.5) |
| 10•2 | 137.3 (6.0) | 33.8 (7.6) |
| 10•3 | 137.7 (6.0) | 34.1 (7.7) |
| 10•4 | 138.2 (6.1) | 34.5 (7.8) |
| 10•5 | 138.6 (6.1) | 34.8 (7.8) |
| 10•6 | 139.1 (6.1) | 35.1 (7.9) |
| 10•7 | 139.6 (6.2) | 35.5 (8.0) |
| 10•8 | 140.1 (6.3) | 35.8 (8.1) |
| 10•9 | 140.7 (6.4) | 36.2 (8.2) |
| 10•10 | 141.2 (6.5) | 36.5 (8.3) |
| 10•11 | 141.7 (6.6) | 36.9 (8.4) |
| 11•0 | 142.2 (6.6) | 37.3 (8.5) |
| 11•1 | 142.7 (6.7) | 37.6 (8.6) |
| 11•2 | 143.2 (6.8) | 38.0 (8.7) |
| 11•3 | 143.8 (6.9) | 38.3 (8.8) |
| 11•4 | 144.3 (7.0) | 38.7 (8.9) |
| 11•5 | 144.8 (7.1) | 39.0 (9.0) |
| 11•6 | 145.3 (7.1) | 39.4 (9.2) |
| 11•7 | 145.9 (7.2) | 39.9 (9.3) |
| 11•8 | 146.6 (7.3) | 40.4 (9.4) |
| 11•9 | 147.2 (7.4) | 40.9 (9.5) |
| 11•10 | 147.8 (7.4) | 41.4 (9.6) |
| 11•11 | 148.5 (7.5) | 41.9 (9.7) |

| Chronological age (years/months) | Standard height (cm) | Standard weight (kg) |
|---|---|---|
| 12•0 | 149.1 (7.6) | 42.4 (9.8) |
| 12•1 | 149.7 (7.7) | 42.9 (9.9) |
| 12•2 | 150.4 (7.8) | 43.4 (10.0) |
| 12•3 | 151.0 (7.8) | 43.9 (10.1) |
| 12•4 | 151.6 (7.9) | 44.4 (10.2) |
| 12•5 | 152.3 (8.0) | 44.9 (10.3) |
| 12•6 | 152.9 (8.1) | 45.4 (10.4) |
| 12•7 | 153.5 (8.0) | 45.8 (10.4) |
| 12•8 | 154.1 (8.0) | 46.2 (10.4) |
| 12•9 | 154.7 (8.0) | 46.7 (10.4) |
| 12•10 | 155.3 (7.9) | 47.1 (10.4) |
| 12•11 | 155.9 (7.9) | 47.5 (10.4) |
| 13•0 | 156.5 (7.9) | 47.9 (10.4) |
| 13•1 | 157.0 (7.8) | 48.3 (10.4) |
| 13•2 | 157.6 (7.8) | 48.7 (10.5) |
| 13•3 | 158.2 (7.8) | 49.2 (10.5) |
| 13•4 | 158.8 (7.8) | 49.6 (10.5) |
| 13•5 | 159.4 (7.7) | 50.0 (10.5) |
| 13•6 | 160.0 (7.7) | 50.4 (10.5) |
| 13•7 | 160.5 (7.6) | 50.8 (10.5) |
| 13•8 | 160.9 (7.5) | 51.2 (10.5) |
| 13•9 | 161.4 (7.4) | 51.7 (10.4) |
| 13•10 | 161.8 (7.3) | 52.1 (10.4) |
| 13•11 | 162.3 (7.2) | 52.5 (10.4) |
| 14•0 | 162.8 (7.1) | 52.9 (10.4) |
| 14•1 | 163.2 (7.0) | 53.3 (10.4) |
| 14•2 | 163.7 (6.9) | 53.7 (10.4) |
| 14•3 | 164.1 (6.8) | 54.2 (10.4) |
| 14•4 | 164.6 (6.7) | 54.6 (10.4) |
| 14•5 | 165.0 (6.6) | 55.0 (10.4) |
| 14•6 | 165.5 (6.5) | 55.4 (10.3) |
| 14•7 | 165.8 (6.4) | 55.8 (10.4) |
| 14•8 | 166.0 (6.4) | 56.1 (10.4) |
| 14•9 | 166.3 (6.3) | 56.5 (10.5) |
| 14•10 | 166.5 (6.3) | 56.8 (10.5) |
| 14•11 | 166.8 (6.2) | 57.2 (10.5) |

| Chronological age (years/months) | Standard height (cm) | Standard weight (kg) |
|---|---|---|
| 15•0 | 167.1 (6.2) | 57.6 (10.6) |
| 15•1 | 167.3 (6.1) | 57.9 (10.6) |
| 15•2 | 167.6 (6.1) | 58.3 (10.7) |
| 15•3 | 167.8 (6.0) | 58.6 (10.7) |
| 15•4 | 168.1 (6.0) | 59.0 (10.7) |
| 15•5 | 168.3 (5.9) | 59.3 (10.8) |
| 15•6 | 168.6 (5.9) | 59.7 (10.8) |
| 15•7 | 168.7 (5.9) | 59.8 (10.8) |
| 15•8 | 168.9 (5.9) | 60.0 (10.7) |
| 15•9 | 169.0 (5.9) | 60.1 (10.7) |
| 15•10 | 169.1 (5.9) | 60.2 (10.6) |
| 15•11 | 169.2 (5.8) | 60.3 (10.5) |
| 16•0 | 169.4 (5.8) | 60.5 (10.5) |
| 16•1 | 169.5 (5.8) | 60.6 (10.4) |
| 16•2 | 169.6 (5.8) | 60.7 (10.4) |
| 16•3 | 169.7 (5.8) | 60.8 (10.3) |
| 16•4 | 169.9 (5.8) | 61.0 (10.2) |
| 16•5 | 170.0 (5.8) | 61.1 (10.2) |
| 16•6 | 170.1 (5.8) | 61.2 (10.1) |
| 16•7 | 170.2 (5.8) | 61.3 (10.1) |
| 16•8 | 170.2 (5.8) | 61.4 (10.2) |
| 16•9 | 170.3 (5.8) | 61.6 (10.2) |
| 16•10 | 170.3 (5.8) | 61.7 (10.2) |
| 16•11 | 170.4 (5.8) | 61.8 (10.2) |
| 17•0 | 170.5 (5.8) | 61.9 (10.2) |
| 17•1 | 170.5 (5.8) | 62.0 (10.2) |
| 17•2 | 170.6 (5.8) | 62.1 (10.3) |
| 17•3 | 170.6 (5.8) | 62.3 (10.3) |
| 17•4 | 170.7 (5.8) | 62.4 (10.3) |
| 17•5 | 170.7 (5.8) | 62.5 (10.3) |
| 17•6 | 170.8 (5.8) | 62.6 (10.3) |

For patients aged 17 years and 7 months or older, apply the normal value for those aged 17 years and 6 months.

<Girls>

| Chronological age (years/months) | Standard height (cm) | Standard weight (kg) |
|---|---|---|
| 0•0 | 48.4 (2.1) | 3.0 (0.4) |
| 0•1 | 52.6 (2.1) | 4.1 (0.5) |
| 0•2 | 56.7 (2.2) | 5.2 (0.6) |
| 0•3 | 60.0 (2.2) | 6.0 (0.7) |
| 0•4 | 62.6 (2.2) | 6.6 (0.8) |
| 0•5 | 64.6 (2.3) | 7.0 (0.8) |
| 0•6 | 66.2 (2.3) | 7.5 (0.8) |
| 0•7 | 67.5 (2.3) | 7.8 (0.8) |
| 0•8 | 68.9 (2.4) | 8.0 (0.9) |
| 0•9 | 70.0 (2.4) | 8.2 (0.9) |
| 0•10 | 71.2 (2.4) | 8.5 (0.9) |
| 0•11 | 72.3 (2.5) | 8.6 (0.9) |
| 1•0 | 73.4 (2.5) | 8.7 (1.0) |
| 1•1 | 74.5 (2.5) | 9.0 (0.9) |
| 1•2 | 75.5 (2.6) | 9.2 (0.9) |
| 1•3 | 76.5 (2.6) | 9.3 (1.0) |
| 1•4 | 77.5 (2.6) | 9.5 (0.9) |
| 1•5 | 78.4 (2.7) | 9.7 (1.0) |
| 1•6 | 79.4 (2.7) | 9.9 (1.0) |
| 1•7 | 80.3 (2.8) | 10.2 (1.1) |
| 1•8 | 81.2 (2.8) | 10.4 (1.1) |
| 1•9 | 82.0 (2.8) | 10.4 (1.0) |
| 1•10 | 82.8 (2.9) | 10.7 (1.2) |
| 1•11 | 83.5 (2.9) | 11.0 (1.2) |
| 2•0 | 84.3 (2.9) | 11.0 (1.1) |
| 2•1 | 85.0 (3.0) | 11.2 (1.2) |
| 2•2 | 85.7 (3.0) | 11.4 (1.2) |
| 2•3 | 86.4 (3.0) | 11.6 (1.3) |
| 2•4 | 87.1 (3.1) | 11.8 (1.3) |
| 2•5 | 87.7 (3.1) | 12.0 (1.4) |
| 2•6 | 88.4 (3.1) | 12.2 (1.4) |
| 2•7 | 89.0 (3.2) | 12.3 (1.4) |
| 2•8 | 89.6 (3.2) | 12.5 (1.4) |
| 2•9 | 90.3 (3.3) | 12.7 (1.5) |
| 2•10 | 90.9 (3.3) | 12.8 (1.5) |
| 2•11 | 91.6 (3.3) | 13.0 (1.5) |

| Chronological age (years/months) | Standard height (cm) | Standard weight (kg) |
|---|---|---|
| 3•0 | 92.2 (3.4) | 13.1 (1.6) |
| 3•1 | 92.8 (3.4) | 13.3 (1.6) |
| 3•2 | 93.5 (3.4) | 13.4 (1.6) |
| 3•3 | 94.1 (3.5) | 13.6 (1.7) |
| 3•4 | 94.7 (3.5) | 13.8 (1.7) |
| 3•5 | 95.3 (3.5) | 13.9 (1.7) |
| 3•6 | 95.9 (3.6) | 14.1 (1.7) |
| 3•7 | 96.5 (3.6) | 14.3 (1.7) |
| 3•8 | 97.1 (3.6) | 14.4 (1.7) |
| 3•9 | 97.7 (3.7) | 14.6 (1.7) |
| 3•10 | 98.3 (3.7) | 14.8 (1.8) |
| 3•11 | 98.9 (3.8) | 15.0 (1.9) |
| 4•0 | 99.5 (3.8) | 15.2 (2.0) |
| 4•1 | 100.0 (3.8) | 15.4 (2.1) |
| 4•2 | 100.6 (3.9) | 15.6 (2.2) |
| 4•3 | 101.2 (3.9) | 15.8 (2.4) |
| 4•4 | 101.7 (3.9) | 15.9 (2.3) |
| 4•5 | 102.3 (4.0) | 16.1 (2.2) |
| 4•6 | 102.8 (4.0) | 16.3 (2.2) |
| 4•7 | 103.4 (4.0) | 16.4 (2.1) |
| 4•8 | 103.9 (4.1) | 16.6 (2.1) |
| 4•9 | 104.5 (4.1) | 16.8 (2.0) |
| 4•10 | 105.0 (4.1) | 17.0 (2.1) |
| 4•11 | 105.6 (4.2) | 17.2 (2.2) |
| 5•0 | 106.2 (4.2) | 17.4 (2.3) |
| 5•1 | 106.7 (4.3) | 17.6 (2.4) |
| 5•2 | 107.3 (4.3) | 17.8 (2.5) |
| 5•3 | 107.8 (4.3) | 18.0 (2.6) |
| 5•4 | 108.4 (4.4) | 18.1 (2.6) |
| 5•5 | 108.9 (4.4) | 18.2 (2.6) |
| 5•6 | 109.5 (4.4) | 18.4 (2.7) |
| 5•7 | 110.0 (4.5) | 18.5 (2.7) |
| 5•8 | 110.6 (4.5) | 18.6 (2.7) |
| 5•9 | 111.1 (4.5) | 18.7 (2.8) |
| 5•10 | 111.6 (4.6) | 19.0 (2.8) |
| 5•11 | 112.2 (4.6) | 19.3 (2.9) |

| Chronological age (years/months) | Standard height (cm) | Standard weight (kg) |
|---|---|---|
| 6•0 | 112.7 (4.6) | 19.6 (3.0) |
| 6•1 | 113.3 (4.7) | 19.9 (3.1) |
| 6•2 | 113.8 (4.7) | 20.2 (3.2) |
| 6•3 | 114.1 (4.6) | 20.4 (3.3) |
| 6•4 | 114.6 (4.7) | 20.7 (3.4) |
| 6•5 | 115.2 (4.8) | 21.0 (3.5) |
| 6•6 | 115.8 (4.9) | 21.3 (3.6) |
| 6•7 | 116.3 (4.9) | 21.5 (3.6) |
| 6•8 | 116.8 (4.9) | 21.7 (3.7) |
| 6•9 | 117.3 (4.9) | 21.9 (3.7) |
| 6•10 | 117.8 (5.0) | 22.1 (3.8) |
| 6•11 | 118.3 (5.0) | 22.3 (3.8) |
| 7•0 | 118.8 (5.0) | 22.6 (3.9) |
| 7•1 | 119.2 (5.0) | 22.8 (3.9) |
| 7•2 | 119.7 (5.0) | 23.0 (4.0) |
| 7•3 | 120.2 (5.1) | 23.2 (4.1) |
| 7•4 | 120.7 (5.1) | 23.4 (4.1) |
| 7•5 | 121.2 (5.1) | 23.6 (4.2) |
| 7•6 | 121.7 (5.1) | 23.8 (4.2) |
| 7•7 | 122.2 (5.2) | 24.1 (4.3) |
| 7•8 | 122.7 (5.2) | 24.3 (4.4) |
| 7•9 | 123.2 (5.2) | 24.6 (4.5) |
| 7•10 | 123.6 (5.3) | 24.9 (4.6) |
| 7•11 | 124.1 (5.3) | 25.1 (4.7) |
| 8•0 | 124.6 (5.4) | 25.4 (4.7) |
| 8•1 | 125.1 (5.4) | 25.7 (4.8) |
| 8•2 | 125.6 (5.4) | 25.9 (4.9) |
| 8•3 | 126.1 (5.5) | 26.2 (5.0) |
| 8•4 | 126.5 (5.5) | 26.5 (5.1) |
| 8•5 | 127.0 (5.5) | 26.7 (5.2) |
| 8•6 | 127.5 (5.6) | 27.0 (5.3) |
| 8•7 | 128.0 (5.6) | 27.3 (5.4) |
| 8•8 | 128.5 (5.7) | 27.6 (5.5) |
| 8•9 | 129.0 (5.7) | 27.9 (5.5) |
| 8•10 | 129.5 (5.8) | 28.2 (5.6) |
| 8•11 | 130.0 (5.8) | 28.5 (5.7) |

<Continued for girls>

| Chronological age (years/months) | Standard height (cm) | Standard weight (kg) |
|---|---|---|
| 9•0 | 130.5 (5.9) | 28.9 (5.8) |
| 9•1 | 131.0 (5.9) | 29.2 (5.9) |
| 9•2 | 131.5 (6.0) | 29.5 (6.0) |
| 9•3 | 132.0 (6.0) | 29.8 (6.1) |
| 9•4 | 132.5 (6.1) | 30.1 (6.2) |
| 9•5 | 133.0 (6.1) | 30.4 (6.3) |
| 9•6 | 133.5 (6.2) | 30.7 (6.4) |
| 9•7 | 134.1 (6.2) | 31.1 (6.5) |
| 9•8 | 134.6 (6.3) | 31.4 (6.6) |
| 9•9 | 135.2 (6.3) | 31.8 (6.7) |
| 9•10 | 135.8 (6.4) | 32.1 (6.8) |
| 9•11 | 136.3 (6.4) | 32.5 (6.9) |
| 10•0 | 136.9 (6.5) | 32.8 (7.0) |
| 10•1 | 137.5 (6.5) | 33.2 (7.1) |
| 10•2 | 138.0 (6.6) | 33.5 (7.1) |
| 10•3 | 138.6 (6.6) | 33.9 (7.2) |
| 10•4 | 139.2 (6.7) | 34.2 (7.3) |
| 10•5 | 139.7 (6.7) | 34.6 (7.4) |
| 10•6 | 140.3 (6.8) | 34.9 (7.5) |
| 10•7 | 140.9 (6.8) | 35.3 (7.6) |
| 10•8 | 141.4 (6.8) | 35.8 (7.7) |
| 10•9 | 142.0 (6.8) | 36.2 (7.7) |
| 10•10 | 142.6 (6.8) | 36.6 (7.8) |
| 10•11 | 143.1 (6.7) | 37.1 (7.9) |
| 11•0 | 143.7 (6.7) | 37.5 (7.9) |
| 11•1 | 144.3 (6.7) | 37.9 (8.0) |
| 11•2 | 144.8 (6.7) | 38.4 (8.1) |
| 11•3 | 145.4 (6.7) | 38.8 (8.1) |
| 11•4 | 146.0 (6.7) | 39.2 (8.2) |
| 11•5 | 146.5 (6.7) | 39.7 (8.3) |
| 11•6 | 147.1 (6.7) | 40.1 (8.4) |
| 11•7 | 147.5 (6.6) | 40.5 (8.4) |
| 11•8 | 147.9 (6.5) | 40.9 (8.4) |
| 11•9 | 148.4 (6.5) | 41.3 (8.4) |
| 11•10 | 148.8 (6.4) | 41.7 (8.4) |
| 11•11 | 149.2 (6.4) | 42.1 (8.5) |

| Chronological age (years/months) | Standard height (cm) | Standard weight (kg) |
|---|---|---|
| 12•0 | 149.6 (6.3) | 42.6 (8.5) |
| 12•1 | 150.0 (6.2) | 43.0 (8.5) |
| 12•2 | 150.4 (6.2) | 43.4 (8.5) |
| 12•3 | 150.9 (6.1) | 43.8 (8.5) |
| 12•4 | 151.3 (6.1) | 44.2 (8.6) |
| 12•5 | 151.7 (6.0) | 44.6 (8.6) |
| 12•6 | 152.1 (5.9) | 45.0 (8.6) |
| 12•7 | 152.4 (5.9) | 45.3 (8.6) |
| 12•8 | 152.6 (5.8) | 45.6 (8.5) |
| 12•9 | 152.9 (5.8) | 45.8 (8.5) |
| 12•10 | 153.1 (5.8) | 46.1 (8.5) |
| 12•11 | 153.4 (5.7) | 46.4 (8.4) |
| 13•0 | 153.6 (5.7) | 46.7 (8.4) |
| 13•1 | 153.9 (5.6) | 46.9 (8.4) |
| 13•2 | 154.1 (5.6) | 47.2 (8.4) |
| 13•3 | 154.4 (5.5) | 47.5 (8.3) |
| 13•4 | 154.6 (5.5) | 47.8 (8.3) |
| 13•5 | 154.9 (5.4) | 48.0 (8.3) |
| 13•6 | 155.1 (5.4) | 48.3 (8.2) |
| 13•7 | 155.2 (5.4) | 48.5 (8.2) |
| 13•8 | 155.4 (5.4) | 48.7 (8.2) |
| 13•9 | 155.5 (5.4) | 48.9 (8.2) |
| 13•10 | 155.7 (5.4) | 49.1 (8.1) |
| 13•11 | 155.8 (5.4) | 49.3 (8.1) |
| 14•0 | 156.0 (5.4) | 49.5 (8.1) |
| 14•1 | 156.1 (5.3) | 49.7 (8.1) |
| 14•2 | 156.2 (5.3) | 49.9 (8.0) |
| 14•3 | 156.4 (5.3) | 50.1 (8.0) |
| 14•4 | 156.5 (5.3) | 50.3 (8.0) |
| 14•5 | 156.7 (5.3) | 50.5 (8.0) |
| 14•6 | 156.8 (5.3) | 50.7 (8.0) |
| 14•7 | 156.8 (5.3) | 50.8 (8.0) |
| 14•8 | 156.9 (5.3) | 50.9 (8.0) |
| 14•9 | 156.9 (5.3) | 51.1 (8.0) |
| 14•10 | 157.0 (5.3) | 51.2 (8.1) |
| 14•11 | 157.0 (5.3) | 51.3 (8.1) |

| Chronological age (years/months) | Standard height (cm) | Standard weight (kg) |
|---|---|---|
| 15•0 | 157.1 (5.3) | 51.4 (8.1) |
| 15•1 | 157.1 (5.3) | 51.5 (8.1) |
| 15•2 | 157.1 (5.2) | 51.6 (8.2) |
| 15•3 | 157.2 (5.2) | 51.8 (8.2) |
| 15•4 | 157.2 (5.2) | 51.9 (8.2) |
| 15•5 | 157.3 (5.2) | 52.0 (8.2) |
| 15•6 | 157.3 (5.2) | 52.1 (8.3) |
| 15•7 | 157.3 (5.2) | 52.2 (8.2) |
| 15•8 | 157.4 (5.2) | 52.3 (8.2) |
| 15•9 | 157.4 (5.2) | 52.3 (8.1) |
| 15•10 | 157.4 (5.2) | 52.4 (8.1) |
| 15•11 | 157.5 (5.2) | 52.5 (8.1) |
| 16•0 | 157.5 (5.2) | 52.6 (8.0) |
| 16•1 | 157.5 (5.2) | 52.6 (8.0) |
| 16•2 | 157.6 (5.2) | 52.7 (8.0) |
| 16•3 | 157.6 (5.2) | 52.8 (7.9) |
| 16•4 | 157.6 (5.2) | 52.9 (7.9) |
| 16•5 | 157.7 (5.2) | 52.9 (7.8) |
| 16•6 | 157.7 (5.2) | 53.0 (7.8) |
| 16•7 | 157.7 (5.2) | 53.0 (7.8) |
| 16•8 | 157.8 (5.2) | 53.0 (7.8) |
| 16•9 | 157.8 (5.2) | 53.0 (7.8) |
| 16•10 | 157.8 (5.2) | 53.0 (7.8) |
| 16•11 | 157.9 (5.2) | 53.0 (7.8) |
| 17•0 | 157.9 (5.2) | 53.1 (7.9) |
| 17•1 | 157.9 (5.2) | 53.1 (7.9) |
| 17•2 | 158.0 (5.2) | 53.1 (7.9) |
| 17•3 | 158.0 (5.2) | 53.1 (7.9) |
| 17•4 | 158.0 (5.2) | 53.1 (7.9) |
| 17•5 | 158.1 (5.2) | 53.1 (7.9) |
| 17•6 | 158.1 (5.3) | 53.1 (7.9) |

For patients aged 17 years and 7 months or older, apply the normal value for those aged 17 years and 6 months.

Appendix 2. Height-specific prednisolone dose table

The dose of prednisolone will be determined in increments of 5 mg based on the body surface area calculated from height (the ones place is 0 for ≥ 0 and < 2.5, 5 for ≥ 2.5 and < 7.5, and 0 for ≥ 7.5 with 1 being added to the tens place).

The body surface area was calculated from height and height-based standard weight using the Du Bois formula.
Body surface area (BSA) ($m^2$) = weight (kg)$^{0.425}$ × height (cm)$^{0.725}$ × 0.007184 (Du Bois)
Standard height/weight table in 2000 was used to determine height-based standard weight. If no corresponding height was available, "nearest height was used" or "greater height was used if there were two nearest heights."

■When the maximum dose is 60 mg/day for 60 mg/m2/day

<Boys> <Girls>

| Height (cm) | Dose of prednisolone (daily dose per body surface area or dose) | | | | Height (cm) | Dose of prednisolone (daily dose per body surface area or dose) | | | |
|---|---|---|---|---|---|---|---|---|---|
| | (1) | (2) | (3) | (4) | | (1) | (2) | (3) | (4) |
| | 60mg/$m^2$ (mg/day) | 60mg/$m^2$ (mg) | 30mg/$m^2$ (mg) | 15mg/$m^2$ (mg) | | 60mg/$m^2$ (mg/day) | 60mg/$m^2$ (mg) | 30mg/$m^2$ (mg) | 15mg/$m^2$ (mg) |
| | In 3 divided doses Every day | Every other morning Once | Every other morning Once | Every other morning Once | | In 3 divided doses Every day | Every other morning Once | Every other morning Once | Every other morning Once |
| 59.6 - | 20 | 20 | 10 | 5 | 58.7 - | 20 | 20 | 10 | 5 |
| 68.4 - | 25 | 25 | 10 | 5 | 69.5 - | 25 | 25 | 10 | 5 |
| 73.4 - | 25 | 25 | 15 | 5 | 73.8 - | 25 | 25 | 15 | 5 |
| 79.4 - | 30 | 30 | 15 | 5 | 80.0 - | 30 | 30 | 15 | 5 |
| 84.0 - | 30 | 30 | 15 | 10 | 84.8 - | 30 | 30 | 15 | 10 |
| 88.1 - | 35 | 35 | 15 | 10 | 89.0 - | 35 | 35 | 15 | 10 |
| 92.9 - | 35 | 35 | 20 | 10 | 93.8 - | 35 | 35 | 20 | 10 |
| 97.6 - | 40 | 40 | 20 | 10 | 97.7 - | 40 | 40 | 20 | 10 |
| 105.8 - | 45 | 45 | 20 | 10 | 106.0 - | 45 | 45 | 20 | 10 |
| 109.2 - | 45 | 45 | 25 | 10 | 109.9 - | 45 | 45 | 25 | 10 |
| 113.0 - | 50 | 50 | 25 | 10 | 113.2 - | 50 | 50 | 25 | 10 |
| 115.9 - | 50 | 50 | 25 | 15 | 116.1 - | 50 | 50 | 25 | 15 |
| 119.4 - | 55 | 55 | 25 | 15 | 119.5 - | 55 | 55 | 25 | 15 |
| 122.8 - | 55 | 55 | 30 | 15 | 123.0 - | 55 | 55 | 30 | 15 |
| 125.6 - | 60 | 60 | 30 | 15 | 125.9 - | 60 | 60 | 30 | 15 |

Appendix 3. Height-specific study drug dose table

<Boy>

| Height (cm) | Dose (mg) |
|---|---|
| 59.6 - | 120 |
| 62.6 - | 130 |
| 67.2 - | 140 |
| 69.5 - | 150 |
| 73.4 - | 160 |
| 76.5 - | 170 |
| 80.2 - | 180 |
| 82.9 - | 190 |
| 86.3 - | 200 |
| 88.8 - | 210 |
| 91.6 - | 220 |
| 94.7 - | 230 |
| 97.6 - | 240 |
| 100.1 - | 250 |
| 102.9 - | 260 |
| 105.2 - | 270 |
| 108.0 - | 280 |
| 110.3 - | 290 |
| 112.5 - | 300 |
| 114.7 - | 310 |
| 116.4 - | 320 |
| 118.9 - | 330 |
| 120.9 - | 340 |
| 122.8 - | 350 |
| 124.6 - | 360 |
| 126.5 - | 370 |
| 128.4 - | 380 |
| 130.0 - | 390 |
| 132.0 - | 400 |
| 133.9 - | 410 |
| 135.2 - | 420 |
| 137.1 - | 430 |
| 138.4 - | 440 |
| 140.4 - | 450 |
| 142.0 - | 460 |
| 143.5 - | 470 |
| 145.6 - | 480 |
| 146.9 - | 490 |
| 148.2 - | 500 |

<Girls>

| Height (cm) | Dose (mg) |
|---|---|
| 61.4 - | 120 |
| 63.5 - | 130 |
| 66.8 - | 140 |
| 70.7 - | 150 |
| 73.8 - | 160 |
| 77.1 - | 170 |
| 81.0 - | 180 |
| 83.5 - | 190 |
| 86.3 - | 200 |
| 89.0 - | 210 |
| 92.4 - | 220 |
| 95.5 - | 230 |
| 97.7 - | 240 |
| 100.4 - | 250 |
| 103.4 - | 260 |
| 105.5 - | 270 |
| 107.9 - | 280 |
| 110.9 - | 290 |
| 112.6 - | 300 |
| 114.9 - | 310 |
| 117.1 - | 320 |
| 119.0 - | 330 |
| 121.0 - | 340 |
| 123.0 - | 350 |
| 124.9 - | 360 |
| 126.8 - | 370 |
| 128.8 - | 380 |
| 130.3 - | 390 |
| 132.3 - | 400 |
| 133.8 - | 410 |
| 136.1 - | 420 |
| 137.8 - | 430 |
| 139.5 - | 440 |
| 141.2 - | 450 |
| 142.9 - | 460 |
| 144.6 - | 470 |
| 145.7 - | 480 |
| 147.3 - | 490 |
| 149.0 - | 500 |

Appendix 4. Pediatric sex- and age-specific blood pressure norms table[22)]

| | Boys | | | Girls | | |
|---|---|---|---|---|---|---|
| | 90th | 95th | 99th | 90th | 95th | 99th |
| 1 year | 99/52 | 103/56 | 110/64 | 100/54 | 104/58 | 111/65 |
| 2 years | 102/57 | 106/61 | 113/69 | 101/59 | 105/63 | 112/70 |
| 3 years | 105/61 | 109/65 | 116/73 | 103/63 | 107/67 | 114/74 |
| 4 years | 107/65 | 111/69 | 118/77 | 104/66 | 108/70 | 115/77 |
| 5 years | 108/68 | 112/72 | 120/80 | 106/68 | 110/72 | 117/79 |
| 6 years | 110/70 | 114/74 | 121/82 | 108/70 | 111/74 | 119/81 |
| 7 years | 111/72 | 115/76 | 122/84 | 109/71 | 113/75 | 120/82 |
| 8 years | 112/73 | 116/78 | 123/86 | 111/72 | 115/76 | 122/83 |
| 9 years | 114/75 | 118/79 | 125/87 | 113/73 | 117/77 | 124/84 |
| 10 years | 115/75 | 119/80 | 127/88 | 115/74 | 119/78 | 126/86 |
| 11 years | 117/76 | 121/80 | 129/88 | 117/75 | 121/79 | 128/87 |
| 12 years | 120/76 | 123/81 | 131/89 | 119/76 | 123/80 | 130/88 |
| 13 years | 122/77 | 126/81 | 133/89 | 121/77 | 124/81 | 132/89 |
| 14 years | 125/78 | 128/82 | 136/90 | 122/78 | 126/82 | 133/90 |
| 15 years | 127/79 | 131/83 | 138/91 | 123/79 | 127/83 | 134/91 |
| 16 years | 130/80 | 134/84 | 141/92 | 124/80 | 128/84 | 135/91 |
| 17 years | 132/82 | 136/87 | 143/94 | 125/80 | 129/84 | 136/91 |

Systolic/diastolic blood pressure (mmHg)

For patients aged 18 years or older, apply the normal value for those aged 17 years.

## Appendix 5. Estimated glomerular filtration rate[23-25)]

<Patients aged between 3 months and less than 2 years>

Calculate the estimated glomerular filtration rate according to the following procedure[23)]:

1) Calculate the normal serum Cr value (mg/dL) from the following formula using height Ht (m):

   Boys: $-1.259\ Ht^5+7.815\ Ht^4-18.57\ Ht^3+21.39\ Ht^2-11.71\ Ht+2.628$

   Girls: $-4.536\ Ht^5+27.16\ Ht^4-63.47\ Ht^3+72.43\ Ht^2-40.06\ Ht+8.778$

2) Calculate the provisional estimated glomerular filtration rate.

   Provisional estimated glomerular filtration rate = 110.2 × (normal serum Cr / measured serum Cr) + 2.93

3) Calculate the estimated glomerular filtration rate ratio R in children aged less than 2 years and children aged 2 years or older.

   R = 0.107 × (age [months]) + 0.656

4) Calculate the estimated glomerular filtration rate from 2) and 3).

   Estimated glomerular filtration rate = provisional estimated glomerular filtration rate × R

<Patients aged between 2 years and less than 19 years>

Calculate the normal serum Cr value using height Ht (m) and then estimated glomerular filtration rate based on it. [24)]

Estimated glomerular filtration rate (mL/min/1.73 $m^2$)

= 110.2 × normal serum Cr (mg/dL) / measured serum Cr (mg/dL) + 2.93

Calculate the normal serum Cr value (mg/dL) from the following formula:

Boys: $-1.259\ Ht^5+7.815\ Ht^4-18.57\ Ht^3+21.39\ Ht^2-11.71\ Ht+2.628$

Girls: $-4.536\ Ht^5+27.16\ Ht^4-63.47\ Ht^3+72.43\ Ht^2-40.06\ Ht+8.778$

<Patients aged 19 years or older>

Use the calculation formula described below (Japanese Society for Nephrology)[25)] based on height and serum Cr (enzymatic method).

Use serum Cr rounded to two decimal places.

Estimated glomerular filtration rate in males = 194 × serum Cr (enzymatic method) $[mg/dL]^{-1.094}$ × age $(years)^{-0.287}$

Estimated glomerular filtration rate in females = 194 × serum Cr (enzymatic method) $[mg/dL]^{-1.094}$ × age $(years)^{-0.287}$ × 0.739

Appendix 6. Pediatric age-specific liver escape enzyme (GOT) norms table[26)]

<Boys>

| Age | Lower limit (U/L) | Upper limit (U/L) |
|---|---|---|
| 0 months | 19.9 | 62.0 |
| 1 month | 21.0 | 64.0 |
| 2 months | 22.0 | 65.0 |
| 3 months | 22.3 | 66.0 |
| 4 months | 23.0 | 67.0 |
| 5 months | 24.0 | 68.0 |
| 6 months | 24.5 | 68.0 |
| 7 months | 25.0 | 67.5 |
| 8 months | 24.5 | 66.5 |
| 9 months | 24.0 | 65.5 |
| 10 months | 23.5 | 63.9 |
| 11 months | 23.0 | 61.5 |
| 1 year | 23.0 | 56.5 |
| 2 years | 24.0 | 49.0 |
| 3 years | 24.0 | 43.0 |
| 4 years | 24.0 | 40.8 |
| 5 years | 24.0 | 38.7 |
| 6 years | 24.0 | 37.5 |
| 7 years | 24.0 | 36.0 |
| 8 years | 22.5 | 34.8 |
| 9 years | 19.0 | 33.0 |
| 10 years | 17.0 | 32.0 |
| 11 years | 16.0 | 31.5 |
| 12 years | 15.0 | 31.0 |
| 13 years | 14.5 | 31.0 |
| 14 years | 14.0 | 30.0 |
| 15 years | 14.0 | 30.0 |
| 16 years | 14.0 | 30.0 |
| 17 years | 14.0 | 30.0 |
| 18 years | 14.0 | 30.0 |
| 19 years | 14.0 | 31.0 |
| 20 years | 14.0 | 32.0 |

<Girls>

| Age | Lower limit (U/L) | Upper limit (U/L) |
|---|---|---|
| 0 months | 19.9 | 62.0 |
| 1 month | 21.0 | 64.0 |
| 2 months | 22.0 | 65.0 |
| 3 months | 22.3 | 66.0 |
| 4 months | 23.0 | 67.0 |
| 5 months | 24.0 | 68.0 |
| 6 months | 24.5 | 68.0 |
| 7 months | 25.0 | 67.5 |
| 8 months | 24.5 | 66.5 |
| 9 months | 24.0 | 65.5 |
| 10 months | 23.5 | 63.9 |
| 11 months | 23.0 | 61.5 |
| 1 year | 24.0 | 57.0 |
| 2 years | 24.0 | 50.0 |
| 3 years | 24.0 | 44.0 |
| 4 years | 24.0 | 41.5 |
| 5 years | 24.0 | 39.0 |
| 6 years | 24.0 | 37.5 |
| 7 years | 24.0 | 35.5 |
| 8 years | 22.5 | 33.5 |
| 9 years | 18.5 | 32.0 |
| 10 years | 17.0 | 31.0 |
| 11 years | 16.0 | 30.0 |
| 12 years | 15.0 | 29.5 |
| 13 years | 14.0 | 29.0 |
| 14 years | 13.5 | 28.0 |
| 15 years | 13.0 | 28.0 |
| 16 years | 12.5 | 28.0 |
| 17 years | 12.0 | 28.0 |
| 18 years | 12.0 | 28.0 |
| 19 years | 12.0 | 27.5 |
| 20 years | 12.0 | 27.0 |

The normal value is 10 to 40 (U/L) for males and females aged 21 years or older.

Appendix 7. Pediatric age-specific liver escape enzyme (GPT) norms table[26)]

<Boys>

| Age | Lower limit (U/L) | Upper limit (U/L) |
|---|---|---|
| 0 months | 11.0 | 45.0 |
| 1 month | 11.7 | 50.0 |
| 2 months | 12.5 | 54.5 |
| 3 months | 13.0 | 56.0 |
| 4 months | 13.0 | 56.0 |
| 5 months | 12.9 | 55.5 |
| 6 months | 12.5 | 54.5 |
| 7 months | 12.3 | 53.0 |
| 8 months | 12.0 | 50.5 |
| 9 months | 11.5 | 48.0 |
| 10 months | 10.5 | 45.0 |
| 11 months | 9.5 | 42.0 |
| 1 year | 9.4 | 38.4 |
| 2 years | 9.0 | 34.0 |
| 3 years | 9.0 | 30.0 |
| 4 years | 9.0 | 28.0 |
| 5 years | 9.0 | 28.0 |
| 6 years | 9.0 | 28.0 |
| 7 years | 9.0 | 28.0 |
| 8 years | 9.0 | 28.5 |
| 9 years | 9.0 | 29.0 |
| 10 years | 9.0 | 30.0 |
| 11 years | 9.0 | 31.0 |
| 12 years | 9.0 | 32.0 |
| 13 years | 9.0 | 33.0 |
| 14 years | 9.0 | 34.0 |
| 15 years | 9.0 | 35.0 |
| 16 years | 9.0 | 36.0 |
| 17 years | 9.0 | 37.0 |
| 18 years | 9.0 | 38.0 |
| 19 years | 9.0 | 39.0 |
| 20 years | 9.0 | 41.0 |

<Girls>

| Age | Lower limit (U/L) | Upper limit (U/L) |
|---|---|---|
| 0 months | 11.0 | 45.0 |
| 1 month | 11.7 | 50.0 |
| 2 months | 12.5 | 54.5 |
| 3 months | 13.0 | 56.0 |
| 4 months | 13.0 | 56.0 |
| 5 months | 12.9 | 55.5 |
| 6 months | 12.5 | 54.5 |
| 7 months | 12.3 | 53.0 |
| 8 months | 12.0 | 50.5 |
| 9 months | 11.5 | 48.0 |
| 10 months | 10.5 | 45.0 |
| 11 months | 9.5 | 42.0 |
| 1 year | 9.4 | 38.4 |
| 2 years | 9.0 | 34.0 |
| 3 years | 9.0 | 30.0 |
| 4 years | 9.0 | 28.0 |
| 5 years | 9.0 | 27.0 |
| 6 years | 9.0 | 27.0 |
| 7 years | 9.0 | 27.0 |
| 8 years | 9.0 | 27.0 |
| 9 years | 9.0 | 27.0 |
| 10 years | 9.0 | 27.0 |
| 11 years | 9.0 | 27.5 |
| 12 years | 9.0 | 28.0 |
| 13 years | 9.0 | 28.0 |
| 14 years | 9.0 | 28.5 |
| 15 years | 9.0 | 29.0 |
| 16 years | 9.0 | 29.5 |
| 17 years | 9.0 | 30.0 |
| 18 years | 9.0 | 30.5 |
| 19 years | 9.0 | 31.0 |
| 20 years | 9.0 | 32.0 |

The normal value is 5 to 40 (U/L) for males and females aged 21 years or older.

Date of creation: 25 July 2018

| Page | Section | Before change (version 1.0, 29 June 2018) | After change (version 1.1, 25 July 2018) | Reason for change |
|---|---|---|---|---|
| 11 | 1.5 Study schedule | Table 1-5 Study schedule in the open-label period (IDEC-C2B8 treatment)<br>— | Table 1-5 Study schedule in the open-label period (IDEC-C2B8 treatment)<br>Measurement of height/weight during the switching period | Addition due to missing description (addition of measurement points in Table 1-5) |
| 33 | 5.2 Exclusion criteria | (6) Patients with uncontrolled hypertension* despite treatment with antihypertensives at enrollment<br>* ≥ 99th percentile in the pediatric sex- and age-specific blood pressure norms table | (6) Patients with uncontrolled hypertension* despite treatment with antihypertensives at enrollment<br>* ≥ 99th percentile in the pediatric sex- and age-specific blood pressure norms table[22] (Appendix 4) | Additions due to addition of Appendix 4 |
| 33 | 5.2 Exclusion criteria | (7) Patients with reduced renal function (estimated glomerular filtration rate** < 60 mL/min/1.73 $m^2$) at enrollment<br>** See the estimated glomerular filtration rate. | (7) Patients with reduced renal function (estimated glomerular filtration rate** < 60 mL/min/1.73 $m^2$) at enrollment<br>** See the estimated glomerular filtration rate[23)-25)] (Appendix 5). | Additions due to addition of Appendix 5 |
| 34 | 5.2 Exclusion criteria | (12) Patients who have at least one of the following laboratory parameters at enrollment ・・・<br>4) AST (GOT) ≥ 2.5 times the upper limit of normal in the pediatric age-specific liver escape enzyme (GOT) norms table for subjects aged less than 21 years or ≥ 2.5 times the upper limit of normal at the study site for subjects aged 21 years or older<br>5) ALT (GPT) ≥ 2.5 times the upper limit of normal in the pediatric age-specific liver escape enzyme (GPT) norms table for subjects aged less than 21 years or ≥ 2.5 times the upper limit of normal at the study site for subjects aged 21 years or older | (12) Patients who have at least one of the following laboratory parameters at enrollment ・・・<br>4) AST (GOT) ≥ 2.5 times the upper limit of normal in the pediatric age-specific liver escape enzyme (GOT) norms table[26] (Appendix 6)<br>5) ALT (GPT) ≥ 2.5 times the upper limit of normal in the pediatric age-specific liver escape enzyme (GPT) norms table[26] (Appendix 7) | Additions due to addition of Appendix 6 and Appendix 7 |
| 45 | 9.1.1 Treatment with prednisolone for the | 9.1.1 Treatment with prednisolone for the last relapse | 9.1.1 Treatment with prednisolone for the last relapse before enrollment | Amendment due to terminology unification |

| Page | Section | Before change (version 1.0, 29 June 2018) | After change (version 1.1, 25 July 2018) | Reason for change |
|---|---|---|---|---|
| | last relapse before enrollment | | | |
| 45 | 9.1.1 Treatment with prednisolone for the last relapse before enrollment | Since the study population is subjects with frequently relapsing/steroid-dependent nephrotic syndrome, which is presumed to be highly active, a 4-week duration of treatment with Regimen (1) is recommended. | Since the study population is subjects with frequently relapsing/steroid-dependent nephrotic syndrome, which is presumed to be highly active, the duration of treatment with Regimen (1) is as follows:<br>1) "4 weeks" if the patient is receiving prednisolone at the time of the last recurrence before enrollment<br>2) "4 weeks" is recommended if the patient is not receiving prednisolone at the time of the last recurrence before enrollment, but "until negative urine protein dipstick for 3 days" is acceptable. | Amendment based on discussion between the clinical trial steering committee and the sponsor-investigator |
| 45 | 9.1.1 Treatment with prednisolone for the last relapse before enrollment | The maximum dose in Regimen (1) is either 80 mg/day or 60 mg/day. | The maximum dose in Regimen (1) is 60 mg/day. | Amendment based on discussion between the clinical trial steering committee and the sponsor-investigator |
| 46 | 9.1.1 Treatment with prednisolone for the last relapse e before enrollment | <When the maximum dose is 80 mg/day for 60 $mg/m^2$/day><br>(1) 60 $mg/m^2$/day (up to 80 mg/day) in 3 divided doses every day<br>(2) 60 $mg/m^2$ (up to 80 mg/day) once every other morning for 14 days<br>(3) 30 $mg/m^2$ (up to 40 mg) once every other morning for 14 days<br>(4) 15 $mg/m^2$ (up to 20 mg) once every other morning for 14 days and discontinued | - (deletion) | Amendment based on discussion between the clinical trial steering committee and the sponsor-investigator |
| 54 | 9.6.1 Treatment criteria for IDEC-C2B8 (open-label period) treatment | (4) Patients with uncontrolled hypertension* despite treatment with antihypertensives at the time of confirmation of IDEC-C2B8 treatment criteria<br>* ≥ 99th percentile in the pediatric sex- and age- | (4) Patients with uncontrolled hypertension* despite treatment with antihypertensives at the time of confirmation of IDEC-C2B8 treatment criteria<br>* ≥ 99th percentile in the pediatric sex- and age- | Additions due to addition of Appendix 4 |

| Page | Section | Before change (version 1.0, 29 June 2018) | After change (version 1.1, 25 July 2018) | Reason for change |
|---|---|---|---|---|
| | | specific blood pressure norms table | specific blood pressure norms table[22] (Appendix 4) | |
| 54 | 9.6.1 Treatment criteria for IDEC-C2B8 (open-label period) treatment | (5) Patients with reduced renal function (estimated glomerular filtration rate** < 60 mL/min/1.73 $m^2$) at the time of confirmation of IDEC-C2B8 treatment criteria<br>** See the estimated glomerular filtration rate. | (5) Patients with reduced renal function (estimated glomerular filtration rate** < 60 mL/min/1.73 $m^2$) at the time of confirmation of IDEC-C2B8 treatment criteria<br>** See the estimated glomerular filtration rate[23)-25)] (Appendix 5). | Additions due to addition of Appendix 5 |
| 54 | 9.6.1 Treatment criteria for IDEC-C2B8 (open-label period) treatment | (9) Patients who have at least one of the following laboratory parameters at the time of confirmation of IDEC-C2B8 treatment criteria ・・・<br>4) AST (GOT) ≥ 2.5 times the upper limit of normal in the pediatric age-specific liver escape enzyme (GOT) norms table for subjects aged less than 21 years or ≥ 2.5 times the upper limit of normal at the study site for subjects aged 21 years or older<br>5) ALT (GPT) ≥ 2.5 times the upper limit of normal in the pediatric age-specific liver escape enzyme (GPT) norms table for subjects aged less than 21 years or ≥ 2.5 times the upper limit of normal at the study site for subjects aged 21 years or older | (9) Patients who have at least one of the following laboratory parameters at the time of confirmation of IDEC-C2B8 treatment criteria ・・・<br>4) AST (GOT) ≥ 2.5 times the upper limit of normal in the pediatric age-specific liver escape enzyme (GOT) norms table[26] (Appendix 6)<br>5) ALT (GPT) ≥ 2.5 times the upper limit of normal in the pediatric age-specific liver escape enzyme (GPT) norms table[26] (Appendix 7) | Additions due to addition of Appendix 6 and Appendix 7 |
| 59 | 11.1 Schedule of observations, tests, and investigations | Table 11-2 Study schedule in the open-label period (IDEC-C2B8 treatment)<br>– | Table 11-2 Study schedule in the open-label period (IDEC-C2B8 treatment)<br>Measurement of height/weight during the switching period | Addition due to missing description (addition of measurement points in Table 11-2) |
| 61 | 11.3.1 Immediately before study drug administration | Table 11-4 Investigation items immediately before study drug administration<br>– | Table 11-4 Investigation items immediately before study drug administration<br>Adverse event assessment | Addition due to missing description |
| 63 | 11.5 Investigations at | Table 11-6 Investigation items at the time of relapse | Table 11-6 Investigation items at the time of relapse | Addition of supplementary |

| Page | Section | Before change (version 1.0, 29 June 2018) | After change (version 1.1, 25 July 2018) | Reason for change |
|---|---|---|---|---|
| | the time of relapse during the blinded observation period | during the blinded observation period<br>Date of confirmation of relapse | during the blinded observation period<br>Date of confirmation of relapse*1<br>*1 This refers to the date when urinalysis is performed at the study site to confirm relapse (date of urinalysis). | explanation |
| 66 | 11.9 Investigations at the time of relapse during the open-label observation period | Table 11-10 Investigation items at the time of relapse during the open-label observation period<br>Date of relapse | Table 11-10 Investigation items at the time of relapse during the open-label observation period<br>Date of confirmation of relapse*1<br>*1 This refers to the date when urinalysis is performed at the study site to confirm relapse (date of urinalysis). | Correction of misdescription and addition of supplementary explanation |
| 89 | 26 References | － | 22. Saji T, Ishikawa S, Echizen H, Okada T, Ogawa S, Suzuki Y, et al. The Japanese Circulation Society. Guidelines for Diagnosis and Treatment of Cardiovascular Diseases (Report by the Joint Working Group in 2010 to 2011). Guidelines for Drug Therapy of Cardiac Diseases in Children. Guidelines for Diagnosis and Treatment of Cardiovascular Diseases 2012. Tokyo: 2012.167-187<br>23. Uemura O, Ishikura K, Gotoh Y, Honda M. Creatinine-based estimated glomerular filtration rate for children younger than 2 years. Clin Exp Nephrol. 2018;22: 483-484.<br>24.Uemura O, Nagai T, Ishikura K, Ito S, Hataya H, Gotoh Y, et al. Creatinine-based equations to estimate glomerular filtration rate in Japanese children and adolescents with chronic kidney disease. Clin Exp Nephrol. 2014;18: 626-633.<br>25. Japanese Society for Pediatric Nephrology. Clinical Practice Guidebook for Diagnosis and Treatment of Chronic Kidney Disease 2012. Jpn | Addition of references due to addition of Appendices 4 to 7 |

| Page | Section | Before change (version 1.0, 29 June 2018) | After change (version 1.1, 25 July 2018) | Reason for change |
|---|---|---|---|---|
| | | | J Nephrol. 2012; 54: 1031-1189.<br>26. Tanaka T. New Pocket Guide for Pediatric Normal Laboratory Values 2nd Edition; Tokyo: Jiho Inc.; 2014. | |
| 91 | 27 Appendices | － | Appendix 4. Pediatric sex- and age-specific blood pressure norms table[22)]<br>Appendix 5. Estimated glomerular filtration rate<br>Appendix 6. Pediatric age-specific liver escape enzyme (GOT) norms table[26)]<br>Appendix 7. Pediatric age-specific liver escape enzyme (GPT) norms table[26)] | Addition of Appendices 4 to 7 |

Date of creation: 27 May 2019

| Page | Section | Before change (version 1.1, 25 July 2018) | After change (version 1.2, 27 May 2019) | Reason for change |
|---|---|---|---|---|
| 18 | 2.2.1 Test drug IDEC-C2B8 | – | (2) For CD20-positive chronic lymphocytic leukemia<br>When co-administered with any other anti-neoplastic agent, the usual adult dosage is 375 mg/m$^2$ of rituximab (genetical recombination) administered by IV infusion as the initial dose and then 500 mg/m$^2$ for the second and subsequent doses administered once per cycle according to the dosing interval of the concomitant anti-neoplastic agent. Up to 6 doses may be administered. | Addition due to the revision of the package insert for Rituxan (additional approval of [Indications] and [Dosage and Administration]) |
| 18 | 2.2.1 Test drug IDEC-C2B8 | (3) Wegener's granulomatosis, microscopic polyangiitis, and chronic idiopathic thrombocytopenic purpura<br>The usual adult dosage is 375 mg/m$^2$ of rituximab (genetical recombination) administered by IV infusion once a week for 4 weeks. | (4) Granulomatosis with polyangiitis, microscopic polyangiitis, and chronic idiopathic thrombocytopenic purpura<br>The usual adult dosage is 375 mg/m$^2$ of rituximab (genetical recombination) administered by IV infusion once a week for 4 weeks. | Amendment associated with the revision of the package insert for Rituxan (change in terminology in the package insert) |
| 18 | 2.2.1 Test drug IDEC-C2B8 | (2) to (6) | (3) to (7) | Change of paragraph number due to addition of "(2) For CD20-positive chronic lymphocytic leukemia" |
| 41 | 7.3.1 Relapse of nephrotic syndrome | Relapse that meets the definition of recurrence in the study will be reported in the " Relapse Report." The date of relapse will be confirmed in the patient diary and recorded in the medical record. The relapse status will be entered into the EDC. | Relapse that meets the definition of recurrence in the study will be recorded in the medical record and the relapse status will be entered into the EDC. In addition, the date of relapse will be confirmed in the patient diary and recorded in the medical record. | Amendment to clarify the procedure |

| Page | Section | Before change (version 1.1, 25 July 2018) | After change (version 1.2, 27 May 2019) | Reason for change |
|---|---|---|---|---|
| 60 | 11.2 Observations, tests, and investigations at screening | Table 11-3 Observation, test, and investigation items at screening<br>Virology<br>HIV antibody, HBs antigen, HBs antibody, HBc antibody, and HCV antibody | Table 11-3 Observation, test, and investigation items at screening<br>Virology<br>HIV antibody, HBs antigen, HBs antibody, HBc antibody, and HCV antibody (and HBV-DNA quantification only for HBs antibody-positive patients) | Clarification of test parameter for HBs antibody-positive patients |
| 64 | 11.6 Investigations to confirm the treatment criteria for IDEC-C2B8 (open-label period) treatment | Table 11-7 Investigation items to confirm the treatment criteria for IDEC-C2B8 (open-label period) treatment<br>Virology*2<br>HIV antibody, HBs antigen, HBs antibody, HBc antibody, and HCV antibody | Table 11-7 Investigation items to confirm the treatment criteria for IDEC-C2B8 (open-label period) treatment<br>Virology*2<br>HIV antibody, HBs antigen, HBs antibody, HBc antibody, and HCV antibody (and HBV-DNA quantification only for HBs antibody-positive patients) | Clarification of test parameter for HBs antibody-positive patients |
| 65 | 11.7.1 Immediately before IDEC-C2B8 administration | Table 11-8 Investigation items immediately before IDEC-C2B8 administration<br>*2 Measured immediately before IDEC-C2B8 administration and 30 minutes after the end of IDEC-C2B8 administration | Table 11-8 Investigation items immediately before IDEC-C2B8 administration<br>*2 Measured within 30 minutes before IDEC-C2B8 administration and within 30 minutes after the end of IDEC-C2B8 administration | Clarification of blood collection timing |
| 71 | 14.1 Adverse events | An adverse event (AE) is any untoward medical event (symptom, sign, disease, or abnormal laboratory finding) that occurs in a subject after enrollment/assignment to this study, whether or not considered related to the investigational product. | An adverse event (AE) is any untoward medical event (symptom, sign, disease, or abnormal laboratory finding) that occurs in a subject after the first dose of investigational product, whether or not considered related to the investigational product. | Correction of misdescription of adverse event collection period |

Date of creation: 2 December 2019

| Page | Section | Before change (version 1.2, 27 May 2019) | After change (version 2.0, 2 December 2019) | Reason for change |
|---|---|---|---|---|
| 9 | 1.4 Target sample size and planned study period | Planned enrollment period: From November 2018 to March 2020 (17 months)<br>Planned study period:<br>Blinded period From November 2018 to March 2021 (29 months)<br>From November 2018 to September 2021 (35 months), including the open-label period | Planned enrollment period: From November 2018 to March 2021 (29 months)<br>Planned study period:<br>From November 2018 to September 2022 (47 months)<br>Blinded period From November 2018 to March 2022 (41 months) | Extension of planned enrollment period and planned study period to achieve the target sample size and change in description |
| 11 | 1.5 Study schedule | Table 1-5 Study schedule in the open-label period (IDEC-C2B8 treatment)<br>– | Table 1-5 Study schedule in the open-label period (IDEC-C2B8 treatment)<br>Measurement of blood pressure during the switching period | Addition due to missing description |
| 29 | 4.4 Planned study period | Planned enrollment period: From November 2018 to March 2020 (17 months)<br>Planned study period:<br>Blinded period From November 2018 to March 2021 (29 months)<br>From November 2018 to September 2021 (35 months), including the open-label period | Planned enrollment period: From November 2018 to March 2021 (29 months)<br>Planned study period:<br>From November 2018 to September 2022 (47 months)<br>Blinded period From November 2018 to March 2022 (41 months) | Extension of planned enrollment period and planned study period to achieve the target sample size and change in description |
| 45 | 9 Treatment plan | 9.1 Treatment before the start of the study<br>9.1.1 Treatment with prednisolone for the last relapse before enrollment | 9.1 Treatment with prednisolone for the last relapse before enrollment | Amendment associated with reconsideration of section title |
| 45 | 9.1 Treatment with prednisolone for the last relapse before enrollment | (1) 60 mg/m$^2$/day (up to 60 mg/day) in 3 divided doses every day | (1) 60 mg/m$^2$/day (up to 60 mg/day) in 3 divided doses every day (or in 2 divided doses if deemed necessary by the investigator) | Addition of the regimen |

| Page | Section | Before change (version 1.2, 27 May 2019) | After change (version 2.0, 2 December 2019) | Reason for change |
|---|---|---|---|---|
| 46 | 9.2.1 Method for preparing study drug | (1) The study drug will be diluted 10-fold with JP physiological saline to a final concentration of 1 mg/mL immediately before administration, and IV infusion will be completed within 24 hours of preparation.<br>(2) For subjects who need to restrict salt intake, 5% glucose for injection may be used instead of physiological saline. In addition, do not mix the diluted solution with other drugs. In addition, do not vigorously stir or foam the solution during dilution. | (1) The study drug will be diluted 10-fold with physiological saline or 5% glucose for injection to a final concentration of 1 mg/mL immediately before administration, and IV infusion will be completed within 24 hours of preparation.<br>(2) Do not mix the diluted solution with other drugs. In addition, do not vigorously stir or foam the solution during dilution. | Review of the method for preparing study drug |

<table>
<tr><th>Page</th><th>Section</th><th>Before change (version 1.2, 27 May 2019)</th><th>After change (version 2.0, 2 December 2019)</th><th>Reason for change</th></tr>
<tr>
<td>49</td>
<td>9.2.3 Pretreatment before study drug administration</td>
<td>(2) Oral antihistamine: D-chlorpheniramine maleate (e.g., POLARAMINE®)<br>1) Subjects with a height-based standard weight of ≥ 50 kg or subjects aged 16 years or older will receive an oral dose of 2.0 mg (tablets).<br>2) Subjects who do not fall under 1) or who cannot take tablets orally will receive syrup (content of 0.04%) at the following doses:<br>
<table>
<tr><th>Age</th><th>Dose</th><th>Age</th><th>Dose</th></tr>
<tr><td>2 to < 3 years</td><td>1.0 mL</td><td>8 to < 12 years</td><td>2.5 mL</td></tr>
<tr><td>3 to < 5 years</td><td>1.5 mL</td><td>12 to < 15 years</td><td>3.0 mL</td></tr>
<tr><td>5 to < 8 years</td><td>2.0 mL</td><td></td><td></td></tr>
</table>
</td>
<td>(2) Oral antihistamine: D-chlorpheniramine maleate (e.g., POLARAMINE®)<br>1) Subjects with a height-based standard weight of ≥ 50 kg or subjects aged 16 years or older will receive an oral dose of 2.0 mg (tablets).<br>2) Subjects who do not fall under 1) or who cannot take tablets orally will receive d-chlorpheniramine maleate in syrup or dry syrup at the following doses:<br>
<table>
<tr><th rowspan="2">Age</th><th colspan="3">Dose</th></tr>
<tr><th>D-chlorpheniramine maleate</th><th>Syrup (content of 0.04%)</th><th>Dry syrup (content of 0.2%)</th></tr>
<tr><td>2 to < 3 years</td><td>0.4 mg</td><td>1.0 mL</td><td>0.2 g</td></tr>
<tr><td>3 to < 5 years</td><td>0.6 mg</td><td>1.5 mL</td><td>0.3 g</td></tr>
<tr><td>5 to < 8 years</td><td>0.8 mg</td><td>2.0 mL</td><td>0.4 g</td></tr>
<tr><td>8 to < 12 years</td><td>1.0 mg</td><td>2.5 mL</td><td>0.5 g</td></tr>
<tr><td>12 to < 15 years</td><td>1.2 mg</td><td>3.0 mL</td><td>0.6 g</td></tr>
</table>
</td>
<td>Review of oral antihistamines for premedication</td>
</tr>
</table>

<table>
<tr><th>Page</th><th>Section</th><th>Before change (version 1.2, 27 May 2019)</th><th>After change (version 2.0, 2 December 2019)</th><th>Reason for change</th></tr>
<tr><td>51</td><td>9.3.2 Change of date of second study drug administration</td><td>9.3.2 Change of date of administration</td><td>9.3.2 Change of date of second study drug administration</td><td>Amendment associated with reconsideration of section title</td></tr>
<tr><td>52</td><td>9.4 Treatment with prednisolone for relapse during the blinded observation period</td><td>(1) 60 mg/m$^2$/day (up to 60 mg/day) in 3 divided doses every day (until negative urine protein dipstick for 3 days if deemed necessary by the investigator)</td><td>(1) 60 mg/m$^2$/day (up to 60 mg/day) in 3 divided doses every day (or in 2 divided doses until negative urine protein dipstick for 3 days if deemed necessary by the investigator)</td><td>Addition of the regimen and correction of misdescription</td></tr>
<tr><td>55</td><td>9.9 Relapse during the open-label observation period</td><td>－</td><td>In this study, relapse and date of relapse are defined as described below.<br>The investigator will assess relapse at the medical examination based on the results of urine protein test performed at the study site.<br><table><tr><td>Recurrence</td><td>Any of the following conditions requiring prednisolone treatment:<br>Morning urine protein dipstick ≥ 3+ (or ≥ 300 mg/dL in quantitative urine protein test) for 3 consecutive days<br>Urine protein dipstick ≥ 2+ (or ≥ 100 mg/dL in quantitative urine protein test) and serum albumin ≤ 3.0 g/dL</td></tr><tr><td>Date of recurrence</td><td>The first date of morning urine protein dipstick ≥ 3+ (or ≥ 300 mg/dL in quantitative urine protein test) for 3 consecutive days or date of urine protein dipstick ≥ 2+ (or ≥ 100 mg/dL in quantitative urine protein test) and serum albumin ≤ 3.0 g/dL</td></tr></table></td><td>Addition of procedures at the time of relapse during the open-label observation period</td></tr>
</table>

| Page | Section | Before change (version 1.2, 27 May 2019) | After change (version 2.0, 2 December 2019) | Reason for change |
|---|---|---|---|---|
| 55 | 9.9 Relapse during the open-label observation period | — | In the event of relapse during the open-label observation period, observations, investigations, and tests will be performed according to the study schedule in Table 11-2. Relapse will be treated with treatment considered best by the investigator (standard treatment) in accordance with the Clinical Practice Guideline for Pediatric Idiopathic Nephrotic Syndrome. | Addition of procedures at the time of relapse during the open-label observation period |
| 56 | 9.10 Discontinuation criteria for study or IDEC-C2B8 treatment | After discontinuation of study or IDEC-C2B8 treatment, the subject will remain in the study unless withdrawn from the study as specified in 9.10, "Withdrawal from the study," and the investigator will continue observations, investigations, and tests according to the study schedule (11.1 or 11.2). Subjects withdrawn from study or IDEC-C2B8 treatment due to toxicity or refusal by the subject/legal representative (9.9) will be followed up from the start day of study treatment to the end of the observation phase unless lost to follow-up. | After discontinuation of study or IDEC-C2B8 treatment, the subject will remain in the study unless withdrawn from the study as specified in 9.11, "Withdrawal from the study," and the investigator will continue observations, investigations, and tests according to the study schedule (11.1 or 11.2). Subjects withdrawn from study or IDEC-C2B8 treatment due to toxicity or refusal by the subject/legal representative (9.10) will be followed up from the start day of study treatment to the end of the observation phase unless lost to follow-up. | Revised description |
| 60 | 11.1 Schedule of observations, tests, and investigations | Table 11-2 Study schedule in the open-label period (IDEC-C2B8 treatment) | Table 11-2 Study schedule in the open-label period (IDEC-C2B8 treatment)<br>Measurement of blood pressure during the switching period | Addition due to missing description |
| 61 | 11.2 Observations, tests, and investigations at screening | *4 Urinalysis will be performed on the day of confirmation of remission. | *4 Urinalysis will be performed on the day of confirmation of remission (if informed consent is obtained after confirmation of remission, data collected from the day of informed consent to the day of enrollment will be used). | Addition of action to be taken when informed consent is obtained after confirmation of remission |

| Page | Section | Before change (version 1.2, 27 May 2019) | After change (version 2.0, 2 December 2019) | Reason for change |
|---|---|---|---|---|
| 65 | 11.6 Investigations to confirm the treatment criteria for IDEC-C2B8 (open-label period) treatment | – | Height<br>Vital signs: Blood pressure (systolic and diastolic) | Addition due to missing description |
| 69 | 11.12.1 Peripheral B cells | Measurement will be outsourced to a contract laboratory (SRL Medisearch Inc.) (using a dedicated tube of the contract laboratory). | Measurement will be outsourced to a contract laboratory (SRL, Inc.) (using a dedicated tube of the contract laboratory). | Amendment due to transfer of business from SRL Medisearch Inc. to SRL, Inc. |
| 69 | 11.12.2 Blood drug concentration | Samples will be collected by SRL Medisearch Inc. | Samples will be collected by SRL, Inc. | Amendment due to transfer of business from SRL Medisearch Inc. to SRL, Inc. |
| 70 | 11.12.3 HACA | Samples will be collected by SRL Medisearch Inc. | Samples will be collected by SRL, Inc. | Amendment due to transfer of business from SRL Medisearch Inc. to SRL, Inc. |

| Page | Section | Before change (version 1.2, 27 May 2019) | After change (version 2.0, 2 December 2019) | Reason for change |
|---|---|---|---|---|
| 76 | 14.3 Follow-up of adverse events | — | The investigator will follow up any AE occurring during the observation phase (from Day 1 of the blinded period to Day 365 of the blinded/open-label period) until it resolves or returns to the same grade as before the start of study treatment. Follow-up may be terminated when the Investigator considers further follow-up unnecessary. | Addition of follow-up of AEs |
| 76 | 14.3 Expedited reporting of adverse events | (1) If an AE is considered serious, the Investigator will complete the "Serious Adverse Event Report" and report the event to the head of the study site as soon as possible and to the clinical trial steering committee, regardless of causality.<br>(2) The clinical trial steering committee will review the SAE received from the investigator and provide the Investigators at the other study sites with information of this AE.<br>(omitted)<br>(8) The Investigator at the study site where the event occurs will report any additional information of the AE to the head of the study site as soon as possible and to the clinical trial steering committee. The clinical trial steering committee will communicate this additional information to the investigators at the other study sites and the investigational product provider. | (1) If an AE is considered serious, the investigator will complete the "Serious Adverse Event Report" and report the event to the head of the study site as soon as possible and to the clinical trial steering committee, regardless of causality.<br>(2) The clinical trial steering committee will review the SAE received from the investigator and provide the investigators at the other study sites with information of this AE.<br>(omitted)<br>(8) The investigator at the study site where the event occurs will report any additional information of the AE to the head of the study site as soon as possible and to the clinical trial steering committee. The clinical trial steering committee will communicate this additional information to the investigators at the other study sites and the investigational product provider. | Revised description |

| Page | Section | Before change (version 1.2, 27 May 2019) | After change (version 2.0, 2 December 2019) | Reason for change |
|---|---|---|---|---|
| 77 | 14.3.2 When other events (pregnancy and overdose, medication error, or misuse/abuse of investigational product) are identified | (2) Overdose<br>The investigational product is administered at a dose higher than specified in the protocol.<br>E.g., administration at a dose of > 500 mg/m$^2$ or 500 mg rather than at a dose of 375 mg/m$^2$ (up to 500 mg) | (2) Overdose<br>The investigational product is administered at a dose higher than specified in the protocol.<br>E.g., administration at a dose of > 375 mg/m$^2$ or 500 mg rather than at a dose of 375 mg/m$^2$ (up to 500 mg) | Correction of misdescription |
| 80 | 15.3.1 Primary analysis and assessment criteria | Cumulative relapse curve, median time to relapse, and proportion of subjects with relapse at each time point will be estimated by the Kaplan-Meier method, and 95% confidence intervals (CIs) will be calculated using the Greenwood formula. | Relapse-free curve, median relapse-free period, and relapse-free rate at each time point will be estimated by the Kaplan-Meier method, and 95% confidence intervals (CIs) will be calculated using the Greenwood formula. | Amendment due to change in diagram output format |
| 80 | 15.3.2 Analyses of secondary endpoints (efficacy endpoints) | (1) Time to treatment failure<br>For the time to treatment failure, an event chart will be plotted for each group to estimate cumulative incidence curve and proportion of subjects with an event at each time point by the Kaplan-Meier method, and between-group comparison will be performed by stratified log-rank test using the randomization factor. | (1) Time to treatment failure<br>For the time to treatment failure, event chart will be plotted for each group to estimate non-treatment failure rate curve and non-treatment failure rate at each time point by the Kaplan-Meier method, and between-group comparison will be performed by stratified log-rank test using the randomization factor. | Amendment due to change in diagram output format |
| 81 | 15.3.2 Analyses of secondary endpoints (efficacy endpoints) | (5) Relationship between the presence or absence of peripheral B-cell count normalization and relapse or AEs<br>(omitted)<br>A similar analysis will be performed for the presence or absence of peripheral B-cell count normalization versus the presence or absence of AEs at each time point. | (5) Relationship between the presence or absence of peripheral B-cell count normalization and relapse or AEs<br>(omitted)<br>A similar analysis will be performed for the presence or absence of peripheral B-cell count normalization versus the presence or absence of AEs at each time point. AEs in this analysis will be classified into two categories: all AEs and infections requiring treatment. | Amendment due to change in diagram output format |

| Page | Section | Before change (version 1.2, 27 May 2019) | After change (version 2.0, 2 December 2019) | Reason for change |
|---|---|---|---|---|
| 81 | 15.3.3 Sensitivity analysis | — | (4) Subgroup analysis relapse-free period<br>For the relapse-free period, which is the primary endpoint (3.1.1 and 13.1), cumulative relapse curve, median time to relapse, and proportion of subjects with relapse at each time point will be estimated by the Kaplan-Meier method for each subgroup, with each level of the randomization factor (frequent relapse and steroid dependence) as a subgroup, and between-group comparison will be performed by the log-rank test. In addition, the hazard ratio of treatment effect between the groups and its 95% CI will be estimated for each subgroup using the Cox proportional hazards model. | Amendment due to change in diagram output format |
| 81 | 15.3.4 Analyses of other endpoints | (1) Relapse-free period during the open-label period<br>For the relapse-free period from the day of confirmation of IDEC-C2B8 treatment criteria, cumulative relapse curve and median time to relapse will be estimated by the Kaplan-Meier method. | (1) Relapse-free period during the open-label period<br>For the relapse-free period from the day of confirmation of IDEC-C2B8 treatment criteria, relapase-free curve and median relapse-free period will be estimated by the Kaplan-Meier method. | Amendment due to change in diagram output format |
| 82 | 15.3.4 Analyses of other endpoints | (4) Relationship between HACA production and relapse or AEs<br>(omitted)<br>A similar analysis will be performed for the presence or absence of HACA production versus the presence or absence of AEs at each time point. | (4) Relationship between HACA production and relapse or AEs<br>(omitted)<br>A similar analysis will be performed for the presence or absence of HACA production versus the presence or absence of AEs at each time point. AEs in this analysis will be classified into two categories: all AEs and infections requiring treatment. | Amendment due to change in diagram output format |

| Page | Section | Before change (version 1.2, 27 May 2019) | After change (version 2.0, 2 December 2019) | Reason for change |
|---|---|---|---|---|
| 82 | 15.3.6 Analysis using a dataset including data from subjects who enter the open-label period (IDEC-C2B8 treatment) | (1) Relapse-free period<br>For the relapse-free period, event chart and Kaplan-Meier cumulative incidence curve will be plotted to estimate the proportion of subjects with an event at each time point and its 95% CI. | (1) Relapse-free period<br>For the relapse-free period, event chart and Kaplan-Meier relapse-free curve will be plotted to estimate the relapse-free rate at each time point and its 95% CI. | Amendment due to change in diagram output format |
| 102 | Appendix 6. Pediatric age-specific liver escape enzyme (GOT) norms table[26)] | <Boys><br>20 years: 30.0<br><br>For patients aged 21 years or older, apply the normal value for those aged 20 years. | <Boys><br>20 years: 32.0<br><br>The normal value is 10 to 40 (U/L) for males and females aged 21 years or older. | Correction of misdescription |

| Page | Section | Before change (version 1.2, 27 May 2019) | After change (version 2.0, 2 December 2019) | Reason for change |
|---|---|---|---|---|
| 103 | Appendix 7. Pediatric age-specific liver escape enzyme (GPT) norms table[26)] | <Boys><br>8 months: 50.0<br><Girls><br>0 months: 62.0<br>1 month: 64.0<br>2 months: 65.0<br>3 months: 66.0<br>4 months: 67.0<br>5 months: 68.0<br>6 months: 68.0<br>7 months: 67.5<br>8 months: 66.5<br>9 months: 65.5<br>10 months: 63.9<br>11 months: 61.5<br>1 year: 57.0<br>2 years: 50.0<br>3 years: 44.0<br>4 years: 41.5<br><br>For patients aged 21 years or older, apply the normal value for those aged 20 years. | <Boys><br>8 months: 50.5<br><Girls><br>0 months: 45.0<br>1 month: 50.0<br>2 months: 54.5<br>3 months: 56.0<br>4 months: 56.0<br>5 months: 55.5<br>6 months: 54.5<br>7 months: 53.0<br>8 months: 50.5<br>9 months: 48.0<br>10 months: 45.0<br>11 months: 42.0<br>1 year: 38.4<br>2 years: 34.0<br>3 years: 30.0<br>4 years: 28.0<br><br>The normal value is 5 to 40 (U/L) for males and females aged 21 years or older. | Correction of misdescription |

Date of creation: 11 May 2020

<table>
<tr><th>Page</th><th>Section</th><th>Before change (version 2.0, 2 December 2019)</th><th>After change (version 2.1, 11 May 2020)</th><th>Reason for change</th></tr>
<tr><td>18</td><td>2.2.1 Test drug IDEC-C2B8</td><td>(4) Granulomatosis with polyangiitis, microscopic polyangiitis, and chronic idiopathic thrombocytopenic purpura</td><td>(4) Granulomatosis with polyangiitis, microscopic polyangiitis, chronic idiopathic thrombocytopenic purpura, and acquired thrombotic thrombocytopenic purpura</td><td>Addition due to the revision of the package insert for Rituxan (additional approval of [Indications] and [Dosage and Administration])</td></tr>
<tr><td>50</td><td>9.2.3 Pretreatment before study drug administration</td><td>
<table>
<tr><th rowspan="2">Age</th><th colspan="3">Dose</th></tr>
<tr><th>D-chlorpheniramine maleate</th><th>Syrup (content of 0.04%)</th><th>Dry syrup (content of 0.2%)</th></tr>
<tr><td>2 to < 3 years</td><td>0.4 mg</td><td>1.0 mL</td><td>0.2 g</td></tr>
<tr><td>3 to < 5 years</td><td>0.6 mg</td><td>1.5 mL</td><td>0.3 g</td></tr>
<tr><td>5 to < 8 years</td><td>0.8 mg</td><td>2.0 mL</td><td>0.4 g</td></tr>
<tr><td>8 to < 12 years</td><td>1.0 mg</td><td>2.5 mL</td><td>0.5 g</td></tr>
<tr><td>12 to < 15 years</td><td>1.2 mg</td><td>3.0 mL</td><td>0.6 g</td></tr>
</table>
</td><td>
<table>
<tr><th rowspan="2">Age</th><th colspan="3">Dose</th></tr>
<tr><th>D-chlorpheniramine maleate</th><th>Syrup (content of 0.04%)</th><th>Dry syrup (content of 0.2%)</th></tr>
<tr><td>< 3 years</td><td>0.4 mg</td><td>1.0 mL</td><td>0.2 g</td></tr>
<tr><td>3 to < 5 years</td><td>0.6 mg</td><td>1.5 mL</td><td>0.3 g</td></tr>
<tr><td>5 to < 8 years</td><td>0.8 mg</td><td>2.0 mL</td><td>0.4 g</td></tr>
<tr><td>8 to < 12 years</td><td>1.0 mg</td><td>2.5 mL</td><td>0.5 g</td></tr>
<tr><td>12 to 15 years</td><td>1.2 mg</td><td>3.0 mL</td><td>0.6 g</td></tr>
</table>
</td><td>Amendment of age categories</td></tr>
</table>

| Page | Section | Before change (version 2.0, 2 December 2019) | After change (version 2.1, 11 May 2020) | Reason for change |
|---|---|---|---|---|
| 58 | 10.1.2 Prohibited medications and therapies | (2) Immunosuppressants or immunosuppressive alkylating agents, etc.,<br>excluding standard treatment given for treatment failure (7.3.2) | (2) Immunosuppressants or immunosuppressive alkylating agents, etc.,<br>excluding standard treatment given for treatment failure (7.3.2) or after relapse during the open-label observation period (9.9) | Addition of prohibited medications after relapse during the open-label observation period |
| 78 | 14.5 Major expected adverse reactions to the investigational product<br>(1) Clinically significant adverse reactions | 1) Anaphylactoid symptoms, pulmonary disorders, and cardiac disorders (incidence unknown)<br>Infusion reaction may manifest as hypotension, angioedema, hypoxemia, bronchospasm, pneumonia (including interstitial pneumonia and allergic pneumonia), obliterative bronchiolitis, pulmonary infiltration, acute respiratory distress syndrome, myocardial infarction, ventricular fibrillation, cardiogenic shock, etc.<br>(Important Precautions) Infusion reaction (symptoms: fever, chills, nausea, headache, pain, pruritus, rash, cough, feeling of collapse, angioedema, etc.) that often occurs during the first dose of Rituxan or within 24 hours after the start of infusion has been reported in approximately 90% of patients. These symptoms are usually minimal to moderate in severity and occur mainly during or after the first dose. Patients should be closely monitored. If an abnormality occurs, appropriate measures (e.g., administration of antipyretic analgesics and/or antihistamines) should be taken, and the patient should be closely monitored until the symptoms resolve. | 1) Infusion reaction (incidence unknown)<br>Infusion reaction (symptoms: fever, chills, nausea, headache, pain, pruritus, rash, cough, feeling of collapse, angioedema, etc.) that often occurs during infusion of Rituxan or within 24 hours after the start of infusion has been reported in approximately 90% of treated patients, and these symptoms are usually minimal to moderate in severity and occur mainly during or after the first dose. In addition, serious adverse reactions such as anaphylaxis, pulmonary disorders, and cardiac disorders (e.g., hypotension, angioedema, hypoxemia, bronchospasm, pneumonia [including interstitial pneumonia and allergic pneumonia], obliterative bronchiolitis, pulmonary infiltration, acute respiratory distress syndrome, myocardial infarction, ventricular fibrillation, cardiogenic shock) may occur. Serious infusion reaction has also been reported in patients pretreated with antihistamines, antipyretic analgesics, and/or corticosteroids. If an abnormality occurs, administration should be immediately discontinued, appropriate measures (e.g., oxygen inhalation, administration of vasopressors, bronchodilators, corticosteroids, antipyretic analgesics, and/or antihistamines) should be taken, and the patient should be closely monitored until the symptoms resolve. | Amendment associated with the revision of the package insert for Rituxan (revision based on the new instructions) |

| Page | Section | Before change (version 2.0, 2 December 2019) | After change (version 2.1, 11 May 2020) | Reason for change |
|---|---|---|---|---|
| 78 | 14.5 Major expected adverse reactions to the investigational product<br>(1) Clinically significant adverse reactions | 2) Tumor lysis syndrome (incidence unknown) | 2) Tumor lysis syndrome (incidence unknown)<br>If an abnormality occurs, administration should be immediately discontinued, appropriate measures (e.g., administration of physiological saline, antihyperuricemics, etc., dialysis) should be taken, and the patient should be closely monitored until the symptoms resolve. | Amendment associated with the revision of the package insert for Rituxan (revision based on the new instructions) |
| 78 | 14.5 Major expected adverse reactions to the investigational product<br>(1) Clinically significant adverse reactions | 3) Fulminant hepatitis or exacerbation of hepatitis due to hepatitis B virus (incidence unknown) | 3) Fulminant hepatitis or exacerbation of hepatitis due to reactivation of hepatitis B virus (incidence unknown)<br>Hepatic failure secondary to fulminant hepatitis or exacerbation of hepatitis due to reactivation of hepatitis B virus may occur and has been reported to be fatal in some cases. If an abnormality occurs, appropriate measures such as administration of antivirals should be immediately taken. | Amendment associated with the revision of the package insert for Rituxan (revision based on the new instructions) |
| 78 | 14.5 Major expected adverse reactions to the investigational product<br>(1) Clinically significant adverse reactions | 4) Hepatic function disorder and jaundice (0.1% to < 5%) | 4) Hepatic function disorder and jaundice (incidence unknown)<br>Hepatic function disorder and/or jaundice with abnormal liver function tests such as increased AST (13.0%), increased ALT (13.3%), increased Al-P (5.3%), and increased total bilirubin (5.0%) may occur. | Amendment associated with the revision of the package insert for Rituxan (revision based on the new instructions) |
| 78 | 14.5 Major expected adverse reactions to the investigational product<br>(1) Clinically significant adverse reactions | 5) Mucocutaneous symptoms (incidence unknown) | 5) Mucocutaneous symptoms (incidence unknown)<br>Oculomucocutaneous syndrome (Stevens-Johnson syndrome), toxic epidermal necrolysis (TEN), pemphigus-like symptoms, lichenoid dermatitis, and vesiculobullous dermatitis have been reported with fatal outcome in some cases. | Amendment associated with the revision of the package insert for Rituxan (revision based on the new instructions) |

| Page | Section | Before change (version 2.0, 2 December 2019) | After change (version 2.1, 11 May 2020) | Reason for change |
|---|---|---|---|---|
| 78 | 14.5 Major expected adverse reactions to the investigational product<br>(1) Clinically significant adverse reactions | 6) Pancytopenia (incidence unknown), leukopenia (5% to < 10%), neutropenia (≥ 10%), agranulocytosis (incidence unknown), and thrombocytopenia (< 5%) | 6) Cytopenia<br>Pancytopenia (incidence unknown), leukopenia (54.7%), neutropenia (51.7%), agranulocytosis (incidence unknown), and thrombocytopenia (18.3%) may occur, and serious cytopenia has been reported. It has been reported that neutropenia occurred more than 4 weeks after the last dose of Rituxan. | Amendment associated with the revision of the package insert for Rituxan (revision based on the new instructions) |
| 78 | 14.5 Major expected adverse reactions to the investigational product<br>(1) Clinically significant adverse reactions | 7) Infections (incidence unknown) | 7) Infections (43.7%)<br>Since serious bacterial, fungal, or viral infections (e.g., sepsis, pneumonia) may occur, patients should be closely monitored during and after treatment with Rituxan. | Amendment associated with the revision of the package insert for Rituxan (revision based on the new instructions) |
| 79 | 14.5 Major expected adverse reactions to the investigational product<br>(1) Clinically significant adverse reactions | 8) Progressive multifocal leukoencephalopathy (PML) (incidence unknown) | 8) Progressive multifocal leukoencephalopathy (PML) (incidence unknown)<br>Patients should be closely monitored during and after treatment with Rituxan. If symptoms such as disturbed consciousness, cognitive disorder, paralytic symptom (hemiplegia or quadriplegia), or language disorder occur, diagnostic MRI and cerebrospinal fluid examination should be performed, administration should be discontinued, and appropriate measures should be taken. | Amendment associated with the revision of the package insert for Rituxan (revision based on the new instructions) |
| 79 | 14.5 Major expected adverse reactions to the investigational product<br>(1) Clinically significant adverse reactions | 10) Cardiac disorders (incidence unknown) | 10) Cardiac disorders (15.7%)<br>Ventricular or atrial arrhythmia, angina pectoris, and myocardial infarction have been reported. | Amendment associated with the revision of the package insert for Rituxan (revision based on the new instructions) |

| Page | Section | Before change (version 2.0, 2 December 2019) | After change (version 2.1, 11 May 2020) | Reason for change |
|---|---|---|---|---|
| 79 | 14.5 Major expected adverse reactions to the investigational product<br>(1) Clinically significant adverse reactions | 11) Renal disorders (incidence unknown) | 11) Renal disorders (incidence unknown)<br>Renal disorders such as increased serum creatinine (1.3%) or increased BUN (2.3%) may occur, and renal disorders requiring dialysis have been reported. If decreased urine output or increased serum creatinine or BUN is observed, administration should be discontinued and appropriate measures should be taken. | Amendment associated with the revision of the package insert for Rituxan (revision based on the new instructions) |
| 79 | 14.5 Major expected adverse reactions to the investigational product<br>(1) Clinically significant adverse reactions | 12) Gastrointestinal perforation/obstruction (incidence unknown) | 12) Gastrointestinal perforation/obstruction (incidence unknown)<br>If an abnormality occurs, X-ray or CT scan should be performed immediately to determine the site of bleeding and the presence or absence of findings of perforation/obstruction, and appropriate measures should be taken. | Addition for consistency with the package insert for Rituxan |
| 79 | 14.5 Major expected adverse reactions to the investigational product<br>(1) Clinically significant adverse reactions | 13) Decreased blood pressure (incidence unknown) | 13) Decreased blood pressure (9.0%)<br>Blood pressure may decrease transiently. | Amendment associated with the revision of the package insert for Rituxan (revision based on the new instructions) |
| 79 | 14.5 Major expected adverse reactions to the investigational product<br>(1) Clinically significant adverse reactions | 14) Cranial nerve symptoms such as posterior reversible encephalopathy syndrome (incidence unknown) | 14) Cranial nerve symptoms such as posterior reversible encephalopathy syndrome (incidence unknown)<br>Posterior reversible encephalopathy syndrome (symptoms: seizure, headache, psychiatric symptoms, visual disturbance, hypertension, etc.) may occur. In addition, visual and hearing impairments such as blindness and deafness, and cranial nerve disorders such as sensory disturbance and facial paralysis have been reported within 6 months after the end of treatment with Rituxan. | Amendment associated with the revision of the package insert for Rituxan (revision based on the new instructions) |

<table>
<tr><th>Page</th><th>Section</th><th>Before change (version 2.0, 2 December 2019)</th><th>After change (version 2.1, 11 May 2020)</th><th>Reason for change</th></tr>
<tr>
<td>80</td>
<td>14.5 Major expected adverse reactions to the investigational product<br>(2) Other adverse reactions</td>
<td>1) Respiratory: Laryngopharyngitis, rhinitis, oropharyngeal discomfort, cough, respiratory disorder<br>2) Circulatory: Increased blood pressure, tachycardia, flushing<br>3) Gastrointestinal: Nausea/vomiting, abdominal pain, stomatitis, anorexia, diarrhea<br>4) Hypersensitivity: Pyrexia, chills, rash, pruritus, hot flush, arthralgia, urticaria<br>5) General: Pain, malaise, feeling of collapse, headache, heavy sweating, edema<br>6) Neuropsychiatric: Dysesthesia, numbness<br>7) Blood/coagulation: Anemia, increased fibrin degradation products [FDP, D-dimer], thrombocytosis<br>8) Kidney: Electrolyte abnormality<br>9) Liver: Increased ALT (GPT), increased AST (GOT), increased Al-P<br>10) Other: Increased CRP, increased LDH, decreased total protein, increased uric acid, conjunctivitis, herpes zoster, muscle twitching</td>
<td>
<table>
<tr><th></th><th>≥ 5%</th><th>< 5%</th><th>Incidence unknown</th></tr>
<tr><td>Respiratory</td><td>Laryngopharyngitis (28.7%), rhinitis (24.7%), oropharyngeal discomfort (15.3%), cough, respiratory disorder</td><td>Wheezing, epistaxis</td><td></td></tr>
<tr><td>Circulatory</td><td>Increased blood pressure (17.3%), tachycardia (11.3%), flushing</td><td>Palpitations, vasodilatation, peripheral ischemia, bradycardia</td><td></td></tr>
<tr><td>Gastrointestinal</td><td>Nausea/vomiting (23.0%), abdominal pain (11.7%), stomatitis (11.7%), anorexia, diarrhea</td><td>Dry mouth, constipation, tenesmus</td><td></td></tr>
<tr><td>Hypersensitivity</td><td>Pyrexia (47.3%), chills (21.7%), rash (20.3%), pruritus (19.3%), hot flush (15.3%), arthralgia, urticaria</td><td>Myalgia, influenza-like symptoms</td><td>Serum sickness</td></tr>
<tr><td>General</td><td>Pain (39.3%), malaise (23.7%), feeling of collapse (22.7%), headache (20.7%), heavy sweating (11.0%), edema</td><td>Chest pain, increased weight, asthenia</td><td></td></tr>
<tr><td>Neuropsychiatric</td><td>Dysesthesia, numbness</td><td>Dizziness, insomnia</td><td></td></tr>
<tr><td>Blood/ coagulation</td><td>Anemia (27.7%)</td><td>Eosinophilia</td><td>Increased fibrin degradation products [FDP, D-dimer], thrombocytosis</td></tr>
<tr><td>Kidney</td><td>Electrolyte abnormality</td><td>Increased BUN, increased creatinine</td><td></td></tr>
<tr><td>Liver</td><td>Increased ALT (13.3%), increased AST (13.0%) increased A1-P, increased total bilirubin</td><td></td><td></td></tr>
<tr><td>Other</td><td>Increased CRP (23.6%), increased LDH (15.0%), decreased total protein, increased uric acid, conjunctivitis, herpes zoster</td><td>Asteatotic eczema, administration site reaction (e.g., pain, swelling), decreased albumin, hiccups</td><td>Muscle twitching</td></tr>
</table>
Note) The incidences of ADRs were tabulated based on the results of a Japanese phase II clinical study in B-cell non-Hodgkin's lymphoma, a Japanese phase II clinical study in chronic lymphocytic leukemia, a Japanese phase III clinical study in refractory nephrotic syndrome, and open-label clinical studies in ABO-incompatible kidney transplantation.
</td>
<td>Amendment associated with the revision of the package insert for Rituxan (revision based on the new instructions)</td>
</tr>
</table>

| Page | Section | Before change (version 2.0, 2 December 2019) | After change (version 2.1, 11 May 2020) | Reason for change |
|---|---|---|---|---|
| 82 | 15.3.1 Primary analysis and assessment criteria | (omitted)<br>- | (omitted)<br>For reference, subgroup analysis will be performed on the FAS, with each level of the randomization factor for analysis (frequent relapse and steroid dependence) as a subgroup. In the subgroup analysis of relapse-free period, which is the primary endpoint, relapse-free curve, median relapse-free period, and relapse-free rate at each time point will be estimated by the Kaplan-Meier method for each subgroup, and between-group comparison will be performed by the log-rank test. In addition, the hazard ratio of treatment effect between the groups and its 95% CI will be estimated for each subgroup using the Cox proportional hazards model. | Addition associated with reconsideration of the description location (the description in 15.3.3, "Sensitivity analysis" is moved to this section) |
| 82 | 15.3.2 Analyses of secondary endpoints (efficacy endpoints) | (3) Time course of peripheral depleted B-cell count<br>For the time course of peripheral depleted B-cell count, time course of peripheral B-cell count will be plotted for each subject, and the proportion of subjects with peripheral B-cell count normalization and its 95% CI will be estimated for each group at each time point of the blinded period. | (3) Peripheral B-cell count<br>For the time course of peripheral B-cell count, time course of peripheral B-cell count will be plotted for each subject, and the proportion of subjects with peripheral B-cell count normalization and its 95% CI will be estimated for each group at each time point of the blinded period. | Amendment associated with reconsideration of the description |

| Page | Section | Before change (version 2.0, 2 December 2019) | After change (version 2.1, 11 May 2020) | Reason for change |
| --- | --- | --- | --- | --- |
| 83 | 15.3.2 Analyses of secondary endpoints (efficacy endpoints) | (5) Relationship between the presence or absence of peripheral B-cell count normalization and relapse or AEs<br>To investigate the relationship between the presence or absence of peripheral B-cell count normalization and relapse or AEs, a 2-by-2 contingency table of the presence or absence of peripheral B-cell count normalization versus the presence or absence of relapse at each time point will be constructed for Fisher's exact test, and the conditional odds ratio and its 95% CI based on the hypergeometric distribution will be estimated.<br>A similar analysis will be performed for the presence or absence of peripheral B-cell count normalization versus the presence or absence of AEs at each time point. AEs in this analysis will be classified into two categories: all AEs and infections requiring treatment. | (5) Relationship between the presence or absence of peripheral B-cell count normalization and the first relapse<br>To investigate the relationship between the presence or absence of peripheral B-cell count normalization and the first relapse, a 2-by-2 contingency table of the presence or absence of peripheral B-cell count normalization versus the presence or absence of the first relapse at each time point will be constructed for Fisher's exact test, and the conditional odds ratio and its 95% CI based on the hypergeometric distribution will be estimated.<br>(6) Relationship between the presence or absence of peripheral B-cell count normalization and the presence or absence of AEs<br>To investigate the relationship between the presence or absence of peripheral B-cell count normalization and the presence or absence of AEs, a 2-by-2 contingency table of the presence or absence of peripheral B-cell count normalization versus the presence or absence of AEs at each time point will be constructed for Fisher's exact test, and the conditional odds ratio and its 95% CI based on the hypergeometric distribution will be estimated.<br>AEs in this analysis will be classified into two categories: all AEs and infections requiring treatment. | Amendment associated with reconsideration of the description (clarification of analysis) |
| 83 | 15.3.3 Sensitivity analysis | (1) Sensitivity analysis with the day of first study drug administration (Day 1 of the blinded period) as the starting point | (1) Sensitivity analysis of the effect of using the day of first study drug administration (Day 1 of the blinded period) as the starting point | Amendment associated with reconsideration of the description (clarification of analysis) |

| Page | Section | Before change (version 2.0, 2 December 2019) | After change (version 2.1, 11 May 2020) | Reason for change |
|---|---|---|---|---|
| 83 | 15.3.3 Sensitivity analysis | (2) Sensitivity analysis with the day of enrollment/assignment as the starting point | (2) Sensitivity analysis of the effect of using the day of enrollment/assignment as the starting point | Amendment associated with reconsideration of the description (clarification of analysis) |
| 83 | 15.3.3 Sensitivity analysis | (3) Sensitivity analysis of relapse-free period | (3) Sensitivity analysis of the effect of changing the definition of relapse | Amendment associated with reconsideration of the description (clarification of analysis) |
| 83 | 15.3.3 Sensitivity analysis | (4) Subgroup analysis of relapse-free period<br>For the relapse-free period, which is the primary endpoint (3.1.1 and 13.1), relapse-free curve, median relapse-free period, and relapse-free rate at each time point will be estimated by the Kaplan-Meier method for each subgroup, with each level of the randomization factor (frequent relapse and steroid dependence) as a subgroup, and between-group comparison will be performed by the log-rank test. In addition, the hazard ratio of treatment effect between the groups and its 95% CI will be estimated for each subgroup using the Cox proportional hazards model. | — | Deletion associated with reconsideration of the description location (described in 15.3.1, "Primary analysis and assessment criteria") |
| 84 | 15.3.5 Analysis of safety | Occurrence of AEs and ADRs and laboratory parameters will be tabulated for the blinded period (including the switching period) and the open-label period. | Occurrence of AEs and ADRs, laboratory parameters, and vital signs will be tabulated for the blinded period (including the switching period) and the open-label period. | Amendment due to addition of analysis items |
| 84 | 15.3.5 Analysis of safety | (2) Laboratory parameters<br>1) Tabulation of laboratory (hematology, serum chemistry, and urinalysis) measurements | (2) Laboratory parameters and vital signs<br>1) Tabulation of laboratory parameter (hematology, serum chemistry, and urinalysis) and vital sign measurements | Amendment due to addition of analysis items |

| Page | Section | Before change (version 2.0, 2 December 2019) | After change (version 2.1, 11 May 2020) | Reason for change |
|---|---|---|---|---|
| 85 | 15.3.6 Analysis using a dataset including data from subjects who enter the open-label period (IDEC-C2B8 treatment) | (4) Laboratory parameters (hematology, serum chemistry, and urinalysis)<br>For laboratory parameters (hematology, serum chemistry, and urinalysis), summary statistics will be calculated at each time point. | (4) Laboratory parameters (hematology, serum chemistry, and urinalysis) and vital signs<br>For laboratory parameters (hematology, serum chemistry, and urinalysis) and vital signs, summary statistics will be calculated at each time point. | Amendment due to addition of analysis items |

Date of creation: 8 September 2020

| Page | Section | Before change (version 2.1, 11 May 2020) | After change (version 3.0, 8 September 2020) | Reason for change |
|---|---|---|---|---|
| 43 | 8.1.2 Procedures to ensure blinding during the blinded period | However, this excludes cases where the B-cell count has not normalized (normal level ≥ 5 cells/μL) at the time of emergency allocation code breaking or at the end of the subject's participation in the study. | − | Deletion associated with reconsideration of the procedures |
| 43 | 8.1.2 Procedures to ensure blinding during the blinded period | − | If any of the conditions for emergency allocation code breaking (8.3.1) is met, the peripheral B-cell count may be measured at the study site only after breaking of the emergency allocation code. | Addition associated with reconsideration of the procedures |
| 56 | 9.9 Relapse during the open-label observation period | Relapse<br>Any of the following conditions requiring prednisolone treatment:<br>[3] Morning urine protein dipstick ≥ 3+ (or ≥ 300 mg/dL in quantitative urine protein test) for 3 consecutive days<br>[4] Urine protein dipstick ≥ 2+ (or ≥ 100 mg/dL in quantitative urine protein test) and serum albumin ≤ 3.0 g/dL | Relapse<br>Any of the following conditions requiring prednisolone treatment:<br>[1] Morning urine protein dipstick ≥ 3+ (or ≥ 300 mg/dL in quantitative urine protein test) for 3 consecutive days<br>[2] Urine protein dipstick ≥ 2+ (or ≥ 100 mg/dL in quantitative urine protein test) and serum albumin ≤ 3.0 g/dL | Correction of misdescription |

| Page | Section | Before change (version 2.1, 11 May 2020) | After change (version 3.0, 8 September 2020) | Reason for change |
|---|---|---|---|---|
| 81 | 15.2 Target sample size and its rationale | According to Kaplan-Meier of relapse-free period in a non-Japanese open-label randomized controlled study in which pediatric patients with steroid-dependent nephrotic syndrome received rituximab at a single dose of 375 mg/m$^2$ in addition to steroids,[16)] the 1-year relpase-free rate was 0.66 in the rituximab group and 0 in the steroid monotherapy group. Assuming an exponential distribution and a 1-year relapse-free rate of 0.01 in the steroid monotherapy group based on the results of the above study, the hazard ratio is 0.09, with hazard of 0.42 in the rituximab group and 4.60 in the steroid monotherapy group. Assuming that the duration of the study will be a total of 29 months with a 17-month enrollment period and a 12-month blinded observation period, the number of subjects required to verify the superiority at a two-sided significance level of 5% and a power of 80% is estimated to be 10 per group. Assuming that the 1-year relapse-free rate in the rituximab group is 0.50, 0.40, and 0.30, the necessary number of subjects calculated under the same conditions is 12, 13, and 16 per group, respectively.<br>(continued on next page) | According to Kaplan-Meier plot of relapse-free period in a non-Japanese open-label randomized controlled study in which pediatric patients with steroid-dependent nephrotic syndrome received rituximab at a single dose of 375 mg/m$^2$ in addition to steroids,[16)] the 1-year relapse-free rate was 0.66 in the rituximab group and 0 in the steroid monotherapy group. Assuming an exponential distribution and a 1-year relapse-free rate of 0.01 in the steroid monotherapy group based on the results of the above study, the hazard ratio is 0.09, with hazard of 0.42 in the rituximab group and 4.60 in the steroid monotherapy group. The number of subjects required to verify the superiority over 12 months, the duration of participation of each subject in the study, at a two-sided significance level of 5% and a power of 80% is estimated to be 13 per group. Assuming that the 1-year relapse-free rate in the rituximab group is 0.50 and 0.40, the necessary number of subjects calculated under the same conditions is 15 and 17 per group, respectively.<br>(continued on next page) | Reconsideration/ addition due to incorrect conditions for calculating the target sample size<br>* The reason for the change is detailed on pages 4 to 5. |

| Page | Section | Before change (version 2.1, 11 May 2020) | After change (version 3.0, 8 September 2020) | Reason for change |
|---|---|---|---|---|
| 81 | 15.2 Target sample size and its rationale | (continued from previous page)<br>According to Kaplan-Meier plot of relapse-free period in a Japanese phase III placebo-controlled randomized study in which patients with complicated nephrotic syndrome (frequently relapsing or steroid-dependent) received rituximab at a dose of 375 mg/$m^2$ once a week for 4 weeks,[9] the 1-year relapse-free rate was 0.44 in the rituximab group and 0.04 in the placebo group. Assuming an exponential distribution based on the results of this study, the hazard ratio is 0.255, with hazard of 0.82 in the rituximab group and 3.21 in the placebo group. Assuming that the duration of the study will be a total of 29 months with a 17-month enrollment period and a 12-month blinded observation period, the number of subjects required to verify the superiority at a two-sided significance level of 5% and a power of 80% is estimated to be 16 per group. Assuming that the 1-year disease-free rate in the rituximab group is 0.40, 0.35, and 0.30, the necessary number of subjects calculated under the same conditions is 18, 20, and 24 per group, respectively.<br>While the planned study population is patients with non-complicated nephrotic syndrome, patients with the disease that may become complicated may be included. Based on the results of the Japanese phase III study in complicated nephrotic syndrome, therefore, a sample size of 20 subjects per group is selected to allow for dropouts. | (continued from previous page)<br>According to Kaplan-Meier plot of relapse-free period in a Japanese phase III placebo-controlled randomized study in which patients with complicated nephrotic syndrome (frequently relapsing or steroid-dependent) received rituximab at a dose of 375 mg/$m^2$ once a week for 4 weeks,[9] the 1-year relapse-free rate was 0.44 in the rituximab group and 0.04 in the placebo group. Assuming an exponential distribution based on the results of this study, the hazard ratio is 0.255, with hazard of 0.82 in the rituximab group and 3.21 in the placebo group. The number of subjects required to verify the superiority over 12 months, the duration of participation of each subject in the study, at a two-sided significance level of 5% and a power of 80% is estimated to be 24 per group.<br>The planned study population for the present study is patients with childhood-onset nephrotic syndrome diagnosed as non-complicated frequently relapsing or steroid-dependent. Although the disease may become complicated in some of these subjects, it may be more appropriate to refer to the results of the above non-Japanese study in pediatric patients with non-complicated steroid-dependent nephrotic syndrome.<br>Based on the above, the target sample size is determined to be 20 treated subjects per group, taking into account the uncertainty. | Reconsideration/ addition due to incorrect conditions for determining the target sample size<br>* The reason for the change is detailed on pages 4 to 5. |
| 96 | Appendix 1. Standard height/weight table in 2000 | — | —<br>(* Numeric details are omitted) | Correction of misdescription |

**Detailed reasons for change to 15.2, "Target sample size and its rationale"**

The target sample size was determined while the framework of the study was being established. According to the information at that time, the enrollment period and observation period were specified, and the sample size was designed on the assumption that the relapse-free period could be measured at the end of the observation period for all subjects. However, the protocol states that the primary endpoint is "relapse during the blinded observation period (Days 1 to 365 of the blinded period)" and that information on the presence or absence of relapse after Day 365 will not be collected from any subjects, including those enrolled early, even if they are still in the study. In August 2020, the statistical analysis manager noticed the inconsistency in the background calculations for designing the sample size.

Since the sample size was originally designed assuming a longer period of observation than described in the current protocol, the sample size should have been overestimated. As such, the extent of this effect was reviewed as described below.

The protocol states that the sample size was designed based on the results of a non-Japanese open-label randomized controlled study by Ravani et al. (2015) in which pediatric patients with non-complicated steroid-dependent nephrotic syndrome received rituximab at a single dose of 375 $mg/m^2$ in addition to steroids and a phase III placebo-controlled randomized study by Iijima et al. (2014) in which patients with complicated nephrotic syndrome (frequently relapsing or steroid-dependent) received rituximab at a dose of 375 $mg/m^2$ once a week for 4 weeks. After the protocol was prepared, Basu et al. (2018) reported a randomized controlled study of rituximab administered at a dose of 375 $mg/m^2$ twice versus tacrolimus in pediatric non-complicated steroid-dependent nephrotic syndrome, and the results from Basu et al. (2018) were also referred to in this review.

For the review, three assumptions were made regarding the 1-year relapse-free rate in the control group. The 1-year relapse-free rate in the control group could not be determined in the study by Ravani et al. (2015), because all subjects in the control group relapsed. In the study by Iijima et al. (2014), on the other hand, the 1-year relapse-free rate in the placebo group was 0.04. Since Basu et al. (2018) compared rituximab with active treatment, the 1-year relapse-free rate in the control group could not be referred to in this review. If the present study is conducted with 20 subjects per group as proposed and 1 subject in the placebo group remains relapse-free until Day 365, a 1-year relapse-free rate of 0.05 at least in the control group is required. Accordingly, the 1-year relapse-free rate was assumed to be 0.01, 0.04, and 0.05. In this review, a two-sided significance level of 5% and a power of 80% were used.

The review results are presented in Table 1.

Table 1 Sample size per group (assuming a two-sided significance level of 5% and a power of 80%)

| | Ravani et al. (2015) | Iijima et al. (2014) | Basu et al. (2018) |
|---|---|---|---|
| Study population | Pediatric non-complicated steroid-dependent nephrotic syndrome | Pediatric complicated frequently relapsing or steroid-dependent nephrotic syndrome | Pediatric non-complicated steroid-dependent nephrotic syndrome |
| Rituximab | 375 mg/m$^2$ single dose | 375 mg/m$^2$ 4 doses | 375 mg/m$^2$ 2 doses |
| 1-year relapse-free rate in the rituximab group | 0.66 | 0.44 | 0.90 |
| | Investigation under the above settings | | |
| Number of subjects required per group specified in the protocol version 2.1 (1-year relapse-free rate in the control group) | 10 subjects (0.01) | 16 subjects (0.04) | Not specified |
| Number of subjects required per group on the assumption that all subjects will be followed up for 365 days (1-year relapse-free rate in the control group) | 13 subjects (0.01)<br>16 subjects (0.04)<br>17 subjects (0.05) | 16 subjects (0.01)<br>24 subjects (0.04)<br>26 subjects (0.05) | 11 subjects (0.01)<br>13 subjects (0.04)<br>13 subjects (0.05) |

When the sample size is recalculated on the assumption that individual subjects will be followed up until Day 365, the number of subjects calculated based on the results from Iijima et al. (2014) appears to be insufficient. Based on the results of the study by Basu et al. (2018) in which the same number of doses of rituximab as in the present study were administered in non-complicated steroid-dependent nephrotic syndrome, however, the assumed 1-year relapse-free rate of 0.44 in the rituximab group described in the protocol seems too conservative. Since the results from Basu et al. (2018) were more optimistic than those from Ravani et al. (2015), it is not considered necessary to include the results from Basu et al. (2018) in the rationale.

Based on the above, it was decided to change the rationale for the sample size by stating that the studies by Ravani et al. (2015) and Iijima et al. (2014) were referred to with more weight on an assumption based on the results from Ravani et al. (2015). In addition, the review results indicate that 20 treated subjects per group may be necessary due to the uncertainty of the assumptions. Accordingly, the description of the target sample size is modified by describing it as the number calculated on the assumption that each subject will be followed up until Day 365 and changing it from 20 subjects per group to allow for dropouts to 20 treated subjects per group.

Date of creation: 1 April 2021

| Page | Section | Before change (version 3.0, 8 September 2020) | After change (version 3.1, 1 April 2021) | Reason for change |
|---|---|---|---|---|
| 78 | 14.5 Major expected adverse reactions to the investigational product<br>(1) Clinically significant adverse reactions | 4) Hepatic function disorder and jaundice (incidence unknown)<br>Hepatic function disorder and/or jaundice with abnormal liver function tests such as increased AST (13.0%), increased ALT (13.3%), increased Al-P (5.3%), and increased total bilirubin (5.0%) may occur. | 4) Hepatic function disorder and jaundice (incidence unknown)<br>Hepatic function disorder and/or jaundice with abnormal liver function tests such as increased AST (12.0%), increased ALT (12.3%), increased Al-P (4.8%), and increased total bilirubin (4.8%) may occur. | Amendment associated with the revision of the package insert for Rituxan |
| 78 | 14.5 Major expected adverse reactions to the investigational product<br>(1) Clinically significant adverse reactions | 6) Cytopenia<br>Pancytopenia (incidence unknown), leukopenia (54.7%), neutropenia (51.7%), agranulocytosis (incidence unknown), and thrombocytopenia (18.3%) may occur, and serious cytopenia has also been reported. | 6) Cytopenia<br>Pancytopenia (incidence unknown), leukopenia (56.0%), neutropenia (54.2%), agranulocytosis (incidence unknown), and thrombocytopenia (16.9%) may occur, and serious cytopenia has also been reported. | Amendment associated with the revision of the package insert for Rituxan |
| 78 | 14.5 Major expected adverse reactions to the investigational product<br>(1) Clinically significant adverse reactions | 7) Infections (43.7%) | 7) Infections (41.6%) | Amendment associated with the revision of the package insert for Rituxan |
| 79 | 14.5 Major expected adverse reactions to the investigational product<br>(1) Clinically significant adverse reactions | 10) Cardiac disorders (15.7%) | 10) Cardiac disorders (14.5%) | Amendment associated with the revision of the package insert for Rituxan |
| 79 | 14.5 Major expected adverse reactions to the investigational product<br>(1) Clinically significant adverse reactions | 11) Renal disorders (incidence unknown)<br>Renal disorders such as increased serum creatinine (1.3%) or increased BUN (2.3%) may occur, and renal disorders requiring dialysis have been reported. | 11) Renal disorders (incidence unknown)<br>Renal disorders such as increased serum creatinine (1.2%) or increased BUN (3.3%) may occur, and renal disorders requiring dialysis have been reported. | Amendment associated with the revision of the package insert for Rituxan |
| 79 | 14.5 Major expected adverse reactions to the investigational product<br>(1) Clinically | 13) Decreased blood pressure (9.0%) | 13) Decreased blood pressure (8.1%) | Amendment associated with the revision of the package insert for Rituxan |

<table>
<tr><th>Page</th><th>Section</th><th>Before change (version 3.0, 8 September 2020)</th><th>After change (version 3.1, 1 April 2021)</th><th>Reason for change</th></tr>
<tr><td></td><td>significant adverse reactions</td><td></td><td></td><td></td></tr>
<tr><td>80</td><td>14.5 Major expected adverse reactions to the investigational product<br>(2) Other adverse reactions</td><td>
<table>
<tr><th></th><th>≥ 5%</th><th>< 5%</th><th>Incidence unknown</th></tr>
<tr><td>Respiratory</td><td>Laryngopharyngitis (28.7%), rhinitis (24.7%), oropharyngeal discomfort (15.3%), cough, respiratory disorder</td><td>Wheezing, epistaxis</td><td></td></tr>
<tr><td>Circulatory</td><td>Increased blood pressure (17.3%), tachycardia (11.3%), flushing</td><td>Palpitations, vasodilatation, peripheral ischemia, bradycardia</td><td></td></tr>
<tr><td>Gastrointestinal</td><td>Nausea/vomiting (23.0%), abdominal pain (11.7%), stomatitis (11.7%), anorexia, diarrhea</td><td>Dry mouth, constipation, tenesmus</td><td></td></tr>
<tr><td>Hypersensitivity</td><td>Pyrexia (47.3%), chills (21.7%), rash (20.3%), pruritus (19.3%), hot flush (15.3%), arthralgia, urticaria</td><td>Myalgia, influenza-like symptoms</td><td>Serum sickness</td></tr>
<tr><td>General</td><td>Pain (39.3%), malaise (23.7%), feeling of collapse (22.7%), headache (20.7%), heavy sweating (11.0%), edema</td><td>Chest pain, increased weight, asthenia</td><td></td></tr>
<tr><td>Neuropsychiatric</td><td>Dysesthesia, numbness</td><td>Dizziness, insomnia</td><td></td></tr>
<tr><td>Blood/<br>coagulation</td><td>Anemia (27.7%)</td><td>Eosinophilia</td><td>Increased fibrin degradation products [FDP, D-dimer], thrombocytosis</td></tr>
<tr><td>Kidney</td><td>Electrolyte abnormality</td><td>Increased BUN, increased creatinine</td><td></td></tr>
<tr><td>Liver</td><td>Increased ALT (13.3%), increased AST (13.0%) increased A1-P, increased total bilirubin</td><td></td><td></td></tr>
<tr><td>Other</td><td>Increased CRP (23.6%), increased LDH (15.0%), decreased total protein, increased uric acid, conjunctivitis, herpes zoster</td><td>Asteatotic eczema, administration site reaction (e.g., pain, swelling), decreased albumin, hiccups</td><td>Muscle twitching</td></tr>
</table>
Note) The incidences of ADRs were tabulated based on the results of a Japanese phase II clinical study in B-cell non-Hodgkin's lymphoma, a Japanese phase II clinical study in chronic lymphocytic leukemia, a Japanese phase III clinical study in refractory nephrotic syndrome, and open-label clinical studies in ABO-incompatible kidney transplantation.</td><td>
<table>
<tr><th></th><th>≥ 5%</th><th>< 5%</th><th>Incidence unknown</th></tr>
<tr><td>Respiratory</td><td>Laryngopharyngitis (26.8%), rhinitis (23.2%), oropharyngeal discomfort (15.1%), cough, respiratory disorder</td><td>Wheezing, epistaxis</td><td></td></tr>
<tr><td>Circulatory</td><td>Increased blood pressure (17.2%), tachycardia (10.2%), flushing</td><td>Palpitations, vasodilatation, bradycardia, peripheral ischemia</td><td></td></tr>
<tr><td>Gastrointestinal</td><td>Nausea/vomiting (21.7%), abdominal pain (10.5%), stomatitis (10.5%), anorexia, diarrhea</td><td>Dry mouth, constipation, tenesmus</td><td></td></tr>
<tr><td>Hypersensitivity</td><td>Pyrexia (43.4%), chills (19.6%), pruritus (19.6%), rash (19.0%), hot flush (14.2%), arthralgia</td><td>Urticaria, myalgia, influenza-like symptoms</td><td>Serum sickness</td></tr>
<tr><td>General</td><td>Pain (36.7%), malaise (22.3%), feeling of collapse (21.4%), headache (19.3%), heavy sweating, edema</td><td>Chest pain, increased weight, asthenia</td><td></td></tr>
<tr><td>Neuropsychiatric</td><td>Dysesthesia, numbness</td><td>Dizziness, insomnia</td><td></td></tr>
<tr><td>Blood/coagulation</td><td>Anemia (25.3%)</td><td>Eosinophilia</td><td>Increased fibrin degradation products [FDP, D-dimer], thrombocytosis</td></tr>
<tr><td>Kidney</td><td>Electrolyte abnormality</td><td>Increased BUN, increased creatinine</td><td></td></tr>
<tr><td>Liver</td><td>Increased ALT (12.3%), increased AST (12.0%)</td><td>Increased Al-P, increased total bilirubin</td><td></td></tr>
<tr><td>Other</td><td>Increased CRP (22.7%), increased LDH (14.5%), decreased total protein</td><td>Increased uric acid, herpes zoster, conjunctivitis, asteatotic eczema, administration site reaction (e.g., pain, swelling), decreased albumin, hiccups</td><td>Muscle twitching</td></tr>
</table>
Note) The incidences of ADRs were tabulated based on the results of a Japanese phase II clinical study in B-cell non-Hodgkin's lymphoma, a Japanese phase II clinical study in chronic lymphocytic leukemia, a Japanese phase III clinical study in refractory nephrotic syndrome, and open-label clinical studies in ABO-incompatible kidney transplantation.</td><td>Amendment associated with the revision of the package insert for Rituxan</td></tr>
<tr><td>81</td><td>15.1 Analysis population</td><td>(2) Per protocol set (PPS)<br>A subset of subjects in the FAS who were determined by monitoring to be compliant with the protocol without violating the protocol during the blinded period</td><td>(2) Per protocol set (PPS)<br>A subset of subjects in the FAS, excluding those who violated the protocol during the blinded period</td><td>Correction of misdescription</td></tr>
</table>

Date of creation: 2 August 2021

| Page | Section | Before change (version 3.1, 1 April 2021) | After change (version 3.2, 2 August 2021) | Reason for change |
|---|---|---|---|---|
| 83 | 15.3.4 Analyses of other endpoints | (2) Time course of blood IDEC-C2B8 concentration<br>For the time course of serum IDEC-C2B8 concentration, the mean ± standard deviation of serum IDEC-C2B8 concentration after the start of IDEC-C2B8 administration will be plotted for all subjects treated with IDEC-C2B8. In addition, the pharmacokinetic parameters Cmax (μg/mL), T1/2 (h), mean residence time (h), and AUC (μg・h/mL) will be estimated. | (2) Time course of blood IDEC-C2B8 concentration<br>For the time course of serum IDEC-C2B8 concentration, the mean ± standard deviation of serum IDEC-C2B8 concentration after the start of IDEC-C2B8 administration will be plotted for all subjects treated with IDEC-C2B8. In addition, the pharmacokinetic parameters Cmax (ng/mL), T1/2 (h), mean residence time (h), AUC (μg・h/mL), clearance (L/h), and volume of distribution (L) will be estimated, and values in individual subjects as well as a summary of number of subjects, mean, standard deviation, minimum, median, and maximum will be presented. | Addition due to missing description of pharmacokinetic parameters and revised description |

Date of creation: 5 November 2021

| Page | Section | Before change (version 3.2, 2 August 2021) | After change (version 3.3, 5 November 2021) | Reason for change |
|---|---|---|---|---|
| 18 | 2.2.1 Test drug IDEC-C2B8 | (2) For CD20-positive chronic lymphocytic leukemia | (2) CD20-positive chronic lymphocytic leukemia | Revised description |
| 18 | 2.2.1 Test drug IDEC-C2B8 | (4) Granulomatosis with polyangiitis, microscopic polyangiitis, chronic idiopathic thrombocytopenic purpura, and acquired thrombotic thrombocytopenic purpura | (4) Granulomatosis with polyangiitis, microscopic polyangiitis, chronic idiopathic thrombocytopenic purpura, acquired thrombotic thrombocytopenic purpura, and systemic scleroderma | Amendment associated with the revision of the package insert for Rituxan |
| 78 | 14.5 Major expected adverse reactions to the investigational product<br>(1) Clinically significant adverse reactions | 4) Hepatic function disorder and jaundice (incidence unknown)<br>Hepatic function disorder and/or jaundice with abnormal liver function tests such as increased AST (12.0%), increased ALT (12.3%), increased Al-P (4.8%), and increased total bilirubin (4.8%) may occur. | 4) Hepatic function disorder and jaundice (incidence unknown)<br>Hepatic function disorder and/or jaundice with abnormal liver function tests such as increased AST (10.5%), increased ALT (10.8%), increased Al-P (4.2%), and increased total bilirubin (4.2%) may occur. | Amendment associated with the revision of the package insert for Rituxan |
| 78 | 14.5 Major expected adverse reactions to the investigational product<br>(1) Clinically significant adverse reactions | 6) Cytopenia<br>Pancytopenia (incidence unknown), leukopenia (56.0%), neutropenia (54.2%), agranulocytosis (incidence unknown), and thrombocytopenia (16.9%) may occur, and serious cytopenia has been reported. | 6) Cytopenia<br>Pancytopenia (incidence unknown), leukopenia (50.0%), neutropenia (48.2%), agranulocytosis (incidence unknown), and thrombocytopenia (15.0%) may occur, and serious cytopenia has also been reported. | Amendment associated with the revision of the package insert for Rituxan |
| 78 | 14.5 Major expected adverse reactions to the investigational product<br>(1) Clinically significant adverse reactions | 7) Infections (41.6%) | 7) Infections (45.0%) | Amendment associated with the revision of the package insert for Rituxan |
| 79 | 14.5 Major expected adverse reactions to the investigational product<br>(1) Clinically significant adverse reactions | 10) Cardiac disorders (14.5%) | 10) Cardiac disorders (12.6%) | Amendment associated with the revision of the package insert for Rituxan |

| Page | Section | Before change (version 3.2, 2 August 2021) | After change (version 3.3, 5 November 2021) | Reason for change |
|---|---|---|---|---|
| 79 | 14.5 Major expected adverse reactions to the investigational product<br>(1) Clinically significant adverse reactions | 11) Renal disorders (incidence unknown)<br>Renal disorders such as increased serum creatinine (1.2%) or increased BUN (3.3%) may occur, and renal disorders requiring dialysis have been reported. | 11) Renal disorders (incidence unknown)<br>Renal disorders such as increased serum creatinine (1.1%) or increased BUN (2.9%) may occur, and renal disorders requiring dialysis have been reported. | Amendment associated with the revision of the package insert for Rituxan |
| 79 | 14.5 Major expected adverse reactions to the investigational product<br>(1) Clinically significant adverse reactions | 13) Decreased blood pressure (8.1%) | 13) Decreased blood pressure (7.1%) | Amendment associated with the revision of the package insert for Rituxan |

| Page | Section | Before change (version 3.3, 5 November 2021) | After change (version 3.4, 14 January 2022) | Reason for change |
|---|---|---|---|---|
| 18 | 2.2.1 Test drug IDEC-C2B8 | － | (6) Refractory pemphigus vulgaris and pemphigus foliaceus The usual adult dosage is 1,000 mg/body of rituximab (genetical recombination) administered by IV infusion once a week for 2 weeks. | Amendment associated with the revision of the package insert for Rituxan |
| 78 | 14.5 Major expected adverse reactions to the investigational product (1) Clinically significant adverse reactions | 4) Hepatic function disorder and jaundice (incidence unknown) Hepatic function disorder and/or jaundice with abnormal liver function tests such as increased AST (10.5%), increased ALT (10.8%), increased Al-P (4.2%), and increased total bilirubin (4.2%) may occur. | 4) Hepatic function disorder and jaundice (incidence unknown) Hepatic function disorder and/or jaundice with abnormal liver function tests such as increased AST (10.0%), increased ALT (10.3%), increased Al-P (4.0%), and increased total bilirubin (4.0%) may occur. | Amendment associated with the revision of the package insert for Rituxan |
| 78 | 14.5 Major expected adverse reactions to the investigational product (1) Clinically significant adverse reactions | 6) Cytopenia Pancytopenia (incidence unknown), leukopenia (50.0%), neutropenia (48.2%), agranulocytosis (incidence unknown), and thrombocytopenia (15.0%) may occur, and serious cytopenia has been reported. | 6) Cytopenia Pancytopenia (incidence unknown), leukopenia (47.5%), neutropenia (45.8%), agranulocytosis (incidence unknown), and thrombocytopenia (14.3%) may occur, and serious cytopenia has been reported. | Amendment associated with the revision of the package insert for Rituxan |
| 78 | 14.5 Major expected adverse reactions to the investigational product (1) Clinically significant adverse reactions | 7) Infections (45.0%) | 7) Infections (43.0%) | Amendment associated with the revision of the package insert for Rituxan |
| 79 | 14.5 Major expected adverse reactions to the investigational product (1) Clinically significant adverse reactions | 10) Cardiac disorders (12.6%) | 10) Cardiac disorders (12.0%) | Amendment associated with the revision of the package insert for Rituxan |
| 79 | 14.5 Major expected adverse reactions to the investigational product (1) Clinically significant adverse reactions | 11) Renal disorders (incidence unknown) Renal disorders such as increased serum creatinine (1.1%) or increased BUN (2.9%) may occur, and renal disorders requiring dialysis have been reported. | 11) Renal disorders (incidence unknown) Renal disorders such as increased serum creatinine (1.0%) or increased BUN (2.8%) may occur, and renal disorders requiring dialysis have been reported. | Amendment associated with the revision of the package insert for Rituxan |

<table>
<tr><th>Page</th><th>Section</th><th>Before change (version 3.3, 5 November 2021)</th><th>After change (version 3.4, 14 January 2022)</th><th>Reason for change</th></tr>
<tr><td>79</td><td>14.5 Major expected adverse reactions to the investigational product<br>(1) Clinically significant adverse reactions</td><td>13) Decreased blood pressure (7.1%)</td><td>13) Decreased blood pressure (6.8%)</td><td>Amendment associated with the revision of the package insert for Rituxan</td></tr>
<tr><td>80</td><td>14.5 Major expected adverse reactions to the investigational product<br>(2) Other adverse reactions</td><td>
<table>
<tr><th></th><th>≥ 5%</th><th>< 5%</th><th>Incidence unknown</th></tr>
<tr><td>Respiratory</td><td>Laryngopharyngitis (30.3%), rhinitis (20.3%), oropharyngeal discomfort (13.2%), cough, respiratory disorder</td><td>Wheezing, epistaxis</td><td></td></tr>
<tr><td>Circulatory</td><td>Increased blood pressure (15.0%), tachycardia, flushing</td><td>Palpitations, vasodilatation, bradycardia, peripheral ischemia</td><td></td></tr>
<tr><td>Gastrointestinal</td><td>Nausea/vomiting (18.9%), stomatitis, abdominal pain, anorexia, diarrhea</td><td>Dry mouth, constipation, tenesmus</td><td></td></tr>
<tr><td>Hypersensitivity</td><td>Pyrexia (38.2%), chills (17.1%), pruritus (17.1%), rash (16.6%), hot flush (12.4%)</td><td>Arthralgia, urticaria, myalgia, influenza-like symptoms</td><td>Serum sickness</td></tr>
<tr><td>General</td><td>Pain (32.1%), malaise (19.7%), feeling of collapse (18.9%), headache (16.8%), heavy sweating, edema</td><td>Chest pain, increased weight, asthenia</td><td></td></tr>
<tr><td>Neuropsychiatric</td><td>Dysesthesia</td><td>Numbness, dizziness, insomnia</td><td></td></tr>
<tr><td>Blood/coagulation</td><td>Anemia (22.4%)</td><td>Eosinophilia</td><td>Increased fibrin degradation products [FDP, D-dimer], thrombocytosis</td></tr>
<tr><td>Kidney</td><td>Electrolyte abnormality</td><td>Increased BUN, increased creatinine</td><td></td></tr>
<tr><td>Liver</td><td>Increased ALT (10.8%), increased AST (10.5%)</td><td>Increased Al-P, increased total bilirubin</td><td></td></tr>
<tr><td>Other</td><td>Increased CRP (20.0%), increased LDH (12.1%)</td><td>Decreased total protein, herpes zoster, increased uric acid, conjunctivitis, asteatotic eczema, administration site reaction (e.g., pain, swelling), decreased albumin, hiccups</td><td>Muscle twitching</td></tr>
</table>
Note) The incidences of ADRs were tabulated based on the results of a Japanese phase II clinical study in B-cell non-Hodgkin's lymphoma, a Japanese phase II clinical study in chronic lymphocytic leukemia, a Japanese phase III clinical study in refractory nephrotic syndrome, a Japanese phase II clinical study in systemic scleroderma, and open-label clinical studies in ABO-incompatible kidney transplantation.
</td><td>
<table>
<tr><th></th><th>≥ 5%</th><th>< 5%</th><th>Incidence unknown</th></tr>
<tr><td>Respiratory</td><td>Laryngopharyngitis (28.8%), rhinitis (19.3%), oropharyngeal discomfort (12.5%), cough, respiratory disorder</td><td>Wheezing, epistaxis</td><td></td></tr>
<tr><td>Circulatory</td><td>Increased blood pressure (14.3%), tachycardia, flushing</td><td>Palpitations, vasodilatation, bradycardia, peripheral ischemia</td><td></td></tr>
<tr><td>Gastrointestinal</td><td>Nausea/vomiting (18.0%), stomatitis, abdominal pain, anorexia, diarrhea</td><td>Dry mouth, constipation, tenesmus</td><td></td></tr>
<tr><td>Hypersensitivity</td><td>Pyrexia (36.5%), chills (16.3%), pruritus (16.3%), rash (15.8%), hot flush (11.8%)</td><td>Arthralgia, urticaria, myalgia, influenza-like symptoms</td><td>Serum sickness</td></tr>
<tr><td>General</td><td>Pain (30.5%), malaise (18.8%), feeling of collapse (18.0%), headache (16.0%), heavy sweating, edema</td><td>Chest pain, increased weight, asthenia</td><td></td></tr>
<tr><td>Neuropsychiatric</td><td>Dysesthesia</td><td>Numbness, dizziness, insomnia</td><td></td></tr>
<tr><td>Blood/coagulation</td><td>Anemia (21.3%)</td><td>Eosinophilia</td><td>Increased fibrin degradation products [FDP, D-dimer], thrombocytosis</td></tr>
<tr><td>Kidney</td><td>Electrolyte abnormality</td><td>Increased BUN, increased creatinine</td><td></td></tr>
<tr><td>Liver</td><td>Increased ALT (10.3%), increased AST (10.0%)</td><td>Increased Al-P, increased total bilirubin</td><td></td></tr>
<tr><td>Other</td><td>Increased CRP (18.7%), increased LDH (11.3%)</td><td>Decreased total protein, herpes zoster, increased uric acid, conjunctivitis, asteatotic eczema, administration site reaction (e.g., pain, swelling), decreased albumin, hiccups</td><td>Muscle twitching</td></tr>
</table>
Note) The incidences of ADRs were tabulated based on the results of a Japanese phase II clinical study in B-cell non-Hodgkin's lymphoma, a Japanese phase II clinical study in chronic lymphocytic leukemia, a Japanese phase III clinical study in refractory nephrotic syndrome, a Japanese phase II clinical study in systemic scleroderma, a Japanese phase II clinical study in refractory pemphigus vulgaris and pemphigus foliaceus, and open-label clinical studies in ABO-incompatible kidney transplantation.
</td><td>Amendment associated with the revision of the package insert for Rituxan</td></tr>
</table>

| Page | Section | Before change (version 3.3, 5 November 2021) | After change (version 3.4, 14 January 2022) | Reason for change |
|---|---|---|---|---|
| 87 | 18.2.1 Explanatory matters | (16) Name, job title, and contact information of the Investigator | (16) Name and contact information of the investigator | Amendment associated with the partial revision of the Ministerial Ordinance on Good Clinical Practice for Drugs |
| 90 | 21.2 Compensation for injury | The chairperson of the clinical trial steering committee will be a policyholder, and the investigator, and study site will be insured under the above insurance. | The head of the study site to which the chairperson of the clinical trial steering committee belongs will be a policyholder, and the investigator, and study site will be insured under the above insurance. | Correction of misdescription of the policyholder |

# Long-term prognosis of patients with uncomplicated frequently-relapsing/steroid-dependent nephrotic syndrome who participated in the JSKDC10 clinical trial

Protocol

Version number: 1.0

Date of creation: February 1, 2023

Study Chair: Kandai Nozu

Study Coordinator: Tomoko Horinouchi

| Management of confidential information |
| --- |
| The study-specific protocol, information sheet/informed consent form, case report forms, and other documents (hereafter referred to as study-related information) are confidential and may be provided only to persons directly involved in the study (heads of the study site, clinical trial coordinating centre, clinical trial steering committee, investigators, clinical trial collaborators, investigational drug administrator, institutional review boards, and independent data and safety monitoring committee). Study-related information may not be disclosed to a third party or used for purposes other than the study unless written consent has been obtained from the chairperson of the clinical trial steering committee, except when the details of the study are explained to a patiient. |

**Table of Contents**

**I. Overview of the study .......... 2**
**1. Background of the study .......... 3**
**2. Purpose of the study .......... 3**
**3. Research methods .......... 3**
**3.1 Study design and overview .......... 3**
**3.2 Inclusion and exclusion criateria .......... 5**
**3.3 Treatment methods subject to observation .......... 6**
**3.4 Observation items .......... 6**
**3.5 Number of patients to be studied .......... 6**
**3.6 Rationale for the study population .......... 6**
**3.7 Outcome Evaluation .......... 6**
**3.8 Criteria/definitions in this study .......... 7**
**3.9 Statistical analysis .......... 7**
**3.10 Ethics .......... 8**
**3.11 Duration of the study .......... 9**
**3.12 Provision of samples and Information to other Institutions .......... 9**
**4. Procedures for obtaining informed consent .......... 10**
**5. Handling and coding of personal and other information .......... 11**
**6. Burdens incurred by research patients, anticipated risks and benefits, and measures to minimise burdens and risks .......... 12**
**6.1 Burden on research patients .......... 12**
**6.2 Anticipated risks .......... 12**
**6.3 Anticipated benefits .......... 12**
**6.4 Overall assessment and measures to minimise such burdens and risks .......... 12**
**7. Method of storage and disposal of samples and information .......... 13**
**8. Contents and method of reporting to the head of the research organisation .......... 13**
**9. Status of the researchers' conflicts of interest related to the research, such as sources of funding for the research .......... 13**
**10. Registration of the research plan .......... 14**
**11. Attribution of the research results and publication of the results .......... 14**
**12. Possibility of using the samples and information obtained in this study for future research .......... 14**
**13. Research organisation .......... 14**
**13.1 Research institutes .......... 14**
**13.2 Research collaborators .......... 14**
**13.3 Institutions that provide only existing samples and information .......... 14**
**13.4 Contact information and hours .......... 15**
**13.5 Contracted services .......... 15**
**14. Reference list .......... 15**

## I. Overview of the study

### i. Schema

Subjects: Patients with uncomplicated frequently relapsing/steroid-dependent nephrotic syndrome who participated in the JSKDC10 clinical trial

Retrospective study: Relapse, additional immunosuppressive treatment, serum immunoglobulin G (IgG) level

↓

Evaluate the medium- and long-term effects of rituximab treatment in the patients

↓

Examine the significance of rituximab treatment in the patients

### ii. Objective

To examine the significance of rituximab treatment in the patients

### iii. Subjects

Patients with uncomplicated frequently relapsing/steroid-dependent nephrotic syndrome who participated in the JSKDC10 clinical trial

### iv. Method

Send survey forms to institutions for data collection and analysis

### v. Research Period

Date of approval by the head of the research institution to perform the research to March 31, 2026

## 1. Background of the study

Many children with nephrotic syndrome respond to steroid therapy and achieve remission, but the disease relapses frequently and is often difficult to control. The JSKDC10 clinical trial is a multicentre, double-blind, placebo-controlled, randomised, parallel-group trial of rituximab in childhood-onset uncomplicated frequently relapsing or steroid-dependent nephrotic syndrome, primarily evaluating relapse-free survival during a blinded period (1 year). Notably, Ravani et al. (2020) reported in their European randomised controlled trial that 8 of 15 patients treated with rituximab remained in remission after 4 years.[1] Because the JSKDC10 clinical trial lasted only 1 year, the long-term benefit in patients who received rituximab in the trial and who were relapse-free cannot be definitively determined, and the results of Ravani et al.'s study (2020) cannot be confirmed. Additionally, although a mid- to long-term decrease in serum IgG has been reported with rituximab,[2] the extent of the decrease and its impact are unclear.

To evaluate the long-term efficacy of rituximab, studies of long-term benefit beyond 1 year after rituximab administration are needed to define the potential and limitations of rituximab treatment in paediatric nephrotic syndrome.

## 2. Purpose of the study

### 2.1 Main Objectives

To evaluate the relapse-free period beyond 1 year in the patients enrolled in JSKDC10.

### 2.2 Secondary objectives

To evaluate changes in serum IgG levels beyond 1 year in the patients enrolled in JSKDC10.

To investigate the status of additional immunosuppressive treatment, including rituximab, for relapse, beyond 1 year in the patients enrolled in JSKDC10.

### 2.3 Exploratory objectives

N/A

## 3. Research methods

### 3.1 Study design and overview

Research centre:

■Kobe University Hospital

Information:

■Use of existing information

■Use of existing samples and information

■Provide existing information to other organisations (domestic and international)

■Provide existing samples and information to other institutions (domestic and international)

■Obtain existing information from other organisations

■Obtain existing samples and information from other institutions

**Study design**

Participants in the JSKDC10 clinical trial will be included in the current study beyond 1 year after receiving

rituximab. Two surveys will be used in this study: one to determine the patients' status through December 31, 2022, and another to determine their status as of December 31, 2024. Both surveys will use existing data from each facility at each time point.

**JSKDC10 clinical trial summary**

A summary of the JSKDC10 clinical trial of rituximab (IDEC-C2B8) treatment for the patients in this study is as follows:

Title: A multicentre, double-blind, placebo-controlled, randomised, parallel-group trial of IDEC-C2B8 in childhood-onset uncomplicated frequently relapsing or steroid-dependent nephrotic syndrome
Clinical Trial Steering Committee: Kazumoto Iijima, Kandai Nozu, Mayumi Sako
Primary objective: To evaluate the efficacy and safety of the study drug administered at a dose of 357 $mg/m^2$ (up to 500 mg/dose) once a week for 2 weeks in patients with childhood-onset nephrotic syndrome (diagnosed as uncomplicated frequently relapsing or steroid-dependent nephrotic syndrome).

JSKDC10 clinical trial flowchart

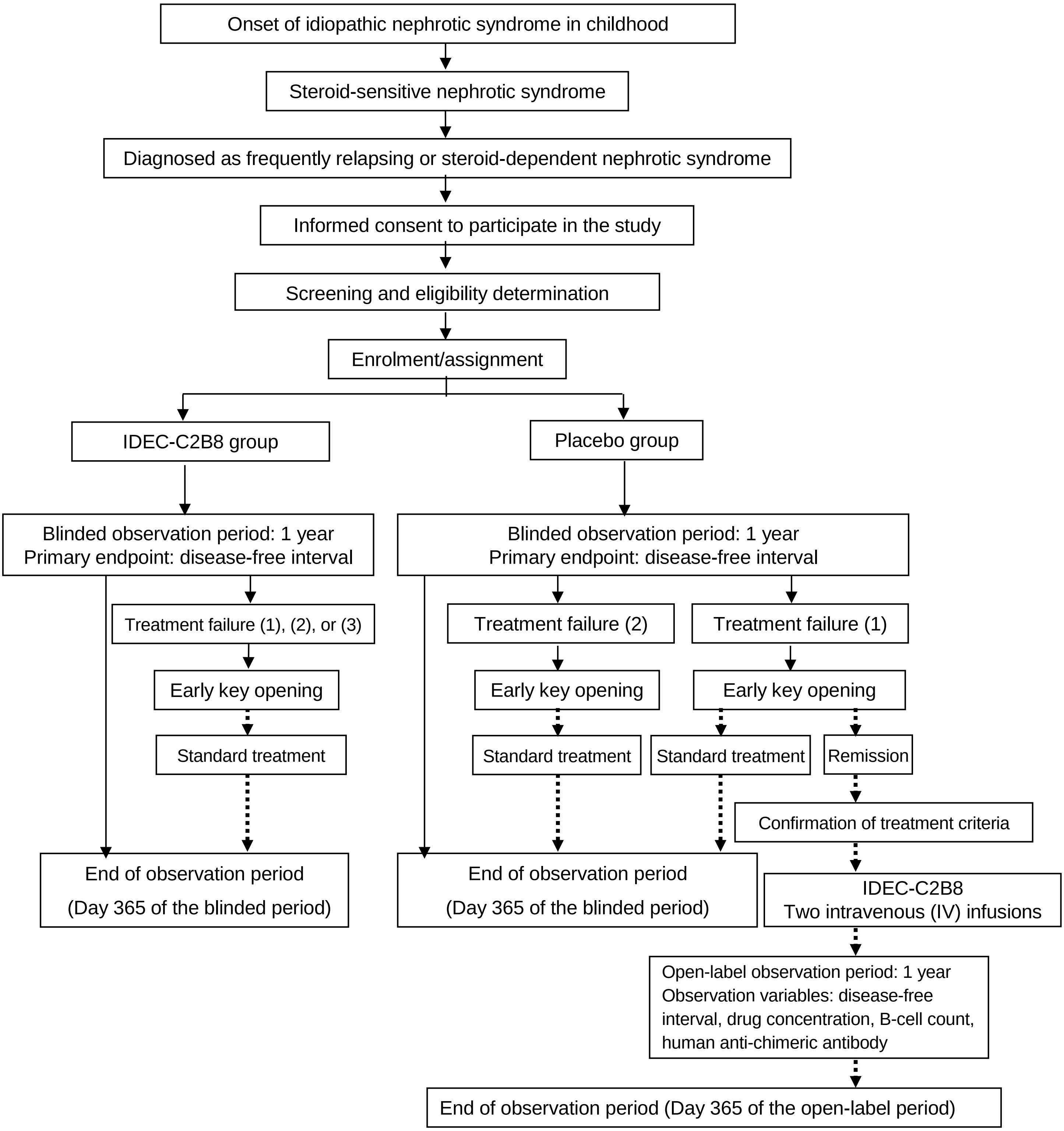


Primary endpoint: relapse-free period during the blinded observation period (Days 1–365 of the blinded period)

Secondary endpoints: time to treatment failure, total steroid dose, and change in peripheral B-cell count during the observation period (Days 1–365 of the blinded period and Days 1–365 of the open-label period)

Number of investigators: 40 (18 in the drug group and 22 in the placebo group)

Clinical Trial Period: November 2018 to September 2022

As shown in the figure above, in the JSKDC10 trial, patients in the placebo group may also receive rituximab if they wish, even if they relapse as Treatment failure (1). These patients will be followed for 1 year.

**3.2 Inclusion and exclusion criteria**

Patients who meet all of the following inclusion criteria and do not meet any of the following exclusion criteria are eligible for participation in the study:

#### 3.2.1 Inclusion criteria

(1) Patients who participated in the JSKDC10 clinical trial

(2) Patients for whom information on medical treatment and prognosis can be obtained from the medical record

#### 3.2.2 Exclusion criteria

(1) Patients who have requested not to participate in this study, based on publicly available information

(2) Patients who, in the opinion of the investigator, are ineligible to participate

### 3.3 Treatment methods subject to observation

Two doses of rituximab and an additional dose in the JSKDC10 study

### 3.4 Observation items

Obtain existing information on the following items:

(1) Relapse

Date of relapse diagnosis

Steroid sensitivity (sensitive/resistant, unknown*); *unknown: cases in which steroid pulse therapy is administered after < 4 weeks

Relapse within 2 weeks of the last dose of prednisolone (yes/no)

(2) Treatment

Immunosuppressive drugs (with or without treatment)

- Drug name, initiation date of administration

Rituximab (with or without treatment)

(3) Serum IgG level (if measured)

(4) Study Patients Identification code in the JSKDC10 study

### 3.5 Number of research patients to be studied

All participants in the JSKDC10 clinical trial (40 patients)

### 3.6 Rationale for the study population

The target number of patients for the JSKDC10 clinical trial was 40. If patients relapse in the placebo group, they may receive rituximab, if they wish.

### 3.7 Outcome evaluation

#### 3.7.1 Outcomes

##### 3.7.1.1 Key outcomes

Relapse-free period (period of time without relapse from the date of allocation or start of treatment)

#### 3.7.1.2 Secondary outcomes

As of the date recorded in the JSKDC10 study and as of December 31, 2022/December 31, 2024, the following are secondary outcomes:

(1) Relapse

(2) Relapse rate (times/person-years)

(3) Progression to frequent relapsing or steroid-dependent nephrotic syndrome or steroid-resistant nephrotic syndrome

(4) Additional immunosuppressant or rituximab treatment

(5) Serum IgG level

### 3.7.2 Exposure or predictors

Administration of rituximab

### 3.7.3 Confounding factors

Sex

Age

Steroid sensitivity at relapse

## 3.8 Criteria/definitions in this study

### 3.8.1 Relapse

Any of the following conditions requiring prednisolone treatment:

[1] Morning urine protein dipstick ≥ 3+ (or ≥ 300 mg/dl in quantitative urine protein testing) for 3 consecutive days

[2] Urine protein dipstick ≥ 2+ (or ≥ 100 mg/dl in quantitative urine protein testing) and serum albumin ≤ 3.0 g/dl

### 3.8.2 Date of relapse

The first date of morning urine protein dipstick ≥ 3+ (or ≥ 300 mg/dl in quantitative urine protein testing) for 3 consecutive days or date of urine protein dipstick ≥ 2+ (or ≥ 100 mg/dl in quantitative urine protein testing) and serum albumin ≤ 3.0 g/dl (or date of diagnosis of recurrence for the last three recurrences before enrolment)

### 3.8.3 Frequent relapse

At least two recurrences within 6 months of the first remission or at least four recurrences within any 12-month period

### 3.8.4 Steroid dependence

Two consecutive recurrences within 2 weeks after prednisolone dose reduction or discontinuation

### 3.8.5 Steroid resistance

Failure to achieve remission despite at least 4 weeks of daily prednisolone treatment at a dose of 60 $mg/m^2$/day

## 3.9 Statistical analysis

### 3.9.1 Analysis population

All enrolled study patients who meet the inclusion and exclusion criteria will be included.

### 3.9.2 Analysis of study patients' backgrounds

Summary statistics (number of cases, mean, standard deviation, minimum, median, maximum) will be calculated for continuous variables.

### 3.9.3 Analysis of the primary outcomes

The Kaplan–Meier method will be used to obtain the relapse-free rate after rituximab administration, to draw a Kaplan–Meier curve, and to estimate the median recurrence-free period and its 95% confidence interval. The 95% confidence interval of the recurrence-free rate will be obtained using Greenwood's formula.

### 3.9.4 Analysis of the secondary outcomes

The following will be analysed on the date recorded in the JSKDC10 clinical trial and on December 31, 2022/December 31, 2024, respectively:

(1) Relapse

Calculated as the percentage of patients with or without recurrence, with the 95% confidence interval, for all patients.

(2) Relapse rate (times/person-years)

Calculated by the recurrence rate (times/person-years) starting from the date of the first administration of rituximab.

(3) Progression to frequent relapsing or steroid-dependent nephrotic syndrome or steroid-resistant nephrotic syndrome

The same analysis as that for the primary endpoint will be performed for the duration of time without progression to frequent relapsing or steroid-dependent nephrotic syndrome or steroid-resistant nephrotic syndrome after rituximab treatment.

(4) Additional immunosuppressant or rituximab treatment

The proportion of additional immunosuppressant or rituximab treatment will be estimated, with its 95% confidence interval.

(5) Serum IgG level

For each patients, a list and figure of serum IgG levels over time after rituximab administration will be created.

### 3.9.5 Other analyses

N/A

## 3.10 Ethics

### 3.10.1 Approval at the start of the research and permission from the head of the research institution

The principal investigator shall consult with the Ethics Review Committee regarding the appropriateness of performing the research.

After obtaining the Ethical Review Committee’s opinion on the implementation of the research, the principal investigator will submit the results, and the documents submitted to the Ethical Review Committee and the documents required by the head of the research institution, to the head of the research institution and obtain permission for the implementation of the research.

Collaborating research institutions and institutions that provide only existing samples and information should notify the head of the institution prior to the provision of the information. If, in the judgment of the head of the institution, it is deemed desirable to refer the matter to the institution’s Ethics Review Committee, the research will be performed after obtaining the approval of that committee.

Prior to the start of the study, the following actions will be taken:

When it is determined that there is no need to undergo review by the institution’s Ethical Review Committee:

1. Notify and release the information disclosure document to the patient after modifying the document in accordance with the requirements of the relevant institution.
2. Submit the "Notification Form Concerning Provision of Existing Samples and Information to Other Research Institutions" (this form can be the institution's form) to the head of the institution and record this notification.
3. A copy of the "Notification Form for Provision of Existing Samples/Information to Other Research Institutions", stamped by the head of the affiliated institution, should be sent to the Research Secretariat.

In the case of review by the institution's Ethical Review Committee:

A copy of the notification of permission from the head of the relevant institution shall be sent to the Research Secretariat.

#### 3.10.2 Study modifications

When it becomes necessary to change the content of the research protocol, or make other changes, the principal investigator shall make the change promptly. After obtaining the Ethics Review Committee's opinion on the changes to the research protocol, or other changes, the principal investigator will submit the results of the review, and the documents submitted to the Ethics Review Committee and those required by the head of the research institution, to the head of the research institution and obtain permission for the implementation of the research.

#### 3.10.3 Completion of the study

When the research is terminated or discontinued, the principal investigator shall report a summary of the termination or discontinuation to the Ethical Review Committee and the head of the research institution without delay.

### 3.11 Study period

Date of approval by the head of the research institution to conduct the research to March 31, 2026

### 3.12 Provision of samples and information to other institutions

In this study, information collected at the participating institutions will be analysed by the representative research organisation/contractor.

(Analysis item and measurement institutions)
Department of Clinical Statistics, Kyoto University Graduate School of Medicine: Statistical Analysis
Department of Pediatrics, Kobe University Graduate School of Medicine: Data confirmation

In this study, this protocol and the "Table: Provision of information" shall be used as a substitute for records related to the provision of information. In this study, the principal investigator will keep such records in paper form at the Department of Pediatrics, Kobe University Hospital, and will substitute for the obligation of each participating institution to create and maintain records. Furthermore, the principal investigator will establish a system that allows for the verification of records upon request by each participating institution.

***Table*: Provision of information**

| | |
|---|---|
| Name of the research institution to which the information is provided | Department of Pediatrics, Kobe University Hospital |

| | |
|---|---|
| Name of the principal investigator at the research institution to which the information is provided | Kandai Nozu |
| Name of the principal investigator(s) at the research institution from which the donation is being made | (Refer to “13.3 Institutions that provide only existing samples/information”) |
| Information items | 3.4 Observations and tests and how they are performed. |
| Background of information acquisition | Obtained in the course of medical treatment at each participating institution |
| How to provide information | Provided by email |
| Status of obtaining consent from research patients | Opt-out |

| | |
|---|---|
| Name of the research institution to which the information is provided | Department of Clinical Statistics, Graduate School of Medicine, Kyoto University |
| Name of the person responsible for the research institution to which the information is provided | Takashi Omori |
| Name of the principal investigator(s) at the research institution from which the donation is being made | Kandai Nozu |
| Information items | 3.4 Observations and tests and how they are performed |
| Background of information acquisition | Collected from institutions that provide only existing samples and information |
| How to provide information | Provided by email |
| Status of obtaining consent from research patients | Opt-out |

## 4. Procedures for obtaining informed consent

Because this is an observational study in which existing information is used by Kobe University, existing information is provided to other institutions, and existing information is obtained from other institutions, we will not ask each research patient to provide written consent, but will provide a written release of information to any research patient with an opportunity for explanation after the date when the head of the research institution permits the research to proceed. However, for research patients with an opportunity for explanation after the date of approval of the research implementation by the head of the research institution, a disclosure of information document will be provided, an explanation will be given in an easy-to-understand manner, verbal consent will be obtained, and the notation will be entered in the medical record. Appropriate disclosure of information regarding this study will be made, and research patients ts will be given the opportunity to refuse enrolment in this study. Opportunities for refusal to participate in this study will be provided by e-mail or telephone.

Contents to be disclosed

(i) Purpose and method of use of the information (including the method of use if the information is provided to other organisations)

(2) Samples/information to be used or provided

(iii) Name of the organisation providing the information and the name of its head

(iv) Method of obtaining information to be provided

(v) Name of the principal investigator (or principal investigators, in the case of multi-institutional research) for the research who will use the provided information, and the name of the research institution to which said person belongs

(6) Scope of the persons who may use the information

(vii) Name or title of the person(s) responsible for the management of the information

(viii) A statement that the use or provision to other research institutions of the sample/information that identifies a research patient will be stopped at the request of the research patient.

(ix) Method of accepting requests from research patients, in (viii)

## 5. Handling and coding of personal and other information

### 5.1 Handling of personal information

All researchers involved in this research will conduct the research in compliance with the guidelines of the Declaration of Helsinki and the Ethical Guidelines for Life Sciences and Medical Research Involving Human Subjects.

The principal investigator or research assistant shall observe the principle of protecting the rights of research patients against invasion of privacy. Additionally, the persons concerned shall make their utmost efforts to protect the personal information and privacy of the research patients, and shall not divulge any personal information obtained in the course of conducting this research without justifiable reason. The same shall apply even after the persons concerned have retired from their positions. In this research, a list of research patient identification numbers will be used to link the research database and research-related documents with the original data of the research patients. Limited research patient information, such as sex, date of birth, and other information, may be used to identify research patients and verify the accuracy of the Research Patient Identification Number List, within the limits of all applicable laws and regulations.

When managing data, the principal investigator or research assistant shall delete code descriptions, and other personal data (e.g., name, initials, address, telephone number, medical record number) that may identify specific individuals. When registering cases and preparing case reports and other documents, the research patient identification number will be used. The list of research patient identification numbers to be coded should be stored and managed by the principal investigator in accordance with “7. Method of storage and disposal of samples and information.”

When the principal investigator releases information obtained through research, sufficient care should be taken to ensure that the research patients cannot be identified.

#### 5.1.1 Creation and management of research patient identification numbers

The responsible person at Kobe University and the institution(s) providing only existing information will prepare a list of research patient identification numbers to facilitate identification of the research patients. The responsible person at each institution will manage the information appropriately in accordance with the regulations of each institution, and will not provide the information to outside parties.

All reports and communications related to the research will be identified by a research patient identification number that codes the research patient.

#### 5.1.2 Preparation of case report forms or data sheets

The principal investigator or research assistant will review the eligibility criteria for the research patients and prepare a case report form (CRF) or other documentation. A list of patient identification codes will be prepared.

#### 5.1.3 Collection of case report forms or data sheets

##### 5.1.3.1 Electronic files of case report forms or data sheets

The principal investigator or research associate will provide an electronic file containing the same contents as those in the signed CRF or data sheet to the research office by e-mail or other means.

#### 5.1.4 Questionnaire inquiries and corrections

The research office will review the data and contact the principal investigator or research associate if any questions

arise. Whether a report to the head of the institution regarding modification of the CRF or an application for revision is required is subject to the rules of the collaborating institution.

#### 5.1.5 Data fixation

The Research Office will inquire about and revise the data, and fix the data of the relevant cases when any unclear points are confirmed.

### 5.2 Data analysis

The research office will perform the necessary analysis using the electronic files of the submitted CRFs.

### 5.3 Coding methods

For the purpose of confirming the willingness of research patients to participate in the study, information will be made public, and opportunities for refusal will be provided. At this time, information on research patients who have refused to participate will be managed such that specific individuals can be identified.

### 5.4 Explanation of results obtained from the study

Even if some results are obtained from the evaluation and analysis of this research, this is an exploratory or early stage of research, and its scientific reliability, including accuracy and certainty, cannot be fully established. Although the research patients have the right to know the results obtained in this study, we will not disclose the results because it may be detrimental to inform them of results or information that may not be scientific reliable.

### 5.5 Approach to disclosure of information on genetic mutations and incidental findings

There is no possibility of significant findings regarding genetic characteristics, in this study.

#### 5.5.1 Genetic counseling

Genetic counseling will not be provided in this study.

## 6. Burdens to be incurred by research patients, anticipated risks and benefits, and measures to minimise burdens and risks

### 6.1 Burden on research patients

Because this study uses only existing information, generally, there is no burden to the patients.

### 6.2 Anticipated risks

This is an observational study using existing information, and there is no burden or risk incurred by being a participant in this study.

### 6.3 Anticipated Benefits

This is an observational study using existing information and will not directly benefit the study patients.

### 6.4 Overall assessment and measures to minimise such burdens and risks

This is an observational study using existing information and is not expected to pose any burden or risk to the

research patients.

**7. Method of storage and disposal of samples and information**

In accordance with the "Guidelines Concerning the Retention Period of Research Data" at Kobe University Graduate School of Medicine, the principal investigator must retain important documents related to the implementation of the research (i.e., copies of applications to the Ethics Review Committee, notification documents from the head of the research institution, copies of various applications and reports, information disclosure documents and other data revision history, documents, or records that support information used in the research, such as entries in notebooks) and store them in a locked location for 10 years after the cessation or termination of the research or 10 years from the date of publication of the research results, such as papers, whichever is later. Thereafter, these documents will be destroyed using a method that does not allow identification of individuals.

**8. Contents and method of reporting to the head of the research organisation**

The principal investigator shall report the following in writing to the Ethical Review Committee and the head of the research institution. The report on (1) shall be made approximately once every 2 years, and the items after (2) shall be reported as appropriate:

1. Progress of the research
2. Facts or information that are obtained that undermine or may undermine the ethical validity or scientific rationality of the research and are considered to affect the continuation of the research
3. Facts or information that are obtained that undermine or may undermine the appropriateness of the conduct of the research or the credibility of the results of the research
4. When the research is terminated (suspended or discontinued)
5. When serious concerns arise from the perspective of respecting the human rights of research patients, or from the perspective of conducting the research, such as the leakage of information related to the research
6. Other

**9. Status of conflicts of interest related to the research, such as sources of funding for the research**

**9.1 Sources of funding for the research**

The research will be funded by the department to which the principal investigator belongs.

**9.2 Conflicts of interest related to the research**

The principal investigator, Kandai Nozu, has received a scholarship donation from Zenyaku Kogyo Corporation, which manufactures and markets the drug, rituximab, which will be used in this study.

The research partner, Kazumoto Iijima, has received a scholarship donation from Zenyaku Kogyo Corporation, which manufactures and markets the drug, rituximab, which will be used in this study.

The conflict of interest status of the principal investigator and research assistant(s) will be submitted to the Management Committee for Conflict of Interest in Clinical Research of Kobe University Graduate School of Medicine and approval will be obtained.

Each collaborating institution shall review and approve conflicts of interest in accordance with its own regulations.

## 10. Registration of the research plan

Prior to the implementation of the study, a summary of the study will be registered in a public database (Japan Registry of Clinical Trials (jRCT)) and updated as appropriate on the basis of changes in the research protocol and the progress of the study.

## 11. Attribution of research results and publication of results

The results, data, and intellectual property rights obtained from this study will belong to the Department of Pediatrics, Division of Internal Medicine, Kobe University Graduate School of Medicine. Specific handling and allocation will be determined upon consultation. Whether the intellectual property of the principal investigator of a joint research institute belongs to the individual or the research institute is subject to the agreement of the research institute to which the principal investigator belongs.

The results of this study will be presented at a conference and submitted as an article to an English-language professional journal. Presentations at domestic conferences will also be performed, as necessary.

In principle, the principal investigator will select the first author of the main publication of the research results in consultation with the person in charge of the research office. Thereafter, the co-authors will be selected in accordance with the restrictions associated with the submission rules of the paper. All co-authors must have reviewed the content of the paper prior to submission and agree to the content of the paper. If there is no agreement on the content after discussion, the principal investigator may choose not to include the disagreeing researcher as a co-author.

Because there may be multiple presentations at a conference, presentations will be made on a rotating basis by the person in charge of the research office, the principal investigator, or a person designated by the principal investigator of the collaborating institution with the highest number of registrations. The presenter will be determined with the approval of the principal investigator.

## 12. Possibility of using samples and information obtained in this study for future research

The data on research patients obtained in this study will be compiled into a database and may be used for other research in the future. When performing other research, a new research plan will be developed, and the research will be performed after obtaining approval from the Ethics Review Committee. In such cases, we will not seek written consent from individual research patients, but will disclose information on the research in an appropriate manner.

## 13. Research organisation

### 13.1 Research institutes

Department of Pediatrics, Division of Internal Medicine, Kobe University Graduate School of Medicine
Kandai Nozu (principal investigator); Telephone: +81-078-382-6090 (Medical Office)

### 13.2 Research collaborators

N/A

### 13.3 Institutions that will provide only existing samples and information

| | |
|---|---|
| Department of Pediatrics, Wakayama Medical University Hospital | Yuko Shima |
| Department of Pediatrics, Faculty of Medicine, Saga University | Masafumi Oka |
| Department of Pediatrics, Shiga University of Medical Science | Tomoyuki Sakai |
| Department of Pediatrics, Osaka University Graduate School of Medicine | Takuo Kubota |
| Department of Nephrology, Hyogo Prefectural Kobe Children's Hospital | Ryojiro Tanaka |
| Department of Pediatrics and Child Health, Nihon University School of Medicine | Tamaki Morohashi |
| Department of Nephrology, Tokyo Metropolitan Children's Medical Center | Riku Hamada |
| Department of Pediatrics, Yokohama City University Medical Center | Aya Inaba |
| Department of Nephrology, Rheumatology, National Center for Child Health and Development | Koichi Kamei |
| Department of Pediatrics, Dokkyo Medical University | Yuji Kano |
| Department of Nephrology, Saitama Children's Medical Center | Shuichiro Fujinaga |
| Department of Pediatrics and Child Health, Kurume University School of Medicine | Seiji Tanaka |

### 13.4 Contact information and hours

Contact information and hours of operation for the consultation service:

Department of Pediatrics, Kobe University Graduate School of Medicine
Contact person: Tomoko Horinouchi
7-5-1 Kusunokucho, Chuo-ku, Kobe 650-0017, Japan
TEL: 078-382-6090
FAX: 078-382-6099
E-mail: tohori@med.kobe-ac.jp
Hours: 10:00–17:00 (except Saturdays, Sundays, and holidays)

### 13.5 Contracted services

Department of Clinical Statistics, Graduate School of Medicine, Kyoto University
Specific Professor: Takashi Omori (statistical analysis)

## 14. References

1. Ravani P, Lugani F, Pisani I, et al. Rituximab for very low dose steroid-dependent nephrotic syndrome in children: a randomized controlled study. *Pediatr Nephrol* 2020. **35**: 1437–44.

2. Inoki Y, Kamei K, Nishi K, Sato M, Ogura M, Ishiguro A. Incidence and risk factors of rituximab-associated hypogammaglobulinemia in patients with complicated nephrotic syndrome. *Pediatr Nephrol* 2022; **37**: 1057–66.

Revision History

| Version number | Creation/Revision Date | Reason for Revision/Contents |
|---|---|---|
| Version 1.0 Edition | February 1, 2023 | First edition |
